# A two-sequence motif-based method for the inventory of gene families in fragmented and poorly annotated genome sequences

**DOI:** 10.1186/s12864-023-09859-4

**Published:** 2024-01-03

**Authors:** Anton Frisgaard Nørrevang, Sergey Shabala, Michael Palmgren

**Affiliations:** 1 NovoCrops Center, Department of Plant and Environmental Sciences, University of Copenhagen, Thorvaldsensvej 40, Frederiksberg C, DK-1871 Denmark; 2 School of Biological Sciences, University of Western Australia, Crawley, WA6009 Australia; 3 International Research Centre for Environmental Membrane Biology, Foshan University, Foshan, 528000 China

**Keywords:** Fragmented genomes, Gene families, Halophytes, P-type ATPase, Plasma membrane H^+^-ATPase, Two-motif

## Abstract

Databases of genome sequences are growing exponentially, but, in some cases, assembly is incomplete and genes are poorly annotated. For evolutionary studies, it is important to identify all members of a given gene family in a genome. We developed a method for identifying most, if not all, members of a gene family from raw genomes in which assembly is of low quality, using the P-type ATPase superfamily as an example. The method is based on the translation of an entire genome in all six reading frames and the co-occurrence of two family-specific sequence motifs that are in close proximity to each other. To test the method’s usability, we first used it to identify P-type ATPase members in the high-quality annotated genome of barley (*Hordeum vulgare*). Subsequently, after successfully identifying plasma membrane H^+^-ATPase family members (P3A ATPases) in various plant genomes of varying quality, we tested the hypothesis that the number of P3A ATPases correlates with the ability of the plant to tolerate saline conditions. In 19 genomes of glycophytes and halophytes, the total number of P3A ATPase genes was found to vary from 7 to 22, but no significant difference was found between the two groups. The method successfully identified P-type ATPase family members in raw genomes that are poorly assembled.

**Supplementary Information:**

The online version contains supplementary material available at 10.1186/s12864-023-09859-4.

## Introduction

Sequencing technology has progressed rapidly, and associated costs have declined, which has resulted in increased throughput in genome sequencing. Consequently, databases of sequenced genomes are now growing exponentially (RefSeq growth statistics [1]; GenBank and WGS Statistics [2]), and raw sequencing data are accumulating at an even greater rate (e.g., NCBI’s Sequence Read Archive [SRA] database [3]) [4, 5]. It has therefore become challenging to assemble and curate the genomes at the same pace as sequencing takes place [6]. For plants, even though published genomes are mostly complete with respect to the sequencing level, many of the assemblies are fragmented, with a scaffold N50 (the statistical value that defines assembly quality in terms of contiguity) that is below the standard 1 Mb [7]. A fragmented genome assembly will impact how well genes can be annotated, both in regards to entirely missing protein-coding genes and structural errors in the coding sequence (CDS) [8, 9]. Thus, it is challenging to identify all members of a gene family of interest in the predicted proteome of a published genome. This is also true in data sets where both sequence similarity and ab initio gene prediction have been used for predictions [10]. A limitation of using transcriptomic data to support the annotation is that gene family members often have a differential expression with respect to development, cell type, and changes in the environment, and the transcriptome may not be representative of all stages. For example, several members of the P3A plasma membrane (PM) H^+^-ATPase family from *Arabidopsis thaliana* are almost exclusively expressed in the gametophyte and not in the sporophyte [11].

Sequence alignment of related genes has identified sequence motifs that characterize members of specific gene families (e.g., as can be found in the Pfam database of protein families and domains [12]), and such motifs can be used as baits when searching new genomes for regions in genes that are similar to the identified sequence motifs. To derive such sequence profiles, or Hidden Markov Models (HMMs), from sequence alignments, methods such as HMMER [13] have been developed [14]. Some families or domains are defined not just by one motif but by the co-occurrence of two or more motifs. Whereas the presence of just one of these motifs may be insufficient to assign a protein to a particular family or domain, the simultaneous occurrence of linked motifs improves the confidence that the sequence belongs to the gene family being considered [15]. Alignment-free analytical methods have also been developed [16].

A problem with fragmented genomes is that they hinder the automatic annotation of genes. In this work, we developed a tool to identify members of gene families in fragmented genomes of plants that have not been fully annotated yet. The method is based on the translation of an entire genome in all six reading frames and the co-occurrence of two sequence motifs. We tested our method on P3A ATPases, a major family of PM H^+^-ATPases in plants. An advantage of our method is that it avoids using the predicted proteome/transcriptome for a given organism, which can potentially be misleading.

## Materials and methods

### Annotation of P-type ATPases in *Hordeum vulgare*

The inventory of P-type ATPase genes in the *H. vulgare* genome was based on identified homology to the *A. thaliana* and *Oryza sativa* inventories. Sequences for *A. thaliana* and *O. sativa* were retrieved from refs. [17, 18], respectively. The *H. vulgare* genome was retrieved from the IPK website together with high-confidence CDS and protein predictions.

To identify P-type ATPases in the *H. vulgare* cultivar Morex, the functional annotation of the high-confidence protein predictions on the IPK database [19] was first searched for proteins belonging to the PFAM00122 family (E1-E2 ATPase) [20]. The identified sequences were inspected for the DKTGT motif, and all sequences not containing this motif were discarded. Subsequently, the sequences were used for preliminary phylogenetic analysis to determine the closest homologs in *O. sativa* and *A. thaliana*. An alignment for each of the *H. vulgare* CDSs, the corresponding genomic sequence, and the CDS for the closest homolog from *O. sativa* was created to correct and check the identified ATPases (Supplementary files 1, 2, 3, 4, 5 and 6). This alignment was used to correct the CDSs by hand, compared to the *O. sativa* CDS, and by inspection of intron–exon splice sites.

### Phylogenetic analysis

Sequences were aligned in MEGA6 using multiple sequence comparison by log expectation (MUSCLE) [21]. The phylogenetic analysis was performed with the CIPRES Science Gateway [22]. For Maximum likelihood analysis, the RAxML-HPC2 workflow implemented in the XSEDE tool was used with the following parameters: bootstraps = 1,000; data type = protein; Protein Substitution Matrix = LG; and the rest of the settings were default. For Bayesian inference analysis, the MrBayes algorithm implemented in XSEDE 3.2.6 was used with the following parameters: number of generations = 100,000; data type = protein; Protein Substitution Matrix = LG; number of runs = 2; likelihood model = inverse gamma distribution; number of chains = 8; temperature parameter = 0.05; and the rest of the settings were default.

### Building the query sequences

The query sequences were created for each family (P1B, P2A, P2B, P3A, P4, and P5) using the ATPases from *A. thaliana*, *O. sativa*, and *H. vulgare* (Supplementary file 7) and were built by aligning all protein sequences for a family in Genius Prime using MUSCLE with standard parameters. From the alignment, two query sequences were constructed. The first query sequence consisted only of the family-specific motif, the superfamily-specific motif, and the distance between the two. The second query sequence contained the consensus surrounding the family- and superfamily-specific motifs.

### Genomes used for identifying P3A ATPases

All genomes used for determining the number of P3A PM H^+^-ATPases were reference genomes as of October 2021 and were retrieved from NCBI with the exception of *H. vulgare* and *Aeluropus littoralis*, for which the genomes were retrieved from IPK in October 2021. Links to all genomes and assemblies used are given in Supplementary Table 1.

### Translation of genomes and building and searching databases

To build the databases for interrogation of the genomes, all genomes were imported into CLC Main Workbench 20.0.4. Following this, the genomes were translated in all six reading frames and basic local alignment search tool (BLAST) databases were created in CLC Main Workbench for each of the now translated genomes. All BLAST searches were also carried out using CLC Main Workbench.

### cDNA cloning

Seeds of *H. vulgare* cv. RGT Planet were surface sterilized by removing the husk, submerging the seeds in a bleach solution (80% primo-bleach, 19% water, and 0.1% Triton X-100), and then placing the submerged seeds on a pivoting table for 20 min. After sterilization, the seeds were washed 10 times in sterilized Mili-Q water. Seeds were germinated on sterile wet filter paper for 3 days at 20 °C with 16 h of light. After germination, the seeds were moved to full MS plates and left to grow for 7 days at 20 °C with 16 h of light. Sterile seedlings were homogenized into a fine powder using a clean mortar submerged in liquid nitrogen, from which RNA was extracted with the Qiagen Plant RNeasy Kit following the manufacturer’s protocol. gDNA was removed with the TURBO DNA-free Kit from Ambion following the manufacturer’s instructions. cDNA was produced using the iScript kit from Bio-Rad according to the provided protocol.

### Identification and cloning of HvHMA7 and HvHMA1-like

The *HvHMA7* transcript was validated by partially cloning it from cDNA of 10-day-old whole seedlings of the cultivar RGT Planet using the following primer pairs: HvHMA7Fp2; 5’-TCCGCAACTGTCAATCT-3’ X HvHMA7Rp10; 5’-TCCTCAAAAAATGTCTTC-3’ and HvHMA7Fp8_BLAST; 5’-AGGCTGCCAACTGCATCAAT-3’ X HvHMA7Rp8_BLAST; 5’-CAAC GGGTAGCCCAACAATG-3’. *HvHMA1B* transcript expression was confirmed by cloning the first 1850 bp of the gene using the following primer pairs: HvHMA1BFp1_BLAST; 5’-ATGCGGCTTGACTCC-3’ X HvHMA1BRp1_BLAST; 5’-CATGCCCGCCTTTCAACAAA-3’ and HvHMA1BFp9; 5’-CAGGTGGTGCTAGGAACCTG-3’ X HvHMA1BRp9; 5’-CACTGGCACGTTGAGCTAGA-3’. All PCR products were sequenced by Eurofins Genomics.

### Statistics

Statistical analysis was performed using GraphPad Prism 9.4.0 using Student’s *t*-tests.

## Results

### Generating query sequences for different P-type ATPase families

P-type ATPases (also called E1-E2 ATPases) are a large superfamily of primary active pumps comprising several families in plants (P1B, P2A, P2B, P3A, P4, and P5). All P-type ATPases have the Asp-Lys-Thr-Gly-Thr (DKTGT) motif in the phosphorylation (P) domain where the phosphorylatable aspartate residue resides [23, 24]. Upstream of the DKTGT motif, each family also has a specific motif surrounding the proline helix breaker in transmembrane helix four, namely, P1B: CPC/SPC; P2A/B: PEXL; P3A: PIA; P4: PIS; and P5: PPXXP [24–27]. We thus considered that the two motifs in close proximity to each other could be used in combination to search the database of translated genomes. A search string for each family only containing the family- and ATPase-specific motifs with a fixed distance between the two was created (see Additional file 14) . We called this approach the two-motif search.

### A reference inventory of P-type ATPases in *H. vulgare*

Several P-type ATPases have been identified in *H. vulgare* [28–31], but an exhaustive inventory of this superfamily has yet to be constructed. To test our method, we started by using the high-quality assembled genome of *H. vulgare* (current N50 = 69.6 Mb). First, we identified and annotated all P-type ATPases using conventional methods. Subsequently, we searched through the *H. vulgare* genome using the above-described method.

**Fig. 1:**
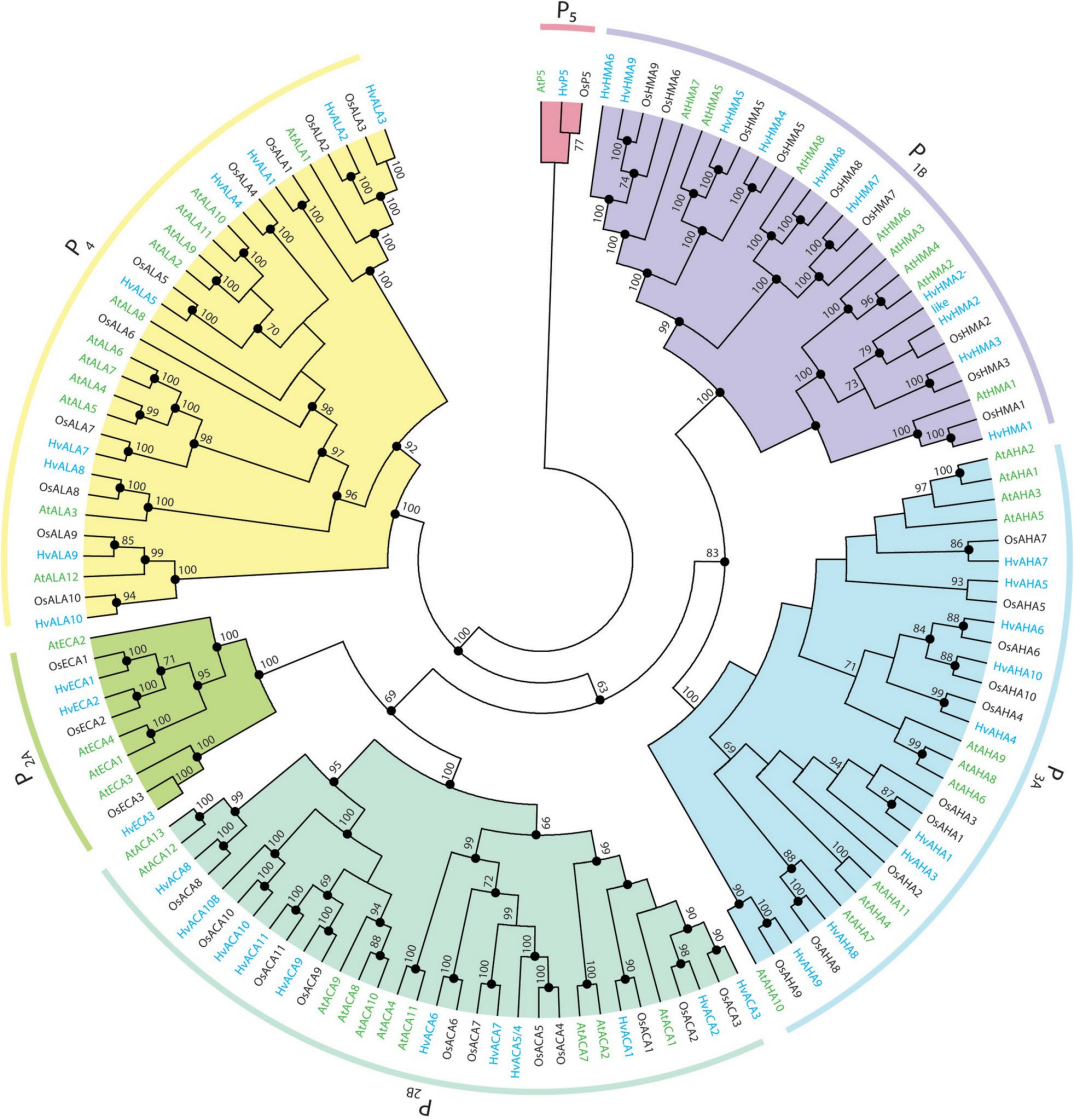
Phylogenetic analysis of P-type ATPases from *A. thaliana*, *H. vulgare*, and *O. sativa*. Full-length protein sequences were aligned in MEGA6 using MUSCLE with standard parameters. The alignment was then subjected to maximum likelihood analysis and Bayesian inference. For maximum likelihood analysis, the RAxML-HPC2 workflow implemented in the XSEDE tool was used on the CIPRES Science Gateway with the following parameters: bootstraps = 1000; data type = protein; Protein Substitution Matrix = LG; and the rest of the settings were default. For Bayesian inference analysis, the MrBayes v. 3.2.7 tool was used on the CIPRES Science Gateway with the following parameters: aamodelpr = fixed(lg); prset statefreqpr = fixed(empirical); lset rates = invgamma; mcmcp ngen = 100,000; mcmcp nruns = 2; mcmcp nchains = 8; mcmcp temp = 0.05; mcmcp mcmcdiagn = yes; and mcmc diagnfreq = 10,000. Numbers at nodes show likelihood from maximum likelihood analysis, and filled circles indicate full support in Bayesian inference analysis. In the phylogenetic analysis, the orthologue of OsALA6 was excluded as this gene is partial

P-type ATPase genes were annotated by retrieving all predictions with a PFAM00122 motif from the high-confidence predictions, which were curated guided by intron–exon splice sites and homology to their orthologues in *O. sativa*. The *HvHMA7* assembled transcript was only identified in Morex V3 and was difficult to curate properly. Therefore, the transcript was validated by partially cloning it from 10-day-old *H. vulgare* seedlings and searching the transcriptomic shotgun assembly (TSA) database at NCBI. All sequences are presented in Supplementary files 1, 2, 3, 4, 5 and 6. Phylogenetic analysis was performed to compare the ATPases found in *H. vulgare* with those of *O. sativa* and *A. thaliana*. *H. vulgare* sequences were named by adopting the name from the closest homolog in *O. sativa* (Fig. 1). HvPAA1 was renamed to HvHMA3 for consistency. A few differences could be found when comparing the number of P-type ATPase genes in *O. sativa* and *H. vulgare*. Two orthologues, *HvHMA2* and *HvHMA2-like*, were identified for *OsHMA2* in the P1B family. There also appeared to be two orthologues of *OsHMA1* in *H. vulgare*, *HvHMA1* and *HvHMA1-like*. The *HvHMA1-like* gene has a premature stop codon after codon number 80 and is therefore characterized as a pseudogene. The premature stop codon was confirmed by partially cloning *HvHMA1-like* from cDNA of 10-day-old seedlings of the cultivar RGT Planet and was also identified in the cultivar Price by searching the TSA database with the CDS of *HvHMA1-like*. In the P2B family, a second homolog of *OsACA10* was identified in *H. vulgare*, and a homolog of *OsACA4/OsACA5* was missing. Furthermore, in the P3A family no ortholog of *OsAHA2* could be identified in *H. vulgare*. For a complete list of the genes, see Table 1.

**Table 1:** Inventory of P-type ATPases in *H. vulgare*

Gene name	Morex V3 gene ID	Chromosome	Length (aa)	Status	Accession to the transcript on NCBI	Ref
**P1B**						
HvHMA1	HORVU.MOREX.r3.7HG0730400	7	828	Complete	XM_045100963.1	[28]
HvHMA2	HORVU.MOREX.r3.7HG0727750	7	1009	Complete	XM_045106273.1	[29]
HvHMA2-like	HORVU.MOREX.r3.7HG0727780	7	946	Complete	XM_045106438.1	
HvHMA3	HORVU.MOREX.r3.5HG0509930	5	837	Complete	XM_045128791.1	[30]
HvHMA4	HORVU.MOREX.r3.5HG0509930	5	980	Complete	XM_045095517.1	[29]
HvHMA5	HORVU.MOREX.r3.2HG0188860	2	995	Complete	XM_045111619.1	
HvHMA6	HORVU.MOREX.r3.6HG0571380	6	1001	Complete	XM_045095317.1	[29]
HvHMA7	HORVU.MOREX.r3.7HG0677890	7	891	Complete	XM_045099939.1^c^	
HvHMA8	HORVU.MOREX.r3.4HG0404710	4	904	Complete	XM_045126876.1	
HvHMA9	HORVU.MOREX.r3.7HG0738190	7	1002	Complete	XM_045102148.1	[29]
**P2A**						
HvECA1	HORVU.MOREX.r3.4HG0386480	4	1062	Complete	XM_045126035.1	
HvECA2	HORVU.MOREX.r3.1HG0007600	1	1049	Complete	XM_045127204.1	
HvECA3	HORVU.MOREX.r3.4HG0339640	4	1000	Complete	XM_045124294.1	
**P2B**						
HvACA1	HORVU.MOREX.r3.4HG0386480	4	1020	Complete	XM_045126605.1	
HvACA2	HORVU.MOREX.r3.5HG0438560	5	1020	Complete	XM_045092435.1	
HvACA3	HORVU.MOREX.r3.4HG0342980	4	1031	Complete	XM_045124470.1^c^	
HvACA4/5	HORVU.MOREX.r3.5HG0461290	5	1044	Complete	XM_045090434.1	
HvACA6	HORVU.MOREX.r3.3HG0317250	3	1035	Complete	XM_045117438.1	
HvACA7	HORVU.MOREX.r3.1HG0069030	1	1044	Complete	XM_045120351.1	
HvACA8	HORVU.MOREX.r3.1HG0077790	1	1022	Complete	XM_045106798.1	
HvACA9	HORVU.MOREX.r3.6HG0570250	6	1093	Complete	XM_045099510.1	
HvACA10	HORVU.MOREX.r3.7HG0682170	7	1083	Complete	XM_045100081.1^c^	
HvACA10-like	HORVU.MOREX.r3.5HG0492640	5	1079	Complete	XM_045094011.1	
HvACA11^b^	HORVU.MOREX.r3.2HG0192750	2	1083	Complete	XM_045111798.1	
**P3A**						
HvAHA1	HORVU.MOREX.r3.4HG0334970	4	962	Complete	XM_045123783.1	
HvAHA3	HORVU.MOREX.r3.5HG0420210	5	958	Complete	XM_045128668.1	
HvAHA4	HORVU.MOREX.r3.7HG0743890	7	952	Complete	XM_045104844.1	
HvAHA5	HORVU.MOREX.r3.1HG0065040	1	957	Complete	XM_045118633.1	
HvAHA6	HORVU.MOREX.r3.6HG0624610	6	950	Complete	XM_045096904.1	
HvAHA7	HORVU.MOREX.r3.2HG0202670	2	951	Complete	XM_045112242.1	
HvAHA8	HORVU.MOREX.r3.4HG0419450	4	970	Complete	XM_045127541.1^c^	
HvAHA9	HORVU.MOREX.r3.4HG0405790	4	908	Complete	XM_045126914.1	
HvAHA10	HORVU.MOREX.r3.7HG0658610	7	946	Complete	XM_045102180.1	
**P4**						
HvALA1	HORVU.MOREX.r3.4HG0378760	4	1247	Complete	XM_045125679.1	
HvALA2	HORVU.MOREX.r3.3HG0257460	3	1162	Complete	XM_045119932.1	
HvALA3	HORVU.MOREX.r3.4HG0379790	4	1121	Complete	XM_045125739.1	
HvALA4	HORVU.MOREX.r3.1HG0000110	1	1230	Complete	XM_045113689.1	
HvALA5	HORVU.MOREX.r3.7HG0676610	7	1205	Complete	XM_045105875.1	
HvALA6	HORVU.MOREX.r3.7HG0717230	7	1227	Partially	XM_045106260.1^c^	
HvALA7	HORVU.MOREX.r3.7HG0717230	7	1217	Complete	XM_045106260.1	
HvALA8	HORVU.MOREX.r3.1HG0034380	1	1238	Complete	XM_045112594.1	
HvALA9	HORVU.MOREX.r3.2HG0134710	2	1110	Complete	XM_045109393.1	
HvALA10	HORVU.MOREX.r3.2HG0160620	2	1172	Complete	XM_045110317.1	
**P5**						
P5	HORVU.MOREX.r3.1HG0063350	1	1175	Complete	XM_045117991.1	[31]
**Pseudogenes**						
	HORVU4Hr1G086810^a^	4		Pseudo gene		
HvHMA1-like	HORVU5Hr1G066370^a^	5		Pseudo gene		
	HORVU7Hr1G091830^a^	7		Pseudo gene		

^a^These genes do not have a gene ID in later versions of the *H. vulgare* genome, and the V1 ID is therefore adopted

^b^HvACA11 could potentially be duplicated in the genomic locations Nr 1: 605591154‐605603057 and Nr 2: 605666642‐605678539 on chromosome 7. However, the two genomic areas are highly identical and they are therefore viewed as one gene here

^c^The transcript was manually curated

### Evaluating the two-motif search in *A*. *thaliana*, *O*. *sativa*, and *H*. *vulgare*

Three genomes in which all P-type ATPases have been identified are now available, which allowed us to test the two-motif method. We searched the translated genomes of *A. thaliana*, *H. vulgare*, and *O. sativa* with the two-motif string and, with the exception of the P4 family, identified all P-type ATPases of a specific family that did not have an intron between the two motifs (Table 2). To identify all members of the P4 family, the search string was made more flexible to accept a distance between the two motifs that varied by 1 amino acid (Supplementary Fig. 5). As P2A and P2B have the same family motif, the two groups are indistinguishable and need to be added together.

**Table 2:** Comparison of ATPases identified by searching translated genomes with the two-motif method and the expanded two-motif method. The inventory of the respective species and the number of exons in which the query sequence is present

	Inventory	One exon	Two exons	Three exons	Two-motif	Expanded two-motif
P1B						
*A. thaliana*	8	4	3	1	4	6
*H. vulgare*	10	5	3	1	5	7
*O. sativa*	9	4	4	1	4	8
P2A						
*A. thaliana*	4	3	1	0	10	10
*H. vulgare*	3	2	1	0	9	9
*O. sativa*	3	2	1	0	10	10
P2B						
*A. thaliana*	10	7	0	3	10	11
*H. vulgare*	10	7	0	4	9	9
*O. sativa*	11	8	0	3	10	11
P3A						
*A. thaliana*	11	8	3	0	8	11
*H. vulgare*	9	5	4	0	5	9
*O. sativa*	10	5	5	0	5	10
P4						
*A. thaliana*	12	10	0	2	10	10
*H. vulgare*	10	7	0	3	6	6
*O. sativa*	10	7	0	3	7	7
P5						
*A. thaliana*	1	0	1	0	0	1
*H. vulgare*	1	0	1	0	1	1
*O. sativa*	1	0	1	0	0	1

### Modifying the two-motif method to include the area surrounding the family- and P-Type ATPase-specific motifs

As the two-motif method alone falls short regarding the identification of all ATPases within a group, we aimed to modify it by including the consensus sequence surrounding the family- and ATPase-specific motifs. To identify genes with an intron between the two motifs, it is necessary to build a bait sequence with additional information between the motifs. We modified the two-motif search and built an enlarged bait sequence for each family (P1B, P2A, P2B, P3A, P4, and P5) using the entire inventory of P-type ATPases from *A. thaliana*, *H. vulgare*, and *O. sativa*. We aligned sequences from the different families and produced a consensus sequence surrounding the DKTGT motif and family-specific motif for each family. The consensus sequences were some 55 to 70 amino acid residues in length (Supplementary Figs. 1-6). Each consensus sequence was used as a short query sequence to identify the number of genes in a specific family.

### Testing the enlarged query sequence on the annotated genomes of *A*. *thaliana,O*. *sativa*, and *H*. *vulgare*

To test the enlarged query sequence, we used the full inventories of P-type ATPases from *A. thaliana*, *O. sativa*, and *H. vulgare*. To avoid including the data used to build the query sequence, we rotated organisms out of the query sequence. Thus, for testing against *H. vulgare*, the query sequence would be created with *A. thaliana* and *O. sativa*. After construction, we used the sequence for a BLAST search against the translated genome of *H. vulgare*. The assumption was that if the DKTGT and the family-specific motifs were located in close proximity to each other and in the correct orientation relative to each other, the hit would be assessed as constituting a P-type ATPase of that family. This way, a gene can be identified even when the motifs are located in different exons.

The testing showed that all genes belonging to the P3A and P5 ATPase families could be scored (Table 2). These families have high overall conservation, which facilitated their identification (Supplementary Table 2 and Supplementary Figs. 4 and 6). Also, these families have the query sequence in two exons. The method identified fewer P1B, P2B, and P4 ATPases than what is present in the inventory. This could have been due to low overall conservation in the families or because several of the genes have the query sequence distributed onto three exons. Lastly, for P2A, the number is overestimated, most likely due to the close similarity between P2A and P2B (see Supplementary Figs. 2 and 3). If P2A and P2B are considered to be one family, all genes where the query sequence is found in one or two exons can be identified. A complete list of the number of exons the query sequences are located in is provided in Supplementary Table 3. Overall, it appears that the number of genes in a family can be relatively well predicted with the exception of genes where the query sequences are spread out onto three exons. To ensure that the result obtained for *H. vulgare* was consistent, we further tested *O. sativa* and *A. thaliana*, with results similar to those from *H. vulgare* (Table 2). Thus, we assumed that the method could be used to determine the number of P3A and P5 genes in a plant genome and estimate the number of P1B, P2A/B, and P4 genes.

### Identification of P3A ATPases in complete assemblies

To further evaluate the ability of our approach to correctly determine the number of P3A ATPases, several different well-sequenced and annotated genomes were retrieved and translated in all six reading frames, and a database was created for each. Using the constructed consensus sequence, the databases were interrogated for the number of P3A ATPases (Supplementary Table 4). The number obtained was compared to the number annotated in the genome found by performing BLAST searches using the protein sequence of AtAHA2 against the protein database for each species in the Kyoto Encyclopedia of Genes and Genomes (KEGG) [32]. From this analysis, it appears that the method can predict the number of P3A ATPases in a genome relatively well, and this applies to both very large genomes such as that of *Triticum aestivum* (genome size = 14.6 Gb) and smaller genomes such as that of *Beta vulgaris* (genome size = 540.5 Mb).

### Testing the method for the P2 family

We further tested the method in the *Glycine max* assembly, where P2 family ATPases have already been annotated [33]. The *G. max* genome assembly used here has a scaffold N50 of 48 Mb and a contig N50 of 419 kb. Twelve P2 Ca^2+^ ATPases were identified [33]. This is a surprisingly low number considering that *G. max* is a tetraploid [34] and that the diploid *A. thaliana* contains 14 P2 ATPases [18]. When searching the translated *G. max* assembly with the bait made from *O. sativa*, *A. thaliana*, and *H. vulgare*, 23 potential Ca^2+^ ATPases were identified (Supplementary Table 5), demonstrating that care must be taken when using protein prediction models.

### Comparing the number of P3A ATPases in glycophytes and halophytes

Under salt stress, plants extrude Na^+^ back into the soil to lower the K^+^/Na^+^ ratio. This extrusion is done in roots by Salt Overly Sensitive 1 (SOS1), which is a Na^+^/H^+^ antiporter, driven by the electrochemical H^+^ gradient generated by the PM H^+^-ATPase [35–38]. The uptake of K^+^ from the soil into roots, as well as K^+^ retention in the cytosol [39], through channel proteins and symporters is also dependent on the activity of the PM H^+^-ATPase [40]. Thus, we speculated that the number of PM H^+^-ATPases could contribute to the difference between halophytes (salt-tolerant plants) and glycophytes (salt-sensitive plants) with respect to salt tolerance as an expansion of genes and their subsequent diversification could possibly enable a more fine-tuned regulation at the tissue expression level.

**Fig. 2:**
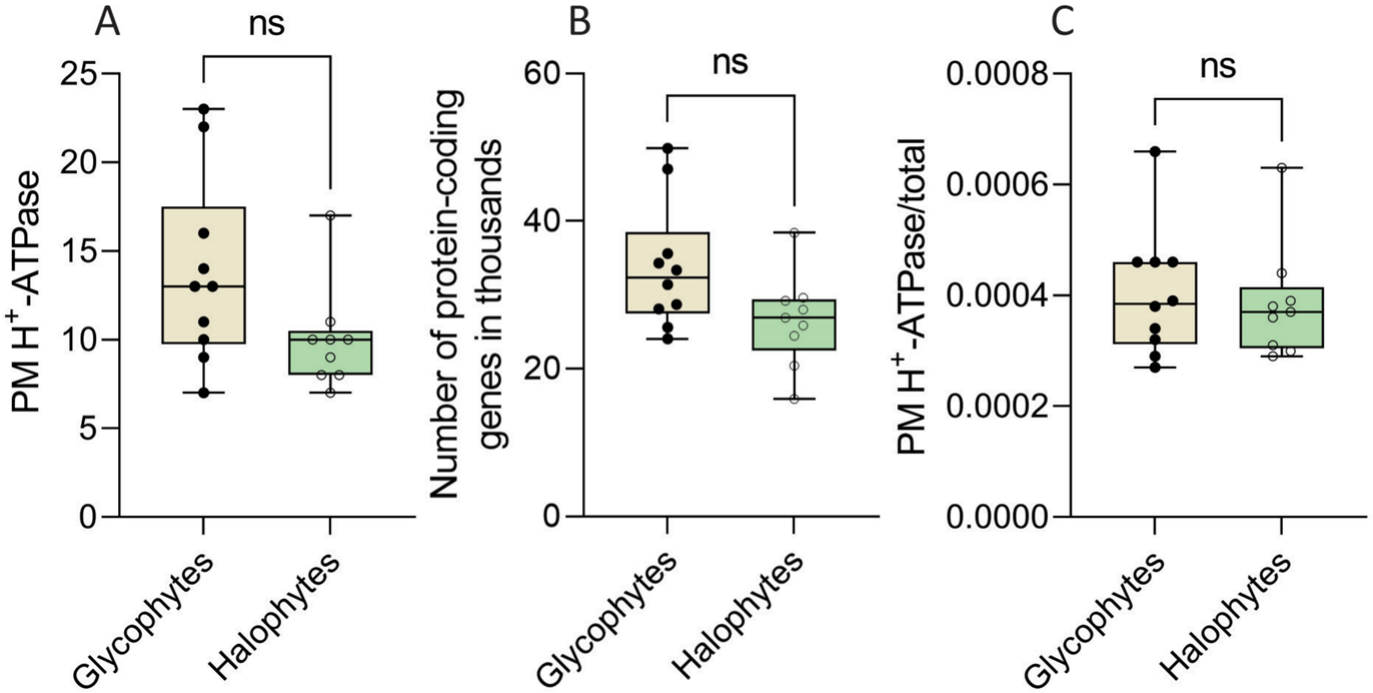
Comparison of the number of plasma membrane (PM) H^+^-ATPases (P3A ATPases) in halophytes and glycophytes. **A **The total numbers of protein-coding genes in the genomes of halophytes and glycophytes are listed in Table 3. **B **Comparison of the number of PM H^+^-ATPases in the listed halophytes and glycophytes. **C **Comparison of PM H^+^-ATPases in halophytes and glycophytes after normalization to the total number of protein-coding genes. Whiskers are minimum and maximum, the box is upper and lower quartile and median. ns, no significance

To determine if there is a difference in the number of P3A ATPases between halophytes and glycophytes, we only used diploid genomes for the analysis, as the ploidy could influence the results. Halophytic species were selected according to the The Food and Agriculture Organization of the United Nations (FAO) [41]. We first investigated if there was a general expansion in the number of genes between the two groups. No significant difference between the number of protein-coding genes in the two groups could be observed (Fig. 2A). Searching through the translated genomes with the consensus sequence for P3A ATPases earlier identified, the number of PM H^+^-ATPases was determined for the two groups (Table 3). On average, there were 10–12 P3A ATPase genes per plant genome (Fig. 2B), which could be normalized to about 0.00035% of protein-coding genes (Fig. 2C). The mean numbers were slightly lower in the halophyte group (10.3 ± 0.9 vs. 13.8 ± 1.6), but due to the sample size being limited by the number of species available for analysis it could not be ascertained whether the difference was significant.

**Table 3:** The number of genes and PM H^+^-ATPases and the ratio between PM H^+^-ATPases and the number of genes in glycophytes and halophytes. Additional references for numbers of protein-coding genes are given in Supplementary Table 6

Plant	Ploidy	Protein-coding genes	PM H^+^-ATPase	PM H^+^-ATPase/total	Scaffold N50	Citation for ploidy
**Glycophytes**						
*Allium cepa*	diploid	47066	22	0,00046	460,7 kb	[42]
*Eucalyptus grandis*	diploid	33352	11	0,00032	58,5 Mb	[43]
*Gossypium raimondii*	diploid	35609	14	0,00039	62,2 Mb	[44]
*Hordeum vulgare*	diploid	31448	9	0,00029	610,3 Mb	[45]
*Oryza sativa*	diploid	28738	10	0,00034	30 Mb	[46]
*Phaseolus vulgaris*	diploid	28134	13	0,00046	50,4 Mb	[47]
*Raphanus sativus*	diploid	49855	23	0,00046	19,9 kb	[48]
*Sesamum indicum*	diploid	24075	16	0,00066	2,1 Mb	[49]
*Solanum lycopersicum*	diploid	25613	7	0,00027	66,7 Mb	[50]
*Zea mays*	diploid	34337	13	0,00038	226,4 Mb	[51]
**Halophytes**						
*Aeluropus littoralis*	diploid	15916	10	0,00063	3,6 kb	[52]
*Beta vulgaris*	diploid	24491	7	0,00029	2 Mb	[53]
*Eutrema salsugineum*	diploid	26943	10	0,00037	13,4 Mb	[54]
*Limonium bicolor*	diploid	38444	17	0,00044	340,4 Mb	[55]
*Phoenix dactylifera*	diploid	29239	11	0,00038	4,7 Mb	[56]
*Setaria viridis*	diploid	28032	10	0,00036	11,2 Mb	[57]
*Solanum chilense*	diploid	25885	8	0,00031	70,7 kb	[58]
*Suaeda aralocaspica*	diploid	29604	9	0,00030	69,5 kb	[59]
*Zostera marina*	diploid	20450	8	0,00039	485,6 kb	[60]

## Discussion

### An expanded two-motif method for determining the number of genes in a family

**Fig. 3:**
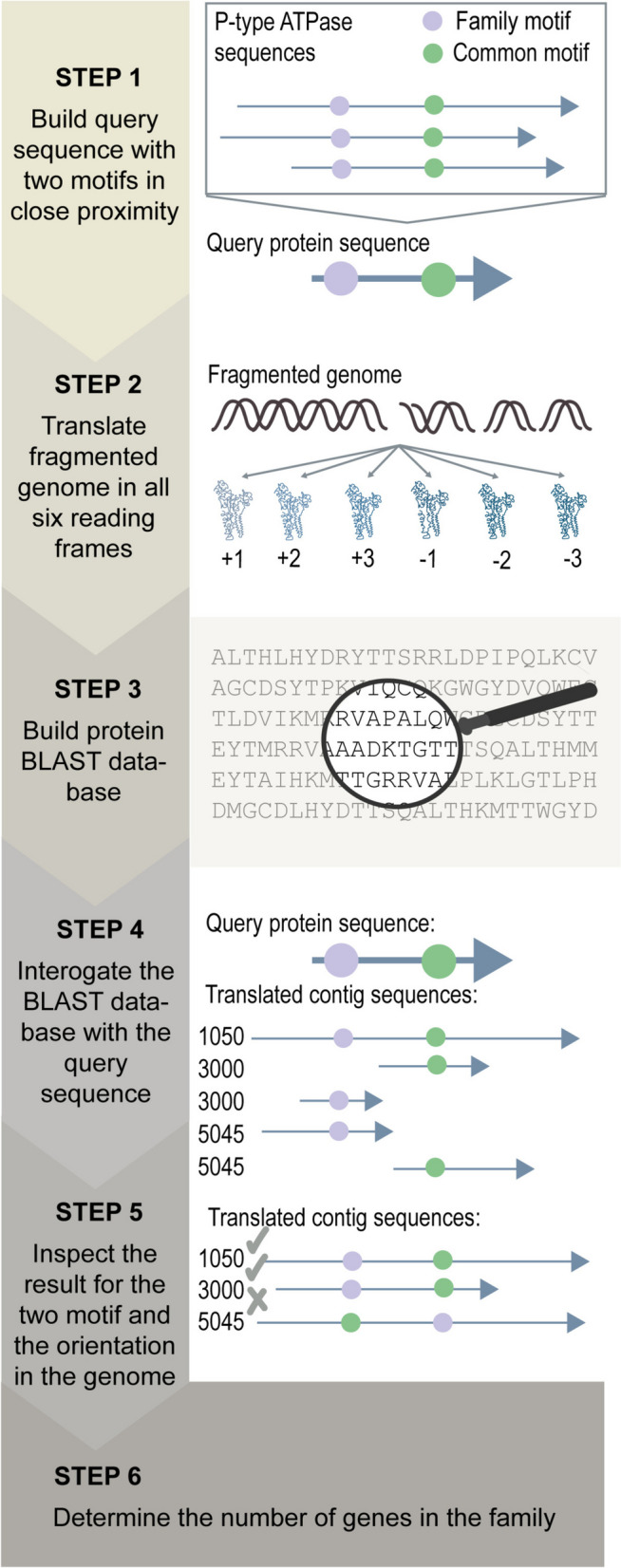
Flow chart of the procedure used to predict the number of genes for a family in a species of an incompletely assembled genome using the expanded two-motif method. An overview of the workflow for identifying all members of a specific family. Step 1: A query sequence is built by aligning sequences from the desired family. The query sequence should contain two motifs, with at least one being unique to the family. Step 2: The genome for the organism of interest is translated in all six reading frames. Step 3: A BLAST database is built using the translated genome. Step 4: The BLAST database is searched with the query sequence. Step 5: The BLAST result is inspected; the orientation and distance (if there are few or no gaps between the motifs in the family) of the two motifs is used to determine if a hit can constitute a member of the gene family. In our hypothetical example, inspection revealed hits on three hypothetical contigs: 1050, 3000, and 5045. For Contig 1050, the two motifs are close and correctly oriented. For 3000, the two motifs are located on each side of an intron; thus, the position in the genome is used to verify the orientation and establish if the hits are located relatively close to each other. 5045 is a negative hit. In this contig, there are two hits, but inspection revealed that the motifs are incorrectly oriented and therefore is deemed not to constitute a member of the family

We have described a simple and unbiased method for determining the number of genes within a gene family that circumvents the use of a transcriptome and/or predicted proteome (Fig. 3). The genome of interest is translated in all six reading frames to produce six protein sequences for each scaffold or chromosome, be it complete or fragmented. The protein sequences are used to build a BLAST database that can be searched with a short query sequence for the gene family in question. To apply the method, a gene family in question must contain two motifs in close proximity to each other. The query sequence can then be produced by aligning the protein sequences, and the consensus sequence adjacent to the two motifs can then be used to search the translated genome database. Once a sequence containing the motifs has been identified, it must be verified that (1) the motifs are located in the correct orientation relative to each other and (2) in close proximity to each other. If the hit fulfills these criteria, it can be considered to constitute a gene in the family. The method described can be used to identify genes of specific families in the P-type ATPase superfamily and most likely also in other gene families.

### Comparison with other methods

Tools are available for such analysis of novel genomes, as the Pfam database is based on sequence alignments and identification of common sequence motifs in gene family members. P-type ATPases form a large superfamily of primary active pumps divided into five major families. A unique motif for all members of the superfamily is the sequence DKTGT, but Pfam entries for the families remain to be identified.

An HMM has been used to identify soybean P2-type Ca^2+^ ATPase genes in the *G. max* genome [33]. Using the automatic annotation according to these criteria, 12 P2-type ATPases were identified, which is a number far below the 23 P2A/B ATPases we identified in this genome using our method based on the expanded two-motif method.

### Inventory of P-type ATPases in H. vulgare and identification of new family members

Using conventional methods, we identified all P-type ATPases presently annotated in the *H. vulgare* cultivar Morex V3 HC genome. *HvHMA7, HvACA3, HvACA10, HvAHA8*, and *HvALA6* were found to be incorrectly annotated when compared to the homologs from *A. thaliana* and *O. sativa* but could be corrected based on homology, intron–exon splice sites, and cloning.

We identified two novel P-type ATPases, namely, *HvHMA1-like* and *HvHMA2-like*. HvHMA2 and OsHMA2 are both involved in the root-to-shoot transport of zinc and belong to the same phylogenetic sub-clade of P1B ATPases. Among monocots, only *T. aestivum* has recently been reported to have two genes belonging to the same clade as *OsHMA2* [61]. *HvHMA2-like* was found to be a paralogue of *HvHMA2* and is located approximately 50 kb upstream of *HvHMA2.* Therefore, this gene could be the result of a gene duplication event by unequal crossover.

*HvHMA1-like* was found to have high homology to *HvHMA1* and *OsHMA1*, but *HvHMA1-like* has a premature stop codon after codon number 80, and neither *HvHMA1* nor *OsHMA1* has a premature stop codon. *HvHMA1-like* is expressed and can be cloned from cDNA from 10-day-old whole-seedling cDNA. This suggests that the N-terminal region of the protein is expressed, but its role, if any, for example, in regulating heavy metal transport, remains unknown.

### Evaluation of methods

We first surmised that the presence of two distinct motifs near each other with a fixed distance between them could be used to identify the genes in a specific family. As we tested this hypothesis, we found that even though the two motifs were relatively close together, a large part of the sought-after genes had an intron between the two motifs and could not be identified using this method. This shortcoming limits the effectiveness of the approach; however, though it can identify genes without an intron extremely well.

To address this shortcoming, we devised a method that expanded on the two-motif method to include more information between and surrounding the two motifs. The expanded two-motif method can successfully identify genes in which the two motifs are separated by an intron but fails to identify genes when the bait is distributed across three exons. This could potentially be negated by including more sequence information on either side of the motifs. However, if this is done, it might not be a feasible method for fragmented genomes where continuity is low.

### P3A ATPases in glycophytes and halophytes

We used the expanded two-motif approach to approximate the number of P3A ATPases in different species divided into two groups, glycophytes and halophytes. We aimed to determine if there is a genetic difference between the two groups in regards to P3A ATPases. We found no significant difference using the species selected here when the data were normalized to the number of protein-coding genes in each species. However, it may be too early to establish whether P3A ATPases have been reduced or expanded in halophytes as part of their salt tolerance mechanism. The number of species used for analysis was rather limited (as we opted to only use diploids), and the variability within each group made the difference not significant. Future studies involving a larger number of species in each group will provide an explicit answer about the possible causal link between salinity tolerance and the number of PM H^+^-ATPase copies.

### Advantages and limitations of the methods

The advantage of the expanded two-motif method described here is that it can be used on poorly annotated and poorly assembled genomes and on any gene family in which two unique sequence motifs occur in close proximity to each other.

When using the two-motif method when the distance between the two motifs is fixed, the rate of false positives will only be impacted by the probability of the two motifs randomly occurring with the specific distance between them. This was only observed once during the testing for HvP5. The larger the distance between the two motifs, the higher the possibility of them being split onto two scaffolds/contigs and vice versa or being separated by an intron. The distance should therefore be kept relatively short.

For the expanded two-motif method, if there is an intron between the motifs, it does not necessarily prevent identification but requires more work. The BLAST output will show two hits from the same scaffold/contig, and it will be necessary to investigate the position of the two hits, both regarding the orientation of the two compared to each other and the distance between them. Furthermore, as is evident from the testing, when the query sequence is distributed onto three exons, it is impossible to identify the family member using this approach. The method described will identify the location of the two motifs and thus will not identify the whole gene. Therefore, the method is prone to error because it cannot differentiate between true genes and pseudogenes. The expanded two-motif method proved useful for identifying gene members of the superfamily of P-type ATPases. Using the expanded two-motif method to screen well annotated genomes, we successfully identified all members of P3A and P5 ATPases even though the two motifs in some members of these families are separated by an intron.

### Supplementary Information

**Additional file 1: Supplementary file 1. ***Hordeumvulgare* P1B ATPase coding sequences.

**Additional file 2: Supplementary file 2. ***Hordeumvulgare* P2A ATPase coding sequences.

**Additional file 3: Supplementary file 3.** *Hordeum vulgare* P2B ATPase coding sequences.

**Additional file 4: Supplementary file 4.** *Hordeum vulgare* P3A ATPase coding sequences.

**Additional file 5: Supplementary file 5.** *Hordeum vulgare* P4 ATPase coding sequences.

**Additional file 6: Supplementary file 6. ***Hordeum vulgare* P5 ATPase coding sequences.

**Additional file 7: Supplementary file 7.** P-type ATPase protein sequences from *A*.* thaliana*, *O*. *sativa*, and *H*. *vulgare.*

**Additional file 8: Supplementary Table 1.** Genomes and assemblies used for the different species.

**Additional file 9: Supplementary Table 2.** Percent amino acid identity between P3A-ATPases (AHAs) from *A*. *thaliana*, *O*. *sativa* and *H*. *vulgare*.

**Additional file 10: Supplementary Table 3.** Number of introns in P-type ATPases from *A*. *thaliana*, *O*. *sativa* and *H*. *vulgare*. 

**Additional file 11: Supplementary Table 4.** Annotated and estimated number of plasma membrane H^+^-ATPases in annotated plant genomes.

**Additional file 12: Supplementary Table 5.** Overview of location, sequence hit, and chromosome location for Ca^2+^-ATPases in the G. max assembly GCA_022114995.1

**Additional file 13: Supplementary Table 6.** Number of protein-coding genes used for generation of Fig. 2.

**Additional file 14: Supplementary Figure 1.** Alignment of Heavy Metal ATPases (P1B ATPases) from A. thaliana, O. sativa, and H. vulgare. **Supplementary Figure 2.** Alignment of P2A ATPases from *A*. *thaliana*, *O*. *sativa*, and *H*. *vulgare*. **Supplementary Figure 3.** Alignment of P2B ATPases from *A*. *thaliana*, *O*. *sativa*, and *H*. *vulgare*. **Supplementary Figure 4.** Alignment of P3A ATPases from *A*. *thaliana*, *O*. *sativa*, and *H*. *vulgare*. **Supplementary Figure 5.** Alignment of P4 ATPases from *A*. *thaliana*, *O*. *sativa*, and *H*. *vulgare*. **Supplementary Figure 6.** Alignment of P5 ATPases from *A*. *thaliana*, *O*. *sativa*, and *H*. *vulgare*.

## Data Availability

Sequence data used in this article are available either at the KEGG webpage (https://www.genome.jp/kegg/), the National Library of Medicine (https://www.ncbi.nlm.nih.gov/), or the The Leibniz Institute of Plant Genetics and Crop Plant Research (https://galaxy-web.ipk-gatersleben.de/). Links to the genome assemblies used can be found in Supplementary Tables 1 and codes for organisms in KEGG can be found in Supplementary Table 4. P-type ATPase CDSs from *A. thaliana* and *O. sativa* can be found in refs. [17, 18]. P-type ATPase CDSs from *H. vulgare* can be found in Supplementary files 1, 2, 3, 4, 5 and 6, with all accession numbers given in Table 1.
