# Supplementary material for: A two-sequence motif-based method for the inventory of gene families in fragmented and poorly annotated genome sequences
Source: BMC Genomics. 2024 Jan 3;25:26. doi: 10.1186/s12864-023-09859-4 (PMC10763278; doi:10.1186/s12864-023-09859-4)
Supplement: Supplementary file 1 — Additional file 1: Supplementary file 1. Hordeumvulgare P1B ATPase coding sequences. [file 12864_2023_9859_MOESM1_ESM.pdf]

Supplementary File 1 – *Hordeum vulgare* P1B ATPase coding sequences

>HvHMA1
ATGCAGTTCCTCACCACCTCCGCCGCGTGCTCCTCCTCCGCGCCGCCGCTCCCGCGCCCC
GCGCACCTCCTCCGGGTCTCGAGGCCCCCTCCCTTCCCCCACCTCCGTCGCCGCCGCGCT
CCCCACCCACCCTCAGTATCTCTATCTCTAGCCCCCAAACCCTCGCTCCTCGCCGCCTCG
CGCCGCTCCCTGCTCTTCACCCCCCGCGCCCACGGCGACCACCACCACCACCACCACCAC
CACCACCACGGCCACGGGCACGGGCACGGCCACCATGGGCACGGCGACGACGGGGTGGAG
GTGCGCGGGGGAGGCGGGGGCGCGGCGGTCATGCGGATGGCGAGGACGATCGGGTGGGCC
GACGTCGCCGACGCGCTGCGGGAGCACCTGCAGCTCTGCTGCATCTCCCTCGGCCTTCTC
CTGACGGCCGCCGTCTGCCCGCACGTCCCGCTGCTGAACTCAGTCGGGCGCCTGCCGGCC
GCGCTCATCGCCATCGCCTTCCCTCTTGTCGGGGTTTCTGCAGCACTTGATGCCCTTGTA
GATATTGCAGATGGAAAAATAAATATCCATGTCCTCATGGCTCTTGCAGCATTTGCTTCT
ATATTTATGGGAAACTCATTGGAGGGTGGTTTACTTCTTGCAATGTTTAACTTAGCCCAT
ATCGCTGAAGAGTACTTTACAAGCAAGTCAATGTATGATGTGAGGGAACTTAAGGAAAAT
CATCCAGAATTTGCACTGTTGTTAGAAACAAGTGGAGACGAATCGGCACACTTTTCAAAT
CTAAATTATGCAAAAGTTCCTGTGCATGACCTTGAAGTGGGTTCTCATATTTTGGTCAGA
GCTGGTGAGGCTGTGCCTGTTGATGGAGAAGTTTACCAAGGATCGTCCACAATCACCATA
GAACACCTCACTGGTGAAACAAAACCTGTTGAGAGGACAGTGGGGGATGCTATACCAGGT
GGAGCCAGGAATTTGGAAGGAATGATGATTGTAAAGGTGACCAAATCATGGGAGGATTCA
ACACTCAACAGAATTGTCCAGCTGACAGAAGAGGGCCAGCTAAACAAGCCAAAGTTGCAA
AGATGGTTAGATGAGTTTGGGGAGCATTATAGCAAAGTTGTCGTGGCTCTGTCTTTGGCT
GTTGCACTACTGGGACCATTCCTCTTTAAGTGGCCCTTTTTCGGTAACTCAGTTTGTAGG
GGTTCAATTTACCGTGGGTTAGGACTTATGGTGGCTGCATCTCCATGTGCATTGGCAGTA
GCTCCATTAGCATATGCCACTGCAATCAGTTCTCTTGCAAGTAAGGGAATTTTATTGAAA
GGCGGGCATGTATTAGATGCCCTTTCTTCTTGTCAGTCTATTGCTTTTGACAAGACAGGC
ACATTAACAACTGGGAAGCTTATGTGCAAAGCAATCGAGCCCATTCATGGACATTTGGAT
GCGAGTAATGGTGTCGACCCCTCTTGCTGTACTCCCAACTGTGAAAGTGAAGCTCTAGCT
GTTGCTGCAGCTATGGAGAAAGGAACAACCCATCCTATTGGAAGGGCAGTTTTAAAACAC
TCTGTTGGGAGAGACCTCCCAGTGGTTGCTGTTGAGAGTTTTGAGAGCTTACCTGGTAGA
GGAGTTGTTGCTACTTTGAGTGGCATAAAGGCAAGAGACAATGAAAGTGAGTTTGCTAAG
GCATCTATCGGTTCCGTGGAATATATTTCTTCACTATACAGATCTTATGGTGAATCTGAA
CAAATAAAAGAGGCAGTAAAGTGTTCTGCATTTGGTCCTGAATTTGTTCAAGCCGCTCTC
TCAGTAGATAAAAAGGTAACCCTTTTCCACTTTGAGGATGAACCCCGTACCGGTGTATGT
GAAGTTATATATACCTTAAGAGAAAAGGCTAAACTTCGAATCATGATGCTTACTGGAGAT
CATGAATCAAGTGCTCAGAGAGTTGCTAAAGCTGTATGTATCGAAGAAGTTCACTTCTCT
TTAAAGCCAGAGGACAAGTTGAATAAAGTAAAAGCAGTTTCAAGGGAAGGAGGTGGAGGT
TTAATAATGGTTGGTGATGGTATAAATGATGCACCAGCTCTTGCAGCTGCAACAGTTGGT
ATTGTTCTAGCACAACGCGCCAGTGCAACAGCGGTAGCTGTTGCGGATGTTCTGTTGTTG
CAGGATAATTTATGTGTGGTGCCATTTTGTATCGCTAAAGCTCGCCAAACAACTTCATTG
GTGAAGCAAAGCGTAGCTCTTGCCTTAACCTGTATTGTTTTTGCTGCACTTCCTTCTGTC
TTAGGATTTCTTCCTCTTTGGTTGACAGTTCTTCTCCATGAAGGAGGGACCCTTCTCGTT
TGCTTGAACTCAATACGAGCTCTCAACCCTCCGACATGGTCTTGGGCAGATGACATCCGA
CAACTCGTCCATAGCCTAAAGAACTATGTCTCGGCAAAGTTGAATAGCTCCTCCTCAGAT
TGCTCAGCTAGCACCGTTCCTTTGTAG
>HvHMA2
ATGGCGGCACCGGCGCCGGCGGCGGCGGGAAAGCTGGAGAAGAGCTACTTCGACGTGCTG
GGCATCTGCTGCCCGTCGGAGGTGCCGCTGGTGGAGAAGCTCCTCGAGCCGCTCGCCGGC
GTGCACAAGGTCACCGTCGTCGTCCCCTCCCGTACCGTCATCGTCCTCCACGACGCCGCC

GCCATCTCCCAGGCCCAGATCGTGAGGGCGCTGAACGGGGCGAGGCTGGAGGCGTCGGTG
AGGGCGTACGGCGGCGCCGGGCAGAGCAAGGTGACCAACAAATGGCCGAGCCCCTACGTG
CTCGTCTGCGGGGTCCTGCTCGTCGTCTCGCTCTTCGAGCACTTCTGGCGGCCGCTCAAG
TGGTTCGCCGTGGCGGGGGCGGCCGCCGGGCTGCCTCCCATCATTCTCCGGAGCGTCGCC
GCGCTCCGGCGACGCACCATGGACGTCAACATACTCATGCTCATCGCAGTTGCTGGGGCC
ATAGCTCTCAAGGACTACTCCGAGGCTGGGTTCATCGTCTTCCTCTTCACCATAGCCGAA
TGGCTCGAAACCAGGGCGTGCGGCAAGGCCACTGCTGGGATGTCGTCACTAATGAGCATG
GCACCACAAAATGCTGTCTTAGCAGAGACTGGACAAGTAGTTGCTACTCAGGATGTGAAG
ATCAATACAGTAATAGCTGTCAAGGCAGGGGAAATCGTCCCGATCGACGGTGTTGTTGTC
GATGGTCGGAGTGAGGTCGACGAGAGCACCCTCACGGGAGAGTCCTTCCCGGTGTCCAAG
CAGGCAGACTCCCAGGTCTGGGCTGGCACACTCAACATAGATGGTTACATTGCTGTGAGG
ACAACTGCTATGGCTGACAACTCTGCGGTGGCCAAAATGGCAAGGCTGGTTGAAGAAGCC
CAAAACAACCGATCCAGTACGCAGAGGCTGATCGACACTTGCGCCAAGTACTACACACCT
GCTGTTATTTTCATGTCTGCAGCAGTGGCAGTGATCCCTGTGTGTCTCAAAGCACGCAAC
CTGAAACACTGGTTTGAACTGGCCCTAGTTCTCCTGGTGAGTGCCTGTCCATGTGCTCTG
GTGCTGTCGACACCCGTGGCAACCTTCTGCGCACTACTGAGGGCCGCGAGGACGGGGCTC
CTCATCAAAGGAGGGGATGTCCTTGAGTCCTTGGCCAGTATCAAAGTTGCTGCCTTTGAC
AAGACTGGTACAATTACTAGAGGGGAGTTCTCTGTGGAGGAGTTTCAGACAGTTGGTGAG
CGTGTTTCGAAGCAACAACTTCTATACTGGGTTTCAAGCATCGAGAGCAGGTCGAGCCAC
CCAATGGCAGCTGCTCTTGTTGGTTATGCTCAATCAAACTCCGTGGAGCCAAAATCAGAA
AATGTTGCTGAATTTCAAATGTATCCTGGTGAGGGGATTTACGGTGAAATTGGTGGAGAG
GGCGTATATGTTGGGAACAAAAGGATCTTGGCAAGGGCATCGTGTCAAATAGTTCCAGAC
ATAGTAGAACACATGAAAGGAGTTACCATCGGATACGTGGCCTGCAACAAGGAATTGATT
GGGGTATTCAGCCTCTCGGATTCATGCCGAACTGGATCAGCCGAGGCCATCAAGGAGTTG
AGATCACTGGGCATCAAGTCAGTGATGCTTACTGGCGATAGTACTGCTGCTGCCACACAT
GCACAGAACCAGCTGGGTAACATTCTAGCTGAGGTTCATGCTGAACTTCTACCAGAAGAC
AAAGTGAGAATTGTTGATGAACTGAAGGCAAGAGATGGCCCTACACTGATGATTGGCGAT
GGCATGAATGATGCCCCAGCACTGGCCAAGGCTGATGTTGGAGTTTCCATGGGCGTGTCC
GGTTCAGCCGTCGCAATGGAGACGAGTCACATTACTCTGATGTCGAATGACATCCGCAGG
ATCCCAAAGGCTATCAAGCTGGCCAGGAGGACTCACCGGACCATCGTTGTGAACATTGTC
TTCTCGGTGACCACGAAGCTTGCAATTGTTGCACTTGCATTTGCCGGTCATCCGCTTATT
TGGGCAGCAGTCCTTGCTGATGTTGGTACATGCTTGTTGGTGATCATGTACAGCATGCTG
CTACTGAGAGAGAAAGGCAGTGGAAAGGTGGCGAAGAAATGCTGTGCTTCTTCTCACTCA
AAGAAGCATGGGCACCGAACTACCCACCACTGCTCAGATGGTCATCACCATGAGAATGTA
TCAACAGGCGGTTGCGTGGATTCGTCTGCAGGTAAGCATTCTTGCCATGATCATCACCAT
GAGCATGACCACCACAAAGAGCCGAGCAACCTGCATTCCGTAGACAAGCATGGCTGCCAT
GATCATGGTCATGTTCATAGCCACTGCAAAGAGCCGAGCAGCCAGATGGTCACAAGCAAG
GATGTTGCCCATGGACATGGCCATACCCACAACATCTGCAACCCTCACCCTGCTGCAAAC
AAGCATGATTGCCATGACCACGAACATAGCCACCACCAAGAACCCAATAGTTCACATTCT
GCCGATGAGCATGATTGCCATGGTCACAAGCACTGTGAAGAACCAACCAGCTTGCTTTGT
GCCACTGAGCATGCTTGCCATGACCATGACCAGAACCATGAGCATCACTGCTGTGATGAA
GAGAAAACAGTCCATGTTGCAGATACGCATTCCTGCCACGACCATAAGCATGAGCAGGGT
GCAGCTGATTCAGTTCCAGAGCTATCGATATGGATCGAGGGTCAATCCCCTGATCACCGT
GAGCAGGAAATTCAATGCAGCACAGAACACAAAGAGGAAGCGTGTGGGCATCACCTGAAG
GTCAAGGATCAGGTCCCAGCTAAGACAGATTGCAGCAGGGGGGGCTGTCACGGTACCGCG
AGCAGCAAAACCTGCGAAAGCAAAGGTAAAAATGTTTGTTCAAGCTGGCCGGTTGGTCGC
ACCGGAGTTGTCCGCCGGTGTTGCAGGACTAGAACGCACAGCTGCTGCAGCCAAAGCATG
TTGAAACTACCTGAGATAATAGTAGGATGA
>HvHMA2B

ATGGCGGGCAAGTTGGAGAAGAGCTACTTCGACGTGCTGGGCATCTGCTGCCCGTCGGAG
GTGCCGCTGGTGGAGAAGCTCCTCGAGCCGCTCGCCGGCGTGCGCAAGGTCACCGTCGTC
GTGCCCTCCCGGACCGTCATCGTCCTCCACGACGCCGCCGCCATCTCCCAGGCCCAGATA
GTGAAGGCGCTGAACGGGGCGAGGCTGCAGGCGTCGGTGCGGGCGTACGGCGGCGGCGGC
CAGAGCAAGATCAACAAATGGCCGAGCCCATACGTGCTCCTCTGCGGGGTCCTCCTGGTC
GCCTCGCTCTTCCAGCACTTCTGGCGCCCGCTGAGGTGGCTGGCGCTCGTCGCCACGGCC
GCCGGCCTGCCGCCCATCGTCCTCAGGAGCGTCGCCGCCGCGCGGAGGCTCACCCTGGAC
GTCAACGTACTCATGCTCATCGCAGTTGCTGGGGCAGTTGCACTCAAGGACTACTCTGAG
GCCGGCTTCATCGTCTTCCTCTTCACCACCGCCGAGTGGCTAGAGACAAGGGCCAGCTGC
AAGGCCACCGCCGGGATGTCGTCGCTGATGAGCATGGCACCACAGAACGCCGTGCTAGCG
GAGACGGGGCAGGTGGTCGCGGCCCAGGACGTGAAGGTCAACACGGTGATAGCAGTGAAG
GCGGGGGAGGTCGTACCGATAGACGGCGTCGTCGTCGAGGGGCGGAGCGAGGTCGACGAG
CAGACCCTCACCGGAGAGTCCTTCCCGGTGGCCAAACAGACGGACTCGCAGGTCTGGGCC
GGCACCCTCAACATAGACGGGTACATTTCCGTGAGGACGACGGCCATGGCCGACAACTCG
GCGGTGGCCAAGATGGCGAGGCTGGTCGAAGAAGCCCAGAACAGTAGGTCCGAGACGCAG
AGGCTCATCGACACCTGCGCCAAGTACTACACGCCTGCGGTGATCGTGATGGCAGCGGCG
GTGGCGGTGACCCCGGTGATCGTGAGAGCGCACAACCTCAGGCACTGGTTTCAGCTGGCC
CTGGTGCTCCTGGTGAGCGCCTGCCCGTGCGCTCTAGTTCTGTCGACGCCGGTCGCCACC
TTCTGCGCGCTGCTCATGGCCGCGAGGACCGGGCTGCTCATCAAAGGAGGGGATGTCCTC
GAGTCCTTGGCCGGGATCAAAGTCGCGGCTTTCGACAAAACCGGCACGATCACCAGCGGA
GAGTTCTCCGTAGCCGAGTTCCGGCCAGTCGGAGAACGTGTTCCGAGGCAGCAGCTTCTC
AGCTGGGTTTCGAGCGTCGAGAGCAGGTCGAGCCACCCAATGGCAGCTGCCCTTGTTGAT
TATGCTCGATCGAACTCCGCGAAACCGACACCGGAGAATGTCATGGAATTTCAGATTTAT
CCTGGCGAGGGGATTTATGGTGAAATCGACGGACATGGCGTATATGTCGGGAACAGAAGG
ATCTTGTCAAGGGCTTCATGTGAAACAGTTCCAGAGGTAAATGACATCAAAGGGGTCACG
GTTGGATACGTGGCCTGCAACAAGGAACTGGTGGGGATATTCGGTCTCTCGGACGTCTGC
CGAACTGGATCCGCCGAAGCCATCAGGGAGCTCAGATCAATGGGCATCAAGTCGGTGATG
CTCACAGGCGATAGCGCTGCCGCTGCCACCCATGCACAGAACCAGCTGGGAAACGTCCTG
GCCGAGGTTCACTCCGACCTGCTGCCGGAGGACAAGGTGCGGCTCGTCGACGAGCTCAAG
GCGAGGGACGGCCCGACGCTGATGATCGGCGACGGCATGAACGACGCGCCGGCGCTGGCC
AGGGCCGACGTCGGGGTCTCCATGGGCGTGTCCGGATCGGCCGTCGCCATGGAGACCAGC
CACGTCACGCTCATGTCCAACGACATCCGCAGGATCCCCAAGGCCGTCCGGCTGGCGAGG
CGGACGCGCCGGACCATCGTCACCAACATCGTCTTCTCGGTCGCGACGAAGCTCGCCATC
GTCGGGCTGGCGCTCGCCGGCCACCCGCTCGTCTGGGCGGCGGTGCTGGCGGACGTCGGC
ACGTGCTTGCTGGTGATCATGTACAGCATGATGCTGCTGAGGGGGGGAGACGGTGCTCGC
CATGGGCCTCGCTCTCACATCGAGAAGCATGGCACGTCGGCGTCCCACCACCACCATTGC
TCCGACGGTCCGTGCAAATCAGCAGGCGGCTGCGGGGATTCGTCTGCTGGTGGCCATGCT
TGCGGCGATGAACATCATTGTCACGGGCATGGCGACCGCGAAGAGCCGGGCAGCCCGCAC
CACCACGGATGTGAAGATCATGGGCATGGCCACGGCCACTGCAAAGAACCGAGCAAGCTG
CACCCCATGGACAGCCATCGCTGTCAAGATCATGGCCATGGCCATGGCCACAGCCACTGC
AAAGAGGCGAGCATCAAGCTGGTCACAAGCAAGAACATTTCCCATGGCCATGGCCATAGC
CACTGCAAAGAGAAGCACGATGAGCACAGCACCAACTCGGTTGAGTCGTCAACCCAAGAG
CACTCCATCCTGATCGACGAATCAGCTGCTGCCCAACAACAAATCCTGTGCGATCACCAG
ATCGAAGAAGAATGCGGGCATCACTCGAAGGCCAAGGCCCGCGCCACGGCTCGTCCGACC
GACTGCGGCAGCCTCAGCCTCCGCCGCGACACGGTGGGCGACGACAACGAGGGATGCGGA
ACCAAAGCGAGGGACGCGTGCTCGAGCCGGCGGGCCGGCTGCGCTGCCGGGGAAACCGGG
CGGTGCTGCCGGAGCGCCAGGGCGAGCAGGTGCGGCGGCCACGCAAGCATGCTGAAGCTG
CCTGAGATCGTGGTGGAGTAG
>HvHMA3

ATGACGGGCAGCGGCGAGTCGTACCCGGCGCTCGAGGCGAGCCTTCTGTCCGACGAAGCG
GCGGCGTCGGCGAGGAGGAAGTGGGAGAAGACGTACCTGGACGTGCTGGGCGTGTGCTGC
TCGGCGGAGGTCGCGCTCGTCGAGCGTCTGCTGGCGCCGCTCGACGGCGTGAGGGCGGTG
TCCGTCGTCGTCCCCTCCCGCACCGTCGTGGTCGAGCACGACCCCTCCGCCGTCTCGCAG
TCCCGTATTGTGAAGGTCCTGAACGGGGCGGGCCTGGAAGCCTCGGTGCGAGCCTACGGC
AGCAGCGGGGTCATCGGCCGATGGCCCAGCCCGTACATCGTCGCCTGCGGCGCCCTCCTC
CTCGCATCCTCCTTCAGGTGGCTCCTGCCTCCCCTGCAGTGGCTGGCCCTGGGGGCGGCC
TGCGCCGGCGCTCCCCCGATGGTTCTCCGAGGGTTCGCCGCCGCCAGCAGGCTCGCGCTG
GACATCAACATTCTCATGCTTATCGCTGTTGTCGGTGCCGTCGCGCTCAAGGACTACACG
GAGGCAGGCGTCATCGTCTTCCTCTTCACCACTGCAGAGTGGCTCGAGACCCTGGCCTGC
ACCAAGGCCAGCGCCGGGATGTCGTCGTTGATGAGCATGATCCCGCCGAAGGCAGTCCTC
GCCGAGACGGGCGAGGTTGTCAATGTACGCGACATCGATGTCGGCGCCGTCATCGCGGTC
AGAGCAGGGGAGATGGTGCCGGTGGACGGCGTGGTTGTCGACGGGCAGAGTGAGGTCGAC
GAAAGGAGCCTCACCGGCGAGTCGTACCCGGTGCCCAAGCAACCGCTGTCCGAGGTCTGG
GCCGGCACGCTCAACTTGGACGGTTACATCGCCGTGAGGACAAGTGCCCTCGCCGAGAAC
TCCACGGTGGCCAAGATGGAGAGGCTGGTGGAAGAGGCGCAGCAGAGCAAGTCCAAGACG
CAGCGGCTGATCGATTCCTGCGCCAAGTACTACACGCCCGCCGTGGTGTTTCTCGGAGCA
GGGGTGGCACTGCTGCCGCCGCTGGTGGGGGCGCGCGACGCGGAGCGGTGGTTCAGGCTG
GCGCTGGTGCTGCTGGTGAGCGCGTGCCCGTGCGCGCTGGTGCTGTCGACGCCGGTCGCG
ACGTTCTGCGCGCTCCTGACGGCGGCGAGGATGGGGCTCCTCGTGAAGGGAGGGGACGTC
CTCGAGTCGCTGGGCGAGATCAAGGCCGTGGCGTTCGACAAGACCGGCACCATCACCAGA
GGGGAGTTCACCGTCGACATTTTCGACGTGGTCGGACACAAGGTTCAGATGAGCCAGCTT
CTTTACTGGATCTCAAGCATCGAGAGCAAATCCAGCCACCCAATGGCGGCTGCGCTGGTG
GAGTACGCGCAGTCGAAATCCATCGAGCCGAAACCCGAATGCGTCGCTGAGTTCCGCATC
CTTCCCGGCGAGGGCATCTATGGCGAGATCGACGGGAAGCGCATCTACGTCGGGAACAAG
AGGGTCTTGGCAAGGGCATCCTCCTGTCAGACAGTTCCAGAAAGAATGAATGGTCTGAAA
GGCGTCTCGATCGGCTACGTGATCTGCGACGGGGACCTCGTCGGGGTGTTCTCGCTCTCC
GACGACTGCCGGACCGGCGCGGCCGAAGCGATTCGAGAGCTGGCGTCCATGGGCATCAGC
TCAGTGCTGCTCACGGGGGACAGCGCGGAGGCGGCCGTGCACGCGCAGGAGCGGCTGGGA
GGAGCCTTGGAGGAGCTCCACTCCGAGCTCTTCCCGGAGGACAAGGTCCGGCTGGTGAGT
GCGGTGAAGGCGAGGGTTGGCCCGACAATGATGGTCGGCGACGGCATGAACGACGCCCCG
GCGCTGGCGATGGCGGACGTGGGCGTCTCCATGGGCATCTCCGGCTCGGCCGCGGCCATG
GAGACCAGCCATGCGACGCTCATGTCTAGCGACATCCTCAGGGTCCCCGAGGCCGTCAGG
CTCGGCAGGCGCGCCCGCCGGACTATCGCCGTAAACATGGTGTCCTCGGTGGCCGCCAAG
GTCGCCGTCCTCGCGCTCGCGCTCGCCTGGCGCCCGGTGCTGTGGGCAGCGGTGCTCGCC
GACGTGGGGACGTGCCTGCTCGTCGTGCTCAACAGCATGCTGCTGCTGGGGGAGGGGGGC
GGACGCCGCGGAAAGGAGGAGGCGTGCCGCGCCACGGCTAGGTCGCTGGAGATGAGAAGG
TCTCAACTCGCCGCCGTTTCACCGGACGCTGCCACTAAAAGCGTTGGAAAGACGGGCGGC
GACGCATCGAAAGGCTGCCATTGTTGCCACAAGCCTATCAAGTCCCCTGAGCACTCGGTT
GTCATCAACGTACGGGTAGACGAGCAACGTGAAGGGCCGACGGACGCGACATGTACGCCG
GCTAAAAATGTCGAGGTCACCGGACTTGTCGACGCCTCCGTAATGCCTGCTTCATCGAGC
TGCGTGTCGGGAGGAGGATGCTGCTCCCGTGAAAAAACAGGTAGGAACATGTAG
>HvHMA4
ATGGAGCGGAATGGACAGAGCCATCTCAAGGAGCCGCTTTTGCACGCGGGCGATGGCGCT
TCTCCTGCCGCCGCCAGGGTGTCCCCGCGGAAGGAGAGGACTACGAGGAAGGTCATGTTC
AATGTCCGGGGCATGTCGTGCGGTTCCTGTGCCGTCTCGATAGAGACAGTGGTGGCAGGA
TTGAAGGGGGTCGAGAGCATCCAGGTCTCAACCCTTCAGGGCCAGGCTGTTGTTCAGTAC
AGCCCCGAGGAGACCGATGCAAGAACCATAAAAGAAGCTATTGAGGATATCAACTTTGAG
GTTGATGAACTCCAAGAACAAGAAATTGCCGTATGCAGGCTCCGGATAAAAGGAATGGCA

TGTACAAGCTGTTCTGAATCTATTGAACGGGCACTTCTGATGGTACCTGGAGTTAAAAAA
GCTGCAGTGGGGCTTGCCCTAGAGGAAGCCAAGGTGCACTTTGATCCAAATATTACCAGC
CGTGATTTACTAATTGAGGCTATTGAGGATGCCGGATTTGGAGCCGATCTCATTAGTTAT
GGGGACGATGTGAACAAAATGCATCTAAAACTTGAGGGTGTGAGTTCTCCAGAAGACACC
AAACTCATTCAGTCGGCACTGGAAACTGTAGAGGGAGTGAATAATGTTGAATGGGACACA
TTGGGTCAAACAGTTACAGTTGCATATGACCCTGATGTCACTGGTCCAAGATTACTTATT
CAGCGCATTCAGGATGCTGCACAGCCCCCTAAATGCTTTAATGCCAGCTTGTACTCACCA
CCAAAGCAAAGAGAAGTAGAACGCCACCATGAAATTATGAGTTACAGGAACCAATTTCTT
TGGAGTTGCCTATTTTCAGTTCCTGTGTTCCTGTTCGCGATGGTTCTGCCCATGCTTCCT
CCTTCTGGAGATTGGCTGTTTTACAAAATCTACAACAACATGACGGTAGGTATGCTACTG
CGGTGGCTGCTATGTTCTCCAGTTCAGTTCATTATTGGTTGGAGATTTTACGTAGGAGCT
TACCATGCACTGAAGCGAGGGTACTCTAACATGGATGTGCTGGTTGCTTTGGGAACAAAT
GCTGCGTACTTCTACTCTGTGTACATTATTGTGAAGGCACTGACATCAGACTCGTTTGAA
GGACAAGATCTTTTTGAAACTAGTTCTATGTTGGTATCTTTTATATTGCTGGGAAAATAC
CTTGAGGTGGTGGCAAAGGGGAAGACATCAGATGCTTTGTCAAAGCTGACAGAACTTGCA
CCAGAAACAGCTGTACTTCTCACAATGGACAAGGATGGAGGTGTCATTTCAGAAGTGGAG
ATCAGCACCCAGCTACTTCAAAGAAACGATTTCATTAAGATTGTCCCTGGTGAAAAGGTC
CCAGTTGATGGTGTTGTCATCAAAGGCCAAAGCCATGTTAATGAAAGTATGATAACTGGG
GAAGCAAGGCCCATTGCAAAGAAACCAGGAGACAAGGTTATTGGTGGTACTGTAAACGAT
AATGGTTTCATAATTGTTAAGGCCACCCATGTTGGGTCAGAGACTGCTCTGTCACAGATA
GTCCAGCTAGTTGAAGCTGCTCAACTTGCAAGAGCTCCAGTACAGCGGTTGGCAGACAAG
ATTTCACGTTTTTTTGTTCCAACTGTTGTGGTGGCTGCATTTCTTACATGGCTTGGCTGG
TTCATACCCGGGCAACTTCACCTCTACCCTCAGCGATGGATTCCAAAGGCAATGGATAGT
TTTGAGCTTGCTCTGCAGTTTGGAATATCTGTCCTAGTCGTTGCATGCCCGTGTGCTCTG
GGATTAGCTACCCCAACTGCTGTTATGGTTGCAACTGGAAAAGGTGCTTCTCTAGGTGTT
CTTATCAAAGGTGGCAATGCACTTGAGAAAGCTCACAAGATTAAAACTATCATATTTGAT
AAAACTGGAACCCTGACTAAGGGTAAACCTTCTGTTGTTCAAACAAAGACCTTCTCCAAG
ATTCCACTTCTAGAATTGTGTGATTTAACTGCCAGTGCTGAGGCAAACAGCGAGCACCCT
CTATCAAAGGCTATTGTCGAATACACAAAGAAGCTCAGAGAACAATATGGATCTCCCAGT
GATCATATGATGGATTCCAAAGATTTTGAGGTGCATCCAGGGGCGGGGGTCAGCGCGAAT
GTTGAAGGCAAGCTGGTTTTGGTTGGGAACAAAAGGCTCATGCAAGAATTTGAAGCTCCA
ATGAGCTCTGAAGTGGAGGAATACATGTCTGAGATGGAGGATCTTGCCAGGACCTGTGTG
CTTGTTGCTATCGATAGGGTTATCTGCGGAGCTCTTGCCGTGTCGGATCCTCTGAAGCCC
GAGGCAGGGCGTGTCATTTCACACCTTAGCTCAATGGGCATCACAAGCATCATGGTGACG
GGCGACAACTGGGCTACAGCCAAATCCATAGCAAAGCAAGTCGGGATCAGCACCGTGTTT
GCTGAGATCGATCCAGTCGGAAAAGCCGAGAAGATCAAGGACTTGCAGACGCAAGGACTG
GCCGTGGCGATGGTCGGCGACGGGGTAAACGACTCGCCGGCCCTGGCCGCGGCGGACGTG
GGCATGGCCATCGGCGCAGGCACGGACGTCGCCATCGAGGCCGCCGACATCGTCCTGATG
AAGAGCAGCCTGGTGGACGTGATCACCGCCATCGACCTGTCGCGGAAGACCCTCGCCAAG
ATCCGGCTCAACTACGTCTGGGCCCTGGGCTACAACGTCCTGGGCATGCCGATCGCCGCC
GGCGTCCTGTTCCCGTTCACGGGCATCCGACTCCCCCCCTGGCTCGCCGGAGCCTGCATG
GCGGCCTCGTCGGTGAGCGTCGTCTGCTCGTCGTTGCTGCTCCAGCTCTACAAGAAGCCG
TTGCACGTCGAGGAGGCGCCGGTGCCGGCAGGACCCGGAGATGGCGGCTCGAATCTGGTG
TGA
>HvHMA5
ATGGCGGCGGGCACTCGGGGCGCCGTCTTCCTCGCCTGCTTCCGCGGCGGCGGCGCCGAG
GGGAGCGGCCACCGCCTCGCGCTGCGGCCGCGGTACCCGCCCATGCCGCGGCGCACCAAG
GCGGCCGCGGTCGCCGGCGATCTGGAGGCCGCGGCGGGGGCGGACGAGGAGGGGGAGGAG
GAGGAGGAGAAGGTGGCGGTGTTTGCAGTGACCGGCATGACCTGCGCCGCGTGCGCCGGG

TCGGTGGAGAAGGCCGTCAAGCGGCTCCCCGGCATCCACGACGCCGCCGTCGACGTCCTC
GGCTGCCGCGCGCAGGTCGCCTTCTACCCGGCATTCGTCTCGGAGGAAAAAATTAGGGAA
ACCATTGAAGATGTTGGTTTCGGAGCTAAACTGATTGACGAGGAGCTTAGGGAAAAGAGC
ATTCTAGTATGCAGGCTGCACATAAAAGGGATGACCTGCACGTCTTGTGCAAACACAGTT
GAATCCACGTTGCAAGCTGTTCCAGGGGTTCAGAGAGCTTCTGTTGCACTGGCTATTGAA
GAGGCGGAAATCCGTTATGATCGGAGGGTTGTTGCTGCTACGCAACTAGTCAATGCAGTT
GAAGAATCTGGATTTGAAGCAATACTGGTCACCGCAGGAGAAGATCGGAGCAGGATAGAC
CTCAAAGTGGATGGCATTCTCGATGAGACATCAGTAATGATAGTGAAAAGTTCTGTCCAA
GCTCTTCCTGGTGTGGAAGACATAAAAATCGATACCGAGCTCCAAAAGATAACCATCTCT
TACAAGCCTGACAAAACAGGTCCGCGAGACCTCATCGAAGTAATTGAGTCAGCTGGATCT
GGTCTTGTTGCTGTATCAATATATCCGGAAGCCGATGGAAGAGAGCAGCATAGAAATGGG
GAGATAAGGCGGTACAGGCAGTCCTTCTTGTGGAGCTTACTGTTCACAATTCCAGTGTTC
CTAACCTCCATGGTGTTCATGTACATCCCAGGGCTGAAGGATGGGCTGGACAAAAAGGTT
GTCAACATGATGAGCATTGGTGAACTATTGCGGTGGATTTTGTCAACACCTGTCCAGTTT
ATAATTGGCCGCAAGTTTTACACTGGTGCTTACAAGGCAATGTGTCATGGCTCACCGAAC
ATGGATGTGCTCATTGCTCTAGGGACCAACACAGCATACTTCTACTCAGTTTACTCGGTC
CTCCGAGCTGCCACTTCTGAGAATTATATGTCAATTGATTTTTTTGAGACAAGTTCCATG
CTCATATCATTTATCCTTCTTGGAAAATACCTCGAGATCTTGGCAAAAGGAAAGACCTCT
GAGGCTATTGCCAAGCTGATGGATCTTGCACCAGAAACTGCAACTGTGCTGATATATGAC
AAAGAAGGGAATGTTGTAAGCGAGAAAGAGATCGACAGTCGGTTGATTCAGAAAAATGAT
GTGATCAAAGTCATACCTGGTGGGAAAGTTGCTTCCGATGGATTTGTTATTTGGGGCCGG
AGCCATGTGAATGAAAGTATGATCACCGGAGAATCACGGCCTGTGGCAAAGAGGAAGGGC
GACACTGTGATTGGAGGGACGGTGAACGAGAATGGCGTGCTCCATGTCAGGGCAACATTT
GTTGGATCAGAGAGTGCCCTGGCACAGATTGTAAGGCTAGTAGAGTCAGCCCAAATGGCA
AAAGCTCCTGTGCAGAAGTTTGCTGATCAGATATCTAAAGTCTTTGTCCCCCTGGTCATC
TTTCTTTCTTTGCTTACTTGGCTTACATGGTTCTTAGCCGGGAGGTTTCATGGCTACCCT
AGCTCGTGGATACCATCTTCCATGGATAGCTTTCAGCTAGCTCTTCAGTTCGGGATATCA
GTTATGGTGATCGCCTGCCCTTGCGCCCTAGGACTTGCAACTCCAACTGCTGTGATGGTT
GCAACTGGAGTCGGTGCCTCGCAAGGTATTCTGATCAAGGGTGGGCAAGCTCTGGAGAGT
GCGCAGAAGGTGGACTGCATTATTTTCGACAAGACAGGAACACTAACCATTGGGAAGCCT
ATTGTCGTCAATACCAGGCTCTTTGAAAACATGGTCCTACGTGAATTCTATGACTATGTT
GCAGCAGCAGAGGTCAACAGTGAGCATCCGCTGGCGAAAGCGATAGTGGAGCATGCCAAG
AAATTCCACTCGGAAGAAACCCATATCTGGCCTGAAGCAAGGGATTTCATTTCAGTCACC
GGACACGGTGTCAAGGCAAAAATCGGCGACAAGAGTGTCATTGTGGGCAACAAGAGCTTT
ATGTTGTCATTGGACATTGATGTCCCCGTGGAAGCTTCAGAAATCCTCATGGAAGAGGAA
GAGAAGGCACACACAGGGATCATCGTGGCCATGGACCAAGAAATTGTGGGCATTATCTCT
GTGTCTGATCCGATAAAACCGAACGCTCATGAAGTGATATCATACCTCGAGTCAATGAAG
GTGGAGTGCATAATGGTGACCGGGGACAATTGGGGAACCGCCAATGCCATCGGCAAAGAG
GTCGGCATCCAAAACATCATCGCTGAGGCGAAGCCAGAGCAGAAGGCTGAGAAGGTGAAG
GAACTTCAGCTGTTAGGAAGAACCGTAGCGATGGTCGGCGATGGGATCAACGACTCCCCG
GCGCTGGTGGCAGCCAACGTGGGGATGGCCATCGGCGCGGGCACCGACGTGGCCATCGAG
GCGGCGGACATCGTGCTCATGAAGAGCAACCTGGAGGACGTGATCACCGCCATCGACCTG
TCCAGGAAGGCCTTCTTCCGCATACGGATGAACTACGTCTGGGCCCTCGGGTACAACATC
ATCGGCATCCCGATCGCCGCGGGCGTGCTGTTCCCGTCGACCCGGTTCCGGCTGCCGCCG
TGGGTCGCCGGCGCCGCCATGGCCGCGTCCTCCGTTAGCGTCGTCTGCTGGTCCCTGCTG
CTGAGGTACTACAAGAGGCCGTTGATCACACAGGAGCGACAGAAATAG
>HvHMA6
ATGGCCCACCTCCAGCTTACGGCGGTCGCCGGCGGCCGCGACGACGAGATGGAGGAGGTG
GCGCTGCTGGGGTCCTACGGCGAGCCGGAGGGCCTGAGCTCGCGGACTGGGCAGGAGGAG

GAGGAGGAGGAGGACGCGGGCATGCGCCGGGCCCAGGTGCGGGTCACGGGCATGACGTGC
TCCGCCTGCACGGGCGCCGTCGAGGCCGCCCTCTCCGCCCGCCGCGGCGTCCGGAGCGCC
GCCGTGTCGTTGCTGCAGAACCGCGCCCACGTCGTGTTCGACCCCGCGCTCGCAAAGGAA
GAAGACATCGTAGAAGCAATAGAAGATGCTGGATTTGAAGCAGAGATCCTCCCAGACTCT
GCTGTTTCTCAGCCAAAGTCACAGAAGGCTTTGTCAGGCCAATTTAGGATAGGGGGCATG
ACTTGTGCTGCCTGTGTGAACTCAGTTGAAGGGATCTTAAAGAAGTTGCCAGGTGTAAAC
AGAGCAGTTGTTGCATTAGCGACCTCATTAGGGGAAGTCGAGTACGATCCGGCTGCTATT
AGCAAAGATGAGATTGTTCAGGCTATCGAGGATGCTGGTTTTGAAGCTGCACTGTTACAA
AGTAGTGAGCAAGATAAGGCTTTATTAGGTTTGATAGGATTGCATACAGAGAGAGATGTA
AATTTATTATATGATATCCTGAGAAAAACAGAAGGCCTGCGCCAGTTTGATGTAAATTCT
GTAAGGGCAGAAGTTGAAATTACATTCGATCCAGAAGTCGTTGGTTTGAGATCGATTGTG
GATATTATTGAGATTGAAAGCAGTGGCAGACTGAAAGCCCATGTACAGAACCCATATGTA
CGGTCTTCTTCAAATGATGCGCAAGAGGCTTCAAAAATGCTTCACCTCCTTCGCTCCAGC
TTATTCCTAAGTATTCCAGTGTTTTTTATGCGCATGGTGTGCCCTCACATATCTTTCATT
AACTCATTCCTACTCATGCACTGCGGACCATTTCGTATAGGAGATCTGCTCAAGTGGATG
CTGGTGAGCGTAGTACAGTTTGTTGTTGGCAAACGTTTCTACGTCGCAGCTTATAGGGCC
CTTAGACATGGCTCTACAAATATGGATGTATTGGTTGTTCTTGGCACAACTGCGACATAT
GTATACTCAGTTTGCGCGCTTCTTTATGGTGCATTCACTGGATTTCATCCTCCAATGTAT
TTTGAGACAAGCGCAATGATAATTACATTTGTGCTATTGGGGAAGTATCTTGAGGTGCTT
GCTAAAGGACGGACATCAGATGCAATTAAGAAACTTGTAGAGCTGGTTCCTGCTACAGCT
ATCTTGCTTCTGAAATACAAAGATGGAAAATATGCTGGAGAGAAGGAAATTGATGCATTG
TTGATCCAACCTGGTGATGTCTTAAAAGTGCTTCCTGGCTCAAAGATTCCTGCTGATGGT
ATCGTCACTTGGGGTACAAGCCATGTTGATGAAAGTATGGTAACTGGTGAATCTGCGTCT
ATCTCCAAGGAAGTATCTAGTTCAGTAATTGGAGGCACCATGAACCTAAATGGCACCCTT
CACATACAAGCGGCGAAAGTAGGATCAGGGACAGTTTTGAGCCAGATAATATCTCTTGTT
GAGACTGCCCAGATGTCTAAAGCACCTATTCAGAAGTTCGCTGATTACGTGGCCGGCATT
TTTGTTCCTATTGTCATCACCTTGTCCTTACTGACATTCTGTACATGGTTTGTATGTGGG
ACGCTGGGGGCATATCCAAATTCATGGGTTTCAGAAACTAGCAATTGCTTTGTTTTCTCC
CTCATGTTCTCCATCTCTGTTGTGGTAATTGCCTGTCCATGTGCTCTTGGTCTGGCGACA
CCAACTGCTGTTATGGTAGCAACTGGAGTTGGTGCCAATCATGGAGTACTTGTAAAGGGT
GGAGATGCACTGGAGAGGGCTCAGAATGTTAAATATATTATATTTGATAAAACTGGGACA
CTGACACAAGGAAAGGCCACTGTAACAACGACGAAAGTGTTCTCAGGAATGGATGTTGGC
GACTTCCTCACATTGGTAGCATCTGCGGAGGCAAGCAGTGAGCATCCACTTGCAAAAGCT
ATCTTGGATTATGCATTTCATTTCCATTTCTTTGGCAAACTCCCCTCATCAAAAGATGAT
GTTAAGAAAAGAAAAGAAGACGCATTCTCTCAATGGCTCTTGGAAGTTGCTGATTTTTCT
GCCTTGCCTGGCAAAGGAGTTCAATGTTTGATCAATGGGAAGATGATTCTGGTGGGCAAC
CGTGCTTTGATATCTGAAAATGGGGTTAACATTCCTGAAGAGGCTGAAAGTTTCTTGGTA
GACATGGAATTGAATGCAAAGACAGGCATTCTTGTAGCATATGATGGCGACTTCATTGGA
TTAATGGGGGTAACTGATCCCTTGAAAAGGGAGGCTGCTGTAGTTATACAAGGCCTAAAA
AAGATGGGCATTTATCCAGTTATGGTGACAGGGGACAATTGGAGGACTGCACTGGCGGTT
GCAAAGGAGATTGGAATTGAAGATGTGAGAGCAGAGGTCATGCCTGCTGGAAAAGCCGAC
GTAATCCGCTCTTTGCAGAAGGATGGGAGTGTGGTTGCAATGGTTGGAGATGGGATCAAT
GATTCCCCTGCCCTAGCAGCAGCCGATGTTGGGATGGCCATCGGTGCAGGGACTGACATT
GCCATCGAGGCAGCAGATTATGTGCTGGTGCGGAATAACCTTGAAGACGTCATCACGGCG
ATTGATCTCTCAAGGAAGACATTCAGCCGAATCCGGTGGAACTACTTCTTTGCCATGGCT
TATAACATCGTTGCCATCCCCGTGGCTGCCGGCGCACTCTTCCCCCTCATCGGCCTGCAG
ATGCCGCCATGGCTGGCTGGTGCTTGCATGGCCTTCTCGTCTGTTAGTGTAGTATGTTCC
TCGCTGCTACTGAGGAGATATAGAAAACCAAGGCTTACCACCGTCTTACAGATAACTGTA
GAGTGA

>HvHMA7
ATGGACCCGGCCACGCCCCTCCTCGCCATCTCCAGGGCTATCTCCTCCCGCTCCAGGACC
TTCCCCGCCTCGTCCCCACACAACATCCTCCTCCGCGGCCGCCCCCCCACGGCCCCGCTC
GGTCGCGCCCCTGTCTTCGCCGCTTCCGCGCAGCGGCGCTTCGCCGTGAGCGGCGACCTC
CTCTTCCTCTCGCTCGCGCGCCTAGCTCTCCGCGGCCCGGCCTCCCCGCGCGCGGGGCCT
CGCCGCTGGTTCGCGAGCGTCTCGGCTTCCTCCCTTGCGTCGGCCGGTCCTCCCGGCGGC
GGGTGCGGTAGGGGGAACGGAGATGGCGGGGGCGGCGGTGATGGAGGCGGCGATGGGTGG
AAGCGGCCTCGTGCTTCCCAGGGGACGGCGGTGGCAGAGGAAGCGGCAGGGCTGGAAGCC
GATACCATAATCCTTGACGTTGGGGGGATGTCTTGTGGGGGATGTGCAGCAAGCGTTAAA
CGCATTTTGGAGAATGAGCCCCAGGTGGTGTCCGCAACTGTCAATCTTGCCACCGAGATG
GCAGTTGTGTGGGCTGTGCCAGAAGATAGAGCTGTACAAGATTGGAAACTGCAGTTGGGT
GAGAAGCTCGCTAGTCAGTTGACAACATGTGGGTACAAATCCAGCCAGCGAGATTCTTCA
AAAGTCAGTTCACAGAATGTTTTCGAAAGAAAGATGGGCGAAAAACTGCAAAATCTGAAG
CAAAGTGGTCGAGAACTTGCTGTATCTTGGGCACTATGTGCTGTTTGCCTACTGGGACAT
ATTTCTCATCTCTTTGGAGTTAATGCACCATTGATGCACCTGTTTCATTCCACTGGATTC
CATTTGTCTCTCTCAATATTTACATTTATTGGGCCTGTCAGCTCAGTTGCAGCCTTCATT
CCAAAACTGGGATGGAAGACATTTTTTGAGGAACCAATTATGTTGATAGCTTTTGTTCTT
CTAGGGAAGAATCTTGAGCAGAGGGCGAAGCTAAAAGCTGCTAGTGATATGACCGGATTA
CTCAATATACTTCCATCAAAAGCACGCCTAATGGACCGCATTCCAGCCGATGGACTTGTG
AAAGCTGGAAGAAGTACAGTTGACGAGTCAAGTTTGACAGGTGAACCTATGCCGGTAACT
AAGATTGCAGGGGCAGAAGTATCAGCGGGGAGCATTAATTTAAACGGTAAACTGACAGTT
GAAGTTCGACGACCTGGCGGTGAGACTGTCATGTCTGACATACTTCACTTAGTGGAAGAA
GCACAGACAAGGGAAGCCCCTGTTCAACGATTAGCTGACAAGGTTGCTGGGAACTTTACA
TATGGTGTTATGGCGCTTTCTTCTGCTACCTTTATGTTCTGGAGTATTTTTGGTTCACAA
CTTGTACCTGCTGCTATCCAGCAGGGAAGTGCAATGTCTCTGGCTTTGCAGCTTTCTTGC
AGTGTTCTGGTAATTGCTTGCCCATGTGCTCTTGGTCTTGCCACACCCACTGCAGTGCTG
GTTGGTACTTCGTTAGGTGCAACGAGAGGACTTCTTTTACGTGGTGGGGATGTTTTGGAG
AAATTCGCGGAAGTTGATGCCATTGTGTTTGACAAGACCGGAACTTTAACAATTGGGAAG
CCTGTAGTGACAAAAGTAATAGCTTCTCACAGCGAGGGAGGTGTAAATACAAAAGATTAC
AGGAACAATGAATGGACAGAAGGTGACGTTCTTAGTTTGGCTGCCGGAGTAGAATCAAAT
ACAAACCACCCACTTGGAAAAGCCATCATGGAAGCTGCCCAGGCTGCCAACTGCATCAAT
ATGAAGGCAAAGGATGGGTCCTTTATGGAAGAACCAGGGTCTGGTGCTGTGGCTACGATT
GGTGAAAAACAGGTTTCGGTTGGGACATTAGACTGGATTAGGAGGCATGGTGTTGTTCGT
GAACCATTTCCTGAAGCAGAAAATTTTGGTCAGTCTGTTGCATATGTAGCAGTTGACGGT
ACTCTAGCTGGTCTTATTTGTTTCGAGGATAAGATCAGAGAAGATTCTCATCAAGTTATC
AATGCCCTGTCTAAGCAAGGAATTAGTGTGTATATGTTATCTGGGGACAAGGAGAGTGCT
GCTATGAATGTTGCCTCAATTGTTGGCATTCAGTTAGACAAGGTGATTTCTGAAGTTAAA
CCACACGAGAAAAAGAAGTTCATATCTGAACTTCAAAAGGAGCACAAATTAGTTGCCATG
GTTGGTGATGGCATTAATGATGCTGCAGCCCTAGCTTTAGCTGACGTTGGAATTGCAATG
GGTGGAGGTGTTGGTGCAGCTAGTGACGTATCTTCAGTTGTTCTCATGGGTAATAGGTTA
TCTCAGCTTGTTGATGCTTTAGAGTTAAGTAAAGAAACCATGAGAACAGTGAAGCAAAAT
CTTTGGTGGGCTTTTCTGTATAACATTGTTGGGCTACCCGTTGCTGCTGGAGCATTGCTG
CCAGTGACGGGTACGATGCTGACCCCGTCGATAGCTGGAGCACTCATGGGTTTTAGTTCA
GTCAGCGTGATGGCCAATTCCTTGCTTTTGAGGGCGAGGATGAGTTCAAAGCATCATGTT
CAGAGCAGACAAAAGCCTCACAACACTATTTCTGATGTGTCAGACGGGGCTGGTGAGGTA
GAGCAAAGTTATCCATCAAAATGGAGGAGTACCTGA
>HvHMA8
ATGGCCACCGCTCCCGCCTCCTCCCTCCTCCGCGCGCGCGCCCAAATTCCCAGCGCCAAT
CCCCTTCTCTTCGCGCGACGCCTCCGCCTGCCGCGCAACCGCCGCCCGGCTCCCGCCTCC

CAGCAACGCCTCCGCCTCCGCCTCCGCCTCATCCCGCGCGAGCTATCAGCGGGCGCGCCC
CGCGCCACGGCCGACCCCTCCGCCTCGACCGCCGCCGTCGACGCGCCCCCCGTGGCGGAG
GAGGGCGCCAGCGCCACCGTGCTGCTCGACGTCAGCGGCATGATGTGCGGCGGCTGCGCG
GCGCGGGTGCGGTCCATCCTCGCCGCGGACGCGCGGGTGGAGAACGCGGCGGTCAACCTC
CTCGCCGAGTCGGCCGCGGTGCGGCTGCGGTCCCCGGCTCCGGGCGCCGGGGAGGAGCTT
GCGGAGAGGCTGACCGTGTGCGGGTTCCCCTCCGCGGCGCGGCGAGGGGGCGCGACCGCG
GGGGCCGCGGAGAGCGCGCTCAAGTGGAAGGAGATGGCCGCCCGGAAGTCGGAGCTCCTC
ACCCGGAGCCGTGGGCGCGTCGCCTTCGCGTGGACGCTCGTGGCGCTCTGCTGCGGCTCG
CACGCGTCCCATCTCCTCCACTCGCTCGGCATCCACATCGGCCATGGAACATTTTTTGAT
GTGTTGCATAACTCGTATGTGAAATGTGGCCTTGCTGTGGTGGCGCTGTTTGGGCCTGGA
AGAGATATACTTTTCGATGGTCTAAGAGCGTTCAAGCAAGGCTCCCCCAACATGAATTCT
CTTGTAGGATTCGGGTCTGCAGCTGCATTTGCTATTAGTGCAGTTTCATTGTTGAACCCT
GAATTGGCATGGAATTCAACCTTCTTCGATGAACCGGTCATGCTTCTTGGATTTGTACTT
CTGGGACGATCTCTTGAGGAAAGTGCTAGGCTTAAGGCATCTAGCGATATGAATGAACTC
ATTTCACTCTTATCTCCTCAGTCGAGGCTAATCGTTACATCCTCAAGTGACGACCTTCCT
TCAGATGGCATTTTGAATTCGGATGCAATAACAGTTGAAGTTCCTGTCGATGATGTCCGT
GTTGGAGACTCGGTATTGGTTCTTCCAGGAGAAACTATTCCTGTAGATGGAAATGTCACT
GGAGGATCAAGTTTTGTGGATGAATCAATGCTTACTGGAGAATCCTTGCCGGTAGCAAAA
GAAAAGGGATGCCCTGTATTTTCTGGAACTGTTAATTGGGATGGACCTTTAAGGATCAAA
GCCACAACGACTGGACCGTCATCAACAATTGCTAAGATAATCCGCATGGTTGAGGATGCA
CAAGCACATGAAGCTCCTGTTCAAAGGCTTGCTGATGCAATTGCAGGGCCGTTTGTGTAT
ACTGTTATGACGCTGTCTGCAGCAACCTTTTCTTTCTGGTACTTATTGGGCACACACCTA
TTTCCGGAGGTCCTTCTAAATGATATCTCTGGTCCTGATGGGGATTCATTGCTTTTGAGC
TTGAAGCTTGCTGTGGATGTACTAGTTGTTTCCTGTCCATGTGCACTTGGATTAGCTACG
CCTACAGCTATCTTAATAGGAACTTCCATGGGTGCTAAAAGAGGGCTACTTATTAGAGGA
GGTGATGTTTTGGAGCGTTTGGCGGGGATAGATGCAATTGTTCTGGATAAGACAGGGACG
CTTACGAAAGGAAAACCAGTTGTTACTTCTATTGCTTCTTTAGCATATGACGAGGTGGAC
ATTCTTCGCCTTGCTGCTGCAGTGGAGAAAACAGCATTGCATCCTATTGCAAATGCTATA
ATGAAGGAGGCTGAACTTTGCAAACTAGATATTCCAACGACAAGTGGGCAGCTTACACAG
CCTGGTTTTGGCTGTTTGGCTGAAGTAGATGGGCGTTTGGTTGCAGTGGGTAATTTGGAC
TGGGTGCACAATCGTTTTGAAACCAAGGCGTCGCCAACTGAACTGAGTGATCTTGGAAAG
CGCCTAGAATTTGTGCCTTCTAGTGAAGCATCATCTTCAAATCAGTCAAAATCAATCGCT
TATATTGGTCGTGAAGGAGAAGGAATAATAGGTGCTATTGCTATCTCAGATGTTCTGCGT
GATGATGCAAAATCAACTGTGGACAGGCTACAGCAGGAAGGAATTGCAACCTATATACTG
TCAGGAGACAGGAAAGAAGCGGTGGAAGGCATTGGGGAAGCTGTTGGAATCAGGAGTGAA
AACAGAAGGTCGTCCCTCACCCCACAGGAAAAGGCAGGCATTATATCAACTTTGCAAGGG
GAGGGGCATAGAGTTGCCATGGTTGGCGATGGAATAAACGACGCACCATCCTTGGCAGCT
GCTGATGTTGGAATAGCAATGCGGACTCACTCGAAAGAGAATGCCGCCTCTGATGCAGCT
TCAGTAGTTCTACTAGGGAACAGACTTTCTCAGGTTGTAGATGCCCTATCCCTCTCTAAA
GCAACTATGGCGAAAGTTCACCAGAACTTGGCCTGGGCGGTGGCGTATAACATAGTTGCC
ATCCCCGTCGCGGCGGGAGCGCTGCTGCCTCAGTTTGATTTCGCCATGACACCATCTCTT
TCAGGAGGACTGATGGCCCTGAGCTCCATCTTTGTCGTCAGCAATTCTTTACTCCTGCAG
CTGCATGGATCGTTTCAGAAGACGGAGAGACCAGGGCCCGATGATCTGAAGTCTAGACCA
AAGTCCCAGATGTAG
>HvHMA9
ATGGCCCACCTCCAGCTCGCGGCGGTCGCCGGGGGCGGCCGCGCGGGCGACGACATGGAG
GACGTGGCGCTGCTGGGATCCTACGACGAGGAGACGGGCGGAGCGGCGCCGGCGGGAGGC
GGCGGCGGCGCGGAGGACGAGGAGGAGGCGGAGGCGCACGTGCGGGTGACGGGCATGACG
TGCTCCGCCTGCACCAGCGCCGTCGAGGCCGCCGTCTCCGCCCGCCGCGGCGTCCGGCGC

GTCGCCGTGTCCCTGCTCCAGAACCGCGCCCGCGTCGTCTTCGACCCCGCGCTCGCCAAG
GTCGAGGATATAATAGAAGCTATAGAAGATGCTGGATTTGACGCTGAGATTCTCCCAGAT
TCTGCAGTCCCTCAGTCTAAGTCACAGAAGACATTGTCAGCACAATTTAGGATAGGAGGA
ATGACATGTGCTAATTGTGTAAACTCGGTTGAGGGTATCTTAAAGAAACAACCCGGTATA
AAAGGGGCAGTCGTTGCCCTGGCAACCTCATTAGGAGAAGTTGAGTATGATCCGTCTACC
ATTAGCAAGGATGAAATCGTCCAGGCCATCGAGGATGCTGGTTTTGATGCTGCATTCTTA
CAGAGTAGCGAGCAAGATAAGGTATTGCTAGGCCTGACTGGCGTCCACGCAGAGAGGGAT
GCAGATATACTACACGATATTCTCAAGAAAATGGATGGTTTGCGACAGTTTGGTGTAAAT
ACTGCCCTCTCAGAAGTTGAGATTGTGTTTGATCCAGAGGCTGTTGGTCTGCGTTCAATT
GTGGATGCTATCGAAATGGGAAGCAATGGGAGATTCAAAGCACACGTGCAGAATCCTTAC
AGTCGAGGGGCTTCGAATGACGCACATGAGGCCTCCAAAATGCTCCATCTTCTTCGTTCT
AGCTTATTCCTAAGTATTCCTGTGTTTTTTATACGTATGATCTGCCCTAGTATACCTTTC
ATCAGTACATTGCTTCTCATGCACTGTGGACCATTTCATATGGGGGATTTGGTGAACTGG
ATATTGGTTAGCATTGTACAGTTCGTTATTGGCAAGAGATTCTATGTTGCAGCTTACAGA
GCCTTAAGACATGGTTCTACAAATATGGATGTGTTGGTTGTTCTTGGCACCACTGCATCG
TATGTATATTCTGTTTGTGCACTACTATATGGAGCATTCACTGGATTTCAACCTCCCATA
TATTTCGAGACAAGTGCAATGATAATTACGTTTGTGTTATTTGGGAAGTATCTTGAGGTT
CTTGCAAAGGGAAAGACATCAGATGCTATTAAAAAGCTTGTAGAACTTGTCCCTGCTACT
GCTGTTTTGCTTCTGAAAGATGAAGAAGGAAAATATGTTGGGGAGAGGGAGATTGATGCT
CTGTTAGTCCAACCTGGTGATGTCTTGAAAGTGCTTCCTGGTTCGAAAGTTCCTTCTGAT
GGTTTTGTTGTTTGGGGAACAAGCCATATCAATGAAAGTATGATAACTGGTGAATCTGCC
CCTATGCCCAAAGAAGTATCGAGTGTAGTAATTGGAGGGACAATCAACTTACATGGCATC
CTTCATATACAAGCAACTAAAGTAGGATCCGGAACAGTTTTGAGTCAGATAATATCTCTG
GTTGAAACTGCTCAAATGTCCAAAGCCCCTATTCAGAAATTTGCCGATTATGTGGCTAGC
ATTTTTGTTCCTATTGTCATCACCTTGTCCATTCTAACGTTCTCCGTGTGGTTCTTATGC
GGATCGTTTGGAGCATATCCACATTCATGGTTTGACAGAACAAGCAATTGCTTCGTCTTC
TCCCTCATGTTTTCCATATCTGTTGTGGTGATTGCTTGTCCTTGTGCCCTTGGTCTGGCA
ACACCAACTGCTGTAATGGTGGCAACTGGAATCGGGGCTAATCACGGAGTACTTGTAAAA
GGTGGAGATGCATTGGAAAGGGCTCAAAATGTGAATTACGTGATCTTTGATAAAACAGGG
ACACTAACACAAGGAAAGGCTGTTGTAACAACTGCAAAGGTTTTCTCTGGAATGGACCTT
GGAGATTTCCTCACACTAGTAGCATCTGCAGAGGCAAGCAGTGAGCATCCTCTTGCAAAG
GCTGTATTGGAATATGCATTTCATTTCCATTTCTTTGGCAAGCTCCCTTCCTCAAAGGAT
GGCCTTGAGCAAAGAAAAGAGCAGATATTGTCGCAATGGTTGCTTGAAGCTGAAGATTTT
TCTGCTGTGCCTGGCAAAGGAGTTCAATGTTTGATCAACGAGAAGAAAGTTTTGATTGGA
AACCGTGCCTTGATGAATGAGAACGGGGTGAGCGTTCCCCCGGAAGCTGAAAGTTTCTTG
GTAGACCTGGAACTGAATGCAAAAACAGGCATTCTGGTGGCATATGACAGCAGTTTCATG
GGGTTGATGGGGATAGCCGATCCCCTGAAAAGAGAGGCTGCCGTAGTCGTGGAAGGACTG
AAGAAGATGGGCATTCATCCAGTGATGCTCACAGGTGACAACTGGAGGACTGCACAAGCT
GTCGCGAAAGAGGTTGGCATTGAGGATGTGAGAGCAGAGGTCATGCCTGCCGGGAAAGCC
GACGTGGTCCGGTCGCTCCAGAAGGACGGGAGCATAGTTGCCATGGTAGGGGACGGCATC
AACGACTCCCCAGCCCTCGCCGCAGCCGACGTCGGGATGGCCATCGGGGGCGGCACGGAC
ATCGCCATCGAGGCCGCGGACTACGTGCTGGTGAGGAACAACCTGGAGGACGTGATCACG
GCGATCGATCTCTCGAGGAAGACCTTCAACCGGATCCGCTGGAACTACTTCTTCGCCATG
GCGTACAACGTAGTCGCCATACCGGTGGCGGCAGGCGCGCTGTTCCCGATGACGGGGCTC
CAGATGCCGCCGTGGCTGGCCGGCGCCTGCATGGCCTTCTCGTCGGTGAGCGTGGTGTGC
TCTTCGCTCCTGTTGAGAAGATACAGAAAACCCAGGCTCACCACCGTGCTGCAGATTACT
ATAGAGTAG
