# Supplementary material for: A two-sequence motif-based method for the inventory of gene families in fragmented and poorly annotated genome sequences
Source: BMC Genomics. 2024 Jan 3;25:26. doi: 10.1186/s12864-023-09859-4 (PMC10763278; doi:10.1186/s12864-023-09859-4)
Supplement: Supplementary file 2 — Additional file 2: Supplementary file 2. Hordeumvulgare P2A ATPase coding sequences. [file 12864_2023_9859_MOESM2_ESM.pdf]

Supplementary File 2 – *Hordeum vulgare* P2A ATPase coding sequences

>HvECA1
ATGGGCAAGGGCGGGCAGGACGAGGCCGTTCGGCCCGATGGATCCGGTTCCCCCGGGCCC
GACCCCGACGTGCCCGTCTTCCCGTTCTGGGCGCGCACCCCGAGCGAGTGCCTGGCGGAG
CTCGGCGTTTCGGCTGACCGCGGCCTCAGCTCCGACGACGCGGCGGCGCGGCTGCACAAG
TACGGGCCCAACGAGCTGGAGCGCCACGCGCCGCCGTCCGTGTGGAAGCTGGTGCTCGAG
CAGTTCAACGACACGCTCGTGCGCATCCTCCTCGCCGCGGCCGTGGTCTCCTTCGTGCTC
GCGCTCTACGACGGCGCCGAGGGCGGCGAGGTCAGGGCCACCGCCTTCGTCGAGCCGCTC
GTCATCTTCCTCATCCTCATCGTCAACGCCGTCGTCGGGGTCTGGCAGGAGAGCAACGCC
GAGAAGGCGCTCGAGGCGCTCAAGGAGATCCAGTCCGAGCACGCCACCGTCAAGCGCGAC
GGCCGCTGGAAGCATGGGCTGCCCGCGCGCGACCTCGTCATCGGAGACATCGTCGAGCTC
CGCGTCGGCGACAAGGTCCCCGCGGACATGCGTGTGCTCCAGCTTATCAGCTCTACCCTT
CGTGTTGAGCAGGGATCCCTCACTGGCGAGACCTCTTCGGTTAACAAGACCAGCCACAAG
ATTCATCTCGAGGACACAGATATCCAGGGGAAGGAGTGCATGGTCTTTGCTGGCACCACC
ATTGTCAACGGCAGTGCCGTCTGTGTCGTGACGGGAACTGGCATGTCCACTGAAATAGGC
AAGATCCATTCGCAGATCCAGGAGGCATCGCAGGAGGAGGATGACACACCGCTGAAAAAG
AAGCTCAATGAGTTTGGTGAGGCGCTCACTGCCATTATTGGAGTGATATGCATCTTGGTT
TGGCTCATTAACGTGAAGTATTTCCTCACCTGGGAGTATGTGGATGGGTGGCCAACGAAT
TTTAAGTTCTCGTTCGAGAAGTGCACATATTACTTTGAGATTGCAGTGGCACTGGCTGTT
GCTGCAATTCCAGAGGGCCTGCCTGCTGTCATCACCACATGCTTGGCACTAGGAACCAGG
AAGATGGCTCAGAAGAATGCACTTGTCAGAAAGTTACCAAGTGTTGAGACATTAGGTTGC
ACAACAGTGATTTGCTCTGATAAGACAGGAACTCTGACCACCAACCAGATGTCAGCGGTG
AGGCTTGTGGCAATTGGGAGGTGGCCCGATACACTTAGGAACTTTAAGGTTGATGGTACC
ACTTATGATCCAAGTGATGGCAAGATACATGACTGGCCAACTTTGAACATGGATGATAAC
CTCCAGATGATCGCGAAGATAGCTGCACTCTGCAATGATGCAAGTATTGCACACTCTGAG
CATCAGTATGTTGCTACTGGGATGCCCACAGAGGCTGCATTAAAGGTTTTGGTCGAGAAA
ATGGGGCTCCCTGGTGGATATACTCCATCACTGGATTCATCTGATTTGCTAAGGTGCTGT
CAATGGTGGAACAACGATGCCAAAAGAGTAGGGACTCTGGAATTTGACCGTACTAGAAAA
TCAATGGGAGTTATTGTGAAAAAAGCTGAGACTGGAAAGAATTTGTTGCTTGTGAAGGGG
GCAGTAGAAAATCTGCTAGAGAGAAGTGCCTATATTCAGTTACTTGATGGATCCGTCGTG
CTCTTGGATGAGGGTGCCAAGGCACTCGTATTGTCAACACTTAGGGAAATGTCTGGCAGT
GCGTTGCGCTGTTTGGGCTTTGCATACAAGGAAGATCTGGCAGATTTTGCAACATATGAT
GGAGAAGAACATCCCGCTCACAAATATCTACTTGATCCTGCATACTACTCTTCCATTGAG
AGTAATCTGATATTCTGTGGTTTTGTTGGTCTAAGGGACCCTCCACGAGAAGAAGTCCAC
AAAGCAATTGAAGATTGCAGGGCTGCTGGTATACGTGTTATGGTGATAACAGGTGATAAC
AAAGAAACAGCAGAGGCAATATGCCGTGAGATTGGGGTTTTTGGCCCCAGTGAAAATATT
AGCTCCAAGAGCTTTGCAGGGAAGGAATTTATGGCTCTTCCTGATAAGAAGAAGCTGTTG
AGGCAAACAGGTGGCCTTCTCTTCTCAAGGGCAGAGCCAAAACATAAGCAAGAAATTGTT
AGGCTGCTCAAAGAAGATGGTGAAGTCGTTGCAATGACTGGTGATGGAGTGAATGATGCA
CCGGCTCTGAAGTTAGCTGATATTGGAATTGCGATGGGTATTACGGGGACTGAGGTTGCA
AAAGAAGCTTCAGATATGGTGCTTGCAGATGATAACTTCAGTACAATTGTCTCAGCTGTT
GGTGAAGGAAGGTCTATTTACAACAACATGAAGGCTTTTATAAGATATATGATATCTTCG
AACATTGGAGAAGTTGCCTCCATATTCCTCACGTCGGCTTTAGGCATCCCAGAAGGCCTC
ATTCCTGTGCAACTTCTGTGGGTCAATCTTGTTACGGATGGCCCTCCTGCAACAGCATTA
GGATTCAATCCACCAGATAAGGATATCATGAAGAAACCTCCTAGAAGAAGTGATGATTCA
TTGATCACTCCTTGGATCCTGTTCCGCTACATGGTTATTGGGCTGTATGTTGGGATTGCA
ACAGTGGGGATCTTTATCATCTGGTACACTCATGGCTCTTTCCTGGGAATTGATCTGGCT
AGTGATGGTCACACTCTTGTTTCATACTCCCAGCTCTCAAACTGGGGCCAGTGCCCCTCA

TGGGAGGGTTTCAATGTGTCATCATTCACAGCAGGGGCAAGGACATTCAACTTTGATGAA
AACCCTTGTGATTACTTCCAGGGTGGCAAAATAAAAGCGACAACCCTCTCCCTGTCCGTC
TTGGTGTCCATTGAGATGTTCAACTCACTGAATGCCCTGTCTGAAGATGGCAGCCTTCTG
AGCATGCCTCCATGGGTGAACCCCTGGCTTCTTCTGGCAATGTCGGTGTCTTTTGGGCTT
CATTTTTTGATCCTCTACGTGCCCTTCCTTACCCAAATCTTCGGGATTGTGCCCCTCAGT
TTCAACGAATGGCTTTTGGTGGTAGCAGTGGCCTTCCCAGTGGTCCTCATCGATGAGGTT
CTTAAGTTTGTTGGGCGGTGCTTGACAGCTCGTGCAAGAAAACAATTAGGAAAGCGGAAG
GAAGAGTAG
>HvECA2
ATGGGCGAGGCCGGCCACGACGCGCCGGCGGCGGTGAAGGAGGGGTTCCCGGCGTGGGCG
CGGAGCGTGGAGGAGTGCGAGAAGCGGTTCGGGACGGACCGTGAGCGCGGGCTGACGTCC
GGCGAGGCGGCGGCGCGGCTGCGCGCGCACGGGCCCAACGAGCTGCTGGAGCACCCGGGG
CCGTCGGTGCTGCAGCTCGTGGCGCAGCAGTTCGAGGACACGCTGGTGCGCATCCTGCTG
GCGGCCGCCGCCGTCTCCTTCGCGCTCGCGCTCTCGTCGTCGGCCGGCGCGCTCACGCTC
TCGGCCTTCGTCGAGCCGCTCGTCATCTTCCTCATCCTCGTCGTCAACGCCGCCGTCGGG
GTCTGGCAGGAGACCAACGCCGAGAAGGCGCTCGAGGCGCTGCGCCAGATCCAGTCCGAC
CACGCCGCCGTGCTGCGGGACGGCGAGTGGGCGCCCGCCCTCCCCGCGCGCGACCTCGTC
CCGGGCGACGTCGTCATGCTCCGCGTCGGGGACAAGGTCCCCGCCGACATGCGCGTCCTC
AGGCTCGTCTCGTCGACCCTCCGGGTCGAGCAGGGGTCGCTCACCGGCGAGACCAACTCC
GTCAACAAGACGGCCCACGCCGTGCCCGCCGAGGACGCCGACATCCAGGCCAAGGAGTGC
ATGGTGTTCGCGGGCACCACCGTCGTCAATGGCAGCGCCGTCTGCCTCGTCGTGCACACC
GGGATGGCCACCGAGATCGGCAAGATCCACTCGCAGATCCACGAGGCCTCGCAGGAGGAC
GACGACACCCCGCTCAAGAAGAAGCTCAACGAGTTCGGCGAGGCGCTCACCAAGATCATC
GGCCTCATATGCATCCTCGTGTGGCTCATCAACGTCAAGTACTTCTTGACCTTCGAGCTC
GACGGATGGGTGCCCAGGAACATACGCTTCTCCTTCGAGAAGTGCACATACTACTTTGAG
ATCGCCGTGGCGCTTGCCGTCGCCGCCATACCCGAAGGATTGCCTGCCGTGATCACCACC
TGCCTCGCCCTGGGCACCAGGAAGATGGCCGCCAAGAATGCACTTGTCAGGAAGCTGCCC
AGTGTGGAGACTCTGGGCTGCACCACCGTGATCTGCTCCGACAAGACCGGGACGCTGACC
ACCAACCAGATGTCTGTCTCAAAGCTTGTTGCCATCGGTGATGCCCCAGGGAAAGTGAGG
AGCTTCAAGGTGGACGGCACGTCGTATGATCCACGTGATGGCAAAATTTACGACTGGCCT
GCAGGGAGGATGGATGCAAATCTTGAGATGATCGCAAAGGTTGCCGCTGTGTGCAATGAT
GCCAGCGTCTCACATTCTTCAAACCAGTATGTTTCCACCGGAATGCCGACCGAGGCCGCT
TTAAAGGTCCTGGTGGAGAAAATGGGTGTACCTGAAGGAAAGAATGGCCTGTCGGTGGAT
CCGTCGACATTAGGCTGCTGCCGATGGTGGAGCAATGCTGCCAAAAGGATCGCCACGCTC
GAGTTCGACCGTATGAGGAAATCAATGGGAATCATCGCTACCTCCAAATCAGGAGGCAAC
ACTTTACTTGTTAAGGGAGCTGTTGAAACCTTGCTGGAGAGGAGTAGCCATATTCAGCTC
CAGGATGGTTCAGTTGTGCCTTTAGACGAGAAATCAAGAAAAGCCGTTTTGGCAAGTCTC
CATGAATTGTCAACAAAAGCTCTGCGGTGCCTCGGATTTGCATACAAGGAGGATCTCGGT
GAATTTGCAACATATGATGGCGAATACCACCCTGCTCACAAGCTTTTGCTGGATCCAGCC
AATTACGCGGCAATTGAAACTGACCTGATATTTGTTGGTCTTGCTGGCCTAAGGGATCCT
CCGAGGGAAGAGGTCTTCGATGCTATTGAGGACTGCAGAGCTGCGGGCATCCGTGTTATG
GTGATTACAGGAGACAACAAAGAAACTGCTGAAGCGATATGCCATGAAATTGGTGTATTT
TCACCTGATGAAGACATTACCTTGAAGAGCTTTACGGGGAGGGAGTTCATGGCACTTGAG
GATAAGAAGACATTGCTGCGAAGGAAAGGTGGCCTTCTGTTCTCTAGAGCAGAGCCTAGG
CACAAGCAAGAGATCGTGAGGCTGTTAAAAGAAGATGGTGAAGTTGTTGCTATGACTGGA
GATGGAGTAAATGATGCCCCTGCTCTAAAACTTGCTGACATTGGTATAGCAATGGGTATT
ACCGGCACTGAGGTTGCCAAAGAGGCTTCTGACATGGTACTAGCTGACGACAATTTCAGT
ACCATAGTTGCTGCAGTTGGTGAAGGAAGATCTATTTACAACAACATGAAAGCTTTCATA
AGATACATGATTTCTTCAAACATTGGTGAAGTTGCCTGTATCTTCCTTACCTCTGCTTTG

GGTATTCCTGAGGGGTTGATACCTGTTCAGCTTCTATGGGTAAATCTTGTCACTGACGGC
CCCCCTGCAACTGCTTTGGGTTTCAACCCACCTGACAAGGACATTATGAAGAAACCACCA
AGGAGGAGCGACGACTCACTGATCACTCCCTGGATTCTGTTCCGTTACCTGGTCATTGGC
CTTTACGTGGGGGTTGCAACCGTTGGTATCTTTGTGATCTGGTACACCCATGGATCTTTC
ATGGGTATTGATCTCACTGGAGACGGCCACACACTTGTCAGCTACTCACAGCTCTCAAAC
TGGGGCCAGTGTTCTACCTGGGATAACTTCACAGTTGCACCCTTCACTGCTGGTGCTAGA
ACTTTCACCTTCGACGACAACCCCTGCGACTACTTCCAGGCCGGCAAAGTGAAGGCGACG
ACGCTCTCTCTGTCCGTGCTCGTGGCGATTGAGATGTTCAACTCTCTCAACGCCCTCTCT
GAGGACACGAGCCTGCTGAGGATGCCTCCGTGGGTCAACCCGTGGCTGCTCCTGGCCATG
TCGGTGTCGTTCGGGCTGCACTTCCTCATCCTCTACGTGCCGTTCCTCGCACAGGTGTTC
GGCATCGTGCCGCTCAGCCTGAACGAGTGGCTCCTGGTGCTCCTTGTCGCGCTCCCCGTG
GTGCTCATCGACGAGGTCCTCAAGTTCGTGGGCAGGTGCATGACCGCTTCAGGCCCCAAG
CGACGCTTAAAAAAGCAGAAGGGCGAGTGA
>HvECA3
ATGGAGGACGCCTACGCCAAGTCCGTCGCGGAGGTGCTCGAAGCTTTCGGCGTGGACCGG
ACCAAGGGCCTCTCCGACTCGCAGGTGGAGCAGCATGCGTTGCGCTACGGCAAAAATGTG
CTGCCCCAAGAAGAAAGTACCCCCTTCTGGAAGTTAGTTTTGAAGCAGTTTGATGATTTA
CTTGTCAAAATATTGATAGCAGCTGCCGTGGTATCCTTCCTTTTGGCTCGATTGAATGGT
GAAACTGGATTAACAGCATTTTTGGAACCATCTGTCATATTTATGATATTAGCAGCAAAT
GCAGCAGTTGGTGTGATCACAGAAACAAATGCTGAAAAAGCTCTCGAGGAGTTGCGAGCA
TATCAAGCAGATGTTGCAACAGTGCTGCGCAATGGTTGTTTTTCTATACTTCCAGCAACG
GAACTTGTCCCTGGAGATATTGTAGAAGTGGGAGTTGGTTGCAAAGTTCCAGCTGACATG
AGAATGGTTGAAATGCTAAGCCATCAATTGCGTGTTGACCAGGCAATTCTAACAGGGGAA
AGCTGTTCAGTGGCTAAAGAGCTTGATTCAACTTCAGCAATGAATGCTGTCTACCAGGAC
AAAACAAACATTCTTTTCTCGGGTACTGTTGTTGTAGCTGGTAGAGCAAGAGCTGTCGTT
ATTGGTGTTGGTTCTAATACTGCAATGGGAAGTATACGTGATGCAATGCTGAGAACAGAG
GATGAAGCAACTCCACTGAAGAAAAAACTGGACGAATTTGGCACATTTCTGGCAAAGGTT
ATAGCAGGGATCTGTATTCTTGTTTGGGTTGTAAATATTGGCCATTTCCGAGATCCTTCT
CATGGTGGCTTTCTTAGGGGTGCCATTCATTATTTTAAGGTAGCAGTTGCCCTTGCTGTT
GCAGCCATTCCAGAAGGTCTCCCAGCTGTAGTAACAACGTGCTTAGCTCTTGGTACCAAG
AGAATGGCTCGCTTGAATGCTATCGTCAGATCTTTGCCCTCTGTGGAGACATTAGGATGC
ACAACAGTCATTTGCAGTGACAAAACTGGCACTCTTACTACAAACATGATGTCCGTGTCT
AAGGTATGTGTTGTGCGGTCTGTGCACCAGAGACCAATAACTGATGAATACTCTATTAGT
GGAACCACATTTGCTCCTGATGGGTTTATATATGATGCCAGTGAAAATCAGCTGGAGTTT
CCTCCTCAATCCCCCTGCCTCCTTCATATAGCCATGTGTTCAGCTCTTTGCAATGAGTCA
ACTTTACAGTACAATCCTGATAAAAAAAGTTATGAGAAAATTGGAGAGTCCACTGAAGTT
GCTCTGCGCGTTCTGGTGGAGAAGGTTGGTCTTCCTGGTTTTGATTCGATGCCCTCAGCC
CTGAACATGCTAACTAAGCATGAGCGTGCATCCTACTGCAATCATTATTGGGAAAATCAG
TTCAGAAAGATATCAGTTCTAGACTTTTCCCGAGATCGCAAAATGATGAGCGTCCTTTGC
AGTCGAAAACAACAGGAGATTATGTTTTCAAAAGGTGCCCCTGAAAGTGTAATGGCAAGA
TGCACACATATATTGTGCAATGATGATGGGTCTTCTGTACCATTGACTATGGACATACGT
AATGAGCTGGAAGCTAAGTTTCAAAGTTTTGCAGGAAAAGATACCCTAAGGTGCTTAGCA
CTAGCATTAAAACGAATGCCAGAAGGCCAACAAAGTCTTTCCTATGACGATGAGGCAAAT
CTCACATTTATTGGATTGGTTGGGATGCTTGACCCTCCAAGAGACGAAGTTCGCAGTGCT
ATCCATTCTTGTATGTCAGCAGGGATTCGTGTTATTGTTGTTACTGGGGATAACAAGTCC
ACTGCAGAATCTCTATGTCGGCAAATTGGTGCGTTTGAGCATTTAGATGACTTTACAGGG
TATTCATATACAGCATCAGAATTTGAAGGGCTTCCTCCTTTGGAAAGGGCAAATGCATTG
CGAAGGATGGTTCTGTTCTCCAGAGTGGAACCTTCTCATAAGAAGATGCTTGTTGAAGCC
TTGCAATCACACAATGAAGTGGTTGCAATGACTGGTGATGGCGTCAATGATGCACCTGCG

CTGAAAAAAGCAGATATAGGAATAGCTATGGGATCAGGAACTGCTGTTGCAAAGAGCGCT
TCAGACATGGTATTGGCAGATGACAACTTCGCTACAATTGTTGCAGCTGTTGCAGAGGGA
AGGGCCATATATAATAACACCAAGCAGTTTATTCGATACATGATTTCATCAAATATTGGG
GAGGTAGTTTGTATTTTTGTGGCAGCGGTGCTTGGGATGCCTGACACACTTGTACCTGTC
CAGCTACTCTGGGTAAACCTTGTTACTGACGGGTTGCCTGCAACTGCAATTGGTTTTAAC
AAGCCTGATGGCAACATCATGGCAGTTAAGCCTCGTAAGGTGAACGAAGCTGTGGTCAGT
GGATGGCTCTTCTTCCGCTATTTAGTCATTGGTGCTTATGTTGGTCTTGCCACTATAGCG
GGGTTTGTTTGGTGGTTTGTTTATTCTGAAGATGGTCCTAGATTACCATACTCTGAATTG
GTCAATTTTGATTCATGTTCAACCAGGCAAACTTCATATCCTTGCAGCATCTTTGAGGAT
CGCCATCCATCAACTGTTTCAATGACTGTTCTTGTTGTTGTCGAGATGTTTAATGCCTTG
AATAACCTAAGTGAAAATCAATCCCTACTTGTCATTCACCCATGGAGTAACCTATGGCTT
GTTGGGTCAATCATTCTGACAATGCTTCTTCACGTAGCAGTCTTGTATACGGAACCCCTG
TCGTCTCTTTTCTCAGTATCTCCATTAACTTTGGCTGAGTGGAAAGTTGTTCTTTATCTA
TCCTTCCCTGTAATTTTGATTGATGAGGTGTTGAAATTTTTCTCAAGAAGACCTCGAGCT
TGGAGTTTTCCTTTACGGTTATGGAGACGTGAGATGCTTCCAAAAGAAGCCCGGGATAAT
TAA
