# Supplementary material for: A two-sequence motif-based method for the inventory of gene families in fragmented and poorly annotated genome sequences
Source: BMC Genomics. 2024 Jan 3;25:26. doi: 10.1186/s12864-023-09859-4 (PMC10763278; doi:10.1186/s12864-023-09859-4)
Supplement: Supplementary file 3 — Additional file 3: Supplementary file 3. Hordeum vulgare P2B ATPase coding sequences. [file 12864_2023_9859_MOESM3_ESM.pdf]

Supplementary File 3 – *Hordeum vulgare* P2B ATPase coding sequences

>HvACA1
ATGGAGAGCTACCTCAACGAGAACTTCGGGGGCGTCAAGCCCAAGCACTCGTCCGACGAG
GCGCTGGGCCGATGGCGCAAGGTCGTCGGCGTCGTCAAGAACCCAAAGCGCCGCTTCCGC
TTCACCGCCAACCTCGGCAAGCGATCCGAGGCCGCCGCCATGAAGCGGACCAACCAGGAG
AAGCTGCGTGTTGCTGTGCTTGTTTCAAAGGCTGCACTTCAGTTCATCCATGGTCTTGCT
CCCCAGAGCGAGTACACAGTCCCTGCCGCCATCAAGGCAGCAGGCTACAGTATCTGTGCC
GAGGAACTGAGCTCTGTTGTTGAGAGCCACGACCTCAAGAAGCTGAAAGTACATGGTGGC
ACCGAGGGCCTCATATCCAAGGTGTCTACCTCAGAGTCGGATGGCCTCAGCACATCCAAG
GACAAGCTTGCGTCCCGGCAGGAGATCTTTGGCATCAACAAATTCGCCGAGACGGAGGCC
CGCAGCTTCTGGGTCTTTGTCTGGGAGGCACTCCAGGACATGACACTCATGATCCTTGCT
GCATGTGCCTTCTTCTCGCTCGTCGTCGGCATTGCCACTGAGGGGTGGCCCAAGGGCGCG
CATGATGGCCTCGGCATCGTGGCTAGTATTCTTCTGGTTGTGTTTGTCACTGCGACAAGC
GACTACCGGCAATCCCTCCAGTTCAAGGACCTTGACAAGGAGAAGAAGAAGATCACGGTG
CAGGTCACCCGGAGTGGGTACCGGCAGAAGCTCTCGATATACGAACTTCTCGTTGGTGAC
ATCGTGCACCTTTCCATTGGTGATCAAGTACCAGCTGACGGTTTGTTCGTATCAGGATTC
TCGTTGCTGATTAATGAATCAAGCCTGACTGGGGAGAGTGAACCAGTTGCTGTCAATGCC
GAGAACCCATTCCTTCTGTCTGGTACTAAAGTGCAGGATGGTTCTTGCAAGATGCTTGTC
ACGACAGTCGGCATGAGGACTCAATGGGGTAAACTCATGGCCACCCTCAGTGAAGGTGGG
GATGATGAGACGCCATTACAGGTCAAGCTCAACGGTGTTGCCACCATCATTGGCAAAATA
GGCCTCGTCTTTGCTGTTGTCACATTTGCAGTGCTCACTGAAAGCCTGTTCCGCCGCAAG
ATCATGGATGGTTCGTACTTGAGTTGGAGTGGAGATGATGCACTGGAGCTGCTTGAGTTC
TTTGCTATTGCTGTTACCATTGTTGTCGTGGCAGTTCCTGAAGGCTTACCGCTTGCTGTG
ACCTTGAGCCTTGCATTTGCCATGAAGAAGATGATGAATGACAAGGCACTTGTCCGACAC
CTTGCAGCCTGTGAGACCATGGGCTCAGCAACCTCCATTTGCAGCGACAAGACTGGCACA
CTCACAACGAATCACATGACTGTCGTCAAGGCTTGCATCTGTGGCAAAATTAAAGAAGTG
GATAAATCTTCAGACACCAAGAGCCTATTCTCTGAGCTACCTGATTCTGTCATGACAATG
CTCTCGCAGTCCATCTTTAACAACACTGGTGGCGATGTTGTCATCAACCAGGGTGGCAAA
CGCGAAATACTGGGCACACCAACTGAGACAGCAATTCTAGAGCTTGGCCTGTCACTCGGT
GGTGATTTCCAGGCAGTGCGAAAAGCAACCACCCTCATCAAAGTCGAGCCATTTAACTCA
GCGAAGAAGAGAATGGGAGTGGTCATCCAGCTGCCAGGGGGTGCATTCCGTGCTCACTGC
AAAGGTGCATCAGAGATCATATTGGCATCTTGCAGCAAGTACCTGAATGATCAAGGCAAT
GCCGTCCCCCTTGATAGTGCAACTATGGCTCACTTGAATGCCACGATTGAGAGCTTTGCA
AATGAGGCACTTCGCACTCTATGCCTTGCTTACATTGAAGTTGCAGATGGGTTCTCAGCT
AATGATGCGATTCCTGAAGAGGGGTACACATGCATTGGCATTGTTGGGATCAAGGACCCA
GTGCGCCCAGGTGTGAAGGAATCTGTCGCCATCTGCAGGTCAGCCGGTATTACTGTCAGG
ATGGTCACGGGCGACAACATCAACACAGCAAAGGCAATTGCCCGGGAATGTGGCATTTTG
ACTGAAGGTGGTCTTGCCATTGAAGGCCCAGATTTTAGAATTAAAAGTGCAGAAGAAATG
TACGAATTGATACCGAAGATACAGGTAATGGCTAGGTCTTCACCACTTGACAAGCACACC
TTAGTGAAGAATCTCCGGACTACACATGAAGAAGTTGTTGCGGTGACTGGTGACGGACA
AATGATGCACCCGCACTTCACGAGGCTGATATTGGACTTGCGATGGGCATTGCTGGAACT
GAGGTTGCGAAAGAGAGTGCCGATGTCATCATTCTTGATGACAATTTCTCCACGATAGTC
ACAGTTGCCAAATGGGGTCGATCTGTGTACATCAACATTCAGAAGTTTGTGCAGTTTCAA
CTGACAGTCAACGTGGTTGCCCTCGTTGTGAACTTCTCGTCAGCTTGCTTGACAGGGAGT
GCTCCCCTTACTGCTGTTCAGTTGCTCTGGGTCAATATGATCATGGACACACTAGGAGCA
TTGGCATTGGCCACAGAACCTCCAAACGACGAACTGATGAAGAGGACTCCTGTTGGAAGG
AAAGGAAACTTCATCAGCAACATTATGTGGAGGAACATTATGGGACAGGCAATCTACCAG
TTCTTTGTAATTTGGTATCTGCAGACTGAAGGAAAGACACTGTTTGCAATTAAGGGTGAC

AATTCCGATCTAGTCTTGAACACTCTCATCTTCAATTGCTTTGTATTCTGCCAGGTGTTC
AACGAGGTGAGCTCCAGAGAGATGGAGAGGATAAATGTATTCAAAGGCATTCTAAACAAC
AACGTGTTCGTCGCTGTGCTCGGCAGCACGGTCATATTCCAGATCATCATAGTACAGTTC
CTTGGCGATTTCGCAAACACCACTCCTCTGTCACTCAAGGAGTGGTTCTCCTGCATTGTC
ATCGGTTTCATAGGCATGCCTATCGCTGCGATAGTCAAGCTTATCCCGGTGGGTTCTCAG
TAGG
>HvACA2
ATGGAGAGCTACCTGGAGGAGAACTTCGGGGGCGTCAAGGGCAAGAACTCGTCCGAGGAG
GCGCTGCGGCGCTGGCGCAAGCTCTGCAGCGTCGTCAAGAACCCCAAGCGCCGCTTCCGC
TTCACCGCCAACCTCGACAAGCGCGGCGAGGCGCAGGCCATCAAGCACGCCAACCACGAG
AAGCTGCGGGTTGCCGTGCTGGTGTCGAAAGCTGCACTGCAGTTTATACATGGTCTCAAA
CTTCGAAATGAATATGTTGTCCCTGAAGAAGTCAAGGCGGCAGGGTTCCAGATTTGCGCT
GATGAGCTGGGGTCCATTGTTGAGGGCCATGACAGTAAAAAATTGCTCACCCATGGTGCA
GTTGCTGGAATAGCAGCGAAGCTTGCAACATCCCCGACGGATGGGCTGGATACAGCTGAG
GACAGCATGCAGCGTAGGCAGGACATATATGGAATAAACAAATTCACAGAAAGCGAGATC
CGCAGTTTCTGGGTGTTTGTGTGGGAAGCTCTTCAAGATACAACTCTTATAATTCTTGCT
ATCTGCGCCTTTGTCTCGTTAGTTGTTGGCATTACGATGGAAGGATGGCCAAAAGGTGCT
CATGATGGTCTAGGAATTGTTGCAAGTATCCTCTTGGTCGTTTTCGTTACCGCGACAAGT
GACTATCGGCAGTCGCTGCAATTCAAGGACCTGGACAAGGAGAAAAGAAAAATTCAAGTG
CACGTTACAAGGAAAGGTTTCAGGCAAAGAATATCAATATATGATCTTCTTCCTGGAGAT
GTCGTCAATCTGGCAATCGGAGATCAGGTTCCTGCTGATGGGCTCTTCATTTCTGGGTTT
TCTCTGTTGATTAATGAGTCCAGCCTAACTGGTGAGAGTGAACCTGTTGTGGTAAATGAA
GAAAACCCTTTTCTTTTGTCGGGTACCAAGGTCCAAGATGGGTCATGCAAGATGCTTGTT
ACAACAGTTGGTATGCGAACCCAGTGGGGAAAACTAATGGCTACTCTCAGCGAAGGCGGG
GATGATGAAACCCCACTGCAGGTCAAACTTAATGGAGTGGCAACTATCATTGGTCAGATC
GGGCTATTTTTCGCTGTCATAACTTTCATTGTCTTGTCGCAAGGTTTACTCGGCAAAAAG
TATCACGATGGGCTGCTTTTAAGCTGGTCAGGGGATGATGCACTGGCGATGTTGGAGCAT
TTTGCTATTGCAGTTACCATTGTTGTGGTTGCTGTTCCTGAGGGATTGCCCTTAGCAGTC
ACACTGAGTCTGGCATTTGCAATGAAGAAAATGATGAATGACAAGGCACTGGTTCGCAAC
TTAGCTGCATGTGAAACTATGGGCTCAGCTACTACCATTTGCAGTGATAAGACAGGAACA
CTAACAACTAATCATATGACTGTTGTCAAGACCTGCATTTGTGGAAATATCAGAGAGGTT
AACAATCCTCAGAATGCCTCCAAGTTGCGTTCAGAACTTCCAGAAAATGTTGTCAGAACT
CTTCTCGAGTCTATATTTAACAATACAGGTGGTGAGGTTGTTATTGACCAAAACGGGAAA
CACCAGATCCTTGGTACCCCAACAGAGACAGCCATATTGGAGTTTGCAATGTCAATAGGT
GGAAACTTTAAGGCAAAGCGTGCTGAAACTAAGATTGCGAAAGTGGAGCCTTTCAATTCA
ACAAAAAAGAGGATGTGTGTCCTTCTTGAGCTTGCCGAAGGAGGATACCGTGCACATTGT
AAAGGTGCTTCAGAAATAGTCTTGGCTGCATGTGATAAGTTCATAGATGAGACAGGTGCT
GTTACCCCTCTTGATAAAGCAACTGCTGGCAAGCTCAATGGTATTATTGATGGTTTTGCT
CATGAAGCTCTTAGGACATTGTGCCTTGCTTACAGGGAAATGGAAGAAGGTTTTTCCATT
GAAGAGCAATTACCACTGCAAGGGTACACATGCATTGCTATTGTAGGTATTAAAGATCCT
GTTCGCCCAGGTGTGAGAGAGTCTGTTGCAATTTGCCGCTCTGCTGGAGTTACGGTGAGA
ATGGTCACAGGCGACAACATAAATACAGCAAAGGCGATTGCCCGTGAATGTGGTATACTC
ACTGAAGATGGTCTGGCTATTGAGGGACCGGATTTCAGGGAGAAAACTCTTGAGGAACTC
CTTGTGCTTGTTCCAAAAATTCAGGTAATGGCCCGATCATCACCCCTGGATAAGCATACA
CTTGTAAAGCATTTACGCACAACATTCAATGAAGTTGTTGCTGTTACTGGTGACGGCACA
AATGATGCTCCCGCTCTGCATGAAGCAGATATTGGACTTGCAATGGGCATTGCTGGGACT
GAGGTGGCGAAAGAGAGTGCCGATGTCATCATTCTGGACGACAACTTCTCTACAATTGTA
ACTGTTGCCAGATGGGGACGCTCTGTTTACGTCAACATTCAAAAATTTGTGCAGTTTCAG
TTAACTGTTAATGTTGTCGCCTTGCTTGTTAACTTCTCCTCAGCTTGTTTTACAGGAAAT

GCGCCACTGACGGCTGTTCAACTTCTTTGGGTCAACATGATTATGGACACACTTGGTGCG
CTTGCATTAGCCACCGAACCACCCAATGATGACTTGATGAAGAGAGAGCCAGTAGGAAGA
ACAGGGAAGTTCATCACAAATGTAATGTGGAGGAACATTTTTGGGCAATCTATATACCAA
TTTGTTGTCATGTGGTATCTCCAGACGCAAGGAAAAACCTTTTTTGGGCTTGAAGGCTCT
GATGCTGATATAGTGCTGAATACAATTATTTTCAACTCATTCGTCTTCTGCCAGGTGTTC
AATGAGATAAGTTCGAGGGAGATGGAGAAGCTCAATGTGCTCAAGGGCATTCTGAACAAC
TATGTCTTCATGTGTGTCCTCAGCAGCACGGTCGTCTTCCAGTTCATCATGGTCCAGTTC
CTCGGCGAGTTTGCCAACACGACGCCTCTCACCAGTCTCCAGTGGCTCGCCAGCGTGCTC
CTTGGCCTCGTCGGGATGCCGATCGCCGTCGTCGTCAAGCTCATTCCTGTTGGGTCCTCC
TGA
>HvACA3
ATGCACAGCGGTCTCCTTGCTCGGCCCCTCCAGCTCGCCCCCCTCTCCGGCGCCGCAGCC
GCTCTCCCCTGCCGTCGGATCCCATGGGCGTGCCTGGGCCTAGGCGTGTCGACGCCCGGC
CGCCGCGGCTTCCGCTTCGCCGCCGCCGCCTGCCAGCTCTCCAGCTCCAGCCCACCCCAC
CGTGAGAAGCTGCAGGTTGCTGTATCCGCATCGAAAGCCGCCGTGCAGCTACAAAATGGT
CTTTCACTTCAGAGTAGTCAGTACGTCGTCCCCGAAGACGTTAGAGCGGCAGGGTTTCAG
ATCGGCGCCGATGAACTGACATCCATCGTCGAAAGCCATGACACAGAAAGGCTGACTGAA
CACGGCCAGTTAGATGGGATTGCAGACAAGTTAGCGACGTCGTTGACCGATGGGATAAGC
ACGCGCGAGGACCTCTTAGAGCAGAGGCAGGAAATTTATGGCGTAAACAAGTTCGCTGAG
AGCGAGCCTCGCAGCTTCTGGGAGTTTGTATGGGATGCAGTGCAAGACACCACTCTGATA
ATCCTTGCTGCCTGTGCTTTCGTCTCTTTGACTGTCGGCATCGCCACCGAAGGCTGGCCC
AACGGCTCCCATGACGGCATCGGAATCTTCGCGAGTATCATCCTGGTTGTTTCTGTTACC
GCGACAAGTGACTACCAGCAGTCTTTGCAGTTCAGGGACCTGGACAAGGAGAAGAGGAAA
ATTCTTGTTCAGGTTACGAGGAACGGGTTCAGGCAAAGGATATTGATAGATGATCTTCTT
CCCGGCGACGTTGTCCATCTAGCGGTTGGAGATCAGGTTCCTGCAGACGGTGTCTTTATT
TCCGGGTTTTCTCTGTTGTTGGATGAGTCCAGTCTAACTGGAGAGAGCGAGCCTGTCGAT
GTAAGTGAAGACAAGCCTTTTCTTTCATCAGGGACCAAAGTCCTAGATGGGTCTGGCCAG
ATGCTGGTTACAGCGGTCGGGATGCGGACACAGTGGGGAAAACTAATGGCTGCTCTCACT
GAAGGGGGGAATGATGAAACTCCACTTCAGGTTAAACTTAGTGGAGTTGCAAATATTATC
GGGAAAATCGGTCTGTTTTTTGCTGTCTTAACTTTCGTTGTCCTCTCCCAAGAGTTAATT
GGCCAGAAATATCAGGATGGGCTTCTTTTAAGCTGGTCAGGAGATGATGTTTTAGAGATA
TTGAATCATTTTGCTGTCGCGGTTACAATTGTTGTCGTTGCTGTGCCTGAGGGGTTGCCA
TTGGCAGTCACACTGAGCCTTGCCTATGCAATGGAGAAGATGATGAATGACAAGGCACTG
GTCCGGCAGTTGGCTGCCTGCGAAACTATGGGATCAGCAACAGTCATTTGCAGTGACAAG
ACAGGAACACTAACATCCAATCGCATGACTGTTGTTAAGGCCTGCATTTGTGGGAACACC
ATGGAAGTTAACGGTCCGCTGATTCCTTCTAGTTTGTCTTCAAAACTCCCAGCAGTTGCA
GTAGAAACTCTTCTAGAATCCATACTTACCAACACTGGTGGTGAAATCGTGATTGACCAA
AATGGAAAGCAAGAAATAATCGGTACCCCTACTGAGACAGCTTTGTTGGAATTTGCACTA
TCATTAGGTGGAAACTATAAACAAAAACGGCAGGAAACTAAGATTCTTAAAGTGGAGCCT
TTTAATTCAGTAAAAAAGAGGATGACTGTTATTCTCGAGCTTCCTGGAGGAGGGTACCGT
GCACATTGTAAGGGTGCCGCAGAAATAGTATTGGCTGCCTGTGATAAGTTCATAGACGGC
AGCGGTAGTATTGTTCCCCTTGATAAGAAAACTGCCAATATGCTCAATGATATCATCGAA
ACCTTTTCTAGTGAAGCACTTCGAACACTATGCCTTGCCTACAGGGGATTGGAAGATGGC
TCTACTCAAGAGGAAATACCACTGCAAGGATACACATTCATTGGCATTGTTGGTATTAAA
GATCCTGTGCGCCCAGGTGTCAGGGAGTCTGTGGCAAGTTGCCGATCTGCTGGCATTGCG
GTTAAAATGGTTACAGGGGACAATATTAATACAGCAAAGGCAATTGCTCGTGAATGTGGT
ATACTTACTGATGGCGGTCTTGCCATTGAAGGTGCTGAGTTCAGAGAGAAAACACCTAAA
GAACTCCTTGAGCTGATTCCCAAAATGCAGGTGCTAGCCCGATCGTCGCCACTTGATAAG
CTTGCACTTGTGAAGCACTTGCGCACAACTTCCAATGAAGTTGTTGCTGTGACTGGTGAT

GGCACTAATGATGCTCCTGCGCTGCGTGAGGCAGATATTGGACTTGCCATGGGCATTGCA
GGGACTGAGGTGGCGAAAGAGAGCGCTGATGTCGTGATTCTGGATGACAACTTCTCCACG
ATTGTTACTGTTGCGAAATGGGGACGCTCTGTTTACATCAACATCCAAAAGTTTGTGCAG
TTCCAGTTGACCGTTAATGTCGTTGCATTACTAGTCAACTTCTCCTCCGCATGCTTTACA
GGAGATGCGCCGCTGACAGCTGTTCAACTTCTTTGGGTCAACATGATCATGGACACCCTA
GGTGCGCTAGCACTTGCCACCGAACCACCTAACGATAACTTGATGGAGAAAGCACCCGTA
GGAAGGACAGGTAAGTTCATCACAAACGTGATGTGGAGAAATATTCTGGGGCAGTCGTTG
TACCAATTCACCGTCATCTGGTACCTACAAAGTCAAGGGAGATATGTGTTTGGGCTTGAA
GGCTCCGAGGCTGATACTGTCCTGAATACAATCATATTCAACACTTTTGTCTTCTGCCAG
GTGTTCAATGAGGTCAGCTCAAGAGAGATGGAGGAGATCAATGTTCTGAAGGGCATGTCG
GAGAACTCTATTTTCGTGGGCGTTCTCGCCGGCACTGTCATCTTCCAGTTCATCCTGGTC
CAGTTCCTGGGCGACTTTGCCAACACCACCCCGCTGACCCAGCTCCAGTGGCTCATCTGC
GTCCTCTTTGGCTTCCTTGGAATGCCCATTGCCGCCATGATCAAGCTCATTTCCGTAGAA
GAGCGTGAAGAACACGACGATTATGGAAAGCTGTAG
>HvACA4_5
ATGGAGAAGTACCTGCAGGACAACTTCGACCTGCCGGCCAAGAACCCCTCCGAGGAGGCG
CAGCGCCGCTGGCGCTCCGCCGTCGGCAGCCTCGTCGTCAAGAACCGTCGCCGCCGCTTC
CGCCACGTGCCCGACCTCGACCAGCGCCACCAGGACGACGCCAAGCGCCGCTCCGTCCAG
GAAAAAATTCGGATTGCCCTCTATGTGCAGCAGGCCGCAATCACTTTCATTGGTGGCACC
AAGAAGAATGAGTACCAGCTAACAGATGATATAATCAAAGCTCGGTTTTCCATCAATCCC
GAAGAACTGGCATCAATAACAAGCAAGCATGACTTGAAAGCTTTAAAGATGCATGGTGGA
GTAGATGGGATATCCAAAAAAGTTCGTACAACATTCGACCGTGGTGTATGTGCTACTGAC
TTGGATACAAGACAAAGCATATATGGTGTCAATCGCTATGCTGAAAAGCCTTCAAGAAGT
TTCTGGATGTTTGTTTGGGATGCATTGCAAGACACGACTCTTATCATTCTGATGGTGTGT
GCTTTGTTATCTGTTGTAGTTGGCCTGGCATCTGAAGGTTGGCCCAAGGGAATGTATGAT
GGCTTGGGGATAATACTCAGCATTCTCTTAGTTGTGATGGTTACTGCTGCCAGTGACTAT
AAGCAATCGCTTCAGTTTAAGGAGCTAGATAATGAAAAGAAAAACATATTTATCCATGTA
ACCAGAGACGGTGGTCGACAGAAGATCTCGATATTTGACTTGGTAGTTGGTGATATTGTG
CATTTATCAATTGGGGACCAAGTGCCTGCTGACGGACTGTTTATACATGGATACTCCCTT
CTCATTGATGAATCCAGCTTGTCAGGTGAGAGTGAGCCAGTGTATACTTCTCAAGATAAA
CCATTCATATTGGCAGGAACTAAAGTGCAAGATGGTTCTGCTAAGATGATAGTAACGGCT
GTCGGTATGCGCACTGAATGGGGAAGGCTGATGAGCACTTTGAGTGAGGGAGGAGAAGAC
GAGACACCATTGCAGGTTAAGCTAAATGGAGTTGCGACCATCATAGGAAAGATTGGCCTG
ATATTTGCCACATTAACATTTGTAGTCCTGATGACTAGATTCCTTATTGACAAAGGTTTG
ACAGTTGGTTTGTCCAATTGGTATTCAGCTGATGCCTTGACTATAGTGAACTACTTCGCA
ACAGCGGTCACTATTATTGTTGTTGCTGTTCCCGAAGGGTTGCCGTTGGCTGTGACTTTG
AGCCTTGCATTTGCAATGAAGAAGTTGATGAATGACAAAGCGCTTGTCAGACACCTTGCA
GCATGTGAAACAATGGGATCTGCTGGTACCATTTGCACCGACAAGACAGGAACTTTGACT
ACTAACCATATGGTGGTTGACAAAATTTGGATAGCCGAGATTTCAAAGTCAGTTACAGGT
AACAATAGTTTGGAAGAACTGAATTCTGCGATTTCTTCAAGCGCATGGAGCCTACTTTTG
CAGGGCATTTTTGAGAACACTAGTGCAGAGGTGGTTAAAGGAAATGATGATAAACAAACT
GTTTTGGGCACTCCAACAGAGATAGCAATATTCGAATATGGCTTGAGCTTGCAAGGATAT
TGTGATGCTGAGGATAGGAGCTGCACCAAGGTTAAGGTTGAGCCTTTCAATTCGGTCAAG
AAAAAGATGGCAGTATTGGTTTCTTTATCTGGCGGGGGACACCGTTGGTTCGTCAAAGGT
GCATCAGAAATTATTGTTGAGATGTGTGACAAGGTGATTGATCAAGATGGAGATGTTATT
CCCTTATCAGATGATCGGAGAAAGAACATCACGGATACCATCAACTCATTTGCTTCAGAT
GCTTTAAGGACATTGTGCTTAGCGTTTAAGGATGTGGATGAATTTGATGAGAATGCAGAT
AGCCCTCCTAATGGTTTTACTCTGATAATCATATTTGGCATCAAGGACCCTGTGCGCCCT
GGAGTGAAGGAAGCTGTTCAGAGCTGCATAACTGCTGGCATCATTGTGAGAATGGTGACT

GGTGATAATATCAATACAGCTAAGGCTATTGCCAAAGAATGCGGCATATTGACTGATGAT
GGCATAGCAATAGAAGGACCAGATTTTCGTAACAAAAGCCCAGAAGAAATGAGGGACTTG
ATACCTAAGATTCAGGTTTGTTTCGTCTTTCTTGTTCTAGTCATGGCCCGTTCATTACCA
TTGGACAAACATTTGCTTGTGACGAACTTGAGAGGCATGTTCCATGAGGTGGTTGCTGTG
ACAGGTGATGGTACAAATGATGCTCCTGCATTACACGAAGCAGATATTGGGCTTGCTATG
GGCATTGCAGGCACAGAGGTTGCTAAGGAGAGTGCTGATGTCATAGTACTTGATGACAAC
TTCACAACTATAATAAATGTTGCGAGGTGGGGTCGTGCGGTTTACATAAACATCCAGAAG
TTCGTGCAGTTTCAGTTGACCGTGAACATTGTTGCTTTGGTGATCAACTTCGTCTCAGCA
TGCATCACAGGTAGTGCTCCTCTCACTGCTGTGCAGCTGCTGTGGGTGAACATGATTATG
GACACATTGGGAGCTTTAGCTCTAGCAACCGAGCCTCCAAATGATGAAATGATGAAGAGA
CCGCCTACTGGGCGAGGGGAAAGCTTCATCACCAAGGTCATGTGGAGAAACATCATTGGT
CAAAGTATATACCAGCTGATTGTGCTCGGCGTTCTCATGTTCGCTGGGGAAAATCTTCTC
AACATCAATGGTCCAGATTCCACGACCGTTCTCAACACCCTCATATTCAACTCCTTCGTG
TTTTGCCAGGTATTCAACGAAGTGAACAGCAGGGAAATGGAGAAGATAAACATTTTCCGC
GGTCTCATTGGCAATTGGGTCTTCCTTGGGGTAATAAGCGCGACGGTGGTATTCCAGGTG
GTGATCATCGAGTTTCTCGGCACTTTTGCGAGCACTGTTCCACTCAGCTGGCAATTCTGG
TTGGTGAGCGTTGGTATAGGATCCATCAGCTTGATTATTGGAGCCATCTTGAAGTGCATT
CCGGTAAAATCGGGTGAGATTTCTGGAAGTCCACATGGTTACAGGCCGCTTGCCAACGGC
CCTGATGACATATAA
>HvACA6
ATGGATTTTCTCAAGACCTTCGACGTGCCGGCGAAGAATCCGTCGGAGGATGCGCAACGC
CGGTGGCGGGAGGCCGTCGGCACGCTCGTCAAGAACAGGCGCCGGCGTTTCCGCATGGTC
CCCGATCTCGACAAGCGATCCCAGGCTGAGACGCAACGCCGCAACATCCAGGAAAAGCTT
CGCGTCGCGCTCTACGTGCAGAAGGCCGCTCTGCAGTTCATCGATGCTGCCCGCCGGGTG
GAGCACCCGCTGTCGGAGCTGGCGCGGCAGTCGGGCTTCTCCATCAGCGCCGAGGAGCTG
GCCTCGTTGGTGCGCGGCCACGACAACAAGAGCCTGCGCCTCCACAAGGGCGTCGAGGGC
CTGGCCCGCAAGGTGAACGTCTCCCTCGCCGACGGCGTCAGATCCGACGACGTGGGCGTC
CGCGGCGAGGTCTACGGCGCCAACCACTACCCCGAGAAGCCGGCCCGCACCTTCTGGATG
TACTTGTGGGACGCCAGCCAGGACATGACGCTCATGCTGCTCGCGCTCTGCGCCGTCGTC
TCCGTAGTCATCGGCATCGCTACCGAGGGCTGGCCCGGGGGCATGTACGACGGCCTCGGC
ATCATGCTCACCATCTCCCTCGTCGTCACCATCACCGCCGCCAGCGACTACAAGCAGTCG
CTCCAGTTCCGGGACCTCGACAGGGAGAAGAAGAAGATCGAGATCCAGGTCACGCGCGAC
GGCTTCCGCCAGAAGGTCTCCATCTACGACATCGTCGTCGGCGATATCGTCCATCTGTCC
ATCGGCGACCAGGTCCCGGCGGACGGGCTGTTCGTTGATGGCTACTCGTTCATCGTCGAC
GAGTCGAGCCTGTCCGGCGAGAGCGAGCCGGTGCACGTGTCGGCCACCAACCGGTTCCTG
CTGGGCGGGACCAAGGTGCAGGACGGGTCGGCGAGGATGCTGGTGACGGCGGTGGGGATG
CGGACGGAGTGGGGGAACCTGATGGAGACGCTGAGCCAGGGCGGCGAGGACGAGACGCCT
CTGCAGGTGAAGCTCAACGGCGTCGCAACCATCATCGGCAAGATTGGGCTCGCCTTCGCG
GTGCTCACCTTCACCGTGCTCATGGCGAGGTTCCTCATCGGCAAGGCTGATGCACCAGGC
GGGCTGCTGACATGGGGGATGGACGACGCGCTGTCGGTGCTCAACTTCTTCGCCGTCGCG
GTCACCATCATCGTGGTCGCGGTGCCGGAAGGCCTGCCGCTCGCCGTCACGCTCAGCCTG
GCGTTCGCCATGAAGAAACTCATGCAGGAGCGCGCTCTCGTGCGGCACCTCTCGGCGTGC
GAGACCATGGGGTCCGCCAGCTGCATCTGCACCGACAAGACCGGCACGCTCACCACCAAC
CACATGGTCGTCGAGAAAGTCTGGGCTGCCGGCGGGGCGACCACGGTGAGCACCGCCAAG
GGCTTCGAGGAGCTCACCTCGTCGGCGCTGTCGGAGGGGTTCGCCAAGCTTCTTCTGGAG
GGCGTCTTCCAGTGCTCCGGCTCCGAGGTCGTGCGCGGCAAGGACGGAAAGACCAGCGTC
ATGGGCACGCCCACCGAGTCGGCCATCCTCGAGTTCGGGCTCGGGGTGGAGAAGAACACC
TGCATCGAGCACGCGGCCGCCCCGAAGCTCAAGGTGGAGCCGTTCAACTCGGTGAAGAAG
ACCATGGGCGTGGTGGTCGCGTCCCCGAACGCCGGCGGCCGGCCACGAGCGTTCCTCAAG

GGGGCCTCCGAGGTCGTGCTCCGCCGGTGCAGCAACGTGGTCGTCGACCGCCACGGCAGC
ATCGTGGCCCTGACGGAGAAGAACTACGGGAAGCAGGTGGCCGGCGCCATCGACACGTTC
GCGTGCGAGGCGCTGCGCACGCTCTGCCTGGCGTACCAGGACGTCGCCAGCGAGAACGAG
GTCCCCAACGACGGTTACACGCTCATCGCCGTGTTCGGCATCAAGGACCCGCTCCGACCA
GGCGTGAGGGAGGCCGTGGAGACCTGCCACATCGCGGGGATCAACGTCCGCATGGTCACC
GGCGACAACATCAGCACGGCCAAGGCCATCGCGAGAGAGTGCGGCATCCTCACGGAGGAC
GGCGTCGCCATCGAGGGTCCCGAATTCCGGCAGATGAGCCCCGACCAGATGAGGGCGATC
ATACCAAAAATCCAGGTGATGGCGCGGTCGCTGCCGCTGGACAAGCACACGCTGGTGACC
AACCTGAGGGGCATGTTCAACGAGGTGGTGGCCGTCACCGGAGACGGCACCAACGACGCG
CCGGCGCTGCACGAGGCTGACATTGGCCTCGCCATGGGCATCGCCGGAACAGAGGTTGCC
AAGGAGAACGCGGACGTGATCATCATGGACGACAACTTCTCCACCATCATCAACGTCGCC
AAATGGGGCCGCTCCGTGTACATCAACATCCAGAAGTTCGTGCAGTTCCAGCTCACCGTC
AACGTGGTGGCCCTCATGGTCAACTTCGTCTCTGCATCCTTCACAGGGAGCGCGCCGCTG
ACGATCGTGCAGCTGCTGTGGGTGAACCTGATCATGGACACCCTGGGCGCGCTGGCGCTG
GCGACGGAGCCGCCGAGCGACGCCATGATGCGGAGGCCGCCGGTCGGCCGGGGCGACAAC
TTCATCACCAAGGTCATGTGGAGGAACATCGCCGGCCAGAGCATCTTCCAGCTCGTCGTG
CTCGGCGCCCTCCTCTTCAGAGGGGACAGCCTCCTGCACATGAACGGCGACGGCCAACTG
CTCAACACCTTCGTCTTCAACACCTTTGTCTTCTGCCAGGTCTTCAACGAGGTGAACAGC
AGAGAGATGGAGAAGATCAACGTCTTCAGCGGCATGTTCAGCAGCTGGGTCTTCTCGGCG
GTGGTCGGCGCCACCGTCGGGTTCCAGGTGATCCTGGTGGAGCTGCTGGGGACGTTCGCC
GGCACGGTGCACCTCAACGGGAGGCTGTGGCTCCTGAGCGTGCTCATCGGGTCGGTCAGC
CTGATCATCGGCGCCGTGCTCAAGTGCATCCCCGTCGGCTCCGGCGACGGCTCGTCGGAT
CGCCACGACGGATACCAGCCCATCCCCGCCGGCCCCGGCGCCGTCTGA
>HvACA7
ATGGAGGGCGGGAGCGGCAGCAGCTGGACGACGAGCATGGAGGGGTATCTGAAGGAGCAC
TTCCACATCCCGGCCAAGAACCCGCCCACCGCCGCCCGCCTCCGGTGGCGCCGCGCCGTC
GGCCTCGTCGTCCGCAACCGCCGCCGCCGCTTCCGCGAGTTCTCCGCCCTCGCCGCCGTC
GACGCCGCCCAGCGCCGCAAGATCCTGGGAAAAGTCCAGGTTGTGATTAACGTGCATAGG
GCAGCACTGCAATTTATTAACGGCGTAAAACAATACCACTTAACACATGAGCTTATTGAG
GAAGGATTTTCTATCAGCCCGGATGAACTAGCAGAAATTACTGGCATGCGCGAAGATTCG
ACAATCTTGAAGTTGCATGGTGGAACCAACGGGATATCTAGGAAACTCAAAGCCTCTTTG
CAAGATGGTGTCAAGGAAACTGAAGTATCAACCAGGCAGAAACTGTATGGTACTAACAAG
CACGCCGAGAAGCCCCCTAGAAGCTTCTGGATGTTTGTGTGGGATGCATTACATGACCTG
ACTCTCAACATTCTCATAGTGTGTGCTCTGGTTTCTCTGGTGGTTGGCCTAGCAACCGAG
GGGTGGCCCAAGGGTATATATGATGGTCTTGGCATAATACTCAGCATTTTGTTGGTGGTA
CTAGTTACTGCGTCCAACGATTACAAGCAGTCAAGGAAGTTTATGGAGCTGGACCGTGAG
AAGCAGAAGATATATGTCCTTGTTACTAGAGATAAGAAAACCAAGAAGGTTTTGATTCAT
GACTTGGTCGTCGGCGACATCTTGCATCTTTCGATAGGTGATGTGGTTCCTGCAGATGGC
TTGTTTATATCTGGCTACTGCCTACTGGTAGATGAATCTAGCTTGTCAGGTGAGAGTGAG
CCAATTCAAGTTTCTGAAGAAAAGCCTTTTCTCCATGGTGGGAGTAAGGTGGTTGATGGG
ACAGCTAAGATGCTTGTCACTGCCGTCGGTTCGCGTACCGAGTGGGGTAAAATCATGGGC
ACTCTTAGTGACAGTGGAGTGGATGAAACTCCTTTGCAAGTTAAGCTCAATGGCGTGGCT
ACGGTCATTGGGCAGATTGGACTCGTGTTTGCTATTCTCACGTTTCTAGTACTTCTAGCA
AGGTTCTTGGTTAACAAGGGAATGGGTGTCGGTTTGATGAATTGGTCGGCAAATGACGCA
TTGACAATAGTCAACTACTTTGCTATTGCCGTGACCATCATCGTAGTTGCAGTCCCTGAA
GGTCTACCGTTGGCTGTGACCCTTAGTCTTGCATTTGCCATGAAGAAGTTGATGAATGAC
AAAGCTCTAGTCAGGCATCTCGCGGCGTGTGAGACGATGGGTTCAGTCAGCTGTATTTGC
ACTGATAAGACAGGAACTTTGACAACCAACCACATGATCGTCGATAAGGTTTGGATCAGC
GACGTATCCAAGTCGGTCAATGGCGATGCAAAAATCACCGAGCTAAAATCTGTAATTTCA

GAAAGAGCTATGGAAATACTTGTACAAGGCATATTTGTGAACACGGGATCCGAGGTGGTG
AAGGGAGATGATGGCAAAAGGACCATCTTGGGCACACCAACCGAAGCAGCATTGTTGGAG
TTCGGCTTGACCATAGAAGCGGATCGATACCTTGAATACAATAGTATCAGGAGAGTAAGA
GTAGAGCCTTTTAATTCAGTCAAGAAAAAGATGTCAGTGATAATAGAGTTACCAAATGGA
GGCTTCCGTTCCTTCTGTAAAGGCGCACCGGAAATTATTCTAGGACACTGCGATAATGTC
CTGAATGGCGAAGGGGATATAGTACCACTGTCAGACATGCAGAAGCAAAATGTCCTAAAC
ATAATCAATTCATTTGCTTCTGAGGCGTTGAGAACACTTTGCGTTGCATTTCAGGATCTT
GATGAATTTTCTGAAGAGCAAACCATACCAGAAAATGGTTACACACTAATTGTGCTTTTT
GGTATCAAGGACCCAGTCCGTCCTGGTGTCAGGGATGCAGTGATGACCTGCATGGCTGCT
GGTATTACGGTAAGAATGGTTACGGGAGACAACATCAACACTGCGAAAGCTATTGCCAAG
GAATGTGGAATATTAACCGAGGACGGGATAGCCATAGAAGGACGGGAGCTTCACGATAAG
AGCTCCGACGAACTGAAGGAGCTACTACCTAAAATTCAGGTAATGGCCCGCTCGCTGCCT
ATGGACAAATTCAAATTGGTAACAAGCTTGAAAAGCATGTATCAAGAGGTAGTTGCTGTT
ACTGGTGATGGAACTAATGATGCCCCGGCATTGTGTGAGTCAGACATTGGATTGGCAATG
GGCATTGCTGGCACTGAGGTCGCAAAAGAGAATGCTGATGTTATAATAATGGATGACAAT
TTCAAAACTATCGTGAATGTTGCTAGATGGGGGCGTGCGGTTTACTTGAACATTCAAAAG
TTTGTGCAGTTCCAGCTTACAGTTAATATAGTGGCTCTGATAGTGAATTTTGTCTCGGCA
TGTGTCATAGGGACTGCACCTCTTACTGCTGTCCAGTTGCTATGGGTTAATATGATCATG
GATACATTGGGAGCCTTGGCCTTAGCAACGGAACCGCCAAATGATGAGATGATGAAGCGG
TCGCCTGTAAGGCGCGGAGATAGTTTTATCACTAAGGTTATGTGGAGAAATATTCTTGGC
CAAGCTTTGTATCAGCTCCTTGTATTGGGTACTCTCATGATAGTTGGAAAGAGACTCCTT
AATATTGAAGGTCCAACTGCTGATAAAACGATCAATACTCTCATATTCAACTCTTTCGTC
TTTTGCCAGGTTTTCAACGAAATAAACAGCAGGGAAATGGAAAAGATCAACGTCTTCCGA
GGGATATTCAGAAATTGGATCTTTGTCGGCATACTGACAGCTACAGTGATATTCCAAGTG
ATCATCGTGGAACTTCTCGGAACTTTTGCGAACACCGTGCCGTTGAGTTTAGAACTATGG
CTGCTCAGCGTCGTTCTTGGCTCGGTTAGTATGATCGTCTCCGTGATCCTCAAGTGCATT
CCAGTTGAATCTGTGAAGAGAGATGCGAAGCCTCATGGGTACGAGCTGATCCCTGAAGGT
CCGGAAGCTCTCTAG
>HvACA8
ATGGAGTGCGCCGACGTCCTGATCGCCGTGGGCCGCCGGTCGACGTCGCCTTCCCCGTCC
TCGTCCTGGCAGCCGGGGAGGCAATGGCGCAAGGCGCTCAACGTCATCCGGACGTGCCAC
AGGCTTGCGCGGCTCGGCATCCTGTCCGCCGGCGTCCTGCCACGGAGCACCTCCTCCTAC
GTCGCCATCAAGATCCACCACGACGGCAGCGACTCCGACGCTGACTTCTCGTCAGGTAAC
GCCAACGCCGCCGCATTCTCTGTCGCCGCGGACGACGAACTTTTCAAGGGTCTGGTCAAG
GAGAAGCGCGAAGACTGTTTCCGCCGCCTCGGGGGCGGCGCCGGCATCGCGGCCGCGCTG
GGGTCTGACGCCGACCGCGGCATCCACGGCGACGGCGACGACCTTCGGCGACGCAGGGAG
TCCTTCGGCGGGAACACGTACCCGAAGCCCAAGCCCAAGAGCTTCTTCAGCCACGTCTGG
GACGCGCTCAAGGACGTCTTCCTCATCGTGCTCCTCGTCTGCGCCGTCGTCTCCCTCGGC
TTCGGCATCAAGGAGCACGGCCTCAAGGACGGCTGGTATGACGGTGTCAGTATCTTTCTC
GCCGTGTTCCTCGTCGCTGCCGTGTCCGCCGTCAGCAACCACAGCCAGGCCAAGAGGTTC
GACAAGCTGGCCAGTGAGTCCGACAACATCGCCGTCACCGTCGTCCGCGCCGGCCGGAGG
CAGGAGGTCTCCATATTCGAAATCCTCGTCGGCGACGTGGTGATGCTCAAGATCGGCGAC
TCGGTGCCGGCGGACGGGGTGTTCCTGGAGGGTCACGGCCTGCAGGTGGACGAGTCGAGC
ATGACAGGCGAGCCCCACCCCGTCGAGATCGATGCCGAGAAGAACCCCTTCCTCACCGGC
GGCGTGAAGATCATCGACGGCTACGGCCGCATGCTCGTCACCGCCGTCGGCACCGACACC
TTGTGGGGCGAGATGATGAGCAGCATAACCAGGGAGAACACCGAGGCAACGCCGCTCCAG
GAGCGCCTCGAGCGCCTCACCTCAAGCATTGGCAAGATCGGCGTCGCCGTCGCCGTGCTC
GTCTTCACCGTGCTCACCGCGCGCCACTTCACCGGCAGCACCAAGGACGACCAAGGGAAG
CCGCTCTTCAACAAGGACCGCGTTACCTTCGACGCCGTGTTCAGCTCCCTCGTCGTCATT

TTCCAGCAGGCCGTCACCATCATTGTCGTCGCCATCCCCGAGGGTCTCCCGCTCGCGGTC
ACGCTGACGCTCGCCTTCTCCATGAAGAGGATGGTGAAGGAGAACGCGCTGGTGCGCCGC
CTGTCGGCGTGCGAGACAATGGGGTCGGTCACAGCCATCTGCACCGACAAGACAGGAACG
CTGACGCTCAACCAGATGAAGGTGACAGAGTTTTGGGTCGGCACTGACCAGCCCAGAGGC
GCCACGGCGATCGCCGGGAGCGTCGTCAGCTTGCTCTGCCAGGGAGCTGGGCTCAACACC
ACGGGGAGTGTTTACAAGCCGGACAACGTGTCGCCGCCGGAGATAACAGGCAGCCCGACG
GAGAAGGCGCTGCTGTCGTGGGCCGTAGCGGACCTCGGAATGGACGCCGACGCGTTGAAG
AGGAGCTGCAAGGTGCTGCACGTTGAGGCCTTCAACTCGGACAAGAAGCGCAGCGGCGTG
ATGATCAAGAACAACGTTACCGGCGGGGTGGTCGCGCACTGGAAAGGCGCGGCGGAGATG
GTGCTGGCGAGCTGCTCCATGTACGTGGACACGGACGGAGCGGCACGCGAGCTCGGTGTA
GAGCAGAGGAGGAACCTTGAGAAGGTGATCAACGATATGGCAGGCGGCAGCCTCCGGTGC
ATCGCCTTCGCCTACAAGCAAGTCAACGGAACCGAGCAATCAAAGATCGACGACGAGGGT
CTGACATTACTGGGCTTCGTCGGGTTGAAAGACCCGTGCCGGCCAGAGGTCAAGGCCGCC
ATTGAAGCTTGCACCAAGGCAGGCGTCGCCGTCAAGATGGTCACGGGCGACAACATCCTC
ACGGCCCGTGCGATCGCCAAGGAGTGCGGCATCATATCCAGCAACGACCCCAACGGCATC
GTCATCGAGGGGCACGAGTTCCGCGCCATGTCGCCGGAGCAGCAGCTCGAGATCGTGGAC
AGGATCCGCGTCATGGCGCGTTCCCTGCCGTTGGACAAGCTGGCGCTGGTGCAGCGGCTG
AAGCAGAAGGGCCACGTGGTGGCCGTGACCGGCGACGGCACCAACGACGCGCCGGCGCTC
AAGGAGGCCGACGTGGGGCTGTCCATGGGCGTCCAGGGCACCGAGGTGGCGAAGGAGAGC
TCCGACATCATCATCCTCAACGACAACTTCGACACGGTGGTGACGGCCACGCGGTGGGGG
CGCTGCGTCTACAACAACATCCAGAAGTTCATCCAGTTCCAGCTCACCGTGAACGTGGCG
GCCCTGGTCATCAACTTCGTGTCGGCGATCACCACGGGCAAGATGCCGCTCACCACGGTG
CAGCTCCTGTGGGTGAACCTGATCATGGACACCATGGGCGCTCTGGCGCTGGCCACGGAC
ACGCCCACCAAGGCGCTCATGGACCGCCCGCCCATCGGCCGCACGGCGCCGCTCATCAGC
AACGCCATGTGGCGCAACCTCGCCGCGCAGGCGGCGTTCCAGATCGCCGTGCTGCTGGCG
CTCCAGTACCGGGGGCGGGACCTCTTCGGCACCGACGAGAAGGCCAACGGCACCATGATC
TTCAACGCCTTCGTGCTCTGCCAGGTGTTCAACGAGTTCAACGCGCGCGACATCGAGAAG
AAGAACGTGTTCGCCGGGGTGCTCAAGAACAGGATGTTCCTGGCCATCATCGCCATCACG
CTCGTCCTGCAGGTGGTCATGGTGGAGGTCCTCACCAGGTTCGCCGGCACCAAGAGGCTG
GGACTGGGGCAGTGGGGCGTCTGCCTCGCCATCGCCGCCGTGTCGTGGCCCATCGGCTGG
GCCGTCAAGTTCATCCCCGTGCCGGACCGGACCCTCCATGACATCTTGACACGCAGCAAG
TCGTCATGA
>HvACA9
ATGCCGGCCGGGGGCGTCGGCGTGCAGGCGCCCGAGGCGTCGCCGGGGCGCTACGTGCGG
CGGGTCGACGAGGTGCCGCCCGACGACGACGGCTGCGACGACGTCCTCGGGGCCGACGGG
CGGGCCACCGGGGACGGCGACCCCTTCGACATTCCCGCCAAGCGCGCGCCCGTCGAGCGG
CTACGGCGGTGGAGGCAAGCTGCACTTGTGCTCAATGCTTCTCGGCGATTCAGATATACA
CTCGACTTGAAAAAGGAGGAAGAAAAAGAACAAACAAGGAGGAAGATTAGGGCTCATGCT
CAAGTCATACGGGCTGCACTACTGTTCAAGGAAGCAGGAGAAAAGCAGAATGGTGATATG
GAATTACCAGAAATTCTCCCACGAGGGTTTCGAATTGGAGAGGATCAACTTACATCGATG
ACAAGGGATCATAACTATTCTGCTCTGCAAGAATATGGAGGGGTTAAAGGGCTCACAAAT
CTACTGAAAACAAACCCAGAGAAGGGTATCCATGGTGATGAAGCAGATTTGTCATGCAGG
GCGAATGCTTTTGGGGCTAACAGATATCCTCGCAAGAAAGGAAAAAGCTTCTGGGTTTTT
CTCTGGGAGGCCTGCCAGGACTTGACATTGGTTATCCTTATTGTAGCTGCAGCCATCTCT
CTTGTATTGGGCATCGCAACAGAGGGCATCAAGGAAGGATGGTATGATGGTGCAAGTATA
GCATTTGCTGTCTTTCTTGTGATACTTGTTACTGCTGTCAGTGATTACAAACAGTCCCTT
CAGTTCCAACACCTCAATGAGGAGAAACAAAATATTCAAGTCGAGGTCATTAGGGGCGGT
CGAAGAATTCAAGTGTCGATCTTTGATATTGTGGTTGGTGATGTAGTAGCTTTAAAAATT
GGTGATCAGGTTCCATCAGATGGCATTTTAATTAGTGGCCATTCTCTTGCCATTGATGAA

TCCAGTATGACTGGGGAAAGCAAAATTGTTATGAAGGATCAGAAGTCACCTTTTCTAATG
GGAGGATGCAAAGTAGCTGATGGTTACGGTACCATGTTGGTAACTGCGGTTGGTCTAAAC
ACTGAATGGGGTTTATTGATGGCCAGCATTTCTGAAGATAATAACGAAGAGACTCCATTG
CAGGTGCGGTTGAATGGAGTAGCGACATTCATAGGCATTGTGGGGCTTGTTGTTGCAGCA
ATGGTCCTTGTAGTCCTCTTTGCAAGATATTTTACAGGACATACTACAGATCCAGATGGT
ACTGTTCAGTTTGTTAAGGGGCGCACAGGTGTGAAATCTATAATATTTGGAGTAATAAAG
ATACTAACTGTTGCGGTGACTATTGTTGTCGTTGCTGTACCTGAGGGACTACCGCTGGCT
GTAACACTAACCCTGGCTTATTCAATGCGGAAAATGATGGCAGATAAAGCTCTGGTGAGG
AGGCTCTCAGCCTGTGAAACGATGGGTTCTGCTACAACAATTTGTAGTGACAAGACAGGT
ACATTAACACTCAACCAGATGACTGTGGTGCGATCAATAGTTGGGGGGATAGAGCTGCAA
CCTCTGGCTACTATTGAGAAGTTGTCACCTACAGTTACCTCTCTTGTACTTGAAGCAATT
GCACAGAATACTTCAGGCAGTGTGTTTGAGCCAGAGGATGGTAGCACTGTTGAGGTAACA
GGCTCACCAACTGAAAAGGCAATCCTTTCTTGGGGTCTTGAGCTTCATATGAAATTTGCG
GTGGAGCGATCAAAATCTGCTATCATTCATGTCTCTCCATTCAACTCAGAAAAAAAACGC
GGAGGCGTTGCAGTGACTGGGAGAGATTCAGATGTTCATGTGCATTGGAAAGGGGCTGCG
GAAATAGTTCTCGCTTTATGTACAAATTGGCTTGATGTAGACGGCTCAGCTCATGAAATG
ACACCTGATAAGGCTAATCACTTCAGAAACTACATAGAAGATATGGCGGAGCAAAGCCTT
CGCTGTGTTGCTTTTGCTTATAGAGATCTTGATCTGAATGACATTCCAAGTGAGGAACAA
AGAATTAATTGGCAGTTGCCTGATAATGACCTGACTCTTATTGGAATTGCGGGGATGAAG
GATCCTTGCCGTCCTGGAGTGAGAGATGCTGTTGAGCTGTGCACTAATTCTGGTGTTAAG
GTACGGATGGTAACCGGAGATAATCTGCAGACAGCTAGAGCAATAGCACTTGAGTGTGGA
ATACTCACTGACCCCCAGGCTTCTGCACCAGTTATAATAGAGGGAAAAGTTTTCCGTGCA
TACAGTGACGCCGAAAGGGAGGCGGTTGCCGACAAGATATCTGTGATGGGAAGATCCTCT
CCGAATGATAAGCTTCTTCTTGTAAAGGCACTGAAGAAGAATGGTCATGTTGTTGCTGTT
ACTGGAGATGGGACAAACGATGCTCCTGCATTGCATGAGGCAGATATTGGTCTTTCTATG
GGCATCCAAGGAACAGAAGTAGCTAAAGAAAGCTCAGACATAATTATTCTGGATGATAAT
TTTGCTTCGGTCGTGAAGGTGGTCCGCTGGGGGCGTTCTGTTTATGCAAACATCCAAAAG
TTTATTCAGTTCCAGCTTACTGTAAATGTTGCGGCTCTTATAATCAATGTGGTTGCTGCT
ATTTCCTCAGGCAATGTTCCTCTAAATGCTGTCCAGCTTCTCTGGGTTAATCTCATAATG
GACACACTTGGTGCACTTGCATTGGCTACTGAACCACCAACGGACCAGCTTATGAAAAGG
ACACCTGTTGGACGGAGAGAACCTCTTGTGACTAATATTATGTGGAGAAACTTGTTCATT
CAGGCTGTCTATCAAGTGGCTGTTCTTTTGACGCTTAACTTTAGGGGCCGAGATCTTCTG
CATTTGACTAAAGATACCCTCGAACACTCCAGTAAAGTGAAAAATTCATTTATATTCAAC
ACATTTGTCCTGTGTCAGGTGTTTAACGAGTTCAATGCACGTAAACCAGAAGAGCTGAAC
ATATTTGAAGGAGTTTCAAGAAACCACCTCTTTTTGGCCGTCGTGAGCGTAACCGTCGTG
CTGCAGGTGATAATTATCGAGTTCCTTGGGAAATTTACATCAACAGTGAAACTCAGTTGG
CAGCTTTGGCTTGTTTCTCTTGCTATTGCTTTTGTCAGCTGGCCGTTGGCTCTTGTCGGA
AAGTTCATCCCAGTTCCCCAGACCCCATTGAAGAATTTGATCCTGAAGTGTTGGCCGAAA
GGGAAGAATCAAGGTGATGAAGGAGCAGCTCCACCGGTGTGA
>HvACA10
ATGGCGCTCGGGAGCTCGCCGACGCCGCCGGAGATCCGCTCGCCGGAGCGGGACAGGCCG
GAGGGTGCGGCGGGCGCGGAGGGGGAGGAGGAGGAGGAGTTCGGCGACGCCTTCGATATA
CCCCATAAGAACGCATCCCACGACAGCTTGCTCCGATGGAGGCAAGCTGCTCTCGTGCTG
AATGCTTCACGTCGCTTTAGATATACTCTAGACCTTAAAAAGGAGGAAGAGAAAGAGATA
ATAAGGAGAACGATCCGATCACATGCACAAGTGATACGGGCAGTATTTCTTTTCAAAGAA
GCTGGAGAAAATGATCCCAAAGAAGCTTGTACTGGTATAAAACATGCAACGGCCTCTCGC
AGTTTTCCAATTGAACTGGAAAAGCTTAAAACTTTAAACAGAAATCATGATAGTGTTCTT
CTTCAGGAATTTCGAGGAGTTAAGGGGCTGTCAGACTTATTAAAGAGTAATTTAGACAGG
GGAATTAATCCAACTGAGGACGAATTGTTACAAAGAAGGAATGCTTTTGGAGCAAATACA

TATCCACGCAAGAAAAGAAAAAACATACTGCGATTTGTATTTGATGCATGCAAAGATTTA
ACTCTCATCATTCTAATGGTAGCTGCTGCCATATCTCTTACGTTGGGCATGGCCACAGAG
GGTGTCGAGGAAGGATGGTATGAGGGGGGGAGTATTTTTTTAGCTGTTTTTCTTGTGATA
CTTGTCACCGCAACCAGTGATTACAGGCAATCACTTCAGTTTCAACATCTGAATGAGGAG
AAACAGAACATACAAGTGGAGGTTCTTAGAGGCGGTAAAAGATTTCGAGCTTCAATATTT
GACCTTGTTGTTGGTGATGTGGTTCCCCTCAATATTGGTGACCAAGTTCCTGGTGATGGT
ATCCTGATATCTGCCCATTCTCTTGCAATAGACGAATCAAGTATGACTGGGGAATCAAAA
ACTGTTCACAAGGATCAAAAGGCTCCATTCTTGATGTCTGGTTGCAAGGTTGCAGATGGC
TATGGTTCTATGTTGGTAACAGGTGTGGGTATCAACACTGAATGGGGCCAGTTGATGGCT
AACCTTTCTGAAGATAATGGTGAAGAAACCCCATTGCAGGTGCGTTTGAATGGTGTTGCT
ACTTTTATTGGCATGGTGGGCCTGTCAGTAGCTGGTGTTGTACTTGGCGTGCTTGCAATA
AGATATTTTACTGGACATACCAAGAATCCGGATGGGACTGTTCAATTTCGGGCTGGAACT
ACTGGTCTCAAACAAGGATTTATGGGTGCAATCAGAATTTTAACAATCGCCGTAACCATC
GTAGTTGTTGCTGTGCCTGAGGGACTTCCACTGGCAGTAACATTGACACTTGCGTATTCA
ATGCGAAAGATGATGCGAGACAAGGCACTGGTGAGGCGGCTCTCATCTTGTGAAACAATG
GGATCAGCAACTACTATATGCAGCGATAAGACGGGAACTCTCACCTTGAATAAGATGACA
GTTGTGGAGGCACACTTTATCGGGACAAGGTTGGATCCTTGTGATGATGTTAGGGCAATC
TCAAGCAGTTCAGCGGCACTGCTTATTGAAGGAATTGCACAAAACACTACGGGAACTGTA
TTTTTGCCGGAGGATGGGGGAGCAGCCGATGTTACAGGTTCGCCAACTGAAAAAGCCATT
CTTTCTTGGGGCCTTAAGATAGGGATGAATTTCAGTGATGTTCGGTCAAAATCTTCAGTT
CTTCATGTATTTCCGTTTAACTCAGAGAAGAAACGAGGTGGTGTTGCAGTGCAGTCAGAT
ACCGGGGTACACATCCATTGGAAAGGTGCAGCTGAGTTAGTGCTATCATCTTGCAAAAGT
TGGCTTTCTCTGGATGGTTCAGTTCAGCCAATGGGTGCGCAGAAGCGTAATGAGTGCAAG
AAATCAATTGAAGACATGGCGAAGTGTTCACTCCGATGTGTTGCTTTTGCATATTGTCAA
TGTGATATTGAAATAATTCCGAAGGAAAATATAGCTGATTGGAAGTTACCTGATGAAGAC
CTAACTCTGCTTGGTATTGTGGGCATCAAGGATCCATGTCGCCCAGGAGTGAGGAATGCT
GTACAATTATGCAAAAATGCTGGTGTAAAGGTGCGCATGGTCACAGGAGATAATATTGAA
ACGGCCAAGGCAATAGCGTTGGAGTGTGGAATACTAGATGCAAATGGTGCTATTTCAGAA
CCATTTGTAATAGAGGGAAAAGTGTTCCGCGAGATGTCTGAAATTGCAAGAGGAGAGATT
GCTGACAAGATTACTGTCATGGGAAGATCATCTCCAAATGACAAACTTTTGCTTGTCCAA
GCTTTGAAAAGGAAAGGCCATGTTGTGGCTGTCACTGGTGATGGCACAAATGACGCTCCT
GCATTGCATGAGGCTGATATAGGTCTTGCAATGGGCATGTCAGGGACAGAAGTTGCTAAA
GAGAGCTCCGACATTATAATCTTGGATGATGACTTCACATCTGTTGTGAAGGTTGTTCGC
TGGGGACGCTCTGTCTATGCAAATATTCAGAAATTCATCCAGTTCCAGCTTACTGTTAAC
GTTGCCGCACTTGTGATTAATGTTATTGCAGCTGTGTCATCTGGCGACGTGCCTCTAAAT
GCTGTTGAGCTTCTCTGGGTGAACCTTATCATGGATACACTAGGAGCTCTTGCTTTAGCA
ACTGAGCCACCAACAGACAACCTTATGAAGAGACAGCCTGTTGGTCGAAGGCATGAGCCA
CTTGTGACCAATATTATGTGGAGAAACTTGTTTGTCCAGGCCATTTACCAAATAGCAATC
CTTCTTATCTTTAATTTCTCTGGGAAAAAGATTCTACGGCTGCAGAATGAAAGTCCAGAT
AATGCCGAGAAAACGAATAACACATTTATCTTCAATACGTTTGTATTTTGCCAGATCTTC
AACGAGTTCAATGCACGCAAACCCGAGGAAAGAAATGTCTTCGAAGGAGTAACAAAGAAC
CACCTCTTCATTGGAATAATTTGTGTAACTACCGTGTTTCAGATACTAATAGTTGAATTC
CTGGGGAAGTTCTTCAAAATTGTGAGACTTAACTGGAGTCTATGGTTAGTTTCAGTTGCC
ATTGGCGTCGTAAGCTGGCCTTTGGCTTATCTAGGGAAATTCATTCCTGTTCCTGTAAGA
CCTCTCCAGGCCTATTTCAAGCCTTGTTGGAAATCATCCCGCCGAGACGAAGAGGAAGGC
AGGCAGGGTTAA
>HvACA10B
ATGGCGTCGCCGCCGCCGCCGCCGGAGGTCGCCGTGGCGGTTGGCGAGGAGGAGGGGGAG
GGCCGGGAGGCGCAGGGAGAGGATGCGTTCGACATACCCGGCAAGAACGCCCCGCGCGAT

CGCCTGCGGCGGTGGAGGCAAATTGCTCTTGTGCTCAACGCTTCACGCCGTTTTAGATAT
ACTCTAGATCTGGAGAGGGATGAAGAGAGAGAAAACTTGAGAAGAATTATACGAGCTCAT
GCACAAGTTATACGGGCAGTGTTCCTTTTCAAAAAGGCTGGTCAAAAGGAGCTACAAGAA
TCTTACAATGGTACAAAACCGGAGTCACTCTCTCAAAGATTTCCAATTGATCTGGAAAAG
CTTGTAATGTTGAACAGAGATCATGATGCAATTATGCTTCAGGAGGTTGGAGGGGTCAGT
GGGCTTTCAGATTTACTAAAGAGTAATTTAGAGAGAGGAGTTAGCTCAAATGAGGATGAT
CTGTTGCACAGAAGAGACCTTTTCGGAGCAAACACCTATCCGCGCAAGAAAAGGAAAGGA
ATATGGCGCTTTGTATTTGAAGCTTGTTGGGATTTAACCCTTGTGATTCTAATGGTAGCT
GCTGCTATATCATTATCACTGGGCATTGCAACAGAGGGTGTAAAAGATGGATGGTATGAT
GGTGGAAGCATATTCTTTGCTGTCTTTCTTGTGATATTTGTTACAGCAACCAGTGATTAT
AGGCAATCTCTTCAGTTTCAACATCTGAACGAGGAGAAACAAAACATAAAAGTTGAGGTT
ATCAGAGGTGGTAAGAGAGTAGGAGTTTCAATATTTGACCTTGTGGTCGGCGATGTTGTT
CCCCTCAAAATTGGCGACCAAGTCCCTGCAGATGGTGTCCTGATATGTGGTCATTCTCTT
GCAATAGATGAATCAAGTATGACGGGAGAGTCCAAAATTGTTCATAAGGACCAGAAGGCA
CCTATGTTGATGTCCGGTTGCAAGGTCGCAGATGGCTATGGCTCTATGTTGGTAACAGGC
GTGGGTACTAATACTGAATGGGGTATGTTGATGGCCAATCTTTCAGAAGATATTGGTGAA
GAAACTCCGTTGCAGGTGCGCTTGAATGGCGTCGCCACTTTAATTGGTATCGTGGGTTTA
TCTGTTGCTGGTGTTGTCCTTGTCGTACTTTGGATAAGATATTTTACCGGGCATAGCAGT
AATCCAGATGGAACTACTGCATTTGTGGCTGGGACTACTGGTGCAAAACAGGGATTTATG
GGGGCAATCAGTATTTTTACAATTGCTGTAACTATTGTGGTTGTTGCTGTTCCTGAAGGA
CTCCCTTTAGCAGTAACATTGACCCTTGCATATTCAATGCGAAAGATGATGCGAGACAAG
GCTCTGGTGAGACGACTTTCATCTTGTGAAACAATGGGGTCGGCGACCACAATTTGCAGT
GACAAGACTGGAACTCTTACCTTGAATAAGATGACAGTCGTGGAAGCATATTTGAGTGGG
ACGAAGTTGAATCCTTGTAATAATACCGGGATGATGTCTAGCAGTGCGGCATCTCTACTT
GTTGAAGGAATTGCACAAAACACGGCAGGGGCTGTGTTTTCACCAGAGGATGGAGGAACT
GCTGAAATTGCGGGTTCGCCAACTGAAAAAGCAATTCTTTCTTGGGGTCTTAAGATTGGG
ATGAATTTCAACGATGTGAGATCAAAATCTTCAGTTCTCCATGTTCTCCCATTTAACTCA
ATGAAGAAATGTGGTGGCGTTGCAGTGCAAGTGTCGGATGCTTATGCACACATCCACTGG
AAAGGTGCTGCTGAGATAGTATTAGCATCTTGCAAAAGCTTGCTTTCTATTGATGGTTCA
GTTCATCCGATGAGTTCTGACAAGTATAATGAATTGAAGAGATCCATTGACGATATGGCA
ATGAGTTCACTGCGCTGTATTGCTTTTGCATATTGCACCTGCGAGCTCACAATGGTTCCT
AGGGAGGATCTCGATAAGTGGCAGTTGCCTGAGGATAATCTGACTCTTCTTGGAATGGTC
GGGATAAAGGATCCCTGTCGCCCAGGAGTAAGGGATGCTGTACAATTATGCAGTGCTGCT
GGTGTGAAGGTACGGATGGTCACAGGAGATAATGTTGAAACAGCTAAGGCCATTGCTCTC
GAATGTGGAATACTAAATGCAAAAGATGTTGCTTCAGAGACAATAATAATAGAGGGGAAG
GTGTTCCGTGAAATGTCTGAAACTGCACGAGAAGAAGTTGCTGACAAGATTACAGTAATG
GGACGGTCTTCTCCAAACGACAAACTTTTGCTTGTACAAGTTTTGAAAAGGAAAGGTCAT
GTAGTAGCTGTAACCGGTGATGGCACCAATGACGCCCCAGCATTACATGAGGCTGATATT
GGTCTTTCAATGGGCATCTCGGGAACAGAAGTTGCTAAAGAAAGCTCGGACATTATAATC
TTGGATGATGATTTCACATCCGTTGTCAAGGTTGTTCGTTGGGGACGGTCTGTCTATGCA
AATATTCAGAAATTTATCCAGTTCCAGCTGACTGTTAATGTCGCTGCCCTGGTAATAAAT
GTGGTTGCTGCTGTGTCCTCTGGTGCTATTCCTCTGAATGCAGTTGAGCTTCTTTGGGTG
AACCTTATCATGGACACACTAGGAGCCCTTGCATTAGCAACTGAACCACCAACAGACAAC
CTAATGAAGAGACATCCTGTTGGCAGAAGGGAACCTCTTGTTACAAATGTCATGTGGAGA
AACCTGTTTATCCAGGCTCTTTACCAGATAGCAGTTCTTCTCATCTTCAATTTTGACGGC
AAAAGGATTTTCCATTTGCATAATGAAAGTCGAGAGCACGCTGACAAAATTAAGAACACC
TTTGTCTTCAATGCATTTGTCTTCTGCCAAATCTTCAATGAGTTCAATGCTCGCAAGCCT
GAGGAGAAGAATGTCTTCCTAGGAGTTACAAGCAACCGCCTTTTCATGGGTATAGTGGGT
ATAACTACCATTCTTCAGATCTTGATAATTGAATTTCTCGGGAAGTTCTTCGGAACTGTT

AGGCTCGGTTGGAAGCTATGGGTGCTATCAGTTGCCATTGGTGCAGTAAGCTGGCCTCTC
GCGTATGTTGGCAAGTCCATTCCTGTCCCCGCCACACCTTTCCAGGATTACTTCAAGCAT
TGTTCTGTCTGGAGAAGGCCATGCCGCCGCGGCGACGAAGAGCAGGGCAGCAAGAGCTAA
>HvACA11
ATGGAGTCGTCCTCGTCGGGCGGCGCCAGGGCGCGGCGGAGCGGCAGCTGGGGCAGCGTC
GGCGGCAGCGGTGGAGGAAGCGACCCCTTCGACATCCCCGGCAAGGGCGCCCCCGTCGAG
CGCCTCAAAAAGTGGAGGCAAGCAGCTCTTGTCCTGAATGCATCAAGGCGATTCAGATAC
ACATTGGACTTGAAAAAAGAGGCGCAAAAGGAGGAAGTCATAAGAAAAATTCGTGCACAA
GCTCATGTTATCAGGGCTGCATTCCGCTTCAAAGAAGCAGCTCGACTTGGTTCTCAGACA
AAGGACGTACCAGAAGCACATGCGGATGGTGCACTTGGCTTTGGAATTAAGGAAGATCAG
CTGACGGCACTGACCAGAGATCATAACTACTCTGCTCTACAGCAGTATGAAGGGATTTCA
GGGCTAGCAAATATGCTAAAGACAGACCCTGACAAGGGGATCAGTGGAGATGACTCTGAT
ATTGATGCGAGAAAAAATGCATTCGGGTCAAACACGTACCCTCGTAAGAAAGGGAGAAGC
TATATGGCCTTCGTGTGGGATGCATGTAAAGATTTGACACTCATCATCCTCATGGTCGCC
GCTGCGGTTTCATTGGCCTTGGGCATATATACAGAGGGGATAAAAGAAGGTTGGTATGAT
GGGGCGAGCATTGCCTTTGCTGTACTCCTTGTTATTTTTGTTACAGCTACCAGTGACTAC
AAACAATCGTTGCAGTTCCAAAACTTGAATGAGGAGAAACAGAACATTCAATTGGAGGTT
GTGAGAGGTGGAAGGCGAATCAAGGTATCAATTTATGATTTGGTTATTGGAGATGTAGTG
CCCCTCAAGATTGGTGACCAGGTACCTGCTGATGGAGTCCTTATTAGTGGTCACTCCTTC
TCCATAGATGAATCAAGCATGACAGGCGAAAGCAAAATAGTAAACAAAGACCAGAAGTCA
CCTTTTCTAATGTCGGGTTGCAAAGTCGCCGATGGCTATGGTACAATGCTGGTGACCGCT
GTTGGTATTAACACTGAATGGGGGCTACTCATGGCAAGCATATCTGAAGACTCTGGTGAA
GAAACACCCTTACAGGTTCGCTTGAATGGTGTTGCTACCTTCATTGGAATAATTGGACTT
TCTGTTGCTGTCGCTGTTCTCGTTGTCCTCTTGGCCAGGTATTTCACTGGGCATACGTAC
AATCCGGATGGCTCTCCACAATACGTGAAAGGAAAGATGGGTGTCGGCGAGACAATACGT
GGAGTAGTTAAAATCTTCACAGTGGCGGTCACAATTGTGGTTGTGGCTGTTCCCGAAGGA
CTACCATTGGCTGTCACATTGACGCTCGCCTTTTCGATGCGCAAAATGATGAGGGATAAA
GCTCTGGTCAGGAGACTTTCTGCATGTGAGACAATGGGTTCCGCAACAACAATTTGCAGT
GACAAGACTGGAACATTAACTTTAAATCAGATGACTGTTGTTGAGGCATACTTTGGTGGA
GAGAAGATGGATCCTCCAGATAACACTCAGAAGCTATCTGCTCCCGTGTCAACAATGATT
ATTGAAGGAATTGCTCAGAATACATCCGGAAGCATATTTGAGCCTGAGGGTGGTCAAGCT
CCAGAGGTTACTGGATCACCAACTGAAAAGGCTATACTCTCTTGGGGGTTGCAGCTTGGT
ATGAAATTCAGTGAAACAAGATCGAAATCCTCCATTCTTCAAGTCTTCCCATTCAATTCA
GAGAAAAAGCGAGGCGGGGTTGCTGTGCAAGTGGGTGACTCTGAGGTTCATGTATACTGG
AAAGGAGCTGCTGAACTTATTTTGGAATCATGCACTAGTTGGGTTGACATGGATGGTTCA
AATCACTCAATGACTCCTGAGAAAGCTGCTGAGTTCAAGAAATTCATTGAGGACATGGCT
GTTGCTAGCCTTCGCTGTGTCGCCTTTGCATACAGACCTTGTGAGATGAGTGATGTTCCG
AAGGAGGACCAGAGGGCTGACTGGGTATTACCCGAAGATAACCTGATTATGCTTGGTATT
GTGGGAATAAAGGATCCCTGCCGCCCAGGAGTTCAAGATTCTATCCGCTTGTGTACGGCA
GCTGGCATTAAGGTCCGCATGGTCACTGGGGATAATCTTCAAACTGCCAGAGCAATTGCC
TTGGAATGTGGTATTCTTACTGATCCTAACGTATCAGAGCCAACCATCATTGAAGGGAAG
ACGTTTCGGGAGTTGACAGATTTGGAAAGGGAGGAAGTTGCCGACAAAATTTCTGTTATG
GGGAGGTCTTCCCCGAATGATAAACTTTTACTTGTTAAGGCACTCAGGAGCAGAGGTCAT
GTTGTTGCTGTAACTGGTGATGGCACAAACGACGCGCCGGCACTGCATGAGGCCGACATT
GGTCTCTCGATGGGCATCCAGGGAACTGAAGTTGCTAAGGAGAGTTCTGATATTATTATC
TTGGATGACAATTTTGCTACACTCGTTAGGGTTGTAAGATGGGGACGTTCAGTTTATGCA
AACATTCAGAAATTCATTCAGTTCCAGCTGACCGTCAACGTTGCAGCCTTGATAATTAAT
GTAGTTTCTGCTGTCAGTTCTGGTGACGTGCCACTGAATGCTGTCCAGTTGCTTTGGGTT
AATCTAATCATGGACACTCTGGGAGCGCTTGCATTGGCGACTGAGCCACCTAACAACCAT

CTTATGGAGAGAGCACCGGTTGGACGGAGGGAGCCTTTGATAACAAATATCATGTGGAGA
AACTTGCTCATAATGGCTTTCTATCAAGTTGCAATCCTCCTTACTCTCAACTTCAAGGGC
CTGAGCCTTCTACGGTTGGAACATGACAACCCAGCGCATGCTGAAATGCTGAAAAATACC
TTCATATTCAACACATTTGTTCTCTGTCAAGTGTTCAGTGAGTTCAACGCTCGGAAACCA
GATGAGCTGAATATTTTCAAGGGTATTGCAGGGAACCGCCTCTTCATTGCCATTATAGCC
ATAACGGTTGTGCTCCAGGTGCTTATCATCGAGTTTCTTGGTAAGTTCACGACAACAGTC
AGATTGAGCTGGCAGCTGTGGTTGGTGTCCATAGGTCTTGCTTTTATCAGCTGGCCGTTA
GCACTCGTGGGAAAGCTTATCCCTGTTGCAGACCGTCCGTTGTTAGATATGTTCTCTTGC
TGCTGCCCAGCCAAGAAAGAGGCTGGTGATGCGAAGGAAGACGACGGTGTGAAGCACATC
GAGGTGGTGTGA
