# Supplementary material for: A two-sequence motif-based method for the inventory of gene families in fragmented and poorly annotated genome sequences
Source: BMC Genomics. 2024 Jan 3;25:26. doi: 10.1186/s12864-023-09859-4 (PMC10763278; doi:10.1186/s12864-023-09859-4)
Supplement: Supplementary file 4 — Additional file 4: Supplementary file 4. Hordeum vulgare P3A ATPase coding sequences. [file 12864_2023_9859_MOESM4_ESM.pdf]

Supplementary File 4 – *Hordeum vulgare* P3A ATPase coding sequences

>4820.8_HvAHA1
ATGGCGGCCATGGCGAGCAGGCAGCAGGAGGGCAGCCTCGACGCCGTCCTCAAGGAGGCC
GTCGACCTGGAGCACATCCCGATCGACGAAGTGTTCGAGAACCTGCGGTGTAGCCATGAG
GGGCTTACTTCCGAGCAGGCGCAGCAGCGGCTGCAGATCTTCGGCCCGAACAAGCTAGAG
GAGAAGGAGGAGAGCAAGCTCCTCAAATTTCTAGGGTTCATGTGGAACCCCCTCTCATGG
GTCATGGAGGCTGCGGCGATCATGGCCATCGCACTGGCCAACGGAGGGGGGAAGCCACCA
GATTGGCAAGACTTCGTCGGTATCATCACGCTGCTGCTTATAAACTCCACCATCAGTTTC
ATCGAGGAGAACAATGCCGGAAATGCCGCCGCCGCGCTTATGGCCCGTCTTGCACCAAAA
GCCAAGGTCCTCCGTGACGGTCGTTGGACCGAGGAGGAGGCCGCCGTCCTTGTGCCCGGG
GACATCATCAGCATCAAACTTGGAGATATCATCCCTGCCGACGCCCGCCTCCTGGATGGT
GATCCTTTGAGGATTGATCAGTCTGCCCTGACCGGAGAATCGCTGCCAGCCACCAAAGGT
CCTGGTGACGGCGTCTACTCCGGTTCGACGGTCAAGCAGGGCGAGATCGAGGCCGTTGTC
ATAGCAACTGGCGTGCACACTTTCTTTGGAAAGGCCGCACATCTCGTCGACTCCACCAAC
CAAGTTGGCCATTTCCAACAGGTGTTGACGGCCATTGGGAACTTTTGCATTTGCTCGATT
GCTGTGGGAATGTTCATCGAGATCATTGTCATGTATCCTATCCAGCACAGGGCGTACCGC
CCCGGGATCGACAACCTTTTGGTGCTTCTCATTGGAGGCATTCCCATAGCGATGCCCACA
GTCTTGTCGGTCACCATGGCGATTGGGTCTCATCGCTTGTCTCAGCAGGGAGCTATAACA
AAGAGGATGACTGCAATTGAAGAGATGGCCGGCATGGATGTTCTTTGCAGTGATAAGACT
GGAACCCTGACTCTAAATAAGCTCAGTGTGGACAAGAACCTAGTCGAGGTTTTTGAAAAA
GGAGTGACTCAGGACCAGGTGATTCTAATGGCTGCTAGAGCATCCCGGATAGAAAATCAA
GACGCCATCGATACGGCAATAGTTGGGATGCTAGGCGATCCAAAAGAGGCGCGTGCCGGT
ATTCAAGAGGTTCATTTTCTGCCATTCAACCCTACCGACAAAAGAACTGCGTTGACATAC
ATCGATGGCGATGGAAAGATGTACCGTGTTAGCAAAGGTGCACCAGAGCAGATTCTCAAC
CTGGCCTACAACAAGTCAGAGATCGCACAAAAAGTCCACACTGTCATCGACAAGTTCGCG
GAACGTGGACTTCGGTCACTTGGTGTAGCATATCAGGACGTGCCAGATGGGAGGAAAGAG
AGCCCCGGTAGCCCGTGGCATTTTGTTGCTCTCTTGCCACTCTTTGATCCACCGAGGCAC
GACAGCGCGGAAACAATTGAAAGGGCACTTAACCTTGGTGTGAATGTGAAGATGATCACA
GGCGACCAGCTAGCTATTGGGAAGGAAACAGGGCGTCGTCTAGGAATGGGTACAAACATG
TACCCTTCATCTGCTTTGCTGGGGCAGAACAAGGATGAGTCTATTGCTGATTTACCGGTC
GATGATCTAATTGAGAAAGCCGATGGTTTTGCTGGCGTATTCCCAGAGCACAAATATGAG
ATTGTGAAGCGCCTACAAGCACGGAAGCACATTTGTGGAATGACCGGCGATGGCGTAAAC
GATGCACCAGCCCTAAAGAAAGCTGATATTGGCATAGCGGTTGCCGATGCGACAGATGCA
GCGAGGAGCGCTTCTGATATCGTACTCACGGAACCTGGTCTAAGTGTGATCATTAGTGCC
GTCCTTACCAGTCGAGCAATTTTCCAGCGGATGAAGAACTACACTATCTATGCGGTCTCG
ATTACGATACGTATTGTGCTTGGATTTATGCTACTTGCGCTCATATGGGAGTTTGATTTC
CCGCCATTTATGGTCCTGATCATAGCAATTCTGAATGATGGCACCATAATGACAATATCC
AAGGATCGAGTAAAGCCTTCTCCACTACCTGACAGCTGGAAGTTGGCTGAAATTTTCACA
ACTGGGGTGGTGCTTGGCGGATACTTGGCAATGATGACTGTCATTTTCTTCTGGGCCGCA
TACAAGACTAACTTTTTCCCGAGGGTTTTTCATGTGAGAAGCCTTGAGAAGACAGCTCAA
GATGACTTCAACAAAATGCTTGCCTCTGCTGTATACCTTCAAGTCAGCACCATCAGCCAA
GCTCTCATCTTCGTTACAAGGTCTCGAAGCTGGTCGTTCCTCGAGCGCCCCGGCTTTCTC
CTGGTCTTCGCTTTCTTTGTCGCGCAGCTGATAGCTACACTGATCGCCGTATACGCCGAT
TGGGCATTCACTTCGATCAAGGGCATCGGGTGGGGCTGGGCCGGTATCGTGTGGCTCTAC
AACCTCGTCTTCTACTTCCCACTCGACATCATCAAGTTCTTCATCCGATACGCTCTGAGC
GGGAAAGCATGGGATCTTGTCATCAACCAAAGAATTGCGTTTACAAGGAAGAAGCACTTT
GGTAAGGAAGAGAGGGAGCTCAAGTGGGCCCATGCACAGAGGACACTCCATGGGCTGCAG
CCGCCGGATGCCAAGCTGTTCCCCGAGAAGGCGGGCTACAACGAGCTGAATCAGATGGCC

GAGGAGGCGAAGCGGAGGGCTGAGATTGCAAGGCTCAGGGAGCTTCACACTCTCAAGGGG
CATGTGGAGTCGGTTGTGAAGCTCAAGGGCCTCGACATCGACACCATTCAGCAATCTTAC
ACCGTGTGA
>0780.2__HvAHA3
ATGGCCGACAAGGAGGCCGGCAACCTGGAGGCCGTCCTCAAGGAGGTCGTCGACCTGGAG
AACATCCCCCTGGAGGAGGTGTTGGACAACCTGCGGTGCAGCCGGGAGGGGCTCACGGCG
GAGCAGGCGCAGCAGCGCCTCCAAATCTTCGGCCCGAACAAGCTGGAGGAGAAGGAGGAG
AGCAAGTTCCTCAAGTTCCTCGGCTTCATGTGGAACCCGCTCTCCTGGGTCATGGAGGCC
GCCGCCATCATGGCCATCGCCCTCGCCAACGGAGGGGGGAAGCCGCCGGACTGGCAGGAC
TTTGTGGGCATCATCACCCTGCTCGTCATCAACTCCACCATCAGTTTCATCGAGGAGAAC
AATGCAGGCAACGCCGCCGCCGCGCTCATGGCCCGTCTCGCGCCCAAAGCCAAGATTCTT
CGCGACGGCCGGTGGGCCGAGGAAGACGCGGCCATCCTCGTGCCAGGGGACGTCATCAGC
ATCAAGCTCGGGGACATCATACCTGCAGACGCACGCCTCCTCGAGGGAGACCCTCTCAAG
ATTGATCAGTCTGCCCTGACCGGAGAATCATTGCCGGCCACCAAAGGCCCGGGCGACGGC
ATCTACTCCGGATCGACGGTCAAGCAAGGCGAGATCGAGGCCGTCGTGATCGCGACCGGC
GTTCACACGTTCTTCGGAAAGGCTGCACACCTTGTTGACTCCACTAACCAAGTGGGCCAT
TTTCAGAAGGTTCTGACGGCCATTGGGAACTTCTGCATTTGTTCGATCGCTGTGGGGATG
TTCATCGAGATCATTGTAATGTATCCTATCCAGCACAGGGCGTACCGCCCCGGAATCGAC
AACCTCTTGGTCCTTCTCATTGGAGGCATTCCCATAGCCATGCCAACTGTCTTATCCGTG
ACTATGGCTATCGGGTCACATCGCTTGTCCCAACAGGGAGCTATCACAAAGAGAATGACT
GCAATCGAGGAGATGGCGGGCATGGATGTCCTTTGCAGTGATAAAACTGGAACTTTGACC
CTCAATAAGCTTACCGTGGACAAGAGCCTTGTTGAGGTTTTCGAAAGAGGGATCACCCAG
GACCAGGTGATTCTCATGGCTGCTAGAGCGTCTCGAACAGAAAACCAAGATGCTATTGAT
ACAGCAATTGTTGGGATGCTCGCTGATCCGAAAGAGGCACGTGCTGGTATTCAAGAAGTT
CATTTTCTGCCATTCAATCCTACTGACAAGAGAACTGCGCTGACATACATTGACGCTGAT
GGCAAAATGCACCGCGTTAGTAAGGGTGCACCTGAGCAGATTCTTCACCTCGCTCACAAT
ACATCGGAGATAGAGCGGAGGGTCCATGCTGTGATTGACAAATTTGCAGAGCGTGGACTT
CGATCGCTTGCTGTAGCGTATCAGGAAGTACCAGATGGGAGGAAGGAAAGCCCTGGTGGC
CCATGGCACTTTGCCGGTCTGATGCCACTTTTTGATCCTCCAAGGCATGACAGTGCTGAA
ACAATCCGGAGGGCACTTAACCTTGGTGTTAATGTCAAGATGATCACAGGTGATCAGCTC
GCCATTGGAAAGGAAACAGGGCGTCGCCTGGGGATGGGTACAAACATGTATCCTTCATCC
GCTCTGTTGGGGCAGAAAAATTCAGACGAGTCCATTTCTGCTTTACCAGTTGACGATCTC
ATTGAGAAAGCTGATGGTTTTGCCGGCGTCTTCCCTGAGCACAAGTATGAGATTGTGAAA
CGCCTGCAAGCACGAAAGCACATCTGTGGAATGACGGGTGACGGAGTAAATGATGCTCCA
GCTCTTAAGAAAGCGGATATTGGTATTGCCGTTGCTGATGCAACTGATGCAGCCAGGAGC
GCTTCGGATATTGTCCTCACAGAACCAGGCCTCAGCGTGATCATCAGTGCTGTGCTCACC
AGCCGTGCAATTTTCCAGCGTATGAAGAACTACACTATCTATGCTGTCTCCATCACAATT
CGTATTGTGCTTGGGTTTATGCTACTTGCCCTCATCTGGAATTTCGACTTTCCACCGTTC
ATGGTCCTGATCATAGCGATTCTAAATGATGGTACCATCATGACTATATCAAAGGATCGG
GTAAAACCATCTCCACTACCTGACAGCTGGAAGTTGGCTGAGATTTTTACAACTGGAGTT
ATCCTTGGTGGTTACTTGGCAATAATGACGGTCATCTTCTTCTGGGCTGCGTACAAGACA
AACTTTTTCCCGAGGCTGTTTCACGTCGAAAGCCTCGAGAAGACAGCTCAGGACGATTTC
CAAAAGCTCGCGTCTGCCATTTACCTCCAAGTCAGCACCATCAGCCAAGCTCTGATCTTC
GTCACCAGGTCCCGCAGCTGGTCATTTGCCGAGCGCCCTGGGTTTCTACTCGTCTTTGCA
TTCTTCGTTGCGCAGCTGATTGCTACACTGATTGCGGTGTATGCTGACTGGAAGTTCGCT
GCGATCAAAGGCATCGGGTGGGGCTGGGCTGGCGTCGTGTGGCTCTACAACATCATCACA
TACTTCCCGCTCGATATCATCAAGTTCCTCATCCGATACACTCTGAGCGGCAAAGCATGG
GACCTCGTCATCGATCAAAGGATCGCGTTCACAAGGAAGAAAGACTTCGGTAAGGAGGAG
AGGGAGCTCAAGTGGGCGCACGCTCAGAGGACCCTCCATGGGCTGCAGCCGCCGGACGCC

AAGATGTTCTCGGACAAGGGGGGCTACAATGAGCTGAACCACATGGCTGAAGAGGCCAAG
AGGAGGGCCGAGATCGCAAGGTTGAGGGAGCTTCATACGCTCAAAGGGCACGTGGAGTCC
GTCGTCAAGCTCAAGGGTCTCGACATCGAGACCATCCAGCAGTCCTACACCGTCTGA
>4340.1_HvAHA4
ATGGCCGCCTCCCTCGAAGACCTCCGCAACGAGAGCATCGATCTCGAGGCGATCCCGATC
GCGGAGGTGTTCCAGGTGCTCAAGTGCAAGCCGCACGGGCTCACCTCCGATGAGGCCGCC
AGCCGCCTCCAGGCATTCGGCCCCAACAAACTCGAGGAGAAGAAGGAGAGCAAGTTCCTC
AAGTTTTTAGGGTTTATGTGGAACCCTCTGTCGTGGGTCATGGAGGCGGCGGCGATCATG
GCCATCGTCCTCGCCAACGGCGGCGGCAAGCCCCCCGACTGGCAGGACTTCATGGGCATC
GTCACCCTGCTCATCATCAACTCCACCATCAGCTTCATCGAGGAGAACAACGCCGGCAAC
GCGGCGGCGGCGCTGATGGCCAGCCTGGCGCCGCAGACCAAGGTGCTCCGGGACGGCAAG
TGGGCGGAGCAGGACGCGGCGATCCTGGTGCCGGGGGACATCATCAGCATCAAGCTGGGC
GACATCATCCCGGCGGACGCGCGGCTGATGGAGGGCGACCCGCTCAAGATCGACCAGTCG
GCGCTCACCGGCGAGTCCCTCCCCGTCAACAAGCTCCCCGGCGACAGTGTGTACTCCGGC
TCCACGTGCAAGCAGGGCGAGATAGAGGCCGTCGTCATCGCCACCGGCGTCCACACCTTC
TTCGGCAAGGCCGCGCATCTCGTCGACAGCACCAACAACGTCGGCCACTTCCAAAAGGTT
CTGACTGCAATCGGCAACTTCTGCATCGTGTCCATCGCCATCGGCATGCTGGTGGAGATC
GTCGTCATGTACCCGATCCAGCACCGCCGGTACCGCGACGGCATCGACAACCTCCTCGTG
CTCCTCATCGGCGGCATCCCCATCGCCATGCCCACCGTGCTCTCCGTCACCATGGCCATC
GGCTCCCACAAGCTGTCGCAGCAGGGCGCCATCACCAAGCGGATGACGGCCATCGAGGAA
ATGGCCGGCATGGACGTGCTCTGCAGCGACAAGACGGGCACACTCACGCTCAACAAGCTC
ACCGTCGACAAGAACATGATCGAGCCCTTCGTCAAGGACGTCGACAAGGACGGCGTGGTT
CTCTATGCCGCCCGAGCGTCGAGGACGGAGAACCAGGACGCCATCGACGCATCCATTGTC
GGCATGCTCGCCGACCCCAAGGAGGCCCGAGCTGGCATTCAGGAGGTGCACTTCATGCCA
TTCAACCCCGTCGACAAGCGTACAGCCATCACCTACGTTGACTCCGACGGAACGTGGCAC
CGGGTTAGCAAGGGCGCCCCGGAGCAGATCATCGATCTGTGCGGGCTGCGCGAGGACGTG
CGGCGGCGGGTGCACGGCATCATCGGCAAGTTCGCCGACCGCGGGCTGCGGTCGCTGGCG
GTGGCTCGGCAGAGCGTGCCAGAGCGGACCAAGGAGGCCAAGGGCGCACCGTGGCAGTTC
CTCGCAGTGCTGCCGCTGTTTGACCCGCCGCGGCACGACAGCGCAGAGACCATCCGCCGC
GCGCTGCACCTGGGCGTGAACGTGAAGATGATCACCGGCGACCAGCTGGCCATCGGCAAG
GAGACGGGCCGCCGGCTGGGCATGGGCACCAACATGTACCCGTCAAGCTCCCTCCTCAAG
GATGGCGACAGCTGCGGGCTGCCCGTCGACGAGCTGATCGAGAAGGCGGACGGGTTCGCC
GGCGTGTTCCCGGAGCACAAGTACGAGATCGTGCGGCGGCTGCAGGAGATGAAGCACATC
TGCGGGATGACGGGCGACGGCGTGAACGACGCGCCGGCGCTCAAGAAGGCGGACATCGGC
ATCGCCGTGGCCGACGCCACGGACGCGGCCCGGAGCGCGTCGGACATCGTGCTGACGGAG
CCCGGGCTGAGCGTGATCATCAGCGCCGTGCTCACCAGCCGCGCCATATTCCAGCGGATG
AAGAACTACACCATCTACGCCGTGTCCATCACCATCCGGGTAGTCCTTGGCTTCCTCCTG
CTGGCGCTCATCTGGAAGTTCGACTTCGCGCCCTTCATGGTGCTCATCATCGCCATCCTC
AACGACGGCACCATCATGACCATCTCCAAGGACCGCGTCGTGCCGTCGCCCACCCCCGAC
TCGTGGCGCCTCAAGGAGATCTTCGCCACCGGCATTGTCCTCGGCACCTACCAGGCCAGC
GCCACCGTGATCTTCTTCTGGGCCGTCCACTCCACCGACTTCTTCACGAATAAACTCCAC
GTGCACCCCATCGGCGGCAACACGGAGGAGCTGATGGCGGCGGTGTACCTGCAGGTGAGC
ATCATCAGCCAGGCGCTCATCTTCGTGACGCGGTCGCGGGGGTGGTCGTTCAGGGAGCGG
CCGGGGGCGCTGCTCTTGGGCGCGTTCCTGCTGGCGCAGATGGTGGCGACGCTGATCGCG
GTGTACGCCGACTGGCCGTTCGCCAAGATGAAGGGGGTAGGGTGGGGGTGGGCGGGCGCC
ATCTGGCTCTTCACCATCGTCACCTACTTCCCGCTCGACGTGCTCAAGTTCGCCATCCGC
TACTTCCTCTCCGGCAGGGGGTGGAGCAACGTGTTCGACGGCAAGACGGCCTTCGCGCAG
GGGGTGGACTACGGCACGGACAAGCGCAAGGCGGAGTGGGCCGTGGCGCAGAGGTCGCTC
CACGGCCTGCACACGTCCGGCGGCGGCGAGGCTTCCTCCTCCGGCGTGCTCGGTGGCGGG

GACGACAAGAACGACATCTCGGAGATCGCCGAGCAGGCCAAGCGGCGCGCCGAGATCGCA
AGGCTGCGGGAGCTGCACACGCTCAAGGGGCACGTCGACTCGGTGGTGAAGCTCAAGGGG
CTCGACATCGAGAACATCAACCACAACTACACCGTCTGA
>3560.6_HvAHA5
ATGGCGGCGGCGGCGGCCGAAGGTCTGGAGCGAATCAAGAACGAGTCCGTCGACTTGGAG
AACATCCCTGTGGAGGAGGTGTTGGAGAATCTCCAGTGCAGCAAGGAAGGCCTCACCTCC
AAGGATGGCCAGGACCGCATGGCCGTCTTCGGCCCCAACAAACTCGAAGAGAAAAAGGAG
AGCGAGATCCTAAAATTCCTGGGGTTCATGTGGAACCCGCTGTCGTGGGTGATGGAGGTG
GCGGCCATAATGGCCATCGCGCTGGCCAACGGCGGCGGCAGGCCGCCGGACTGGCAGGAC
TTCGTCGGCATCATCGCGCTGCTCCTGCTCAACTCCACCATCTCTTACATCGAGGAGAGC
AACGCTGGCAGCTCGGCCAAGGCACTCATGGCCAACCTCGCCCCCAAGACAAAGGTGCTC
CGCGACGGAAAGTGGAGCGAGCAGGACGCCTCCATCCTCGTGCCCGGCGACATTATTAGC
ATCAAGCTCGGCGACATAGTCCCGGCTGATGCGCGCCTCCTGCTCGAGGGCGACCCGCTC
AAGATCGACCAGTCGGCGCTCACGGGCGAGTCGCTGCCCGTCACCAAGAACCCCGGCGAC
AGCGTCTACTCGGGGTCCACGTGCAAGCAGGGCGAGATCGAGGCCGTCGTCATCGCCACC
GGCGTGCACACCTTCTTCGGCAAGGCGGCGCACCTCGTCGACAGCACCAACCAGGTCGGC
CACTTCCAGAAGGTGCTCCGCGCCATCGGCAACTTCTGCATCGGCGCCATCGCCATTGGC
ATGATCGTCGAGGTCATCGTCATGTACTTCATCCAGCACCGCCGCTACCGCGACGGCATC
GACAACCTCCTGGTGCTCCTCATCGGTGGTATCCCCATCGCCATGCCCACGGTGCTGTCG
GTCACCATGGCTATCGGCTCCCACCGGCTGTCCAAGCAGGGAGCCATCACCAAGCGCATG
ACGGCCATCGAGGAGATGGCCGGCATGGACGTGCTCTGCAGCGACAAGACGGGCACGCTC
ACCCTCAACAAGCTCAGCGTCGACCGCAACCTCATCGAGGTGTTCGCCGGCGGCGTCGCC
AAGGACGATGTCTTGCTTTTCGCCGCCATGGCATCCAGGGTGGAGAACCAGGATGCTATC
GACGCTGCCATGGTCGGCATGCTCGCCGACCCAAAGGAGGCCAGGGCTGGCATCCAGGAG
ATGCACTTCCTCCCCTTCAACCCCGTCGACAAGCGCACAGCGCTCACCTACCAAGACCTC
GCCGATGGGACATGGCACCGCGTCAGCAAGGGTGCTCCCGAGCAGATCTTGGAACTGTGC
AACTGCAGAGAGGATGTCAAGAACAAGGCGCACGCCATCATCGACAAGTACGCCGAGCGC
GGCCTCCGGTCCCTCGCGGTAGCCAGGCAGGAGGTGCCGGAGAGGAGCAAGGACAGCTCC
GGAGGGCCATGGGAGTTCATCGGTCTTCTGCCGCTCCTCGACCCGCCCAGGCACGACAGC
GCCGAGACGATCAAGCAGGCGCTCAATCTTGGCGTCAACGTCAAGATGATCACCGGCGAT
CAGCTGGCCATCGCCAAGGAGACGGGCAGGAGGCTCGGCATGGGCACCAACATGTACCCC
TCGTCCGCCCTGCTTGGCCAGAGCGTCGACGAGTCCATCGTGTCACTCCCTGTTGACGAG
CTCATCGAGAAGGCCGATGGGTTTGCCGGTGTTTTTCCCGAGCACAAGTACGAGATCGTC
AAGAAGCTGCAGCAGATGAAGCACATCTGCGGGATGACCGGGGATGGGGTGAACGACGCG
CCGGCTCTCAAGAAGGCCGACATTGGCATCGCCGTGGCCGACGCCACCGACGCCGCCAGG
AGCGCATCGGACATCGTGCTCACCGAGCCAGGGCTCAGCGTCATCATCAGCGCCGTGCTG
ACTAGCCGTGCCATATTCCAGAGAATGAAGAACTACACCATTTACGCGGTGTCCATCACT
ATCCGTATAGTGCTAGGGTTTATGCTGATCGCACTGATTTGGAAGTTCGATTTCTCGCCG
TTTATGATCCTGGTCATTGCCATTCTGAACGACGGCACAATCATGACCATCTCCAAGGAC
AGGGTGAAGCCATCCCCACACCCAGATAGCTGGAAGCTGCCGGAGATCTTCATCACCGGC
ATCGTCTACGGCGCCTACCTCGCGGTGACGACCGTGGTGTTCTTCTTCGCCATGACCAGC
ACCGACTTCTTCAGCGAGAAGTTCAACGTGCGTTCGCTAAGGGGTAACAAGGACGCGATG
ATGTCTGCCCTCTACCTTCAAGTGAGCATTATCAGCCAGGCCCTCATCTTCGTCACCCGG
TCGCGCCGCTGGTGCTTCCAGGAGCGCCCGGGGCTCTGGCTCTGCTTCGCCTTCGTGGTC
GCGCAGATCATAGCCACCGTCATCGCCGTGTACTGCAACTTGCCATTCGCGCACATCAGA
GGCATCGGCTGGGGCTGGGCCGGCGTGATCTGGCTCTACAGCATCATCACCTTCATCCCC
TTGGACCTCTTCAAGTTCGCCATCGGCTACGCGCTCAGCGGCAAGGCATGGGACACCCTC
TTCGAGAACAAGATCGCCTTCACGAACAAGAAGGACTACGGGAAGGAGAAGCGGGAGCTG
CAGTGGGCGACGGCGCAGCGCACGCTGCACGGCCTGCCGACGGCGAACCCGGACAGCACG

CCGCAAGAGAGGAGCAACTACGGCGAGCTCTCGGAGATGGCCGAGCAGGCCAAGCGCCGG
GCGGAGATGGCGAGGCTGCGCGAGCTCAGCACGCTCAAGGGAAGGGTGGAGTCGGCGGTG
AGGCTCAAGGGCCTCGACGTGGAGACCGTCGACAACCACCACTACACTGTATGA
>7350.1_HvAHA6
ATGGCGTCCATGACGCTGGAGGACGTGAAGAACGAGACGGTGGACCTGGAGACCATCCCG
GTGCCGGAGGTCTTCAGCCACCTCAAGTGCAGCAAGCAGGGGCTGAGCGGCACCGAGGCG
CAGAACCGGCTCGCCATCTTCGGGCCCAACAAGCTGGAGGAGAAGACGGAGAACAAGCTG
CTCAAGTTCCTCGGCTTCATGTGGAACCCGCTGTCCTGGGTCATGGAGGCCGCCGCCATC
ATGGCCATCGTGCTCGCCAACGGCGGCGGCAAGCCCCCCGACTGGCAGGACTTCGTCGGC
ATCGTCACCCTGCTCTTCATCAACTCCACCATCAGCTTCATCGAGGAGAACAACGCCGGC
AACGCCGCTGCCGCCCTCATGGCCGGCCTCGCCCCCAAAACCAAGTGTCTGAGGGACGGC
AAGTGGAGCGAGATGGACGCCTCCTTCCTCGTACCCGGCGACATTATCAGCATCAAGCTC
GGGGACATCATCCCCGCCGACGCCAGGCTGCTCGAGGGCGACCCGCTCAAGGTCGACCAG
GCCGCGCTCACCGGCGAGTCCATGCCCGTGAACAAGCACTCCGGCCAGGGGGTTTTCTCC
GGGTCGACGGTGAAGCAGGGCGAGATCGAGGCCGTCGTCATCGCCACCGGCGTGCACACC
TTCTTCGGCAAGGCGGCGCACCTCGTCGACAGCACCAACAACGTCGGTCACTTCCAGCAG
GTGCTCACCGCCATCGGCAACTTCTGCATCATCTCCATCGCCGCCGGGATGCTGGTGGAG
GTTGTCGTCATGTACCCGATCCAGCACCGCGCCTACCGCGACGGCATCGACAACCTGCTC
GTGCTCCTCATCGGCGGCATCCCCATCGCCATGCCCACCGTGCTCTCCGTCACCATGGCC
ATCGGCTCCCACCGGCTGTCCCAGCAGGGCGCCATCACCAAGCGCATGACCGCCATCGAG
GAGATGGCCGGCATGGACGTGCTGTGCAGCGACAAGACGGGCACGCTCACGCTCAACAAG
CTCACCGTCGACAAGACGCTCATCGAGGTGTACGGCAGAGGGATAGACAAGGACACGGTG
CTCCTCTACGCCGCCAGGGCGTCCCGCGTGGAGAACCAGGACGCCATCGACACATGCATC
GTCGGCATGCTGGCCGACCCCAAGGAGGCCCGCGCCGGCATCCAGGAGGTGCACTTCCTC
CCCTTCAACCCCGTGGAGAAGCGCACGGCCATCACCTACATCGACGGCAACGGCGACTGG
CACCGGATCAGCAAGGGCGCGCCGGAGCAGATCATCGAGCTGTGCAGGATGCCCAAGGAG
GCCGAGAAGAGGGTCCACGGCCTGATCGACCAGTACGCCGACCGGGGGCTGCGGTCGCTG
GGCGTGTCGTACCAGCCGGTGCCGGCCAAGAACAAGGACAGCCCCGGCGAGCCGTGGCAG
TTCGTGGGGCTGCTGCCGCTGTTCGACCCGCCCCGGCACGACAGCGCGGAGACGATCCGG
CGCGCGCTGCACCTGGGCGTGAACGTGAAGATGATCACCGGCGACCAGCTGGCCATCGGC
AAGGAGACGGCGCGGCGGCTGGGCATGGGCACCAACATGTACCCTTCCACGACGCTGCTG
GGCGACAAGAGCACGGAGATGAGCGGGCTCCCCATCGACGAGCTGATCGAGAAGGCGGAC
GGGTTCGCCGGGGTGTTCCCGGAGCACAAGTACGAGATCGTGAAGCGGCTGCAGGACCGG
AAGCACATCTGCGGCATGACCGGGGACGGCGTGAACGACGCGCCGGCGCTGAAGAAGGCG
GACATCGGGATCGCGGTGGACGACGCGACGGACGCGGCGCGGTCGGCGTCGGACATCGTG
CTGACGGAGCCCGGGCTGAGCGTGATCGTGAGCGCGGTGCTCACCAGCCGCGCAATCTTC
CAGCGGATGAAGAACTACACCATCTACGCCGTGTCCATCACCATCCGGATCGTGGTCGGG
TTCATGCTGGTGGCGCTGCTGTGGAAGTTCGACTTTGCGCCCTTCATGGTGCTCATCATC
GCCATCCTCAACGACGGCACCATCATGACCATCTCCAAGGACCGCGTCAAGCCGTCGCCC
ACCCCCGACTCGTGGAAGCTCAAGGAGATCTTCGCCACCGGCGTCGTCCTCGGCACCTAC
ATGGCCCTCGTCACCGTGCTCTTCTTCTACCTCGCCCACGACACCGAGTTCTTCCCGGAG
ACGTTCGGGGTGAGGTCGATCCGGGAGAACGAGAAGGAGATGATGGCGGCGCTGTACCTG
CAGGTGAGCATCATCAGCCAGGCGCTCATCTTCGTGACGCGGTCGCGGAGCTGGTCGTTC
GTGGAGCGGCCGGGGGCGCTGCTGGTGATCGCCTTCTTCGTGGCGCAGCTGCTGGCGACG
TGCATCGCCGTGTACGCCAACTGGGAGTTCTGCAAGATGCAGGGGATCGGGTGGGGGTGG
GGGCTCTCCATCTGGGCCTTCACCGTCGTCACCTACATCCCGCTCGACATCCTCAAGTTC
ATCATCCGCTACGCCCTCAGCGGCAGGGCCTGGAACAACATCAACAACAAGACGGCCTTC
ACCAACAAGAACGACTACGGCAAGGTGGAGAGGGAGGCGCAGTGGGCGACGGCGCAGAGG
ACGCTGCACGGCCTCAACCAGGGCAGCAACAACTCCGACCTCTTCGCCGACAACAATGGC

TACCGCGAGCTCTCGGAGATCGCCGAGCAGGCCGCCAAGCGGGCGGAGGTGGCCAGGCTC
AGGGAGCTGCACACGCTCAAGGGCCACGTCGAGTCGGTCGTCAAGCTCAAGGGGCTCGAC
ATTGAGACAATAAACCAGAGCTACACGGTATGA
>1640.2_HvAHA7
ATGGGCGGCCTCGAGGAGATCAGGAACGAGGCCGTCGATCTGGAGAACATCCCCATCGAG
GAGGTGTTCGAGCAGCTGAAGTGCACGCGCCAGGGGCTCACCTCCGATGAGGGGGCACAG
CGTGTTGAAATCTTCGGCCTCAACAAGCTCGAAGAGAAGAAGGAGAGCAAAGTCCTCAAG
TTCCTGGGATTCATGTGGAACCCGCTGTCCTGGGTCATGGAGATGGCCGCCATCATGGCC
ATTGCACTGGCCAACGGTGGTGGGAAGCCTCCGGATTGGCAGGATTTTGTTGGTATCATC
GTTCTTCTGGTCATCAACTCCACCATCTCCTTCATCGAAGAGAACAATGCCGGCAACGCC
GCTGCTGCTCTCATGGCCAACCTTGCACCCAAGACAAAGGTCCTCAGGGATGGTCGATGG
GGCGAGCAGGAGGCATCAATCCTGGTTCCTGGTGACATTGTCAGCATCAAGCTTGGTGAC
ATCGTCCCTGCTGATGCTCGTCTCCTTGAGGGTGATCCTTTGAAGATTGATCAGTCTGGG
CTTACCGGAGAGTCTCTCCCAGTGACCAAGAACCCTGGGGATGAGGTCTTTTCTGGATCA
ACCTGCAAGCAGGGTGAGATTGAGGCTGTGGTCATTGCCACAGGAGTACACACTTTCTTT
GGCAAGGCTGCTCATCTCGTTGACAGCACCAACCAAGTCGGGCACTTCCAGCAGGTCCTC
ACTGCAATTGGAAACTTCTGTATCATCTCGATCGCGGTGGGAATTGTCATTGAGATCATC
GTCATGTTCCCGATCCAGCGCCGCAAGTACCGTGCCGGTATTGAGAACCTGTTGGTCCTC
TTGATCGGTGGTATCCCGATTGCCATGCCTACAGTTTTGTCGGTCACCATGGCCATTGGT
TCTCACAAGTTGTCCCAGCAGGGTGCTATCACCAAGAGGATGACTGCCATTGAAGAGTTG
GCTGGCATGGATGTGCTTTGCAGTGATAAGACTGGCACACTCACCCTTAACAAGCTCAGT
GTTGACAAGAACCTGGTTGAGGTGTTTGCGAAGGGTGTCGACAAAGAACATGTGTTGTTG
TTGGCTGCAAGGGCATCGAGGGTTGAAAACCAGGATGCCATTGATGCTTGCATGGTTGGT
ATGCTTGCTGATCCGAAGGAGGCAAGAGCTGGCATCAGGGAAGTGCACTTCTTGCCCTTC
AACCCTACTGACAAGAGGACTGCTCTGACTTACATTGATGCTGAGGGTAACTGGCACCGT
GCCAGCAAGGGTGCTCCTGAGCAGATCATTACCCTGTGCAACTGCAAGGAGGATGTGAAG
AGGAAGGTGCACTCTGTGATTGAGAAGTACGCTGAGCGTGGGCTTCGTTCGCTTGCTGTT
GCAAGACAGGAAGTACCTGAGAAATCCAAGGATTCTGCTGGTGGACCATGGCAATTCATT
GGTCTGTTGCCCCTGTTTGATCCCCCAAGGCATGACAGTGCCGAGACCATCCGCAAGGCA
CTTGTTCTTGGTGTCAACGTTAAGATGATCACAGGTGACCAACTTGCTATTGGAAAGGAG
ACGGGTAGGAGACTTGGGATGGGCACAAACATGTATCCTTCTTCTGCATTGCTTGGCCAA
AGCAAGGATGGTTCACTTGAGTCACTCCCCGTCGATGAGCTGATTGAGAAGGCTGATGGA
TTTGCCGGGGTCTTCCCTGAGCACAAGTATGAGATCGTCAAGAGGCTGCAAGAGAAGAAG
CACATTGTTGGTATGACTGGAGATGGTGTCAATGACGCTCCTGCTCTTAAGAAGGCCGAC
ATTGGTATTGCTGTTGATGATGCTACAGATGCTGCTCGAAGTGCTTCCGACATCGTGCTT
ACCGAGCCAGGTCTTAGTGTCATTATCAGTGCTGTCCTGACCAGCAGATGCATTTTCCAA
AGGATGAAGAACTACACAATCTACGCAGTTTCCATCACCATCCGTATTGTGCTTGGCTTT
TTGCTTATTGCCTTGATCTGGAAATTTGATTTCGCTCCCTTCATGGTCCTTATCATTGCC
ATTCTCAATGATGGCACTATCATGACAATATCCAAGGACAGAGTTAAGCCATCTCCCTTA
CCCGACAGCTGGAAGCTCAATGAAATCTTCGCCACTGGTGTTGTGCTCGGAACCTACCTT
GCTCTGATGACAGTGGTCTTCTTCTGGATCATCCACAGGACCGACTTCTTCACAAACAAA
TTCGGTGTCAGGTCAATCAGGGAAAATGAAACTGAGAAGATGTCTGCACTGTACCTCCAA
GTCAGTATTGTGAGCCAGGCTCTTATCTTTGTGACTCGTTCTCGCAGCTGGTCCTTTGTT
GAGCGCCCTGGTTTCCTCTTGGTTATCGCCTTCCTTCTCGCGCAATTGGTTGCGACACTC
ATTGCTGTGTATGCCAACTGGGGATTTGCAAGGATCAGTGGAATCGGGTGGGGCTGGGCT
GGTGTTATCTGGCTCTTCAGCATTGTGTTCTACTTCCCACTTGACATTTTCAAGTTCTTC
ATCCGATTTGTGCTGAGTGGCAGGGCCTGGGACAACCTCCTGCAGAACAAGACTGCTTTC
ACCACCAAAGAGAACTACGGCAAAGGTGAGAGGGAGGCACAGTGGGCTACCGCACAGAGA
ACACTCCATGGCCTTCAAGCACCTGAGCCGGCTTCCCACACACTCTTCAACGACAAGAGC

AGCTACCGTGAGCTTTCTGAGATCGCTGAGCAAGCCAAGAGAAGAGCTGAGATTGCAAGG
TTGAGGGAGCTCAACACACTCAAGGGGCACGTTGAGTCCGTGGTGAAGCTCAAGGGCCTT
GACATCGACACCATCAACCAAAACTACACCGTGTGA
>9560.4_HvAHA8
ATGGACTCCAGGGAGGAGCAGCCGCCCGCCGCGGAAGCCAAGCAGGAGCAGCAGCCCGCC
AATGGCCTCTCCGACCTCAACAACGAGACCGTCGATCTGGAGCACATCCCCGTGGATGAG
GTTTTCGAGCACCTGAGGTGCACCAAGGAGGGCCTCACCACCGAGGCCGCCCAGCAGCGG
GTCGAAATCTTCGGCCTCAACAAGCTCGAGGAGAAAAACGAGAGCAAGATCCTCAAGTTC
CTGGGATTCATGTGGAACCCGCTCTCGTGGGTGATGGAGGCCGCCGCAATCATGGCCATC
GCCCTCGCGCACGGAGGAAGCGATCTCAGGGGAAAGTCGATGGGCGTAGACTACCACGAC
TTCGTTGGGATCGTCATTCTGTTGGTCGTCAACTCTACCATCAGCTTCATAGAGGAAAAC
AACGCTGGCAATGCCGCTGCCGCGCTCATGGCCCGCCTCGCACCAAAGGCCAAGGCGCTG
CGTGATGGCACCTGGAATGAATTGGACGCATCGTTGTTGGTTCCGGGCGATATCATCAGT
ATTAAGCTTGGAGACATCATTCCTGCAGATGCGCGTCTTCTGCAGGGCGATCCGCTCAAA
ATTGATCAGTCTGCACTTACGGGAGAATCACTACCCGTAACTAAGCATCCTGGGGGTGGA
GTTTACTCTGGTTCGACTTGTAAGCAGGGTGAAATAGAGGCAGTTGTTATTGCCACAGGG
ATCCATACTTTCTTTGGAAAAGCTGCTCACCTCGTTGAATCAACGACTCATGTTGGCCAC
TTTCAAAAGGTTCTGACATCGATCGGAAACTTCTGCATTTGCTCCATTGCCATTGGGATG
ACTATCGAGTTGATTGTCATGGCAGCTGTTCAACACAGACCGTACCGCCAGACAGTTGAT
AACCTTCTGGTGCTTCTCATTGGAGGGATTCCAATTGCGATGCCCACGGTTCTGTCTGTA
ACTATGGCTATTGGATCACATAAGCTTGCACAACAGGGTGCTATTACCAAGAGAATGACT
GCAATTGAAGAGATGGCCGGAATGGACGTGCTTTGCAGTGACAAAACAGGAACATTGACG
CTCAACAAACTGACTGTAGACAACAATATAATTGAGGTTTTTACTAGAGGTTACGAAAAG
AGTGATGTCGTGTTGATGGCCGCAAGGGCCTCAAGACTGGAGAATCAGGATGCTATTGAT
TTCGCTATTGTCGCAATGCTGCCAGACCCAAAAGAGGCACGTGCTGGCATTGAAGAAGTC
CACTTCCTTCCATTTAACCCAACGGACAAGCGGACAGCTCTCACGTACTTGGATGCTAAG
GGTAAAATGCACAGGGTTAGCAAAGGTGCTCCGGAACAGATTCTGAACCTGGCAGCAAAT
AAATCTGAGATTGAAAGAAAGGTCCATCAAGTAATTGACAGTTTTGCTGAGAGAGGGCTG
CGGTCGCTTGCTGTTGCATACCAGGAAGTTCCTGAAGGTACCAAGGAAAGTGCTGGTGGA
CCCTGGCAATTTATTGGTCTTCTCCCACTCTTTGATCCTCCCCGCCATGATAGTGCTGAA
ACCATACGCCGAGCTCTAGACCTTGGAGTCAGTGTGAAAATGATTACAGGTGATCAGTTG
GCTATTGGTAAGGAAACTGGACGGAGGCTAGGAATGGGCACCAACATGTATCCGTCATCA
TCATTGCTTGGTGACAAAGTAGATAGTGATATCGCGGTCCTACCAGTGGACGAATTGATT
GAGCAGGCAGATGGTTTTGCTGGAGTGTTCCCTGAGCACAAATATGAAATTGTCAAGAGG
CTCCAAGCCAGAAAGCACATATGCGGCATGACAGGAGATGGAGTTAATGATGCACCTGCT
CTGAAGGTGGCAGACATTGGAATCGCGGTGGCTGATGCAACTGATGCTGCTCGTGGGGCC
TCTGATATTGTTCTGACAGAGCCTGGATTGAGTGTGATCATCAGTGCTGTTTTAACGAGT
CGCGCCATTTTTCAGCGGATGAAAAATTATACAATATACGCTGTTTCTATAACCATCCGT
ATTGTGCTTGGTTTTTTGCTCTTGGCGTGCCTTTGGAAGTTTGATTTCCCTCCAATGATG
GTACTTCTGATAGCTATCCTTAATGATGGGACCATCATGACCATATCCAAAGATAGGGTG
AAGCCATCTCCGTGTCCAGACAGTTGGAAGCTAGCAGAGATCTTCGCGACAGGAGTGGTA
CTGGGGACTTACCTGGCCGTAACAACCGTTCTGTTCTTCTGGGCAGCTTACAAGACAGAC
TTTTTCCCGAGACACTTTAATGTTGACACGATGAATATGAAGAAGAAATTGGCCTCTGCC
GTGTACCTCCAAGTGAGCACCATCAGCCAGGCCCTGATATTCGTGACGCGGTCGAGAGGC
TGGTCCTTCACAGAGAGGCCCGGCTTCCTGCTTATGTTTGCGTTCGTCTTAGCGCAGCTG
ATCGCGTCCTTGCTGTCTGCCCTGTTGAACTGGGAAACAGCCAGCATCAGAGGCATCGGG
TGGGGCTGGACGGGCGTCATCTGGCTGTACAACATCGTCATCTACATGCTCCTGGACCCG
ATCAAGTTCGCCGTGCGTTACGGGCTTAGCGGGAGGGCCTGGAACCTCGTCACGGACCGC
AAGGTGGCGTTTTCGAACCAGAAGAACTTTGGGAAGGAGGCGTCGCAGGCGGCATGGGCG

CACCAGCAGCGCACGCTGCACGGGCTTGAGTCGGCGCCGGGGCGGGAGAAGGCGGCGTCC
ACGGAGCTGGGCCACATGGTGGAGGAGACGAAGCGGCGCGCGGAGATCACGAGGCTGAGG
ACGGTGCACACGCTCAAGGGTAAGGTGGAGAACGCCGCCAAGCTCAAGGGCATCGACCTG
GACGACATCAACAACCAGCATTACACCGTGTGA
>77430.3_HvAHA9
>HvAHA9
ATGGATGATGACGGGCTGCGAAAGCCTCTGCTTGGACCCGAGAGTCTCTGCACTGAAGAC
ATTGATCTGGAGAACAAGGTTCTCAAGTTCATCAGCTTCATGTGGAACCCTCTGTCCTGG
GTGATGGAGGCAGCAGCCATCATGGCATTGGTCCTGGCCAATGGGGGTAGTCAGGGTCCT
GACTGGGAGGACTTTGTGGGAATCGTCTGCCTTCTGATCATCAACTCGACGATCAGCTTC
ATCGAGGAGAACAATGCCGGCAATGCCGCGGCTTCGCTCATGGCTCGCTTGGCGCCTAGA
ACAAAGGTTCTTAGAGATGGGCAATGGCAAGAGCTGGATGCCTCTGTTTTGGTGCCCGGG
GACATCATCAGCATTAGGCTCGGTGACATTGTCCCTGCGGATGCGCGGCTGCTCGAGGGG
GATCCTCTCAAAATTGATCAGTCAGCTCTCACTGGAGAATCACTTCCTGTGACCAAAAGG
ACCGGCGACCTAGTGTTCACTGGTTCAACATGCAAGCATGGCGAGATCGAAGCCGTCGTC
ATCGCGACCGGGATCCGCTCATTCTTCGGGAAGGCGGCTCATTTGGTGGACTCCACAAAG
GTTGTTGGCCATTTCCAGAAGGTTCTTACCTGCATAGGCAATTTCTGTATCTGCTCAATT
GTGGTGGGGGTTTTCATTGAGGTTATCATCATGTTCGCGGTCCAGCACCGACCGTACCGG
GAGGGGATCAACAATGTTCTTGTTCTTCTGATCGGAGGGATACCGATCGCGATGCCGACG
GTTTTGTCTGTCACACTCGCAATAGGTTCCCATCGCCTATCTCAACAGGGTGCCATCACC
AAAAGGATGACAGCCATTGAGGAAATGGCAGGCATGGATGTTCTCTGCTGCGACAAAACC
GGAACTCTCACTCTCAACCATCTTACCGTCGACAAAAACCTAATCGAGGTTTTCAGTGGA
GGAATGGACAGGGACATGATCATTCTGTTGGCTGCAAGGGCATCAAGGGTGGATAACCAA
GATGCAATCGACATGGCCATCATAAATATGCTTTCTGATCCCAAAGAGGCACGCGCAAAC
ATCGCCGAAGTTCACTTTCTCCCGTTCAATCCGGTCGACAAGAGAACGGCCATAACATAC
ATTGATTCCGGTGGCAATTGGTTCCGGGTCAGCAAAGGTGCTCCTGAGCAGATCCTCAAC
CTGTGCCACAACAAGGATGACATCGCCGAGAAGGCGCAGCGGGTCGTCGACAGCTTCGCC
GAGAGGGGCCTCCGTTCACTAGCAGTTGCTTACCAGGAGGTCCCGGAGAGATCGAGGCAC
GGCGATGGCGGGCCGTGGGTGTTCTGCGGCGTGCTGCCGCTGTTCGACCCGCCGCGGCAC
GACAGCGCCGACACCATCCGCAAGGCGCTGGACCTCGGCGTGTGCGTGAAGATGATCACC
GGCGACCACCTGGCGATCGCCAAGGAGACCGGCCGGCGGCTCGGGACGGGGACCAACATG
CACCCGTCGGCGGCTCTGTTCGGCCGGCGCGACGGCGACGGCGATGGGGCGGCGGCGGTG
CCGGTGGAGGAGCTGGTGGAGAGCGCGGACGGGTTCGCGGGCGTGTTCCCGGAGCACAAG
CACGAGATCGTGCGGCTCCTGCAGGCGAGCGGGCACGTGTGCGGGATGACGGGCGACGGC
GTGAACGACGCGCCGGCGCTGAAGAAGGCGGACATCGGCATCGCGGTGTCGGACGCGACG
GACGCCGCCAGGGCCGCCGCCGACATCGTGCTCACGGAGCCCGGCCTCGGCGTCATCGTC
TGCGCCGTCCTCACCAGCCGCGCCATCTTCCAGCGCATGAAGAACTACACGATCTACGCC
GTGTGCATCACCATACGGATAGTGGTTGGGTTTGTTCTTCTGGCGTCCATATGGGAGTAC
GACTTCCCGCCATTCATGGTGCTCGTCATAGCCATACTGAACGACGGGACGATCATGGCG
ATATCCAAGGACCGGGTGAAGCCGTCGCGGAGGCCGGACAGCTGGAAGCTGGAGGAGATA
TTCGCCACCGGGGTCGTCATCGGCACCTACCTCGCGCTGCTCACGGTGCTCTTCTACTGG
GCGGTCACCGGAACCACCTTCTTCGAGTCCCACTTCGGGGTGCGGTCGCTGAAGCTGGAC
GCGGAGGAGCTCTCGTCGGCGGTGTACCTGCAGGTGAGCATCACCAGCCAGGCCCTGATA
TTCGTGACGCGCAGCCGGGGCATATCCTTCCTCGACCGGCCGGGGGCGCTGCTCGTCTGC
GCCTTCGTCGTCGCGCAGCTGGTGGCCACCCTGGTGGCCGTGTACGCGGCCGTCGGCTTC
GCGTCGATCAGCGGCGTCGGGTGGCGGTGGGCCGGCGTGATATGGCTCTACAGCCTGGTG
TCCTACCTCCCGCTGGACCTCATCAAGGTGGCCGTCCGCTACGCCCTCAGCGGCGACGCC
TGGGGCCTGCTCTTCGACAGGAAGGCTGCATTTGCAAGGAGGAGGGACTACTACGGCGAG
GAGGATCATCGGAGGGGGGCGGCGCTGTCGACGCGGCGGGCGCTCTCTGATCATCTCCTC

AGCAGCCGGACGCCGCGCTCTGCCGTCGCGGAGCAGGCGAGGCGGCGCGCCGAGATCGCC
AGGCTGGGAGAAACACACGCACTGAGGGCGCACGTCGAGTCTGCCATGAAGCTCGAGCCC
GTCCGGTCGTCGTCCCACTCCGTTTGA
>9780.21_HvAHA10
ATGGCGTCCCTCTCCCTCGAGGACGTCCGCAACGAGACCGTCGACCTGTCGACGGTCACG
GTGGATGAGGTGTTCAAGACGCTAAAGTGCGACAAGAAGGGGCTGAGCGAGGCGGAGGGG
GAGAACCGGCTCAAGCTCTTCGGCCCCAACAAGCTGGAGGAGAAGAAGGAGAGCAAGCTG
CTCAAGTTCCTCGGCTTCATGTGGAACCCGCTGTCCTGGGTCATGGAGATCGCCGCCATC
ATGGCCATCGCCCTCGCCAACGGCGGCGGCAGGCCTCCGGACTGGCAGGACTTCGTCGGC
ATCGTCTCCCTCCTCTTCATCAACTCCACCATCAGCTACATCGAGGAGGCCAACGCCGGA
GACGCCGCCGCCGCGCTCATGGCCGGGCTCGCGCCCAAGACCAAGCTGCTCAGGGACGGC
ACGTGGGACGAGCGGGACGCCGCTATCCTCGTCCCCGGAGACATCATCAGCATCAAGCTC
GGCGACATCATCCCCGCCGACGCCAGGCTGCTCGACGGCGACGCGCTCAAGATCGACCAG
TCGGCGCTCACGGGCGAGTCCATGCCGGTCAACAAGTACGCCGGGCAGGAGGTGTTCTCG
GGATCCACCGTGAAGCAGGGCGAGCTCGAGGCCGTCGTCATCGCCACCGGGGTGCACACT
TTCTTCGGCAAGGCTGCGCACCTCGTCGACAGCACCAACAACGTCGGACACTTCCAGCAG
GTGCTCACGGCCATCGGCAACTTCTGCATCATCTCCATCGCCGCGGGCATGCTCGTGGAG
ATCATCGTCATGTACCCGATCCAGCACCGCGCGTACCGCGACGGCATCGACAACCTCCTC
GTGCTGCTCATCGGCGGCATCCCCATCGCCATGCCCACCGTGCTCTCTGTCACCATGGCC
ATCGGCTCCCACCGGCTGTCGCAGCAGGGCGCCATCACCAAGCGCATGACGGCCATCGAG
GAGATGGCCGGCATGGACGTGCTCTGCAGCGACAAGACCGGCACGCTCACGCTCAACAAG
CTCACCGTCGACAAGACCCTCATCGAGGTGTGCTCCAGGGGCGTCGACAAGGACATGGTG
CTCCTCTACGCCGCCAGGGCGTCCCGTGTCGAGAACCAGGACGCCATTGACACATGCATC
GTCAACATGCTCGCCGACCCCAAGGAGGCCCGCGCCGGCATCAAGGAAGTCCACTTCCTC
CCCTTCAACCCCGTCGACAAGCGCACGGCCATCACCTACATCGACGGCAACGGGGACTGG
CACAGGGTCAGCAAGGGCGCGCCCGAGCAGATCATCGAGCTCTGCAAGATGGCGCCGGAC
GCCGAGAAGAAGGTGCACGCGCTCATCGACTCGTACGCCGACCGCGGCCTCCGTTCCCTC
GGCGTGTCGTACCAGCAGGTCCCGGAGAAGAACAAGGATAGCGCCGGCGAGCCGTGGCAG
TTCATCGGGCTGCTCCCGCTATTCGACCCTCCGAGGCACGACAGCGCGGAGACCATCCGG
CGCGCGCTCCACCTCGGGGTGAACGTGAAGATGATCACCGGCGACCAGCTGGCCATCGGG
AAGGAGACCGGTCGGCGTCTCGGCATGGGCACCAACATGTACCCGTCGACGGCCCTCCTC
GGCGACAAGAACACGCAGGTGGACGGGCTCCCCATCGACGAGCTGATCGAGAAGGCGGAC
GGGTTCGCGGGGGTGTTCCCGGAGCACAAGTACGAGATCGTGAAGCGGCTGCAGGACAAG
AAGCACATCGTGGGGATGACGGGGGACGGCGTGAACGACGCGCCGGCGCTGAAGAAGGCG
GACATCGGCATCGCGGTGGACGACGCGACGGACGCGGCGCGGTCGGCGTCGGACATCGTG
CTGACGGAGCCCGGGCTGAGCGTGATCGTGAGCGCGGTGCTGACGAGCCGCGCCATCTTC
CAGCGGATGAAGAACTACACCATCTACGCGGTGTCCATCACGATCCGGATCGTGATGGGG
TTCACGCTGGTGGCGCTGATCTGGAAGTTCGACTTCGCGCCCTTCATGGTGCTCATCATC
GCCGTCCTCAACGACGGCACCATCATGACCATCTCCAAGGACCGGGTGAAGCCGTCGCCC
AAGCCGGACTCGTGGAAGCTGGACGAGATCTTCGCGACGGGGGTGGTGCTGGGCACCTAC
ATGGCGCTGGTCACCGTGGTCTTCTTCTACCTGGCGCACGACACGGACTTCTTCACGGAG
ACGTTCGGGGTGCCGCCGATCCGGGACAACGACAGGCAGCTCATGGCGGCGCTGTACCTG
CAGGTGAGCATCATCAGCCAGGCGCTCATCTTCGTGACGCGGTCGCGGAGCTGGTCCTTC
GTGGAGCGGCCGGGGTTCCTGCTGCTCTTCGCCTTCTTCGCCGCACAGCTGGTGGCGACG
GCGATCGCCGTGTACGCAAACTGGGACTTCTGCGACATGCAGGGGATCGGGTGGGCGTGG
GGAGGCGCCATCTGGGTCTTCACCCTCGTCACCTACGTCCCGCTGGACGTGCTCAAGTTC
ATCATCCGCTACTCGCTCAGCGGCAAGGGCTGGGACAACGTCCAGAACAAGACGGCGTTC
ACCAACAAGAAGGACTACGGCAGGGGGGAGCGGGAGGCGCTGTGGGCCAAGGAGCAGAGG
ACGCTCCACGGCCTCAGCCAGCCGGCCGCATCCGACCTCCTCAGTAGCAACGAGGAGCTC

TCCGACATCGCCGAGCAGGCCGCCAAGCGCGCCGAGATCGCCAGGCTCCGGGAGCTGCAC
ACGCTCAAGGGCCACGTCGAGTCGGTGGTGAAGCAGAAGGGCATCGACATCGACGCCATC
CCGCAGAACTACACCGTCTAG
