# Supplementary material for: A two-sequence motif-based method for the inventory of gene families in fragmented and poorly annotated genome sequences
Source: BMC Genomics. 2024 Jan 3;25:26. doi: 10.1186/s12864-023-09859-4 (PMC10763278; doi:10.1186/s12864-023-09859-4)
Supplement: Supplementary file 5 — Additional file 5: Supplementary file 5. Hordeum vulgare P4 ATPase coding sequences. [file 12864_2023_9859_MOESM5_ESM.pdf]

Supplementary File 5 – *Hordeum vulgare* P4 ATPase coding sequences

>HvALA1
ATGAGTACCGTTGACCCCTTGCTCCTGTCATCATCCGGAAGTGCAGACTCGCCTTCAAAG
CATACGGCTCCTGCTCGGTCATCGGTGGGCTCATGCCTCTGCCGTGCTGATTCATGCTCT
TCCTCAGTGTATGAGGATTGCGACACTGCTTCTGTTAATTTTGTGGATGAAGGGGATGCC
ATACCAAGGCATTGTCCGGAGGAGTCTGATGTGAGCAGGGTGGCAGAGCGTTTCCAGTCT
GCTGATTCGCATTTCTTCCATCGTCTCTCGGTGGAATGCTCTCAGAAGGAGAGGCAACGG
AAGATTTCTTGGGGTGGTGTAATGGAGATGCAGCGCTCTCCATCATCCCTAGAGGTTGGA
GTAGTATCTTCCTCCCAGGAGAAACCAAATCGACCCCCGAGGGAGCATGACCCAAGGCTA
ATTTACATCAATGATCCCGACAGAACAAACGACAGATATGAATTTACAGGAAATGAGATT
CGGACAAGCAAGTATACTTTGATTACTTTCCTGCCCAAGAACCTCTTCATCCAGTTCCAT
CGGCTGGCTTATGTATACTTCCTAGTTATTGCTGCTCTTAACCAGCTGCCTCCTTTAGCT
GTGTTTGGAAGGACCGCTTCATTGTTCCCGCTACTTTTTGTGTTGTTTGTGACTGCTATA
AAAGATGGTTATGAAGACTGGCGTCGTCACAGATCTGACAGGAATGAAAATAACCGAGAG
GCACTTGTTCTTCAACATGGTGATTTTCGGTCAAAAAAATGGAAACACATTTGTGCGGGG
GAGGTTGTCAAAATTCATTCCAATGAAACAATGCCATGTGATATGGTCCTGTTGAGTACA
AGTGATCCAAATGGTATAGCTTACATCCAAACAATGAATTTGGATGGCGAGTCAAACCTG
AAAACAAGGTATGCTCGCCAGGAAACTGTTACCATGATAAGCAACAGCTCATATTTAGGG
TTGATCAAATGTGAGCAGCCTAACAGAAATATCTATGAGTTTACAGCTACCATGGAGTTG
AATAGTCAGAGGATTCCGCTTGGCCAATCTAACATTGTATTGCGTGGGTGCCAGCTCAAA
AACACAGAATGGATTATCGGGGTAGTCGTCTATGCTGGTCAGGAGACAAAAGCTATGCTA
AACAGTACAATATCCCGTTCAAAGAGCAGCAATCTAGAGAGTTACATGAACAGGGAAACC
CTTTGGTTATCAGCTTTCCTCTTGATCACATGCTCAGTTGTTGCAACAGGGATGGGTGTA
TGGTTATTTAAGAATACTAAAAACCTTGACGCACTACCATACTACAGGAGAAAGTACTTC
ACCTTTGGCCGGGAAAACAGAAAGGATTTTGAGTTCTATGGCCTTGCATTGGAGATATTT
TTCTCCTTCTTGAGTTCTGTGATTATCTTTCAGATAATGATACCAATATCATTATACATC
ACCATGGAGCTAGTTAGAGTGGGGCAGTCATACTTCATGATTGGAGACACTCGGATGTAT
GACAGTAGTTCAGGTTCGAGATTTCAGTGCCGGTCATTGAATATCAATGAAGACCTAGGC
CAAATACGGTATATATTTTCTGACAAAACAGGTACCTTGACTCAAAATAAAATGGAATTT
CAGCAAGCCAGCATCTATGGGAGGAATTATGGGAGCTCCTTGCAGGTCACTAAGTCATCG
AGACAACATGACAGGAAGCCCAAGTCAGAAATCAATGTTGATGCACTACTCTTGGCACTT
CTGAAACAGCCATTGTTTGGGGAGGAAAGACTCGCAGCCCACGATTTTTTCCTTACTTTG
GCTGCATGCAATACTGTCATTCCAGTTAGCACAGGAGGTTCTCCCGATTTGACCAATGAA
GTAAGTGAGGTTGGTGCAATTGATTACCAGGGCGAATCGCCTGATGAGCAGGCCTTAGTA
ATTGCAGCTTCTGCTTATGGATACAAGCTTGTTGAAAGAACAACTGGCCACATTGTTATA
GATGTTCAGGGCGAGAGGATAAGGTTGGATGTTCTTGGTCTGCATGAATTTGATAGTGTG
AGAAAAAGAATGTCTGTTGTTGTAAGATTTCCAGACGACACTGTGAAAGTTCTTGTCAAG
GGTGCTGATACTTCAATGCTTAATATTTTGAAGACCAGGAACCATGATGGACTTTTTGAC
TCTTTGCACGCCAAAACTATAGAAACTACAGAAAATCATTTGTCAAGTTATTCCTCAGAG
GGTCTACGAACCTTAGTAATTGGCTCGAAGTACCTGAGCAATGAAGAATTCAGCGAATGG
CAGGAAAGGTACGAAGAAGCTAGCACCTCCATGACAGAGAGATCGGCAAAGCTCCGTCAG
GCGGCAGCCCTTGTAGAGTGTGATTTGACTCTTCTGGGTGCAACTGGAATTGAAGATAAG
TTGCAGGATGGTGTGCCTGAGGCTATTGAGTCCCTCCGTCAGGCTGGAATCAAGGTTTGG
GTTCTCACAGGGGATAAGCAAGAAACTGCTATATCGATTGGTTTATCATGTCGATTATTA
ACTCAAGGTATGCATTCAATTATCATAAATGGTTCGTCAGAGATTGAATGCAGGCGCCTT
CTAGCTGACGCTAAAGCCAAATTTGGAATCAATGGAGTTATTGCTAATGACAAGTCAGTA
AACATTGAGGAATCACCAAATTTTGATGATGCCGAGTTAGCACTGATAATTGATGGGAAC
TCCCTTGTGTATATTCTAGAGAAAGATCTTGAATCAGAGTTATTTGACTTGGCAACCTCC

TGTAAAGTTGTCATCTGCTGCCGTGTTGCTCCTCTACAAAAGGCTGGAATTGTCGATCTG
ATTAAAAGCAGGACGAGTGACATGACCTTGGCAATCGGTGATGGGGCAAATGATGTTTCA
ATGATACAGATGGCTGATGTTGGTGTTGGTATATGTGGTCAAGAAGGTCGTCAAGCAGTG
ATGGCTTCAGATTTTGCTATGGGGCAGTTCCGCTTTTTGAAGAGATTGCTTCTTGTACAT
GGGCACTGGAATTATCAGCGTATGGCGTACATGATTCTCTACAACTTCTATCGGAATGCT
GTCTTTGTGCTAATGCTCTTCTGGTATATCTTGCACACTGCATATTCCGCAACTCTTGCG
CTAACAGATTGGAGTAGTGTTTTCTATTCCCTTATCTACACATCAGTTCCCACAGTGGTG
GTTGGTATTCTAGACAAGGACTTGAGTCACAATACTCTTCTTTATTATCCGAGACTATAT
GAGGCAGGGCTTCGAAATGAGGGGTACAATATGACACTCTTCTGGATCACAATGCTAGAT
ACCTTGTGGCAAAGCCTCGTCCTTTTCTATGTACCTTTCTTCACATACAGTATCAGTACG
ATGGACATATGGAGTATGGGAAGTTTATGGACAATTGCGGTGGTTATACTGGTCAATATT
CATCTGGCCATGGACATTCAACGTTGGGTGCTTATAACCCATCTTGCCATATGGGGCTCT
ATAGCTGCAACGTTTTTGTGCATGGTGTTAATAGATTCTATACCTATATTCCCTAATTAC
GGGACACTATACAATATGGCTGCTTCAAGAACTTACTGGCTTAGTGTTTGCCTCATAATA
GTCTTGGGGTTGCTTCCCCGGTTTCTTTGCAAAGTAATATATGAGACCTTCTGGCCATCT
GATATTCAAATAGCAAGAGAAGCTGAGTTGTTAAAGAAGCTGCCTCAACAGTGTCGATCA
AGGCCTGAAAGTGATATCAGCTAA

>HvALA2
ATGACGTCTGAGCGCCCGCTCATCGACGCCGCCTCCCCGCACCTGCCGGCGGCGGCGTCG
CAACTCCCGCCGTCACAGCCGGAGCCTCCCGTTCGCGCTGACCACCTGGGCTTATCCGTT
GATGTCCCGGACCCCTTCCGCTCCTCCCGCCGCGACCATCCCGACCCGTCCGCGTCGGAG
CGGGAGCTCCACGAGGGGGGTGAGTACCGCGCCGTCGCCGTGGGGGAGCCCTCGCCGGAG
TTTGACGGAAACTCTGTACGGACGGCCAAGTACTCCGCGCTCACCTTCCTGCCGCGCAAC
CTCTTCGAGCAGTTCCGACGGCTGTCGTATGTCTACTTCCTCGCCATCACCGTGCTCAAC
CAGCTCCCTCAGGTAGCCGTCTTTGGCCGTGGCGCGTCTGTGCTCCCGCTGGCCTTCGTG
CTCTTCGTCACCGCTGTCAAGGACGCCTACGAAGACATCCGCCGCCACCGCTCGGACCGC
AGGGAGAACAACCGCCTCGCCGTCGTCCTCGCGCCACAAACTGCCGGCGAGTTCCTCCCC
AAGAAATGGAAGCACATACGCGTCGGGGACGTCGTGCGCTTCGCGTCCAACGAGACACTC
CCCGCCGACATGGTGCTGCTCGCCACTAGTGACCCCACTGGTCTTGCGCACGTCCAGACC
GTGAATCTTGACGGGGAGACCAACCTCAAGACGAGGTACGCCAAGCAGGAGACGCAGTTG
AGGTTCTCTCAAGATGGCCACGTAGCTGGTATCCTGCATTGCGAGCGGCCCAACAGGAAC
ATTTATGGGTTCCAGGCGAATTTGGAGATCGACGGCAAGCGTGTTTCACTCGGACCCTCC
AACATTGTCCTCCGTGGTTGTGAGCTCAAGAATACCACCTGGGCAATAGGGGTCGTGGTT
TATGCTGGAAAGGAGACTAAGGTCATGCTCAACAACTCTGGGCCGCCATCCAAGCGTAGT
CGCTTGGAGACACAGTTGAATCGGGAGACAGTCATATTGTCCATTATGCTCATTGGGATG
TGCATAACAGCCTCTGTGCTCGCAGGGATTTGGCTGCTGAATCACCAGCGGGAGCTTGAG
TTCACCCAGTTCTTTAGGGAAAAGGATTACACAACTGGGAAGAACTACAACTACTATGGA
ATCGGAATGCAAATATTTGTTACATTCCTCATGGCCGTCATAGTGTATCAGGTCATCATT
CCCATCTCACTGTATATATCAATGGAGTTGGTACGACTTGGGCAAGCATATTTCATGGGC
GCCGACAATGACTTGTATGACGGATCGTCAAGGTCCAGGTTCCAGTGCAGGGCTCTGAAT
ATAAATGAGGATTTGGGGCAGATTAAGTATGTCTTCTCTGATAAGACAGGGACGCTGACA
GAGAACAAGATGGAGTTTGTGTGTGCATCAATTCATGGCGTTGATTACAGTTCTGGCAAA
CATGCATGTGGGTACTCAGTTGTAGTTGATGATCTTCTTTGGACACCAAAAATGGCAGTT
AGGACTGACCCTCAGCTTTTGAAGTTATTGAGCAACCACAGCTCAAATGGGGAAGCAAAG
TTTGTCCTTGAATTCTTCCTTGCTCTTGCTGCCTGCAATACAATCGTGCCACTTGTCCTG
GACACTAGAGATCCTAGACAAAAGTTAATTGATTATCAGGGTGAGTCCCCTGATGAGCAG
GCACTAGCTTATGCTGCAGCATCTTATGGCATTGTGCTTGTTGAGCGGACATCTGGTTAC
GTAGTGATCGATGTCCTGGGTGACAGACAGAGATATGATGTATTAGGACTTCATGAGTTT

GATAGTGATCGTAAGAGGATGTCTGTCATTGTCGGCTGCCCTGATAAGACTGTTAAGTTA
TATGTCAAAGGTGCAGACAGTTCAATGTTTGGAATTATCAATTCATTAGAGCTGGACAAT
GTTCGTGCTACAGAGGCACATCTCCACAAATATTCATCACTTGGCCTAAGAACTCTTGTT
GTTGGTATGCGTGAATTGAGTCAACCTGAATTTGAGGAGTGGCAGTTGGCATACGAGAAG
GCTAGCACAGCAGTACTTGGCAGGGGAAATTTACTCCGATCAATAGCAGCCAATGTAGAG
TGCAATATCCACATATTGGGGGCCTCTGGGATTGAAGATAAGCTTCAAGATGGGGTACCA
GAAGCAATAGAATCTCTTCGGCAAGCAGGCATGAAAGTTTGGATTTTAACAGGGGATAAG
CAGGAGACAGCAATTTCTATTGGCTATTCTTGTAAGCTGCTGACGAATGACATGACACAA
ATTGTGATAAATAACAATTCTAAGGAGTCTTGCAAGAAAAGTCTCGAGGAAGCACTTGCA
AGAACTAAGGAGCACAGAGTTGCTTCATCAATTGGCTCACCAAATCCAGTGTTCGCAACA
GAATCTTCTGGTACAGTTCTTGCTTTGATTGTGGACGGTAACAGCCTTGTTTACATACTT
GAGACAGAGTTGCAAGAAGAGCTTTTCAAAGTAGCAACAGAATGTAGTGCTGTCTTATGT
TGTCGAGTGGCTCCGCTACAAAAGGCAGGGATAGTTGCACTTATCAAAAACAGGACAGAT
GATATGACCTTAGCAATTGGTGATGGAGCAAATGATGTTTCAATGATTCAAATGGCTGAT
GTTGGGGTTGGTATCAGTGGCCAAGAAGGAGGTCAAGCTGTTATGGCATCAGATTTTTCT
ATGGGACAATTCAGATTCTTGGTTCCTCTTCTATTAGTTCATGGTCACTGGAATTATCAA
AGAATGGGTTACATGATCCTTTACAACTTCTACAAGAATGCCACATTTGTCTTGGTTCTT
TTCTGGTATGTGCTTTATACTTCATTCACTTTGACGACTGCTATCACCGAATGGAGTAGT
CTCCTGTATACTGTGCTTTATACATCCCTTCCGACAATTATTGTTGGTATTCTTGACAAG
GATCTTAGCAAGTCAACACTGCTAGCTTACCCAAAGCTTTATGGATCTGGCCAACGGAAT
GAGAAATATAATTTGAATTTGTTTGTCCTCAATATGCTTGAAGCACTTTGGCAGAGTTTG
ATTGTGTTCTACATACCGTATTTTGCATATCGACAAAGTACTATAGGCATGTCTAGTCTG
GGAGATTTGTGGGCACTTGCATCTGTCATCGTTGTAAATATGCAATTGGCCATGGACATC
ATTCAGTGGAATTGGATTATACATGCGTTCATATGGGGGACGATAGCAGCAACAGTGATT
TGTCTCTTTGTGATAGATTCGATATGGGTTCTTCCTGGTTATGGGGTCATCTATCACATA
ATGGGACAAGGCTTGTTTTGGTTACTGCTGCTTATTATTGTTGTCACAGCAATGGTCCCA
CATTTTGCAATCAAGGCTTTCATGGAGCATTTTGTTCCCACTGACATTCAAATCGGACAA
GAAATAGAGAAGTTTAAGGCTTTAAATCAGGTGAATCGTTCAGAAATTCCAATGCGAACG
TTTTCATGA

>HvALA3
ATGGCCGAGGACCATGGATCGTCGCGGCACATGTCGTCCATGTCCATGTCGCACAAGGAG
CTGGGAGACGACGACGCGCGGGTGGTGCATGTGGGCGACGCGGACCGCACCAACGAGCGC
CTCGAGTTCGCCGGGAACGCCGTGCGCACCGCCAAGTACTCGCCGCTCACCTTCCTGCCG
CGGAACCTGTTCGAGCAGTTCCACCGCCTCGCCTACATCTATTTCCTAGTCATCGCCGTG
CTCAACCAGCTCCCCCAGCTCGCGGTCTTCGGCCGCGGGGCCTCCGTCATGCCGCTCGCC
CTGGTCCTCGCGGTCACCGCCGTCAAGGACGCGTACGAGGACTGGCGCCGCCACCGCTCC
GACCGCGCCGAGAATAACCGCCTCGCCGCTGTCCTGTCTCCCGGCGCCGGCGCCCAATTC
GTACCCACCGAGTGGAAGCACGTCCGCGTCGGAGACGTCGTCCGGGTCGGTGCCAACGAG
TCGCCCCCAGCGGACATGGTCCTCCTGGCCACCAGCGACACCACCGGCGTCGCCTACGTG
CAGACGCTCAACCTTGACGGCGAGTCCAACCTAAAGACTCGCTACGCTAAGCAAGAAACC
CTCACGACGCCGCTCGAGCATCTCGCCGGCACCGTCGTCAGGTGCGAGCGCCCGAACCGC
AACATCTACGGCTTCCAGGCCAACCTGGAGCTCCAGGGGGAGAGCCGCCGGATACCACTC
GGCCCGTCCAACATATTGCTGCGCGGCTGCGACCTCAAGAACACGTCCTGGGCCGTGGGC
GTGGTGGTCTACGCGGGGCGCGAGACCAAGGCGATGCTGAACAACGCGGGCACGCCGACC
AAGCGCAGCCGCGTCGAGACGCAGATGAACCGCGAGACGCTCTTCCTCTCCGGCATCCTC
ATCGTGCTCTGCTCGGCTGTGGCCACGCTCACGGGCGTCTGGCTGCGCACCCACCAGGCC
GACCTGGAGCTCGCGCAGTTCTTTCATAAGAAGGACTACCTGAAGGTCGGCAAGGACGGC
AACGAGAACTACAACTACTACGGCATCGCGGCGCAGATCGTGTTCAACTTCCTCATGGCG

GTGATCGTGTTCCAGATCATGATACCCATCTCGCTCTACATCTCCATGGAGCTGGTGAGG
CTCGGGCAGGCATACTTCATGATCCGGGACGCCAAGCTGTACGACGCGTCGACCGACTCG
AGGTTCCAGTGTAGGGCTCTCAACATCAACGAGGACCTGGGGCAGGTCAAGTGCGTCTTC
TCCGATAAGACCGGCACGCTGACGCAGAACAAGATGGAGTTCCGGTGCGCAAGCATCGAT
GGCGTCGATTACAGCGACGTCGCACGGCAGCGACCTGTCGAGGGCGAGCCGGCTTGGGTG
CCAAAGGTGCCGGTGAACGTCGACAGAGAAGTGATGGAGCTGGTGAGGAATGGGGGTGCT
ACGGAGCAAGGGATGAACGCCGGAGAGTTCTTTCTCGCCCTGGCAACGTGCAACACCATT
GTTCCTTTGATCGTTGATGGCCCTGACCCAAAGAAGAAGGTGATTGACTACCAAGGCGAG
TCGCCGGACGAGCAGGCATTGGTTTCTGCCGCCGCGGCGTATGGCTTTGTGCTCGTTGAG
CGAAGCTCCGGGCACATTGTCATTGATGTCCTTGGTCAGAAGCAGAGGTTTGATGTCCTT
GGTCTTCATGAGTTTGACAGCGATCGCAAGAGGATGTCAGTTATAATTGGCTGCCCGGAC
AAGACTGTCAAGCTGTTTGTAAAAGGCGCAGATAGTTCCATGTTTGGAATCATCGACAAA
ACACTGAATCCAGATGTTGTCCAGGCAACTGAGAAACACCTCCATTCATATTCGTCAGTC
GGCCTGCGAACGCTTGTCATCGGTGTGCGGGAACTTAGCCAGGCAGAGTTTCAGGAGTGG
CAAATGGCTTACGAGAAGGCTAGCACTGCATTATTAGGCAGAGGCAATCTCCTACGCAGT
GTGGCCGCCAACATTGAAAGGAACATGCGCCTGCTGGGAGCCTCCGGTGTCGAAGACAAG
CTGCAAGATGGGGTTCCTGAAGCAATTGAGAAACTAAGGGAAGCAGGCATTAAGGTTTGG
GTGCTGACAGGTGACAAACAAGAAACTGCCATCTCCATTGGCTATTCCTGCAAGCTTCTG
ACAAGGGACATGACACAGATTGTAATCAATAGTAATTCAAGAGAGTCATGCAGAAAAAGT
CTGGATGATGCCATCTCCATGGTTAACAAACTTCGGTCGCTATCTTCAGACTCTCAATCT
AGAGTTCCCCTCGCTTTGATCATTGACGGTAACAGTCTCGTCTACATTTTTGACACAGAC
CGGGAGGAGAAGCTTTTTGAAGTCGCGATAGCGTGCGATGTTGTTCTATGTTGTCGAGTG
GCTCCTCTACAGAAGGCTGGGATTGTTGATTTAATAAAGAAGCGAACAAGTGACATGACT
CTAGCTATTGGAGATGGTGCAAATGATGTGTCCATGATCCAAATGGCCGATGTTGGCATT
GGCATCAGTGGTCAAGAAGGAAGGCAAGCTGTGATGGCCTCAGATTTTGCCATGGGGCAG
TTTCGATTTTTGGTCCCACTGTTGTTAGTTCATGGCCACTGGAACTACCAGAGAATGAGC
TACATGATCCTATACAACTTTTACAGAAATGCTACTTTTGTCTTTGTGCTTTTCTGGTAT
GTACTTTACACTGGTTATACCCTGTCAACAGCAATAAATGAGTGGAGCAGTGTGTTATAC
TCTGTGGTCTATACCTCTGCGCCGACTGTCATTGTCGCCATTCTCGATAAGGATCTGAGT
CGAAGGACATTGCTGAAATATCCCCAACTCTACGGTGCGGGGCAGCGCGAGGAGAGTTAC
AATCTAAGATTATTCATTTTCATCATGGTAGACTCCGTTTGGCAGAGCGTTGCAGTTTTC
TTCATCCCTTACCTCGCATACAAAAACAGCGCAATCGACAGCGGTAGCCTGGGAGACCTG
TGGACACTGTCTGTTGTCATTCTTGTCAACATTCACCTTGCCATGGATGTCATTAGATGG
ACTTGGGTCACTCATGCAGCAATATGGGGCAGTATTGTGGCGACATGGATTTGCGTTATC
ATCATAGACTCCATACCTACCTTGCCCGGTTTCTGGGCAATCTATAAGGTGATGGGAACT
GCATTGTTCTGGGCATTGCTTCTTGCGGTGATTGTGGTTGGAATGATCCCTCATTTCGCT
GCAAAGGCCATCAGGGAACATTTCATGCCCAATGACATCCAGATTGCGAGAGAGATGGAG
AAGTCGCAAGATTCTCGTGATGTTTATCATCCAGAAGTCCAGATGAGTACATCCACTCGA
GCGTAG

>HvALA4
ATGGCCTCCTCCGGCGGCGGCAGAGGCGACACGGACGACGGGCACGGGCGCCGGGGCAGG
CGGCGGCGGCGGAAGGTGCTGCTGAGCAAGCTCTACACCTTCGCGGCCTGCGCGCGCCGG
CCGAGCGCCGTGGACGACGAGGGGTCCCGGATCGGCGGGCCCGGGTTCTCCCGTGTGGTC
CACGCCAACGACGCCGCGGCGGCCGCCGACGCCGCCGCCGCCGGAGGGTACCGGTCCAAC
TACGTCTCCACAACAAAGTACAACGCCGTCACCTTCGTCCCCAAGTCGCTCTTCGAGCAG
TTCCGGCGGGTGGCCAACATCTACTTCCTCGTCGTCGCCTGCCTCTCCTACACCCCCATC
GCCCCCTTCCGCGGCGCCACCGCCGTCGGGCCCCTCGTCCTCGTCCTCCTCGTCACCATG
ATCAAGGAGGCCATCGAGGACTGGCGCCGCAAGCAGCAGGACATTGAGGTGAACAACAGG

AAGACAAAGGTGTTCCAGGACGGCGCTTTCCGGCACACCAAATGGACCAAGCTCCGGGTC
GGCGACGTCGTCAAGGTCGAGAAGGACGAGTTCTTCCCCGCGGACCTCGTCCTCCTCTCC
TCCAGCTACGACGACGCCATCTGCTACGTCGAGACCATGAACCTCGACGGCGAGACCAAC
CTCAAGCTCAAGCAGTCTCTCGAGGTCACCTCGCGCCTCCAGGACGACGACAGCTTCGCC
GGCTTCGAGGCCGTCATAAGGTGCGAGGACCCCAACGCCAACCTGTATTCGTTCGTCGGG
AACATAGAGATCGAAGAGCAGCAGCAGCAGTATCCTCTCTCGCCCCAGCAGCTCCTTCTC
AGGGACTCCAAGCTCCGGAACACCGAGTACGTGTACGGCGTGGTGGTTTTCACCGGCCAT
GACACAAAGGTGATGCAGAATGCCACCAGTGCTCCCTCCAAGAGGAGTAAGATTGAGAAG
AAGATGGACGAGGCCATTTACGTCCTTATGTCCATGCTCGTCCTAATTTCGGTCATTGGC
TCCGTCGTCTTTGGCCTTGCGACCAAGCATGATTTGGTGGATGGCAGGATGAAAAGGTGG
TACCTCAGGCCAGATGAACCCGACAAGCTCTACGATCCGAATAACCCGGCCGTGTCGGCG
GCACTGCATTTCTTCACAGCCATGATCCTTTACGGCTACTTCATCCCCATCTCCCTCTAC
GTCTCCATCGAGCTCGTCAAGCTGCTGCAGGCTCTCTTCATCAACAGTGACATCCATATG
TATCATGAGGAGAGTGACACGCCAGCTCATGCCAGGACGTCCAACTTGAACGAGGAGCTG
GGCCAGGTCTACACGATCCTCACGGATAAGACCGGGACTCTGACCTGCAACTCCATGGAG
TTCATCAAATGCTCCATCGCGGGCACGGCGTATGGACGTGGAATCACCGAGGTCGAGAGA
GCCATGGCAAAGAGAAACGGTTCACCCATGATCGCTGATATTGAGGATGGCGTCGAAGCC
TTTCATCAGTCCGAGGGGAGAGCTGCGGTCAAAGGATTTAATTTCAGAGACGAGCGTGTC
ATGGATGGTAACTGGGTTCACCAAGAGCACTCCGGTGCGATCGAGATGTTCTTCCGGTTG
CTGGCCATCTGCCACACATGCATACCGGAGGTCGACGAAGTAACAGGGAAGATCTCATAT
GAAGCAGAGTCTCCAGATGAGGCTGCCTTTGTTGTTGCGGCACATGAACTTGGTTTCACT
TTTTACCAAAGGACGCAGGCAGGCGTTTATCTGCATGAGTTGGATTCCTCGTCTGGAGAA
CAAGTTGACAGATTTTATAAGGTCTTGCATGTCCTTGAGTTCAGCAGTGCTCGAAAGCGG
ATGTCGGTGATAGTTCAGGACGAGGAGGGGAAAACATTTATCTTCAGTAAAGGCGCTGAC
AGTATAATGTACGAGAGGCTGTCAAACTCTGAGAGTGCGTATGGTGAAGCGACGCAGAAG
CACATAAATGATTACGCCGATGCTGGCCTGAGAACACTAGTTCTTGCATACCGTCCACTT
GAGGAGGTCGAATACGCTAAATTCGAAAGGAAGTTCACTGCAGCTAAGAATTCTGTCAGC
GCTGACCGAGATGAGCTGATTGACGAGGCTGCAGATTTGGTAGAGAGGGATTTGATTCTT
CTTGGCGCCACCGCCGTCGAGGACAAACTACAAAAGGGGGTACCCGACTGCATCGACAAA
CTTGCTAAAGCTGGCATCAAGATATGGGTGCTGACGGGCGACAAGATGGAGACCGCCATC
AACATTGGATATGCCTGCAGTCTACTCAGACAAGGGATGAAACAAATAACCATCACACTG
GATACACCAGATATCATTGCCTTGGAGAAAGGTGGCGACAAAGGAGCCATTAATAAGGCG
TCAAAGGTTAGCGTGGTGCAGCAAATCAATGAAGGAAAGAAACTCATAAATGCATCTGGC
AATGAGTCATTTGCTTTGATCATCGATGGGAAGTCACTTACATATGCGCTCAAAGACGAC
ACCAAGGCCACGTTCCTTGATCTTGCAATTGCTTGCGGATCAGTCATTTGCTGTCGTTCT
TCCCCAAAGCAGAAGGCTCTTGTCACGAGGCTAGTCAAAACCGGCACTGGTAAAGTTACG
TTGGCAATTGGCGATGGTGCGAATGATGTAGGCATGATTCAGGAGGCAGATATTGGGGTT
GGGATCAGCGGTGCCGAAGGGATGCAGGCTGTCATGGCGAGCGATGTTTCCATCGCGCAG
TTCCGCTTCCTTGAGCGTCTGCTACTTGTCCATGGGCATTGGTGTTATAGCAGGATCTCG
TCAATGGTATGCTACTTCTTATATAAGAACATCACATTCGGCGTGACCCTGTTCCTGTAC
GAGTCCTTGACAACATTTTCCGGACAAACTTTGTACAACGACTGGTCCATGTCGCTCTAC
AATGTCCTTTTCACGTCACTGCCTGTGATCGCCATGGGCGTGTTCGACCAAGATGTTTCC
GCTCGGTTCTGTCTCAAGTACCCGATGCTCTACCAGGAGGGTCCTCAGAACCTGCTCTTC
CGGTGGTCAAGGCTCCTAGGCTGGATGCTGCATGGCGTCGGCAGTGCTGTGATCATCTTC
TTCCTCACCATCGCGTCACTCAAGCACCAAGCATTCCGTAAAGATGGCGAGGTCATAGAC
CTGTCGATCCTCGGCGCGACCGCCTACACGTGCGTTGTCTGGGCCGTCAACATGCAGATG
GCTATCACAGTGAACTACTTCACCCTCGTCCAGCACATCTGCATCTGGAGCGGCATCTTT
CTATGGTACCTGTTCCTCATAATCTACGGTGCGATCACACCGTCGTTCTCGACCACATTC
TTCATGGTGTTCTCGGAGGCGCTGGGGGGCGCGCCAGCCTACTGGGTGGTGACCCTGCTG

GTGGCGGTGGCGGCGCTCATCCCCTACTTCACCCTGGCGGTGGTGAAGACGTGGTTCTTC
CCGGACTACCACAACAAGATCCAGTGGCTGCAGCACACGGCGAAGCACGAGGACCCCGAG
GAGGAGCTGGGCGTCGTCCTGCGCCAGTTCTCGGTGAGGTCCACCGGGGTGGGTGTGTCG
GCGCGCCGTGACGCCAAGCTCGTCCGCACCAACAGCAAGATCTTCCACGCGGACTCGTCG
TCGCAATCACAAGCCACCATTGAGTTGACATAG

>HvALA5
ATGGCACTGGGAAGGAGGAGGAGGAAGCTGGAGAAGCTGAAGCTGAGTGCATTGTACAGC
TTCGCGCTCTGCGCCAAGGGCGCCACCGAAGATCACTCCAAGATCGGCACCGCGGGGTTC
TCCCGGGTCGTCTACGTCAATGACCCAGACAGGCATGAAGGGGAGGGCTTCAGATACCCC
AAAAATGAGGTGTCCACTACAAAGTACTCCTTGGTCACCTTCGTGCCCAAGTCGCTCTTT
GAGCAGTTCCGGAGGGTGGCCAACTTCTACTTCCTTGTCTCGGGGATCCTCACGTTGACC
CCGCTCGCGCCCTACTCGGCGGTAAGCGCGCTGCTGCCATTGTCGGTCGTGATTACGGCG
ACCATGCTGAAAGAAGGGGTTGAGGATTGGAGGAGGAAAAAACAGGACATTGAGCTCAAC
AATCGAATAGTAAAAGTGCATAGAGGGAATGGCAGTTTTGAAGAGACAAAATGGAAGTAT
ATCAAAATTGGAGATGTGATAAAGGTGGAGAAGGATAATTTCTTTCCTGCTGACTTGATT
CTACTTTCATCTAACTATCCGGATGGAATCTGCTATGTGGAGACTATGAACCTTGATGGT
GAAACAAATTTGAAAATTAAACAAGCTCTTGAGGTGACATTGGATTTACAAGAGGATGGT
AGTTTCACAAGCCTTAGACAAATAATCAAATGCGAAGATCCGAATGCCAATCTTTATTCT
TTTATTGGTACTATGGATTATAAAGGCATGCAGCATCCTCTGTCACCCCAACAACTCCTT
CTTCGGGACTCAAAGCTGCGGAACACTGATTACATATATGGGGCTGTCATCTTCACAGGT
CATGATACAAAAGTGATGCAAAATGCAACTGAGCCACCATCTAAAAGAAGCAAGATTGAG
AAAAAAATGGATAACATCATTTACCTGCTGCTATGTTCTTTACTTGGAATTGCTTTACTT
GGTTCAGTCTTTTTTGGTATATGGACTAAAGACGATTTAAGGAATGGTGAACCAAAACGG
TGGTATCTTCGCCCAGATGATTCGACCGTCTTCTATGACCCGAAACGAGCACCTCTGGCA
TCCTTTTGTCATCTGTTGACAGCCTTAATGTTGTACAACTACTTTATCCCAATTTCTTTG
TACATATCCATTGAGATGGTCAAGATCTTACAGGCTGTATTCATCAACCAGGACATTGAA
ATGTATGATGAAGAGTCAGATAAGCCAACTCATGCTCGAACCTCAAATCTAAACGAGGAA
CTAGGTCAAGTTGACACAATTCTCTCTGATAAGACTGGCACATTGACCTGCAACATGATG
GAGTTTATCAAGTGTTCGATTGCTGGCACTGCATATGGTCAGTCTGTCACAGAAGTTGAG
AAAGCTATGGCCCTGAGGAAAGGAGTGCCACTAGGTGACGAGATAGTAGGTGGAGAGCAC
AAGGAGAAACAAATCGAGGAGAGTCCTCATGTCAAAGGTTTTAATTTGAAGGATCCGCGT
ATAATGGATGGAAATTGGGTACATGAACCTAATAAAGATGTCATCAGGGATTTTTTCCGT
CTGCTAGCCATCTGCCACACATGCATACCTGAAGTAGATGAAACTAATAAAGTTACATAT
GAAGCCGAGTCCCCTGATGAAGCTGCATTTGTTATTGCAGCAAGAGAATTAGGGTTTGAG
TTTTACAAGAGGACACAGACAAGTATAGTTATTCGTGAACGCAATCCTAACCAGAACGTT
GAAGATTATCAGTATAGAAAATATGAGCTCCTAAATGTCTTGGAATTCAGTAGCTCACGA
AGACGGATGTCTGTGATAGTGAAAGAACCGGAGGGAAGGGTATTACTGTTTAGCAAGGGC
GCTGATAGTGTGATGTTCAGAAGGCTTGCACCAGATGGAAGAAAATTTGAGGAAGAGACT
AAAAAGCACATAAATGAGTATTCTGATTCTGGTCTAAGAACGTTGGTTCTTGCATACCGT
GTCTTGGATGAGAAAGAGTACCAGAGCTTCGCTGAAAAATTCAGGACTGCCAAAATATCT
GGAAGTGCCGACAGAGATGAACAAATTGGGGAGGCTGCTGACAGCATTGAGCGGGACTTG
ATTCTTCTTGGTGCTACTGCTGTTGAAGACAAGCTCCAGAAAGGGGTACCAGAATGCATT
GACAAGCTTGCACAAGCAGGAATTAAGATATGGGTGTTGACGGGTGACAAAATGGAGACA
GCTATCAATATTGGCTTTGCATGTAGCCTACTTAGACAAGGAATGATACAAATAATCATC
ACCCTGGAAGCACCTGACATCATTGCATTGGAGAAAAATGGAGACAAAGATTCCATTGCC
AAGGCATCAAAGCAAAGTGTTATGGATCAGATAGAGGATGGAATAAAACAAGTCCCAGCC
TTGGGTCAGTCCGGCATGGAATCTTTTGCGCTGATAATTGATGGTAAATCATTAACTTAT
GCTTTGGAAGATGATGTCAAGTTCAAGTTCTTGGATCTTGCTGTCAAGTGTGCATCAGTC

ATATGCTGCCGATCTTCACCAAAGCAGAAGGCATTGGTTACGAGGCTTGTTAAACATTCA
CATAAAGTTACCTTAGCAATTGGTGATGGGGCAAATGATGTTGGCATGCTTCAGGAAGCT
GACATAGGGGTTGGAATTAGTGGTGTTGAAGGGATGCAGGCTGTCATGGCAAGTGATATT
GCCATCGCCCAATTCCGCTTCCTGGAACGGTTGCTTTTGGTGCATGGACATTGGTGTTAC
CGACGTATTTCAGTGATGATATGCTATTTCTTCTACAAGAACGTGACTTTTGGAGTCACC
ATCTTTCTGTATGAAGCATTTGCATCCTTTTCAGGGAAGCCAGCTTACAATGATTGGTTC
TTGTCACTGTATAATGTATTTTTCACCTCCCTTCCTGTCATTGCGTTGGGCGTATTTGAT
CAGGATGTTTCTTCCCGGCTGTGTTTACAGTATCCGGAGCTGTACCAAGAAGGTGTGCAG
AATGTATTATTCAGCTGGCGTCGAATACTTGGCTGGATGTTTAATGGTGTCGTGAATGCC
ATCTTAATATTTTTCTTCTGCACCACGGCCCTGAAGGACCAAGCATTTCGTCAGGATGGC
CAAGTTGCAGGCTTGGATGCCCTAGGAGCTGCCATGTACACTTGTGTCGTATGGGTCGTC
AACTGCCAAATGGCCCTCTCAGTGAACTACTTCACCATAATCCAACACATATTCATATGG
GGTAGCATTGCTGTGTGGTACATCTTCCTCATGGTTTATGGTAGTATAGACCCAAAGTAC
TCCAAGACAGCATACATGGTCTTCATTGAACAGTTGGCCCCAGCGCTATCATATTGGTTG
GTGACACTTTTCGTGGTGACGGCCACACTCGTCCCGTACTTCTGCTATGCCGCAATTCAG
ATCCGTTTCTTTCCAATGTTCCATAACAAGATTCAGTGGAAAAGATACTTGGGGAAGGCT
GAAGATCCAGAGGTGGCAAGGCAGTTGTCTTCACGACACCGGTCATCGTCGCACCCAAGG
ATGGTTGGGATCTCTGCTCGTCGTGACGGCAAGGCTATGCAAGTTAAAAAGGGAACTGAT
GTAGAGGTTGAAGGATAA

>HvALA6_partiel
ATGGCCCGTGCGCGGAAGCGCGACCGCCTGCGATGGAGCAAGCTCTACACCTTCTCCTGC
TTCCGCCAACCCCAAACCGACGAGGCCGCCGGGCCCGCCGCCGTCAGCGGCAGCCCCGTC
GGCGGCCCTGGCTTCTCGCGCATCGTGCACTGCAACAACTCCATCCTCCACCGCCGGAAG
CCGCTCAAGTACCCCACCAACTACATCTCCACCACCAAGTATAACGTCCTCACCTTCCTC
CCCAAGGCCATCTTCGAGCAGTTCCGCCGCGTCGCCAACCTCTACTTCCTCCTCACCGCC
ATCCTCTCGCTCACCCCGGTCTGCCCCTTCTCCCCCGTCAGCATGATCGCTCCCTTGGCC
TTTGTTGTCGGGCTCAGTATGATCAAGGAGGCCCTGGAGGACTGGCGAAGGTTCATGCAG
GACATGAAAGTGAACAACCGCAAGGTTAGCGCGCACAAGGGTGACGGTGAATTTGAGTTC
CGACATTGGGAGGACCTTTGTGTTGGTGATGTGGTCAGGGTTGAGAAGGACCAGTTCTTC
CCTGCTGATTTGTTGCTCTTGTCCTCGAGCTATGAGGATGGCATTTGCTACGTCGAGACA
ATGAACCTGGATGGCGAGACAAACCTGAAGCTCAAGAGGTCACTGGAGGTTACTCTGCCA
TTGGAAGAGGATGAAACGTTTAAGGATTTCCGGGGAGTGGTAAGGTGTGAAGACCCAAAC
GCGAGCTTGTACACATTCATTGGTAACCTGGAATATGAGAGGCAGATATATTCCCTTGAT
CCGTCTCAGATACTTCTCAGGGACTCAAAACTGAGGAACACAGCCTTCATATATGCAGTA
GTCATTTTTACAGGGCACGACAGTAAGGTGATGCAGAATTCAACCGAGTCGCCATCAAAG
AGGAGCAGGATTGAGAAGAAGATGGATTTGATCATATATATTTTGTTCACTGTTCTGGTT
TTAATATCGCTCATTAGTTCGATTGGTTTTGCTGTGAGGATCAAGCTTGATCTGCCCAGA
TGGTGGTACTTGCAGCCTCAGAACTCCAACAAATTGGACGATCCAACACGCCCTGCTCTT
TCTGGGATTTTCCATCTCATTACAGCACTCATTCTCTACGGGTATTTGATTCCGATCTCA
CTATATGTCTCTATTGAAGTTGTGAAGGTGGCACAAGCACATTTCATTAACCAGGACATT
CATATGTTTGATGAGGAGACTGGCAATACTGCTCAGGCCCGTACGTCAAACTTGAATGAA
GAGCTTGGCCAAGTTCATACGATTTTGTCAGATAAAACTGGCACTTTGACCTGTAATCAG
ATGGATTTCTTGAAGTGTTCAATTGCTGGGGTTTCTTATGGTGTGTGTGCGAGTGAAGTT
GAAAGGGCTGCTGCAAAGCAGATGGCATCAGGCGCTGCTGACCAAGATATTCCCGTGGAA
GATGTATGGGAGAGTAATGAGGATGAAATCCAGTTAGTGGAAGGAGTTACCTTCAGCGTG
GGAAAGACCCAAAAAACCTCGATAAAAGGCTTTAGTTTTGAGGATGACCGTCTTATGCAA
GGGCACTGGACCAAAGAACCAAATTCCAACATGATTCTTATGTTCTTCCGGATACTTGCT
GTTTGTCACACTGCAATCCCCGAGGTGAATGAGGCAACAGGTGCTCTTACTTATGAAGCA

GAATCACCTGACGAGGGGGCTTTTCTTGTGGCAGCCAGAGAATTTGGATTTGAATTTTTC
AAGAGAACACAAGCGAGTGTCTTCCTCAAAGAGAAATACACTTCCTCTAATGGCACAACT
GAGAGGGAGTTCAAGATTCTCAATTTATTGGAGTTCAACAGCAAAAGAAAGCGAATGACG
GTAATTATGAGGGATGAAGATAACCGTATTGTTCTTCTTTGCAAAGGAGCAGATACCATT
ATATTTGATAGACTAGCAAAAAATGGAAGGTTGTATGAGCCAGATACAACCAAGCATCTC
AATGAATATGGTGAGGCAGGCTTGCGGACGTTAGCGCTATCATACAGAATGCTTGAGGAA
TCAGAATATGAATCTTGGAATGCTGAGTTTCTTAAAGCAAAGACATCCATTGGGCCTGAT
AGGGAATTGCAACTTGAGCGAGTCGCAGATTTGATTGAGAAGGAGCTGATCCTTGTTGGT
GCAACAGCGGTTGAGGACAAACTACAAACAGGGGTTCCTCAGTGCATTGATCGCTTGGCG
CAAGCAGGTCTTAAAATCTGGGTTCTGACAGGCGATAAGATGGAAACTGCAATTAACATA
GGATACGCATGCAGTTTACTTAGGCAAGGCATGAAACAGATATCCTTGTCCACAACCGCT
GGTGACCAAGTAGCCCAGGATGCACAAAAGGCTTTGCATTCATCTTTGGAACCTGAGCAG
GCTGCGAAGGAGAGTCTTATGTTGCAAATTGCCAATGGTTCACAAATGGTAAAGCTAGAG
AAGGATCCTGATGCAGCATTTGCTCTAGTTATTGATGGAAAAGCTCTTACATTTGCTTTG
GAAGATGACATGAAGCATATGTTCTTGAATCTTGCAATAGAGTGTGCTTCTGTCATATGT
TGCCGTGTGTCTCCAAGACAGAAAGCACTGGTGACCCGACTGGTCAAAGAAGGCATTGGA
CAAACCACTTTAGCAATAGGTGATGGTGCAAATGATGTGGGCATGATTCAAGAAGCTGAT
ATTGGTGTTGGTATAAGTGGGGTCGAAGGCATGCAGGCTGTGATGGCGAGTGACTTTTCT
ATTTCCCAATTTAGGTTCCTTGAGCGACTTCTTGTTGTACATGGCCATTGGTGCTACAAG
AGAATTGCCCAAATGATCTGTTACTTCTTTTACAAGAATATTACCTTTGGACTTACAATA
TTTTACTTCGAGGCATTCGCTGGATTTTCTGGGCAATCAGTCTATGATGATTGGTTTATG
CTGCTTTTCAACGTTGTTCTTACCTCTCTGCCTGTTATATCACTTGGAGTATTTGAGCAA
GATGTTTCTGCTGAAATCTGCTTGCAGTTCCCAGCGTTATATCAGCAAGGACCAAATAAC
CTTTTCTTTGACTGGTACCGGATTTTAGGATGGATGGCGAATGGCCTCTACTCATCTCTG
GCGATATTCTTCCTCAACATCTGTATATTCTATGATCAAGCAATCCGCTCTGGTGGACAG
ACTGCCGACATGGCTTCAGTGGGAACCACCATGTTTTCCTGCATCATCTGGGCTGTCAAT
ATACAGATTGCTCTGACAATGAGCCATTTCACCTGGATTCAACATCTGTTTGTTTGGGGC
AGCATAGGGACTTGGTATGTTTTCATTATCACATACGGGATGGCTTTGAAGTCCCGAGAC
AACTTCCAGATTATGACAGAAGTTCTCGGGCCAGCTCCCATATACTGGGCAGCAACCCTT
CTGGTGACTGCTGCTTGCAACATCCCCTACCTGATTCACATATCCTACCAGAGATCGTGC
AATCCACTTGATCACCATGTGATTCAGGAGATCAAGTACCTACGGAAAGACGTCGAAGAC
GAAACAATGTGGAAGAGGGAACGGTCCAAGGCGAGACAGAGGACCAAGATTGGTTTCACC
GCAAGGGTAGACGCAAAGATTAAGCAGATCAAGGGGAGGTTGCATAAGAAAAGCCCATCG
TTAACCATCCATACTGTAGCATAG

>HvALA7
ATGGCCCGTGCGCGGAAGCGCGACCGCCTGCGATGGAGCAAGCTCTACACCTTCTCCTGC
TTCCGCCAACCCCAAACCGACGAGGCCGCCGGGCCCGCCGCCGTCAGCGGCAGCCCCGTC
GGCGGCCCTGGCTTCTCGCGCATCGTGCACTGCAACAACTCCATCCTCCACCGCCGGAAG
CCGCTCAAGTACCCCACCAACTACATCTCCACCACCAAGTATAACGTCCTCACCTTCCTC
CCCAAGGCCATCTTCGAGCAGTTCCGCCGCGTCGCCAACCTCTACTTCCTCCTCACCGCC
ATCCTCTCGCTCACCCCGGTCTGCCCCTTCTCCCCCGTCAGCATGATCGCTCCCTTGGCC
TTTGTTGTCGGGCTCAGTATGATCAAGGAGGCCCTGGAGGACTGGCGAAGGTTCATGCAG
GACATGAAAGTGAACAACCGCAAGGTTAGCGCGCACAAGGGTGACGGTGAATTTGAGTTC
CGACATTGGGAGGACCTTTGTGTTGGTGATGTGGTCAGGGTTGAGAAGGACCAGTTCTTC
CCTGCTGATTTGTTGCTCTTGTCCTCGAGCTATGAGGATGGCATTTGCTACGTCGAGACA
ATGAACCTGGATGGCGAGACAAACCTGAAGCTCAAGAGGTCACTGGAGGTTACTCTGCCA
TTGGAAGAGGATGAAACGTTTAAGGATTTCCGGGGAGTGGTAAGGTGTGAAGACCCAAAC
GCGAGCTTGTACACATTCATTGGTAACCTGGAATATGAGAGGCAGATATATTCCCTTGAT

CCGTCTCAGATACTTCTCAGGGACTCAAAACTGAGGAACACAGCCTTCATATATGCAGTA
GTCATTTTTACAGGGCACGACAGTAAGGTGATGCAGAATTCAACCGAGTCGCCATCAAAG
AGGAGCAGGATTGAGAAGAAGATGGATTTGATCATATATATTTTGTTCACTGTTCTGGTT
TTAATATCGCTCATTAGTTCGATTGGTTTTGCTGTGAGGATCAAGCTTGATCTGCCCAGA
TGGTGGTACTTGCAGCCTCAGAACTCCAACAAATTGGACGATCCAACACGCCCTGCTCTT
TCTGGGATTTTCCATCTCATTACAGCACTCATTCTCTACGGGTATTTGATTCCGATCTCA
CTATATGTCTCTATTGAAGTTGTGAAGGTGGCACAAGCACATTTCATTAACCAGGACATT
CATATGTTTGATGAGGAGACTGGCAATACTGCTCAGGCCCGTACGTCAAACTTGAATGAA
GAGCTTGGCCAAGTTCATACGATTTTGTCAGATAAAACTGGCACTTTGACCTGTAATCAG
ATGGATTTCTTGAAGTGTTCAATTGCTGGGGTTTCTTATGGTGTGTGTGCGAGTGAAGTT
GAAAGGGCTGCTGCAAAGCAGATGGCATCAGGCGCTGCTGACCAAGATATTCCCGTGGAA
GATGTATGGGAGAGTAATGAGGATGAAATCCAGTTAGTGGAAGGAGTTACCTTCAGCGTG
GGAAAGACCCAAAAAACCTCGATAAAAGGCTTTAGTTTTGAGGATGACCGTCTTATGCAA
GGGCACTGGACCAAAGAACCAAATTCCAACATGATTCTTATGTTCTTCCGGATACTTGCT
GTTTGTCACACTGCAATCCCCGAGGTGAATGAGGCAACAGGTGCTCTTACTTATGAAGCA
GAATCACCTGACGAGGGGGCTTTTCTTGTGGCAGCCAGAGAATTTGGATTTGAATTTTTC
AAGAGAACACAAGCGAGTGTCTTCCTCAAAGAGAAATACACTTCCTCTAATGGCACAACT
GAGAGGGAGTTCAAGATTCTCAATTTATTGGAGTTCAACAGCAAAAGAAAGCGAATGACG
GTAATTATGAGGGATGAAGATAACCGTATTGTTCTTCTTTGCAAAGGAGCAGATACCATT
ATATTTGATAGACTAGCAAAAAATGGAAGGTTGTATGAGCCAGATACAACCAAGCATCTC
AATGAATATGGTGAGGCAGGCTTGCGGACGTTAGCGCTATCATACAGAATGCTTGAGGAA
TCAGAATATGAATCTTGGAATGCTGAGTTTCTTAAAGCAAAGACATCCATTGGGCCTGAT
AGGGAATTGCAACTTGAGCGAGTCGCAGATTTGATTGAGAAGGAGCTGATCCTTGTTGGT
GCAACAGCGGTTGAGGACAAACTACAAACAGGGGTTCCTCAGTGCATTGATCGCTTGGCG
CAAGCAGGTCTTAAAATCTGGGTTCTGACAGGCGATAAGATGGAAACTGCAATTAACATA
GGATACGCATGCAGTTTACTTAGGCAAGGCATGAAACAGATATCCTTGTCCACAACCGCT
GGTGACCAAGTAGCCCAGGATGCACAAAAGGCTGCGAAGGAGAGTCTTATGTTGCAAATT
GCCAATGGTTCACAAATGGTAAAGCTAGAGAAGGATCCTGATGCAGCATTTGCTCTAGTT
ATTGATGGAAAAGCTCTTACATTTGCTTTGGAAGATGACATGAAGCATATGTTCTTGAAT
CTTGCAATAGAGTGTGCTTCTGTCATATGTTGCCGTGTGTCTCCAAGACAGAAAGCACTG
GTGACCCGACTGGTCAAAGAAGGCATTGGACAAACCACTTTAGCAATAGGTGATGGTGCA
AATGATGTGGGCATGATTCAAGAAGCTGATATTGGTGTTGGTATAAGTGGGGTCGAAGGC
ATGCAGGCTGTGATGGCGAGTGACTTTTCTATTTCCCAATTTAGGTTCCTTGAGCGACTT
CTTGTTGTACATGGCCATTGGTGCTACAAGAGAATTGCCCAAATGATCTGTTACTTCTTT
TACAAGAATATTACCTTTGGACTTACAATATTTTACTTCGAGGCATTCGCTGGATTTTCT
GGGCAATCAGTCTATGATGATTGGTTTATGCTGCTTTTCAACGTTGTTCTTACCTCTCTG
CCTGTTATATCACTTGGAGTATTTGAGCAAGATGTTTCTGCTGAAATCTGCTTGCAGTTC
CCAGCGTTATATCAGCAAGGACCAAATAACCTTTTCTTTGACTGGTACCGGATTTTAGGA
TGGATGGCGAATGGCCTCTACTCATCTCTGGCGATATTCTTCCTCAACATCTGTATATTC
TATGATCAAGCAATCCGCTCTGGTGGACAGACTGCCGACATGGCTTCAGTGGGAACCACC
ATGTTTTCCTGCATCATCTGGGCTGTCAATATACAGATTGCTCTGACAATGAGCCATTTC
ACCTGGATTCAACATCTGTTTGTTTGGGGCAGCATAGGGACTTGGTATGTTTTCATTATC
ACATACGGGATGGCTTTGAAGTCCCGAGACAACTTCCAGATTATGACAGAAGTTCTCGGG
CCAGCTCCCATATACTGGGCAGCAACCCTTCTGGTGACTGCTGCTTGCAACATCCCCTAC
CTGATTCACATATCCTACCAGAGATCGTGCAATCCACTTGATCACCATGTGATTCAGGAG
ATCAAGTACCTACGGAAAGACGTCGAAGACGAAACAATGTGGAAGAGGGAACGGTCCAAG
GCGAGACAGAGGACCAAGATTGGTTTCACCGCAAGGGTAGACGCAAAGATTAAGCAGATC
AAGGGGAGGTTGCATAAGAAAAGCCCATCGTTAACCATCCATACTGTAGCATAG

>HvALA8
ATGGTGCGGGTGGCCACCGCACGGCTGGGCGGCGAGCTGTCGCCGCGGGCGGGACCTTCT
CAGCCCGGACGTGCGGAGTCGTCCAGGACGGCACGTCTCGGTGGCGGAGGCGCCAGCCTC
CGGCGCCAGCCTCAACCGCAGGCGCCCACCGTGCGCACCATCTACTGCAACGATCGCGAG
GCCAACGCGCCCGTCGCATACAAGGGAAATTCTGTATCGACTACAAAGTACAGCGTCTTG
ACATTTCTACCTAAAGGGTTGTTTGAACAGTTCAGGCGGGTGGCAAATCTTTACTTCCTC
ATGATTTCAATCTTGTCGACTACACCAATTAGTCCAGTCCACCCTGTCACCAATGTGGTT
CCCCTTAGTCTAGTGCTTCTGGTGTCACTCATCAAGGAAGCTTTTGAGGACTGGAAACGT
TTCCAGAATGATATGTCAATTAATAATGCACATGTCGATGTATTACAAGGTCAAAAATGG
GAAAGTAGTCCATGGAAAAGACTGCAGGTTGGAGATATTGTGAGGATCAAGCAAGACAGT
TACTTCCCTGCTGATTTGCTCTTCCTATCAAGTACGAACGCTGATGGTGTCTGCTACATC
GAGACAGCTAATCTTGATGGGGAAACCAACCTGAAAATAAGGAAGGCTTTGGAGAAAACT
TGGGACTATGTTCTTCCTGAAAAGGCTTCGGAATTCAAAGGTGAAATACAGTGTGAACAG
CCAAACAATTCGCTTTACACATTTACTGGGAATCTTATTGTGGACAAGCAAACTATACCA
ATTTCACCAAACCAAATACTTCTAAGGGGATGCAGCCTTCGTAATACTGAGTACATTGTT
GCGGTTGTTATATTCACGGGCCATGAGACGAAAGTTATGATGAACTCGATGAATGTTCCC
TCTAAAAGAAGTACATTGGAGAAAAAGCTAGACAAGCTTATTCTAGCTCTATTTGCAACC
CTCTTTACGATGTGTGTCATTGGTGCTATTGGAAGTGGTGTGTTCATTAATGAGAAATAC
TTTTATCTTGGATTGCGTGGGCATGTTGAGGACCAGTTTAATCCCAAGAACAGACTTGTG
GTGACAATTTTAACCATGTTTACTCTAATAACTCTATACTCAACGATCATCCCCATATCT
CTTTACGTGTCCATTGAGATGATCAAATTCATTCAGTGCGCACAGTTTATTAACAATGAT
GTGAATATGTATCATGCTGAGAGCAACACCCCAGCTTTGGCCCGTACTTCTAATCTGAAT
GAGGAGCTTGGGCAGGTTGAGTATATATTTTCTGACAAAACTGGAACACTTACAAGAAAC
TTGATGGAATTCTTTAAATGTTCAATTGGTGGGAAATATATGGAACTGGCATTACAGAG
ATTGAAAAGGGAGGAGCTGAGCGAGCCGGAGTTAGGATTGATGATGATGAGGGTAAAAGA
TCAGCCGTTGCAGTTCACGAGAAAGGATTCAACTTTGACGACGCTAGAATAATGCGTGGT
GCATGGAGAAATGAACCTAATCCTGAGGCCTGCATGGAATTCTTCAGATGCCTGGCAATC
TGTCATACAGTTCTTCCTGAGGGTGAGGAGACACCGGAAAAGATCACTTATCAAGCTGCC
TCTCCTGATGAGGCCGCACTTGTGGCTGCAGCAAAGAATTTTGGTTTCTTTTTTTATAGA
CGTACGCCGACCACAGTAATGGTTCGTGAATCACATGTTGATAGGATGGGTAGTATGCAA
GACGCTGCCTATGAAATTCTGAATGTTTTGGAATTTAACAGTACAAGGAAGCGCCAATCT
GTGGTTTGCCGTTTTCCAAATGGAAAACTTGTTCTCTATTGCAAGGGTGCTGATAATGTA
ATCTATGAACGTTTAGCTGATGGAAATTATGACATTAAAAAGACAAGCAGAGAACATCTA
GAGCAATTTGGATCTGCTGGCTTGCGTACACTTTGCCTTGCTTATCGAGATCTCAGCTTG
GACCAATACAAAAGCTGGAATGAGAAGTTTGTGCAAGCTAAATCTTCTTTACGTGACCGC
GATAAGAAGCTTGATGAGGTGGCTGAATTGATTGAGAAGGACCTTATATTGATAGGTTGC
ACTGCTATTGAAGACAAACTGCAAGAAGGGGTGCCAGCCTGCATCGAAACTCTTTCTGCA
GCTGGCATAAAAATTTGGGTGCTAACTGGAGATAAAATGGAAACAGCAATTAATATAGCA
TACGCATGCAGCCTGGTGAACAACGACACAAAACAGTTCATCATAAGTTCAGAGACAGAC
GCAATTAGAGAGGCTGAAGACAGGGGAGACCCTGTGGAAATTGCGCGAGTTATTAAAGAC
TCGGTAAAACAGAGTCTAAGAAGTTACCTCGAGGAAGCCCACCGTTCTCTAAATAACACA
CCGGAACGAAAGTTGGCTTTTATTATTGATGGAAGATGCTTGATGTATGCTCTAGACCCA
GCTCTGCGTGTGAATCTTCTTGGTTTGAGTTTAATTTGTCACTCAGTTGTATGTTGTCGA
GTTTCTCCACTGCAAAAGGCACAGGTTACTAGCTTAGTTAGGAAGGGTGCTCGTAAGATA
ACTCTCAGCATTGGCGATGGCGCTAATGATGTAAGCATGATTCAAGCCGCTCATGTTGGG
ATTGGCATTAGTGGACAAGAAGGAATGCAAGCAGTTATGGCTAGTGATTTTGCCATCGCT
CAATTTCGTTATCTTACTGATTTGCTTCTTGTACATGGACGGTGGTCATACTTGAGATTG
TGCAAGGTTATCACGTACTTCTTCTACAAGAATCTGACATTTACGCTAACTCAGTTCTGG
TTCACTTTCCAAACTGGCTTTTCTGGTCAGCGGTTTTATGATGACTGGTTCCAGTCGCTG

TATAATGTCATTTTCACAGCACTACCCGTAATTATGGTTGGATTATTTGATAAGGATGTG
AGTGCATCTCTATCAAAGAAATACCCACAACTTTACCAGGAAGGAATTAGGAATACATTC
TTCAAGTGGAAAGTGATAGCGGTGTGGGGTTTCTTTGCTTTCTACCAGTCAATAGTGTTC
TATTACTTCACTGCAGCTGCAAGTCAGCATGGTCATGGCTCATCTGGCAAGATTCTTGGA
CAATGGGATGTTAGCACGATGGCCTTTACTTGTGTTGTGGTTACTGTGAACCTCCGCCTC
CTCATGTCATGCAATTCTATTACCAGATGGCATTATTTCAGTGTAGCTGGCAGTATAGCG
GCCTGGTTTCTGTTTATCTTTATATACTCTGCCATAATGACATCATTTGACAGACAGGAA
AATGTATATTTTGTGATTTATGTTCTGATGAGTACCTTTTTCTTCTACCTCACACTAATT
CTTGTTCCGGTCATCGCTCTCTTTGGTGACTTCCTATATCTATCGCTTCAGCGATGGCTA
TTCCCTTATGACTACCAAGTTGTTCAAGAAATGCACAAGGATGACCCCCATGAATACAGT
ATGATACGTCTTCCAGAGAGGAGCCATTTGAGCCCTGAAGAAGCGAGAAGCTACGCGATT
TCCATGCTCCCCCGAGAGAACTCCAAGCACACTGGTTTTGCTTTTGATTCCCCAGGTTAT
GAGTCATTTTTTGCATCGCAGCAAGGTGTCTGTGTGCCTCACAAGTCATGGGATGTCGCA
AGGAGAGCCAGCATGAAGCAGCAGCGGCAGCAGCCACAACGAACAGGAAGATCCTAA

>HvALA9
ATGAAGCGCTTTGTCTACATAAATGACGAATCTTACCAGAATGATTACTGTGATAACCAG
ATTTCTAACACAAAGTATACCTTATGGAACTTCCTGCCCAAGAATCTATGGGAGCAATTC
AGGCGTTTCATGAATCAATATTTCTTATTGATAGCATGTCTCCAACTGTGGTCACTTATT
ACTCCTGTAAATCCTGCAAGCACATGGGGCCCACTTATAGTAATTTTTGCCGTTTCGGCA
ACCAAAGAGGCCTGGGATGACTACAATCGGTACATTTCAGACAAGCAAGCGAATGAGAAG
AAAGTGTGGATTGTAAAGAATGGTGCACGCAAACATATTCAAGCACAAGATATCCGTGTT
GGTAACATAGTATGGATTCGAGAGAACGAAGAGGTCCCATGTGACCTTGTCTTAACAGGA
ACTTCTGAACCACAAGGCGTTTGTCATGTTGAGACAGCTGCCCTTGACGGGGAAATTGAC
TTGAAAACAAGAGTAATCCCAACAACATGTGTAGGACTGGACTCTGAGCAGTTGCACAAA
ATAAAGGGTGTCATTGAGTGCCCAATCCCAGACAAAGACATAAGAAGATTTGATGCAAAC
ATCAGGCTGTTTCCTCCATTTATTGATAACGATATTTGTCCATTGACTATTAATAACACA
TTACTACAATCATGCTACTTGAGAAATACAGAATGGGCTTGTGGGGTGGCAGTTTATACA
GGCAATGAGACTAAGTTGGGCATGAGTAGGGGAGTCCCAGAGCCCAAGCTTACTGCTATG
GATGCAATGATTGATAAGCTTACTGCTGCTATATTCTTATTTCAACTTGCAGTTGTTGTC
GTTCTTGGATCTGCAGGCAATGTTTGGAAGGACACTGAAGCTCGCAAGCAATGGTATGTC
AAGTATGACGATGATGAACCATGGTATCAGATTTTGGTTATCCCTTTGCGGTTTGAGCTA
CTGTGTTCCATTATGATTCCCATTTCAATAAAGGTTTCATTGGACTTCGTTAAAAGCATG
TATGCGAAGTTCATAGATTGGGACGAGGAGATGTATGACCAGGAAACAGATACACCTGCC
CATGCAGCGAACACAGCAATTAGTGAAGACTTGGGACAAGTTGAGTATATTTTGACAGAT
AAAACTGGGACTTTGACTGAGAACAAAATGATCTTCAGAAGGTGCTGTATTGCTGGCACC
TTGTATGGGAATGAAAGTGGAGACGCGCTTAAAGATATTGAACTGCTGAATGCTGTTGCC
AATAATTCGCCCCATGTCATTAAATTCTTGACAGTCATGGCACTTTGCAATACAGTTATT
CCTATAAAAAGGTTATATCTTTGCCTTCCTGGCGGAACGATATCATATAAAGCTCAGTCC
CAGGATGAGGATGCTCTTGTTAATGCAGCCTCTAATTTGCATGTGGTGCTTGTCAGCAAA
AATGGAAATGATGCTGAAATTCACTTCAACAGACGGGTGATTCAATATGAGATACTTGAT
ATTCTAGAATTCACCTCTGATCGGAAAAGAATGTCTGTTGTAATATCGGATAGTCAAAGT
GGCAAGATTTTCCTCTTGTCCAAAGGCGCAGATGAGGCTATGCTTCCTTTGGCTTATTCT
GGACAGCAAATAAAGACATTTGTTGACGCGGTTGACAAATATGCTCAGCTGGGATTACGC
ACACTCTGCTTGGGATGGCGTGAGCTAAGTTTGGAGGAGTACCTAGAATGGTCTCGGTTA
TTTAAGGAGGCCAACAGCGCGTTAGTTGACCGGGAGTGGAAAGTTGCTGAGGTTTGCCAG
AAATTAGAGCACACCCTGGATATACTAGGTATCAGTGCGATAGAGGATCGCCTCCAGGAT
GGTGTGCCAGAAACTATTGAAATACTGAGACAGTCAGGAATTAATTTTTGGATGTTAACT
GGAGATAAACAAAGCACTGCTATTCAAATAGCTCTTTTGTGCAACTTAATTTCTTCAGAG

CCCAAGGGTCAACTCTTGTATATCAATGGGAAAACCGAAGATGAAGTTGCCAGGAGCTTG
GAAAGAGTTCTGCTCACTATGCGGATAACTTCTTCTGAACCTAAGGAGTTAGCGTTTGTT
GTGGATGGATGGGCTCTTGAAATTATTCTAACACGCTACAAGGAAGCTTTTACAGAACTA
GCAGTTCTTTCAAAAACAGCAATATGCTGTCGTGTAACACCTTCACAGAAAGCACAGCTT
GTCAAGCTTCTGAAGTCGTGTGACTATCGAACTTTGGCAATTGGCGACGGTGGGAATGAT
GTCAGAATGATACAGCAGGCTCACATTGGTGTAGGAATTAGTGGTAGAGAAGGTCTTCAA
GCAGCAAGGGCTGCTGACTATAGCATTGGCAAGTTCAGGTTCTTGAAAAGGCTCATTCTT
GTCCATGGACGATACTCATACAATCGTACTGCCTTCCTTTCACAGTATTCGTTCTACAAA
TCATTGTTAATTTGCTTTATTCAGATACTTTTTTCTTTTGTTTCAGGTATTGCCGGAACA
AGTTTATTCAATTCAGTTAGTTTAATGGCCTATAATGTCTTCTACACAAGCATTCCTGTT
TTAACTACTGTTCTGGACAAGGATTTATCTGAAAAGACAGTGACACAGAACCCAGAAATT
TTACTTTACTGCCAGGCTGGAAGGCTTTTGAATCCAAGTACCTTTGCTGGTTGGTTTGGC
CGATCGTTATATCATGCAATTGTTGTTTTCTTAATCACCATTCATGCTTATGCCAATGAA
AAGAGTGAGATGGAGGAGTTGTCAATGGTTGCTCTCTCAGGAAGTATTTGGTTGCAGGCG
TTTGTCGTGACACTGGAGATGAGTTCTTTCACTTTTCTGCAGTTTCTGGCAATATGGGGA
AACTTTGCTGCTTTCTACGTCATAAATCTCTGTATCAGCACCATACCAACTGCTGGGATG
TACACTATCATGTTCCGCCTTTGTAGACAGCCGTCATACTGGATAACAATGCTGCTAATC
AGTGGAGTTGGCATGGGTCCTGTACTAGCTCTGAAATATTTCAGATACACATACAGTCCG
AGTGCGATTAACATTCTTCAGAAGGCCGAGCGATCTCGTGGGCCTATGTACACCCTGGTA
AATCTGGAATCTCAACTCAGATTAGAGAAAGACAATTCAACCAACTCCATTTTGACCACA
CCAGTCAAGAATAAGAGTTCAGTTTATGAGCCCCTACTGTCTGATTCTCCTATGGCATCC
AGGAGATCACTGGCATCGTCATCATTTGACATCTTTCAGCCAGCTCAGTCAAGGGTTCCT
GCTGCTTATCCCAGAAATATTAAAACCAGTTA

>HvALA10
ATGCAACGTTTTGTATATATCAACGATGAGTCCTGCCGGGATTCCTACTGTGACAATCGG
GTTTCCAATACCAAATACACATTGTGGAATTTTCTCCCTAAGAATTTATTGGAACAATTC
AGGCGTTTCATGAATCAGTATTTTCTGCTAATCGCCTGCCTTCAGTTGTGGCCCACTATT
ACTCCTGTAAGTCCTGCAACTACATGGGGTCCGCTTGCCATAATCTTCATTGTTTCTGCT
TCAAAAGAGGCTTGGGATGATTACAATAGAGATCTTTCCGACAAGAAAGCAAATGAAAGG
ACAATCTGGGTGGTAAAGGATGGCATCCGTAGACAGATCAAAGCAAAGGAGATACATGTC
GGAAATATAGTGTGGCTCCATGAGAATGATGAGATCCCATGCGATCTTGTTCTTATTGGA
ACCTCTGATCCTCAAGGCATCTGTTATGTTGAGACTGCAGCTTTAGATGGTGAAACTGAT
TTGAAAACCAGAATTATCCCTTCAATTTGTGCTGACCTGTCATCGGAGCAATTGGGGAAA
ATCAAGGGTGTAGTCGAGTGCCCTAACCCAGATAATGACATAAGAAGATTTGATGCTAAC
ATGCGCTTGTTCCCTCCTATCATTGATAATGAGAAATGTCCTTTAACTATCAACAATACA
CTTCTTCAGTCATGTTACTTGCGGTACACGGAATGGGCATGTGGAGTTGCAATTTACACA
GGCAATGAAACCAAATCAGGAATGAGCAGGGGAACTGCGGAGCCCAAGCTTACAGCTGCC
GATTCAATGATAGACAAACTCACTGTTGCAATATTTGTTTTCCAAATTGCTGTCGTTCTT
TTGTTGGGTCTTGCTGGCAATATCTGGAAGGACCGCCATGGTCGTAAGCAATGGTATCTC
ATGTATCCTGCTGAAAGACTATGGTACGATTTCCTGGTTATCCCGTTGCGCTTTGAACTA
CTATGTTCAATAATGATTCCAATTTCCATAAAGGTTACTCTTGATCTGGCTAAAGGTGTA
TATGCGAAGTTTATTGATTGTGATGATCAAATGTTTGATCCGGAAACAAATACACCTGCC
CACTCAGCTAACACAGCTATTAGCGAAGACCTTGGGCAGGTTGAATATATATTAAGCGAC
AAAACTGGGACATTGACAGAGAATAGAATGATTTTCAGAAGATGTTGCATAAGTGGTGCT
CTATATGGAGACAACACTGGAGATGCTTTAAAAGATGCCAGACTTCTGAATGCTGTCTCA
AGTAACGATCCGGATGTAGTCAAATTTCTGATGGTGATGGCTCTTTGCAACACAGTTGTT
CCTATCAAAAGCAATGATGGTACTATTTCATACAAAGCACAATCACAAGATGAGGAAGCG

TTGGTCAATGCTGCATCAAACCTGAATATGGTGCTTTCAAGTAAAGACAGTGGCGGCATT
GTTGAGATTTGTTTCAATGGTTCTAAATTTTATTATGAGGTATTGGACGTTTTGGAGTTC
ACTTCTGATCGCAAAAGAATGTCTATTGTGGTAAAGGAGGTGAAGAGCGGGAAGTTTCTT
CTTCTCTCTAAAGGTGCTGATGAAGCTATGTTCCCACGTTCTTGTCCAGGGCAACAAACA
AGGACCTATCTTGAGGCAGTAGAAATGTATTCTCATTTGGGACTGCGAACATTATGTTTG
GGATGCCGTGACTTAGAAGAAGATGAATATAAAGAATGGTCCAAAAAATTTCAAGATGCT
AGCTGCTCACTGGACAACAGGGAGCATAGAATTGCCGAAGTCTGTAACAGCTTAGAGCAA
AGCATTCACATTCTTGGCATCACTGCCATAGAGGACCGTCTCCAGGATGGTGTGCCTGAA
ACTATTAAATTGCTAAGGAAGGCCGGAATCAATGTGTGGATGCTAACTGGCGATAAGCAA
ACTACAGCAATCCAGATTGGACTTCTCTGTAACCTCATAACACCTGAGTCCAACGGTCAA
TTGTTGTCCATCAATGGAAAAACTGAAGATGACATATTACAGAGCTTAGAGAGGGCATTG
GCAATTATGAAGACTGCGTCGGAAAGAAAGGATCTTGCATTCGTTCTGGATGGTTGGGCA
CTCGAAATAATTCTGAAACGTTCGTTGGACTCTTTCACTAAGCTGGCCATGATGTCAAGA
ACAGCAATATGCTGTCGAATGACACCTTTGCAGAAAGCACAGGTTGTTGGGCTCCTGAAA
TCCTCTGGTTCTTTAACTCTTGCAATTGGTGATGGTGGTAATGATGTAAGAATGATTCAA
GAGGCTAACGTTGGAGTAGGGATTAGTGGTAGGGAGGGACTGCAAGCTGCAAGAGCTGCT
GACTATAGCATTGGAAAGTTCAAGTTTCTCAGAAGATTGATACTTGTCCATGGTCGATAT
TCATACAATCGCACAGCATTTATTTCACAGTACTCCTTCTACAAGTCACTGCTGATTTGC
TTTATACAGATTCTTTTTTCCTTTTCGTCAGGGCTTTCTGGAACTAGTATGTTCAACTCG
ATTAGCCTGATGGCCTACAATGTTTTCTACACAAGTCTTCCAGTTATGACAATAATTTTT
GACAAGGATATCTCTGAAACAACAGTTCTGCGATATCCCCAGATTTTACTTTATTCTCAA
GCTGGGAGGCTTTTGAATCGCAGTACATTTGCTGAATGGTTTGGGCGATCGCTGTACCAT
GCATTTGTTGTTTTCGTGATTACCATCAACGCATATGCTGATGAAAAAAGTGACATGGAG
GAACTCTCCATGGTTGCCCTGTCTGGATGCATTTGGTTGCAGGCTTTTGTGGTGACCCTG
GATACTAACTCATTCACTTGTCCGCAAATCACCCTCATATGGGGGAACTTTGTAGCCTTC
TACATGATCAACTTAATACTCAGTGCGGTACCAACCCTTCAGATGCACACTGTCATGTGG
CATCTGTGTAATCAACCTTCATACTGGATCACCATGGCCCTGATTGTTGCCGTAGGAATG
GGCCCGGTATTGGCTCTCAGATACTTGAGGAACGTGTACCGACCTAGTGCCATCGATGTT
CTCCAGCAAATTGAACAAGCCGATGGACATGCCCAAGCCTCTGGGAATTTGGAGTCGTCG
ACTGGCACCTATCTCGACTATTTACTGACTGACTTGCGTAGGAACAAGAGTTCTATCCAT
CAACCTCTGCTTTCGGATTCTGTAGTATCTAGCAGATGA
CTACACCGTCTAG
