# Supplementary material for: A two-sequence motif-based method for the inventory of gene families in fragmented and poorly annotated genome sequences
Source: BMC Genomics. 2024 Jan 3;25:26. doi: 10.1186/s12864-023-09859-4 (PMC10763278; doi:10.1186/s12864-023-09859-4)
Supplement: Supplementary file 6 — Additional file 6: Supplementary file 6. Hordeum vulgare P5 ATPase coding sequences. [file 12864_2023_9859_MOESM6_ESM.pdf]

Supplementary File 6 – *Hordeum vulgare* P5 ATPase coding sequence

>HvP5
ATGGCGCGGTTCGAGGTGAACGGCAAGTCGGTGGAGGGGGTGGACCTGCTGCGGCGGCGC
CACTGGACGGCGCGCCTCGACTTCTGGCCCTTCCTCGCCCTCTACGCGCTCTGGCTGCTG
CTCGCCGTCCCGGCGCTCGACTTCACCGACGCCCTCGTCATCCTCGGCGTGCTCTCCGCC
TCCCACATCCTTGCGTTCCTCTTCACAGCTTGGTCCGTCGATTTCCGGGCGTTCGTCGGG
CACTCCAAGGTTAAGGATATCCATGCGGCTGACGCGTGCAAAGTAATCCCGGCGAAGTTC
CTGGGGTCGAAAGAGATTGTGCCTCTGCATATACAGAAAACTGTTGCCTCATCGTCGGCT
GCAGGTGAGACGGAGGAGATCTACTTTGATTTCCGGAAGCAGAGATTCTTTTACTCGGCA
GAGAAGGATAACTTCTTCAAGCTCCGTTACCCGACAAAGGACTTATTTGGGCATTATATC
AAGGGCACCGGGTATGGAACAGAGGCCAAGATTAACACTGCTATGGACAAGTGGGGGAGA
AACATATTTGAGTATCCACAGCCCACATTTCAGAAATTAATGAAGGAGCAATGCATGGAG
CCCTTCTTCGTTTTCCAGGTTTTTTGTGTTGGCCTTTGGTGTCTGGATGAGTATTGGTAC
TACAGTTTGTTTACACTTTTCATGCTCTTCCTATTTGAGTCTACCATGGCAAAGAATAGA
TTGAAGACATTAACTGAGCTTAGGCGTGTGAAAGTTGATAATCAGATTGTGTTGACTTAC
CGCTGTGGAAAATGGGTTAAAATCTCAGGCACAGAACTACTGCCTGGAGATATTGTGTCA
ATAGGCCGCTCGCCTAGTGGTGAAGATAGATCTGTACCAGCAGATATGCTGTTACTGTCT
GGGTCTGCCATAGTAAATGAAGCTATTCTTACAGGAGAGTCTACTCCACAGTGGAAGGTC
TCAGTTGCTGGCCGTGGCCCTGACGAAATGTTATCGATAAAGAGAGATAAGAATCATATC
CTATTTGGCGGCACGAAGATACTGCAACACACACCAGATAAGTCTGTAAATCTTCGGGCA
CCTGATGGTGGTTGTGTAGCTTTTGTATTGAGAACTGGATTTGAGACTAGCCAGGGAAAA
TTGATGAGAACTATCTTATTCTCAACCGAGAGGGTTACTGCAAATAGCAAGGAAAGTGGG
TTGTTTATACTGTTTTTGCTTTTCTTTGCAATAATTGCATCAGGCTATGTGCTTATGAAG
GGACTGGAGGATCCCACAAGAAGCAGATATAAACTTTTTTTAAGTTGTTCACTGATTCTT
ACTTCTGTGATTCCTCCTGAACTGCCAATGGAGCTGTCCATAGCAGTCAATACATCTTTA
ATTGCTTTAGTACGGCGTGGCATTTTCTGCACGGAACCATTCAGAATACCATTTGCTGGG
AAGGTTGACATATGCTGCTTCGATAAGACTGGGACATTGACATCGGATGATATGGAGTTC
CAAGGAGTTGTTAGTTTGGAAAGCGATGCAGAATTAATATCTGACGCAAATAAGTTGCCT
CTCCGCATTCAAGAAGTGCTTTCCAGCTGCCATGCATTGGTTTTTGTGGACAACAAGCTG
GTTGGTGACCCCCTTGAAAAAGCTGCAATAAAAGGCATAGACTGGATCTACACCTCTGAT
GAGAAAGCCATGTCTAGGAGGCCTGGTGGTCAACCTGTACAGATTGTACACAGACATCAC
TTTGCTTCTCACTTGAAGAGAATGTCTGTTATTGTCCGTATCCAGGAGAAATTTTATGCT
TTCATAAAGGGTGCACCGGAGACCATTCAGGAGAGGTTAGTTGATTTACCTGCTGCATAT
GTGGAAACATACAAAAAATACACGCGTCAGGGCTCCCGGGTCTTGTCTCTTGCATACAAA
CTGCTTCCGGAGATGCCTGTTAGTGAAGCTAGAAGTCTGGAAAGGGATCAAGTGGAAAGT
GACTTAATTTTTGCTGGTTTTGCGGTCTTCAACTGTCCTATAAGGAGTGACTCTGCCGCT
GTCTTGCTCGAACTGGAACAATCTTCACATGACTTGGTTATGATCACTGGGGATCAAGCT
TTGACTGCTTGTCATGTTGCTAGCCAAGTGAATATCTGTTTGAAGCCGGTTCTAATTTTA
ACACGGATGAAGACTGGTGGATTCGAGTGGGTTTCCCCCGATGAAACTGATAGAGTTCCA
TACAGAGCTGAGGAGGTTAAAGAATTATCAGAATCACACGATCTTTGCGTTAGTGGGGAC
TGCTTTGAAATGCTACAAAGGACTGATGCTGTTGTCCAAGTCATTCCTCATGTGAAGGTT
TTTGCTCGCGTTGCTCCAGAACAGAAGGAACTTGTACTGACAACATTTAAGACTGTTGGG
AGGATGACACTGATGTGTGGAGATGGAACCAATGATGTTGGTGCACTGAAACAGGCACAT
GTTGGCATAGCTCTGTTAAATGCTGAACCGGTGCAGAAAGCTGGCTCGAAATCTCAGTCA
TCCAAACTTGAAAGCAAATCAGGGAAGCTGAAAAAACCGAAACCTGCTACGGAGTCATCA
TCACAACTGGTTCCACCAGCTACCAGTTCGGCTAAAGCGCCCAGCAGCCGCCCATTGACT
GCTGCTGAGAAACAGCGCGAAAAGCTGCAGAAGATGTTAGATGAAATGAATGACGAAAGT
GATGGCCGCTCAGCACCGATTGTAAAGCTTGGGGATGCATCCATGGCCTCACCTTTCACA

GCAAAGCATGCCTCTGTTGCCCCCACACTTGATATCATCCGCCAGGGGCGAAGCACCCTA
GTCACTACACTCCAAATGTTCAAGATTCTTGGACTCAACTGCCTTGCAACAGCATACGTT
CTCAGTGTAATGTACTTGGATGGTGTGAAATTGGGTGATGTTCAAGCTACAATCAGTGGT
GTCTTCACTGCAGCATTCTTCCTCTTCATTTCCCATGCTCGCCCACTTCAAGCACTGTCG
GCAGAGCGCCCCCATCCCAACATCTTCTGTGCATACGTCTTCCTTTCCATTCTTGGCCAG
TTTGCAATGCACTTGTTCTTCTTGATGTCAGCTGTCAACTTAGCATCCAAGTATATGCCA
GAGGAATGCATCGAGCCTGATTCAGAGTTCCATCCAAACCTCGTCAACACAGTTTCATAC
ATGGTGAACATGATGATCCAGGTGGCAACCTTTGCTGTGAACTACATGGGCCACCCATTT
AACCAGAGCATATCAGAGAACAAGCCATTCAAGTATGCTCTCTATTCAGCTGTTGTTTTC
TTCACGGTGATCACATCGGATATGTTCAGGGATCTGAACGACTACATGAAGCTCGAGCCC
TTGCCAGAAGGAATGAGGGGCAAACTGCTGCTTTGGGCTATGCTTATGTTTTGCGGTTGC
TATGGATGGGAGCGGTTTTTGCGATGGGCGTTCCCAGGCAAGATGCCAGCATGGGAGAAG
CGACAGAAACAGGCGGTTGCAAACCTAGACAAGAAGCAGGCATAG
