# Supplementary material for: A two-sequence motif-based method for the inventory of gene families in fragmented and poorly annotated genome sequences
Source: BMC Genomics. 2024 Jan 3;25:26. doi: 10.1186/s12864-023-09859-4 (PMC10763278; doi:10.1186/s12864-023-09859-4)
Supplement: Supplementary file 7 — Additional file 7: Supplementary file 7. P-type ATPase protein sequences from A. thaliana, O. sativa, and H. vulgare. [file 12864_2023_9859_MOESM7_ESM.pdf]

Supplementary File 7 – P-type ATPase protein sequences from *A. thaliana*, *O. sativa*, and *H. vulgare*

>HvACA1
MESYLNENFGGVKPKHSSDEALGRWRKVVGVVKNPKRRFRFTANLGKRSEAAAMKRTNQEKLRVAV
LVSKAALQFIHGLAPQSEYTVPAAIKAAGYSICAEELSSVVESHDLKKLKVHGGTEGLISKVSTSE
SDGLSTSKDKLASRQEIFGINKFAETEARSFWVFVWEALQDMTLMILAACAFFSLVVGIATEGWPK
GAHDGLGIVASILLVVFVTATSDYRQSLQFKDLDKEKKKITVQVTRSGYRQKLSIYELLVGDIVHL
SIGDQVPADGLFVSGFSLLINESSLTGESEPVAVNAENPFLLSGTKVQDGSCKMLVTTVGMRTQWG
KLMATLSEGGDDETPLQVKLNGVATIIGKIGLVFAVVTFAVLTESLFRRKIMDGSYLSWSGDDALE
LLEFFAIAVTIVVVAVPEGLPLAVTLSLAFAMKKMMNDKALVRHLAACETMGSATSICSDKTGTLT
TNHMTVVKACICGKIKEVDKSSDTKSLFSELPDSVMTMLSQSIFNNTGGDVVINQGGKREILGTPT
ETAILELGLSLGGDFQAVRKATTLIKVEPFNSAKKRMGVVIQLPGGAFRAHCKGASEIILASCSKY
LNDQGNAVPLDSATMAHLNATIESFANEALRTLCLAYIEVADGFSANDAIPEEGYTCIGIVGIKDP
VRPGVKESVAICRSAGITVRMVTGDNINTAKAIARECGILTEGGLAIEGPDFRIKSAEEMYELIPK
IQVMARSSPLDKHTLVKNLRTTHEEVVAVTGDGTNDAPALHEADIGLAMGIAGTEVAKESADVIIL
DDNFSTIVTVAKWGRSVYINIQKFVQFQLTVNVVALVVNFSSACLTGSAPLTAVQLLWVNMIMDTL
GALALATEPPNDELMKRTPVGRKGNFISNIMWRNIMGQAIYQFFVIWYLQTEGKTLFAIKGDNSDL
VLNTLIFNCFVFCQVFNEVSSREMERINVFKGILNNNVFVAVLGSTVIFQIIIVQFLGDFANTTPL
SLKEWFSCIVIGFIGMPIAAIVKLIPVGSQ
>HvACA2
MESYLEENFGGVKGKNSSEEALRRWRKLCSVVKNPKRRFRFTANLDKRGEAQAIKHANHEKLRVAV
LVSKAALQFIHGLKLRNEYVVPEEVKAAGFQICADELGSIVEGHDSKKLLTHGAVAGIAAKLATSP
TDGLDTAEDSMQRRQDIYGINKFTESEIRSFWVFVWEALQDTTLIILAICAFVSLVVGITMEGWPK
GAHDGLGIVASILLVVFVTATSDYRQSLQFKDLDKEKRKIQVHVTRKGFRQRISIYDLLPGDVVNL
AIGDQVPXXXXXXXPADGLFISGFSLLINESSLTGESEPVVVNEENPFLLSGTKVQDGSCKMLVTT
VGMRTQWGKLMATLSEGGDDETPLQVKLNGVATIIGQIGLFFAVITFIVLSQGLLGKKYHDGLLLS
WSGDDALAMLEHFAIAVTIVVVAVPEGLPLAVTLSLAFAMKKMMNDKALVRNLAACETMGSATTIC
SDKTGTLTTNHMTVVKTCICGNIREVNNPQNASKLRSELPENVVRTLLESIFNNTGGEVVIDQNGK
HQILGTPTETAILEFAMSIGGNFKAKRAETKIAKVEPFNSTKKRMCVLLELAEGGYRAHCKGASEI
VLAACDKFIDETGAVTPLDKATAGKLNGIIDGFAHEALRTLCLAYREMEEGFSIEEQLPLQGYTCI
AIVGIKDPVRPGVRESVAICRSAGVTVRMVTGDNINTAKAIARECGILTEDGLAIEGPDFREKTLE
ELLVLVPKIQVMARSSPLDKHTLVKHLRTTFNEVVAVTGDGTNDAPALHEADIGLAMGIAGTEVAK
ESADVIILDDNFSTIVTVARWGRSVYVNIQKFVQFQLTVNVVALLVNFSSACFTGNAPLTAVQLLW
VNMIMDTLGALALATEPPNDDLMKREPVGRTGKFITNVMWRNIFGQSIYQFVVMWYLQTQGKTFFG
LEGSDADIVLNTIIFNSFVFCQVFNEISSREMEKLNVLKGILNNYVFMCVLSSTVVFQFIMVQFLG
EFANTTPLTSLQWLASVLLGLVGMPIAVVVKLIPVGSS
>HvACA3
MHSGLLARPLQLAPLSGAAAALPCRRIPWACLGLGVSTPGRRGFRFAAAACQLSSSSPPHREKLQV
AVSASKAAVQLQNGLSLQSSQYVVPEDVRAAGFQIGADELTSIVESHDTERLTEHGQLDGIADKLA
TSLTDGISTREDLLEQRQEIYGVNKFAESEPRSFWEFVWDAVQDTTLIILAACAFVSLTVGIATEG
WPNGSHDGIGIFASIILVVSVTATSDYQQSLQFRDLDKEKRKILVQVTRNGFRQRILIDDLLPGDV
VHLAVGDQVPADGVFISGFSLLLDESSLTGESEPVDVSEDKPFLSSGTKVLDGSGQMLVTAVGMRT
QWGKLMAALTEGGNDETPLQVKLSGVANIIGKIGLFFAVLTFVVLSQELIGQKYQDGLLLSWSGDD
VLEILNHFAVAVTIVVVAVPEGLPLAVTLSLAYAMEKMMNDKALVRQLAACETMGSATVICSDKTG
TLTSNRMTVVKACICGNTMEVNGPLIPSSLSSKLPAVAVETLLESILTNTGGEIVIDQNGKQEIIG
TPTETALLEFALSLGGNYKQKRQETKILKVEPFNSVKKRMTVILELPGGGYRAHCKGAAEIVLAAC
DKFIDGSGSIVPLDKKTANMLNDIIETFSSEALRTLCLAYRGLEDGSTQEEIPLQGYTFIGIVGIK
DPVRPGVRESVASCRSAGIAVKMVTGDNINTAKAIARECGILTDGGLAIEGAEFREKTPKELLELI

PKMQVLARSSPLDKLALVKHLRTTSNEVVAVTGDGTNDAPALREADIGLAMGIAGTEVAKESADVV
ILDDNFSTIVTVAKWGRSVYINIQKFVQFQLTVNVVALLVNFSSACFTGDAPLTAVQLLWVNMIMD
TLGALALATEPPNDNLMEKAPVGRTGKFITNVMWRNILGQSLYQFTVIWYLQSQGRYVFGLEGSEA
DTVLNTIIFNTFVFCQVFNEVSSREMEEINVLKGMSENSIFVGVLAGTVIFQFILVQFLGDFANTT
PLTQLQWLICVLFGFLGMPIAAMIKLISVEEREEHDDYGKL
>HvACA4_5
MEKYLQDNFDLPAKNPSEEAQRRWRSAVGSLVVKNRRRRFRHVPDLDQRHQDDAKRRSVQEKIRIA
LYVQQAAITFIGGTKKNEYQLTDDIIKARFSINPEELASITSKHDLKALKMHGGVDGISKKVRTTF
DRGVCATDLDTRQSIYGVNRYAEKPSRSFWMFVWDALQDTTLIILMVCALLSVVVGLASEGWPKGM
YDGLGIILSILLVVMVTAASDYKQSLQFKELDNEKKNIFIHVTRDGGRQKISIFDLVVGDIVHLSI
GDQVPADGLFIHGYSLLIDESSLSGESEPVYTSQDKPFILAGTKVQDGSAKMIVTAVGMRTEWGRL
MSTLSEGGEDETPLQVKLNGVATIIGKIGLIFATLTFVVLMTRFLIDKGLTVGLSNWYSADALTIV
NYFATAVTIIVVAVPEGLPLAVTLSLAFAMKKLMNDKALVRHLAACETMGSAGTICTDKTGTLTTN
HMVVDKIWIAEISKSVTGNNSLEELNSAISSSAWSLLLQGIFENTSAEVVKGNDDKQTVLGTPTEI
AIFEYGLSLQGYCDAEDRSCTKVKVEPFNSVKKKMAVLVSLSGGGHRWFVKGASEIIVEMCDKVID
QDGDVIPLSDDRRKNITDTINSFASDALRTLCLAFKDVDEFDENADSPPNGFTLIIIFGIKDPVRP
GVKEAVQSCITAGIIVRMVTGDNINTAKAIAKECGILTDDGIAIEGPDFRNKSPEEMRDLIPKIQV
CFVFLVLVMARSLPLDKHLLVTNLRGMFHEVVAVTGDGTNDAPALHEADIGLAMGIAGTEVAKESA
DVIVLDDNFTTIINVARWGRAVYINIQKFVQFQLTVNIVALVINFVSACITGSAPLTAVQLLWVNM
IMDTLGALALATEPPNDEMMKRPPTGRGESFITKVMWRNIIGQSIYQLIVLGVLMFAGENLLNING
PDSTTVLNTLIFNSFVFCQVFNEVNSREMEKINIFRGLIGNWVFLGVISATVVFQVVIIEFLGTFA
STVPLSWQFWLVSVGIGSISLIIGAILKCIPVKSGEISGSPHGYRPLANGPDDI
>HvACA6
MDFLKTFDVPAKNPSEDAQRRWREAVGTLVKNRRRRFRMVPDLDKRSQAETQRRNIQEKLRVALYV
QKAALQFIDAARRVEHPLSELARQSGFSISAEELASLVRGHDNKSLRLHKGVEGLARKVNVSLADG
VRSDDVGVRGEVYGANHYPEKPARTFWMYLWDASQDMTLMLLALCAVVSVVIGIATEGWPGGMYDG
LGIMLTISLVVTITAASDYKQSLQFRDLDREKKKIEIQVTRDGFRQKVSIYDIVVGDIVHLSIGDQ
VPADGLFVDGYSFIVDESSLSGESEPVHVSATNRFLLGGTKVQDGSARMLVTAVGMRTEWGNLMET
LSQGGEDETPLQVKLNGVATIIGKIGLAFAVLTFTVLMARFLIGKADAPGGLLTWGMDDALSVLNF
FAVAVTIIVVAVPEGLPLAVTLSLAFAMKKLMQERALVRHLSACETMGSASCICTDKTGTLTTNHM
VVEKVWAAGGATTVSTAKGFEELTSSALSEGFAKLLLEGVFQCSGSEVVRGKDGKTSVMGTPTESA
ILEFGLGVEKNTCIEHAAAPKLKVEPFNSVKKTMGVVVASPNAGGRPRAFLKGASEVVLRRCSNVV
VDRHGSIVALTEKNYGKQVAGAIDTFACEALRTLCLAYQDVASENEVPNDGYTLIAVFGIKDPLRP
GVREAVETCHIAGINVRMVTGDNISTAKAIARECGILTEDGVAIEGPEFRQMSPDQMRAIIPKIQV
MARSLPLDKHTLVTNLRGMFNEVVAVTGDGTNDAPALHEADIGLAMGIAGTEVAKENADVIIMDDN
FSTIINVAKWGRSVYINIQKFVQFQLTVNVVALMVNFVSASFTGSAPLTIVQLLWVNLIMDTLGAL
ALATEPPSDAMMRRPPVGRGDNFITKVMWRNIAGQSIFQLVVLGALLFRGDSLLHMNGDGQLLNTF
VFNTFVFCQVFNEVNSREMEKINVFSGMFSSWVFSAVVGATVGFQVILVELLGTFAGTVHLNGRLW
LLSVLIGSVSLIIGAVLKCIPVGSGDGSSDRHDGYQPIPAGPGAV
>HvACA7
MEGGSGSSWTTSMEGYLKEHFHIPAKNPPTAARLRWRRAVGLVVRNRRRRFREFSALAAVDAAQRR
KILGKVQVVINVHRAALQFINGVKQYHLTHELIEEGFSISPDELAEITGMREDSTILKLHGGTNGI
SRKLKASLQDGVKETEVSTRQKLYGTNKHAEKPPRSFWMFVWDALHDLTLNILIVCALVSLVVGLA
TEGWPKGIYDGLGIILSILLVVLVTASNDYKQSRKFMELDREKQKIYVLVTRDKKTKKVLIHDLVV
GDILHLSIGDVVPADGLFISGYCLLVDESSLSGESEPIQVSEEKPFLHGGSKVVDGTAKMLVTAVG
SRTEWGKIMGTLSDSGVDETPLQVKLNGVATVIGQIGLVFAILTFLVLLARFLVNKGMGVGLMNWS
ANDALTIVNYFAIAVTIIVVAVPEGLPLAVTLSLAFAMKKLMNDKALVRHLAACETMGSVSCICTD
KTGTLTTNHMIVDKVWISDVSKSVNGDAKITELKSVISERAMEILVQGIFVNTGSEVVKGDDGKRT
ILGTPTEAALLEFGLTIEADRYLEYNSIRRVRVEPFNSVKKKMSVIIELPNGGFRSFCKGAPEIIL

GHCDNVLNGEGDIVPLSDMQKQNVLNIINSFASEALRTLCVAFQDLDEFSEEQTIPENGYTLIVLF
GIKDPVRPGVRDAVMTCMAAGITVRMVTGDNINTAKAIAKECGILTEDGIAIEGRELHDKSSDELK
ELLPKIQVMARSLPMDKFKLVTSLKSMYQEVVAVTGDGTNDAPALCESDIGLAMGIAGTEVAKENA
DVIIMDDNFKTIVNVARWGRAVYLNIQKFVQFQLTVNIVALIVNFVSACVIGTAPLTAVQLLWVNM
IMDTLGALALATEPPNDEMMKRSPVRRGDSFITKVMWRNILGQALYQLLVLGTLMIVGKRLLNIEG
PTADKTINTLIFNSFVFCQVFNEINSREMEKINVFRGIFRNWIFVGILTATVIFQVIIVELLGTFA
NTVPLSLELWLLSVVLGSVSMIVSVILKCIPVESVKRDAKPHGYELIPEGPEAL
>HvACA8
MECADVLIAVGRRSTSPSPSSSWQPGRQWRKALNVIRTCHRLARLGILSAGVLPRSTSSYVAIKIH
HDGSDSDADFSSGNANAAAFSVAADDELFKGLVKEKREDCFRRLGGGAGIAAALGSDADRGIHGDG
DDLRRRRESFGGNTYPKPKPKSFFSHVWDALKDVFLIVLLVCAVVSLGFGIKEHGLKDGWYDGVSI
FLAVFLVAAVSAVSNHSQAKRFDKLASESDNIAVTVVRAGRRQEVSIFEILVGDVVMLKIGDSVPA
DGVFLEGHGLQVDESSMTGEPHPVEIDAEKNPFLTGGVKIIDGYGRMLVTAVGTDTLWGEMMSSIT
RENTEATPLQERLERLTSSIGKIGVAVAVLVFTVLTARHFTGSTKDDQGKPLFNKDRVTFDAVFSS
LVVIFQQAVTIIVVAIPEGLPLAVTLTLAFSMKRMVKENALVRRLSACETMGSVTAICTDKTGTLT
LNQMKVTEFWVGTDQPRGATAIAGSVVSLLCQGAGLNTTGSVYKPDNVSPPEITGSPTEKALLSWA
VADLGMDADALKRSCKVLHVEAFNSDKKRSGVMIKNNVTGGVVAHWKGAAEMVLASCSMYVDTDGA
ARELGVEQRRNLEKVINDMAGGSLRCIAFAYKQVNGTEQSKIDDEGLTLLGFVGLKDPCRPEVKAA
IEACTKAGVAVKMVTGDNILTARAIAKECGIISSNDPNGIVIEGHEFRAMSPEQQLEIVDRIRVMA
RSLPLDKLALVQRLKQKGHVVAVTGDGTNDAPALKEADVGLSMGVQGTEVAKESSDIIILNDNFDT
VVTATRWGRCVYNNIQKFIQFQLTVNVAALVINFVSAITTGKMPLTTVQLLWVNLIMDTMGALALA
TDTPTKALMDRPPIGRTAPLISNAMWRNLAAQAAFQIAVLLALQYRGRDLFGTDEKANGTMIFNAF
VLCQVFNEFNARDIEKKNVFAGVLKNRMFLAIIAITLVLQVVMVEVLTRFAGTKRLGLGQWGVCLA
IAAVSWPIGWAVKFIPVPDRTLHDILTRSKSS
>HvACA8
MQHMPMDTVIRVDDGKGNGESFKFLVKEKRQDCFLRMGGGACIAAKLQSHPEHGICGEIADVRRRK
EVFGENKYQKPAETSNSFFNHLCDALRDVVLVALLVCAAVDLGFGIKEHRLKYVLYGVGIFLAVLL
VSAVTAVIGHTQAKWDDKLAAESANIDVTVVRAARRQEVSILDIVVGDVVILKAGDAVPADGVFLT
GHGLRVDESSITCEPQPIEIDDKKYPFLASGVKVIAGYGRMVVTAVGTDTSWGELMKIEADDPKPL
QERLEGLSLTIGKISVAVAAVTFTVRHFTSSTAKLPLLDNSIPLPLPLAVTLMLTFYMMRMVKDNA
LVHRLSALETMASVTVICTDKTGTLTLNQMKVTELWVGTDRVKDTTAIASGVVSLLCQGAGLNTTG
SVYRPDCLLPPEISGSPMEKALLSWAVAYLGMDAEELKRSCEVLHVEAFHSVKKRSRAITMDNTTG
KVVAHWKGAAEVVLANCSMYMDMDGAAHELGVKQMKKLEKVINDMAVVGLGCIAFAYKQVNGTEQT
NIDDEEGLTLLGLVGLKDPCRPEVKAAVEACTKAGVIVQMLTGDNILTARAIAKECGVISSSDLNG
VVMEGQEFRAMSAAQQLEVVDKIRIMARFRPLDKLVLVRRLKQKGHVVAVTGDSTNDSLALKEADV
MLSMGVQGTDVTKDTIILNDVFGTVVMAIRWGRCAYNNFLRFVQFHLTVNGVAIVVNFVSAITTGD
APLTTVQLIMWVNLVMGTLSALAVATDKPADALMDRPPISRTAPLISRAMWRNLAAQAAFQIAVLL
ALQYCGRDVFGTDGKANGKANGTMIFNVFVLCQVLNEFNAREVENKNVFAGVLKSTTFLVVIGVAL
VLQVLMVEVLARFAGTESMGLVQWSVCVAIAALSWPIGWAVKFIPAVPVDSLTRLP
>HvACA9
MPAGGVGVQAPEASPGRYVRRVDEVPPDDDGCDDVLGADGRATGDGDPFDIPAKRAPVERLRRWRQ
AALVLNASRRFRYTLDLKKEEEKEQTRRKIRAHAQVIRAALLFKEAGEKQNGDMELPEILPRGFRI
GEDQLTSMTRDHNYSALQEYGGVKGLTNLLKTNPEKGIHGDEADLSCRANAFGANRYPRKKGKSFW
VFLWEACQDLTLVILIVAAAISLVLGIATEGIKEGWYDGASIAFAVFLVILVTAVSDYKQSLQFQH
LNEEKQNIQVEVIRGGRRIQVSIFDIVVGDVVALKIGDQVPSDGILISGHSLAIDESSMTGESKIV
MKDQKSPFLMGGCKVADGYGTMLVTAVGLNTEWGLLMASISEDNNEETPLQVRLNGVATFIGIVGL
VVAAMVLVVLFARYFTGHTTDPDGTVQFVKGRTGVKSIIFGVIKILTVAVTIVVVAVPEGLPLAVT
LTLAYSMRKMMADKALVRRLSACETMGSATTICSDKTGTLTLNQMTVVRSIVGGIELQPLATIEKL
SPTVTSLVLEAIAQNTSGSVFEPEDGSTVEVTGSPTEKAILSWGLELHMKFAVERSKSAIIHVSPF

NSEKKRGGVAVTGRDSDVHVHWKGAAEIVLALCTNWLDVDGSAHEMTPDKANHFRNYIEDMAEQSL
RCVAFAYRDLDLNDIPSEEQRINWQLPDNDLTLIGIAGMKDPCRPGVRDAVELCTNSGVKVRMVTG
DNLQTARAIALECGILTDPQASAPVIIEGKVFRAYSDAEREAVADKISVMGRSSPNDKLLLVKALK
KNGHVVAVTGDGTNDAPALHEADIGLSMGIQGTEVAKESSDIIILDDNFASVVKVVRWGRSVYANI
QKFIQFQLTVNVAALIINVVAAISSGNVPLNAVQLLWVNLIMDTLGALALATEPPTDQLMKRTPVG
RREPLVTNIMWRNLFIQAVYQVAVLLTLNFRGRDLLHLTKDTLEHSSKVKNSFIFNTFVLCQVFNE
FNARKPEELNIFEGVSRNHLFLAVVSVTVVLQVIIIEFLGKFTSTVKLSWQLWLVSLAIAFVSWPL
ALVGKFIPVPQTPLKNLILKCWPKGKNQGDEGAAPPV
>HvACA10
MASPPPPPEVAVAVGEEEGEGREAQGEDAFDIPGKNAPRDRLRRWRQIALVLNASRRFRYTLDLER
DEERENLRRIIRAHAQVIRAVFLFKKAGQKELQESYNGTKPESLSQRFPIDLEKLVMLNRDHDAIM
LQEVGGVSGLSDLLKSNLERGVSSNEDDLLHRRDLFGANTYPRKKRKGIWRFVFEACWDLTLVILM
VAAAISLSLGIATEGVKDGWYDGGSIFFAVFLVIFVTATSDYRQSLQFQHLNEEKQNIKVEVIRGG
KRVGVSIFDLVVGDVVPLKIGDQVPADGVLICGHSLAIDESSMTGESKIVHKDQKAPMLMSGCKVA
DGYGSMLVTGVGTNTEWGMLMANLSEDIGEETPLQVRLNGVATLIGIVGLSVAGVVLVVLWIRYFT
GHSSNPDGTTAFVAGTTGAKQGFMGAISIFTIAVTIVVVAVPEGLPLAVTLTLAYSMRKMMRDKAL
VRRLSSCETMGSATTICSDKTGTLTLNKMTVVEAYLSGTKLNPCNNTGMMSSSAASLLVEGIAQNT
AGAVFSPEDGGTAEIAGSPTEKAILSWGLKIGMNFNDVRSKSSVLHVLPFNSMKKCGGVAVQVSDA
YAHIHWKGAAEIVLASCKSLLSIDGSVHPMSSDKYNELKRSIDDMAMSSLRCIAFAYCTCELTMVP
REDLDKWQLPEDNLTLLGMVGIKDPCRPGVRDAVQLCSAAGVKVRMVTGDNVETAKAIALECGILN
AKDVASETIIIEGKVFREMSETAREEVADKITVMGRSSPNDKLLLVQVLKRKGHVVAVTGDGTNDA
PALHEADIGLSMGISGTEVAKESSDIIILDDDFTSVVKVVRWGRSVYANIQKFIQFQLTVNVAALV
INVVAAVSSGAIPLNAVELLWVNLIMDTLGALALATEPPTDNLMKRHPVGRREPLVTNVMWRNLFI
QALYQIAVLLIFNFDGKRIFHLHNESREHADKIKNTFVFNAFVFCQIFNEFNARKPEEKNVFLGVT
SNRLFMGIVGITTILQILIIEFLGKFFGTVRLGWKLWVLSVAIGAVSWPLAYVGKSIPVPATPFQD
YFKHCSVWRRPCRRGDEEQGSKS
>HvACA10
DNDTSAAHYVGCRGRSQAALVLNASRRFRYTLDLKKEEEKEIIRRTIRSHAQVIRAVFLFKEAGEN
DPKEACTGIKHATASRSFPIELEKLKTLNRNHDSVLLQEFRGVKGLSDLLKSNLDRGINPTEDELL
QRRNAFGANTYPRKKRKNILRFVFDACKDLTLIILMVAAAISLTLGMATEGVEEGWYEGGSIFLAV
FLVILVTATSDYRQSLQFQHLNEEKQNIQVEVLRGGKRFRASIFDLVVGDVVPLNIGDQVPGDGIL
ISAHSLAIDESSMTGESKTVHKDQKAPFLMSGCKVADGYGSMLVTGVGINTEWGQLMANLSEDNGE
ETPLQVRLNGVATFIGMVGLSVAGVVLGVLAIRYFTGHTKNPDGTVQFRAGTTGLKQGFMGAIRIL
TIAVTIVVVAVPEGLPLAVTLTLAYSMRKMMRDKALVRRLSSCETMGSATTICSDKTGTLTLNKMT
VVEAHFIGTRLDPCDDVRAISSSSAALLIEGIAQNTTGTVFLPEDGGAADVTGSPTEKAILSWGLK
IGMNFSDVRSKSSVLHVFPFNSEKKRGGVAVQADASDTGVHIHWKGAAELVLSSCKSWLSLDGSVQ
PMGAQKRNECKKSIEDMAKCSLRCVAFAYCQCDIEIIPKENIADWKLPDEDLTLLGIVGIKDPCRP
GVRNAVQLCKNLGKVRMVTGDNIETAKAIALECGILDANGAISEPFVIEGKVFREMSEIARGEIAD
KITVMGRSSPNDKLLLVQALKRKGHVVAVTGDGTNDAPALHEADIGLAMGMSGTEVAKESSDIIIL
DDDFTSVVKVVRWGRSVYANIQKFIQFQLTVNVAALVINVIAAVSSGDVPLNAVELLWVNLIMDTL
GALALATEPPTDNLMKRQPVGRREPLVTNIMWRNLFVQAIYQIAILLIFNFSGKKI
>HvACA11
MESSSSGGARARRSGSWGSVGGSGGGSDPFDIPGKGAPVERLKKWRQAALVLNASRRFRYTLDLKK
EAQKEEVIRKIRAQAHVIRAAFRFKEAARLGSQTKDVPEAHADGALGFGIKEDQLTALTRDHNYSA
LQQYEGISGLANMLKTDPDKGISGDDSDIDARKNAFGSNTYPRKKGRSYMAFVWDACKDLTLIILM
VAAAVSLALGIYTEGIKEGWYDGASIAFAVLLVIFVTATSDYKQSLQFQNLNEEKQNIQLEVVRGG
RRIKVSIYDLVIGDVVPLKIGDQVPADGVLISGHSFSIDESSMTGESKIVNKDQKSPFLMSGCKVA
DGYGTMLVTAVGINTEWGLLMASISEDSGEETPLQVRLNGVATFIGIIGLSVAVAVLVVLLARYFT
GHTYNPDGSPQYVKGKMGVGETIRGVVKIFTVAVTIVVVAVPEGLPLAVTLTLAFSMRKMMRDKAL

VRRLSACETMGSATTICSDKTGTLTLNQMTVVEAYFGGEKMDPPDNTQKLSAPVSTMIIEGIAQNT
SGSIFEPEGGQAPEVTGSPTEKAILSWGLQLGMKFSETRSKSSILQVFPFNSEKKRGGVAVQVGDS
EVHVYWKGAAELILESCTSWVDMDGSNHSMTPEKAAEFKKFIEDMAVASLRCVAFAYRPCEMSDVP
KEDQRADWVLPEDNLIMLGIVGIKDPCRPGVQDSIRLCTAAGIKVRMVTGDNLQTARAIALECGIL
TDPNVSEPTIIEGKTFRELTDLEREEVADKISVMGRSSPNDKLLLVKALRSRGHVVAVTGDGTNDA
PALHEADIGLSMGIQGTEVAKESSDIIILDDNFATLVRVVRWGRSVYANIQKFIQFQLTVNVAALI
INVVSAVSSGDVPLNAVQLLWVNLIMDTLGALALATEPPNNHLMERAPVGRREPLITNIMWRNLLI
MAFYQVAILLTLNFKGLSLLRLEHDNPAHAEMLKNTFIFNTFVLCQVFSEFNARKPDELNIFKGIA
GNRLFIAIIAITVVLQVLIIEFLGKFTTTVRLSWQLWLVSIGLAFISWPLALVGKLIPVADRPLLD
MFSCCCPAKKEAGDAKEDDGVKHIEVV
>HvAHA1
MAAMASRQQEGSLDAVLKEAVDLEHIPIDEVFENLRCSHEGLTSEQAQQRLQIFGPNKLEEKEESK
LLKFLGFMWNPLSWVMEAAAIMAIALANGGGKPPDWQDFVGIITLLLINSTISFIEENNAGNAAAA
LMARLAPKAKVLRDGRWTEEEAAVLVPGDIISIKLGDIIPADARLLDGDPLRIDQSALTGESLPAT
KGPGDGVYSGSTVKQGEIEAVVIATGVHTFFGKAAHLVDSTNQVGHFQQVLTAIGNFCICSIAVGM
FIEIIVMYPIQHRAYRPGIDNLLVLLIGGIPIAMPTVLSVTMAIGSHRLSQQGAITKRMTAIEEMA
GMDVLCSDKTGTLTLNKLSVDKNLVEVFEKGVTQDQVILMAARASRIENQDAIDTAIVGMLGDPKE
ARAGIQEVHFLPFNPTDKRTALTYIDGDGKMYRVSKGAPEQILNLAYNKSEIAQKVHTVIDKFAER
GLRSLGVAYQDVPDGRKESPGSPWHFVALLPLFDPPRHDSAETIERALNLGVNVKMITGDQLAIGK
ETGRRLGMGTNMYPSSALLGQNKDESIADLPVDDLIEKADGFAGVFPEHKYEIVKRLQARKHICGM
TGDGVNDAPALKKADIGIAVADATDAARSASDIVLTEPGLSVIISAVLTSRAIFQRMKNYTIYAVS
ITIRIVLGFMLLALIWEFDFPPFMVLIIAILNDGTIMTISKDRVKPSPLPDSWKLAEIFTTGVVLG
GYLAMMTVIFFWAAYKTNFFPRVFHVRSLEKTAQDDFNKMLASAVYLQVSTISQALIFVTRSRSWS
FLERPGFLLVFAFFVAQLIATLIAVYADWAFTSIKGIGWGWAGIVWLYNLVFYFPLDIIKFFIRYA
LSGKAWDLVINQRIAFTRKKHFGKEERELKWAHAQRTLHGLQPPDAKLFPEKAGYNELNQMAEEAK
RRAEIARLRELHTLKGHVESVVKLKGLDIDTIQQSYTV
>HvAHA3
MADKEAGNLEAVLKEVVDLENIPLEEVLDNLRCSREGLTAEQAQQRLQIFGPNKLEEKEESKFLKF
LGFMWNPLSWVMEAAAIMAIALANGGGKPPDWQDFVGIITLLVINSTISFIEENNAGNAAAALMAR
LAPKAKILRDGRWAEEDAAILVPGDVISIKLGDIIPADARLLEGDPLKIDQSALTGESLPATKGPG
DGIYSGSTVKQGEIEAVVIATGVHTFFGKAAHLVDSTNQVGHFQKVLTAIGNFCICSIAVGMFIEI
IVMYPIQHRAYRPGIDNLLVLLIGGIPIAMPTVLSVTMAIGSHRLSQQGAITKRMTAIEEMAGMDV
LCSDKTGTLTLNKLTVDKSLVEVFERGITQDQVILMAARASRTENQDAIDTAIVGMLADPKEARAG
IQEVHFLPFNPTDKRTALTYIDADGKMHRVSKGAPEQILHLAHNTSEIERRVHAVIDKFAERGLRS
LAVAYQEVPDGRKESPGGPWHFAGLMPLFDPPRHDSAETIRRALNLGVNVKMITGDQLAIGKETGR
RLGMGTNMYPSSALLGQKNSDESISALPVDDLIEKADGFAGVFPEHKYEIVKRLQARKHICGMTGD
GVNDAPALKKADIGIAVADATDAARSASDIVLTEPGLSVIISAVLTSRAIFQRMKNYTIYAVSITI
RIVLGFMLLALIWNFDFPPFMVLIIAILNDGTIMTISKDRVKPSPLPDSWKLAEIFTTGVILGGYL
AIMTVIFFWAAYKTNFFPRLFHVESLEKTAQDDFQKLASAIYLQVSTISQALIFVTRSRSWSFAER
PGFLLVFAFFVAQLIATLIAVYADWKFAAIKGIGWGWAGVVWLYNIITYFPLDIIKFLIRYTLSGK
AWDLVIDQRIAFTRKKDFGKEERELKWAHAQRTLHGLQPPDAKMFSDKGGYNELNHMAEEAKRRAE
IARLRELHTLKGHVESVVKLKGLDIETIQQSYTV
>HvAHA4
MAASLEDLRNESIDLEAIPIAEVFQVLKCKPHGLTSDEAASRLQAFGPNKLEEKKESKFLKFLGFM
WNPLSWVMEAAAIMAIVLANGGGKPPDWQDFMGIVTLLIINSTISFIEENNAGNAAAALMASLAPQ
TKVLRDGKWAEQDAAILVPGDIISIKLGDIIPADARLMEGDPLKIDQSALTGESLPVNKLPGDSVY
SGSTCKQGEIEAVVIATGVHTFFGKAAHLVDSTNNVGHFQKVLTAIGNFCIVSIAIGMLVEIVVMY
PIQHRRYRDGIDNLLVLLIGGIPIAMPTVLSVTMAIGSHKLSQQGAITKRMTAIEEMAGMDVLCSD
KTGTLTLNKLTVDKNMIEPFVKDVDKDGVVLYAARASRTENQDAIDASIVGMLADPKEARAGIQEV

HFMPFNPVDKRTAITYVDSDGTWHRVSKGAPEQIIDLCGLREDVRRRVHGIIGKFADRGLRSLAVA
RQSVPERTKEAKGAPWQFLAVLPLFDPPRHDSAETIRRALHLGVNVKMITGDQLAIGKETGRRLGM
GTNMYPSSSLLKDGDSCGLPVDELIEKADGFAGVFPEHKYEIVRRLQEMKHICGMTGDGVNDAPAL
KKADIGIAVADATDAARSASDIVLTEPGLSVIISAVLTSRAIFQRMKNYTIYAVSITIRVVLGFLL
LALIWKFDFAPFMVLIIAILNDGTIMTISKDRVVPSPTPDSWRLKEIFATGIVLGTYQASATVIFF
WAVHSTDFFTNKLHVHPIGGNTEELMAAVYLQVSIISQALIFVTRSRGWSFRERPGALLLGAFLLA
QMVATLIAVYADWPFAKMKGVGWGWAGAIWLFTIVTYFPLDVLKFAIRYFLSGRGWSNVFDGKTAF
AQGVDYGTDKRKAEWAVAQRSLHGLHTSGGGEASSSGVLGGGDDKNDISEIAEQAKRRAEIARLRE
LHTLKGHVDSVVKLKGLDIENINHNYTV
>HvAHA5
MAAAAAEGLERIKNESVDLENIPVEEVLENLQCSKEGLTSKDGQDRMAVFGPNKLEEKKESEILKF
LGFMWNPLSWVMEVAAIMAIALANGGGRPPDWQDFVGIIALLLLNSTISYIEESNAGSSAKALMAN
LAPKTKVLRDGKWSEQDASILVPGDIISIKLGDIVPADARLLLEGDPLKIDQSALTGESLPVTKNP
GDSVYSGSTCKQGEIEAVVIATGVHTFFGKAAHLVDSTNQVGHFQKVLRAIGNFCIGAIAIGMIVE
VIVMYFIQHRRYRDGIDNLLVLLIGGIPIAMPTVLSVTMAIGSHRLSKQGAITKRMTAIEEMAGMD
VLCSDKTGTLTLNKLSVDRNLIEVFAGGVAKDDVLLFAAMASRVENQDAIDAAMVGMLADPKEARA
GIQEMHFLPFNPVDKRTALTYQDLADGTWHRVSKGAPEQILELCNCREDVKNKAHAIIDKYAERGL
RSLAVARQEVPERSKDSSGGPWEFIGLLPLLDPPRHDSAETIKQALNLGVNVKMITGDQLAIAKET
GRRLGMGTNMYPSSALLGQSVDESIVSLPVDELIEKADGFAGVFPEHKYEIVKKLQQMKHICGMTG
DGVNDAPALKKADIGIAVADATDAARSASDIVLTEPGLSVIISAVLTSRAIFQRMKNYTIYAVSIT
IRIVLGFMLIALIWKFDFSPFMILVIAILNDGTIMTISKDRVKPSPHPDSWKLPEIFITGIVYGAY
LAVTTVVFFFAMTSTDFFSEKFNVRSLRGNKDAMMSALYLQVSIISQALIFVTRSRRWCFQERPGL
WLCFAFVVAQIIATVIAVYCNLPFAHIRGIGWGWAGVIWLYSIITFIPLDLFKFAIGYALSGKAWD
TLFENKIAFTNKKDYGKEKRELQWATAQRTLHGLPTANPDSTPQERSNYGELSEMAEQAKRRAEMA
RLRELSTLKGRVESAVRLKGLDVETVDNHHYTV
>HvAHA6
MASMTLEDVKNETVDLETIPVPEVFSHLKCSKQGLSGTEAQNRLAIFGPNKLEEKTENKLLKFLGF
MWNPLSWVMEAAAIMAIVLANGGGKPPDWQDFVGIVTLLFINSTISFIEENNAGNAAAALMAGLAP
KTKCLRDGKWSEMDASFLVPGDIISIKLGDIIPADARLLEGDPLKVDQAALTGESMPVNKHSGQGV
FSGSTVKQGEIEAVVIATGVHTFFGKAAHLVDSTNNVGHFQQVLTAIGNFCIISIAAGMLVEVVVM
YPIQHRAYRDGIDNLLVLLIGGIPIAMPTVLSVTMAIGSHRLSQQGAITKRMTAIEEMAGMDVLCS
DKTGTLTLNKLTVDKTLIEVYGRGIDKDTVLLYAARASRVENQDAIDTCIVGMLADPKEARAGIQE
VHFLPFNPVEKRTAITYIDGNGDWHRISKGAPEQIIELCRMPKEAEKRVHGLIDQYADRGLRSLGV
SYQPVPAKNKDSPGEPWQFVGLLPLFDPPRHDSAETIRRALHLGVNVKMITGDQLAIGKETARRLG
MGTNMYPSTTLLGDKSTEMSGLPIDELIEKADGFAGVFPEHKYEIVKRLQDRKHICGMTGDGVNDA
PALKKADIGIAVDDATDAARSASDIVLTEPGLSVIVSAVLTSRAIFQRMKNYTIYAVSITIRIVVG
FMLVALLWKFDFAPFMVLIIAILNDGTIMTISKDRVKPSPTPDSWKLKEIFATGVVLGTYMALVTV
LFFYLAHDTEFFPETFGVRSIRENEKEMMAALYLQVSIISQALIFVTRSRSWSFVERPGALLVIAF
FVAQLLATCIAVYANWEFCKMQGIGWGWGLSIWAFTVVTYIPLDILKFIIRYALSGRAWNNINNKT
AFTNKNDYGKVEREAQWATAQRTLHGLNQGSNNSDLFADNNGYRELSEIAEQAAKRAEVARLRELH
TLKGHVESVVKLKGLDIETINQSYTV
>HvAHA7
MGGLEEIRNEAVDLENIPIEEVFEQLKCTRQGLTSDEGAQRVEIFGLNKLEEKKESKVLKFLGFMW
NPLSWVMEMAAIMAIALANGGGKPPDWQDFVGIIVLLVINSTISFIEENNAGNAAAALMANLAPKT
KVLRDGRWGEQEASILVPGDIVSIKLGDIVPADARLLEGDPLKIDQSGLTGESLPVTKNPGDEVFS
GSTCKQGEIEAVVIATGVHTFFGKAAHLVDSTNQVGHFQQVLTAIGNFCIISIAVGIVIEIIVMFP
IQRRKYRAGIENLLVLLIGGIPIAMPTVLSVTMAIGSHKLSQQGAITKRMTAIEELAGMDVLCSDK
TGTLTLNKLSVDKNLVEVFAKGVDKEHVLLLAARASRVENQDAIDACMVGMLADPKEARAGIREVH
FLPFNPTDKRTALTYIDAEGNWHRASKGAPEQIITLCNCKEDVKRKVHSVIEKYAERGLRSLAVAR

QEVPEKSKDSAGGPWQFIGLLPLFDPPRHDSAETIRKALVLGVNVKMITGDQLAIGKETGRRLGMG
TNMYPSSALLGQSKDGSLESLPVDELIEKADGFAGVFPEHKYEIVKRLQEKKHIVGMTGDGVNDAP
ALKKADIGIAVDDATDAARSASDIVLTEPGLSVIISAVLTSRCIFQRMKNYTIYAVSITIRIVLGF
LLIALIWKFDFAPFMVLIIAILNDGTIMTISKDRVKPSPLPDSWKLNEIFATGVVLGTYLALMTVV
FFWIIHRTDFFTNKFGVRSIRENETEKMSALYLQVSIVSQALIFVTRSRSWSFVERPGFLLVIAFL
LAQLVATLIAVYANWGFARISGIGWGWAGVIWLFSIVFYFPLDIFKFFIRFVLSGRAWDNLLQNKT
AFTTKENYGKGEREAQWATAQRTLHGLQAPEPASHTLFNDKSSYRELSEIAEQAKRRAEIARLREL
NTLKGHVESVVKLKGLDIDTINQNYTV
>HvAHA8
MDSREEQPPAAEAKQEQQPANGLSDLNNETVDLEHIPVDEVFEHLRCTKEGLTTEAAQQRVEIFGL
NKLEEKNESKILKFLGFMWNPLSWVMEAAAIMAIALAHGGSDLRGKSMGVDYHDFVGIVILLVVNS
TISFIEENNAGNAAAALMARLAPKAKALRDGTWNELDASLLVPGDIISIKLGDIIPADARLLQGDP
LKIDQSALTGESLPVTKHPGGGVYSGSTCKQGEIEAVVIATGIHTFFGKAAHLVESTTHVGHFQKV
LTSIGNFCICSIAIGMTIELIVMAAVQHRPYRQTVDNLLVLLIGGIPIAMPTVLSVTMAIGSHKLA
QQGAITKRMTAIEEMAGMDVLCSDKTGTLTLNKLTVDNNIIEVFTRGYEKSDVVLMAARASRLENQ
DAIDFAIVAMLPDPKEARAGIEEVHFLPFNPTDKRTALTYLDAKGKMHRVSKGAPEQILNLAANKS
EIERKVHQVIDSFAERGLRSLAVAYQEVPEGTKESAGGPWQFIGLLPLFDPPRHDSAETIRRALDL
GVSVKMITGDQLAIGKETGRRLGMGTNMYPSSSLLGDKVDSDIAVLPVDELIEQADGFAGVFPEHK
YEIVKRLQARKHICGMTGDGVNDAPALKVADIGIAVADATDAARGASDIVLTEPGLSVIISAVLTS
RAIFQRMKNYTIYAVSITIRIVLGFLLLACLWKFDFPPMMVLLIAILNDGTIMTISKDRVKPSPCP
DSWKLAEIFATGVVLGTYLAVTTVLFFWAAYKTDFFPRHFNVDTMNMKKKLASAVYLQVSTISQAL
IFVTRSRGWSFTERPGFLLMFAFVLAQLIASLLSALLNWETASIRGIGWGWTGVIWLYNIVIYMLL
DPIKFAVRYGLSGRAWNLVTDRKVAFSNQKNFGKEASQAAWAHQQRTLHGLESAPGREKAASTELG
HMVEETKRRAEITRLRTVHTLKGKVENAAKLKGIDLDDINNQHYTV
>HvAHA9
MDDDGLRKPLLGPESLCTEDIDLGNLPLEEVFEQLSTSRCGLSSADAAERLQLFGANRLEEKRENK
VLKFISFMWNPLSWVMEAAAIMALVLANGGSQGPDWEDFVGIVCLLIINSTISFIEENNAGNAAAS
LMARLAPRTKVLRDGQWQELDASVLVPGDIISIRLGDIVPADARLLEGDPLKIDQSALTGESLPVT
KRTGDLVFTGSTCKHGEIEAVVIATGIRSFFGKAAHLVDSTKVVGHFQKVLTCIGNFCICSIVVGV
FIEVIIMFAVQHRPYREGINNVLVLLIGGIPIAMPTVLSVTLAIGSHRLSQQGAITKRMTAIEEMA
GMDVLCCDKTGTLTLNHLTVDKNLIEVFSGGMDRDMIILLAARASRVDNQDAIDMAIINMLSDPKE
ARANIAEVHFLPFNPVDKRTAITYIDSGGNWFRVSKGAPEQILNLCHNKDDIAEKAQRVVDSFAER
GLRSLAVAYQEVPERSRHGDGGPWVFCGVLPLFDPPRHDSADTIRKALDLGVCVKMITGDHLAIAK
ETGRRLGTGTNMHPSAALFGRRDGDGDGAAAVPVEELVESADGFAGVFPEHKHEIVRLLQASGHVC
GMTGDGVNDAPALKKADIGIAVSDATDAARAAADIVLTEPGLGVIVCAVLTSRAIFQRMKNYTIYA
VCITIRIVVGFVLLASIWEYDFPPFMVLVIAILNDGTIMAISKDRVKPSRRPDSWKLEEIFATGVV
IGTYLALLTVLFYWAVTGTTFFESHFGVRSLKLDAEELSSAVYLQVSITSQALIFVTRSRGISFLD
RPGALLVCAFVVAQLVATLVAVYAAVGFASISGVGWRWAGVIWLYSLVSYLPLDLIKVAVRYALSG
DAWGLLFDRKAAFARRRDYYGEEDHRRGAALSTRRALSDHLLSSRTPRSAVAEQARRRAEIARYKV
SLLAMAVPWKGQ
>HvAHA10
MASLSLEDVRNETVDLSTVTVDEVFKTLKCDKKGLSEAEGENRLKLFGPNKLEEKKESKLLKFLGF
MWNPLSWVMEIAAIMAIALANGGGRPPDWQDFVGIVSLLFINSTISYIEEANAGDAAAALMAGLAP
KTKLLRDGTWDERDAAILVPGDIISIKLGDIIPADARLLDGDALKIDQSALTGESMPVNKYAGQEV
FSGSTVKQGELEAVVIATGVHTFFGKAAHLVDSTNNVGHFQQVLTAIGNFCIISIAAGMLVEIIVM
YPIQHRAYRDGIDNLLVLLIGGIPIAMPTVLSVTMAIGSHRLSQQGAITKRMTAIEEMAGMDVLCS
DKTGTLTLNKLTVDKTLIEVCSRGVDKDMVLLYAARASRVENQDAIDTCIVNMLADPKEARAGIKE
VHFLPFNPVDKRTAITYIDGNGDWHRVSKGAPEQIIELCKMAPDAEKKVHALIDSYADRGLRSLGV
SYQQVPEKNKDSAGEPWQFIGLLPLFDPPRHDSAETIRRALHLGVNVKMITGDQLAIGKETGRRLG

MGTNMYPSTALLGDKNTQVDGLPIDELIEKADGFAGVFPEHKYEIVKRLQDKKHIVGMTGDGVNDA
PALKKADIGIAVDDATDAARSASDIVLTEPGLSVIVSAVLTSRAIFQRMKNYTIYAVSITIRIVMG
FTLVALIWKFDFAPFMVLIIAVLNDGTIMTISKDRVKPSPKPDSWKLDEIFATGVVLGTYMALVTV
VFFYLAHDTDFFTETFGVPPIRDNDRQLMAALYLQVSIISQALIFVTRSRSWSFVERPGFLLLFAF
FAAQLVATAIAVYANWDFCDMQGIGWAWGGAIWVFTLVTYVPLDVLKFIIRYSLSGKGWDNVQNKT
AFTNKKDYGRGEREALWAKEQRTLHGLSQPAASDLLSSNEELSDIAEQAAKRAEIARLRELHTLKG
HVESVVKQKGIDIDAIPQNYTV
>HvALA1
MSTVDPLLLSSSGSADSPSKHTAPARSSVGSCLCRADSCSSSVYEDCDTASVNFVDEGDAIPRHCP
EESDVSRVAERFQSADSHFFHRLSVECSQKERQRKISWGGVMEMQRSPSSLEVGVVSSSQEKPNRP
PRGRNKSSHFEDLFSSEQEHDPRLIYINDPDRTNDRYEFTGNEIRTSKYTLITFLPKNLFIQFHRL
AYVYFLVIAALNQLPPLAVFGRTASLFPLLFVLFVTAIKDGYEDWRRHRSDRNENNREALVLQHGD
FRSKKWKHICAGEVVKIHSNETMPCDMVLLSTSDPNGIAYIQTMNLDGESNLKTRYARQETVTMIS
NSSYLGLIKCEQPNRNIYEFTATMELNSQRIPLGQSNIVLRGCQLKNTEWIIGVVVYAGQETKAML
NSTISRSKSSNLESYMNRETLWLSAFLLITCSVVATGMGVWLFKNTKNLDALPYYRRKYFTFGREN
RKDFEFYGLALEIFFSFLSSVIIFQIMIPISLYITMELVRVGQSYFMIGDTRMYDSSSGSRFQCRS
LNINEDLGQIRYIFSDKTGTLTQNKMEFQQASIYGRNYGSSLQVTSDSSHEITIAESSRQHDRKPK
SEINVDALLLALLKQPLFGEERLAAHDFFLTLAACNTVIPVSTGGSPDLTNEVSEVGAIDYQGESP
DEQALVIAASAYGYKLVERTTGHIVIDVQGERIRLDVLGLHEFDSVRKRMSVVVRFPDDTVKVLVK
GADTSMLNILKTRNHDGLFDSLHAKTIETTENHLSSYSSEGLRTLVIGSKYLSNEEFSEWQERYEE
ASTSMTERSAKLRQAAALVECDLTLLGATGIEDKLQDGVPEAIESLRQAGIKVWVLTGDKQETAIS
IGLSCRLLTQGMHSIIINGSSEIECRRLLADAKAKFGIKSADLGKQDVEDLHNGDVSKLRSSNGQA
SESGIQNFQLTGVIANDKSVNIEESPNFDDAELALIIDGNSLVYILEKDLESELFDLATSCKVVIC
CRVAPLQKAGIVDLIKSRTSDMTLAIGDGANDVSMIQMADVGVGICGQEGRQAVMASDFAMGQFRF
LKRLLLVHGHWNYQRMAYMILYNFYRNAVFVLMLFWYILHTAYSATLALTDWSSVFYSLIYTSVPT
VVVGILDKDLSHNTLLYYPRLYEAGLRNEGYNMTLFWITMLDTLWQSLVLFYVPFFTYSISTMDIW
SMGSLWTIAVVILVNIHLAMDIQRWVLITHLAIWGSIAATFLCMVLIDSIPIFPNYGTLYNMAASR
TYWLSVCLIIVLGLLPRFLCKVIYETFWPSDIQIAREAELLKKLPQQCRSRPESDIS
>HvALA2
MTSERPLIDAASPHLPAAASQLPPSQPEPPVRADHLGLSVDVPDPFRSSRRDHPDPSASERELHEG
GEYRAVAVGEPSPEFDGNSVRTAKYSALTFLPRNLFEQFRRLSYVYFLAITVLNQLPQVAVFGRGA
SVLPLAFVLFVTAVKDAYEDIRRHRSDRRENNRLAVVLAPQTAGEFLPKKWKHIRVGDVVRFASNE
TLPADMVLLATSDPTGLAHVQTVNLDGETNLKTRYAKQETQLRFSQDGHVAGILHCERPNRNIYGF
QANLEIDGKRVSLGPSNIVLRGCELKNTTWAIGVVVYAGKETKVMLNNSGPPSKRSRLETQLNRET
VILSIMLIGMCITASVLAGIWLLNHQRELEFTQFFREKDYTTGKNYNYYGIGMQIFVTFLMAVIVY
QVIIPISLYISMELVRLGQAYFMGADNDLYDGSSRSRFQCRALNINEDLGQIKYVFSDKTGTLTEN
KMEFVCASIHGVDYSSGKHACGYSVVVDDLLWTPKMAVRTDPQLLKLLSNHSSNGEAKFVLEFFLA
LAACNTIVPLVLDTRDPRQKLIDYQGESPDEQALAYAAASYGIVLVERTSGYVVIDVLGDRQRYDV
LGLHEFDSDRKRMSVIVGCPDKTVKLYVKGADSSMFGIINSLELDNVRATEAHLHKYSSLGLRTLV
VGMRELSQPEFEEWQLAYEKASTAVLGRGNLLRSIAANVECNIHILGASGIEDKLQDGVPEAIESL
RQAGMKVWILTGDKQETAISIGYSCKLLTNDMTQIVINNNSKESCKKSLEEALARTKEHRVASSIG
SPNPVFATESSGTVLALIVDGNSLVYILETELQEELFKVATECSAVLCCRVAPLQKAGIVALIKNR
TDDMTLAIGDGANDVSMIQMADVGVGISGQEGGQAVMASDFSMGQFRFLVPLLLVHGHWNYQRMGY
MILYNFYKNATFVLVLFWYVLYTSFTLTTAITEWSSLLYTVLYTSLPTIIVGILDKDLSKSTLLAY
PKLYGSGQRNEKYNLNLFVLNMLEALWQSLIVFYIPYFAYRQSTIGMSSLGDLWALASVIVVNMQL
AMDIIQWNWIIHAFIWGTIAATVICLFVIDSIWVLPGYGVIYHIMGQGLFWLLLLIIVVTAMVPHF
AIKAFMEHFVPTDIQIGQEIEKFKALNQVNRSEIPMRTFS
>HvALA3

MAEDHGSSRHMSSMSMSHKELGDDDARVVHVGDADRTNERLEFAGNAVRTAKYSPLTFLPRNLFEQ
FHRLAYIYFLVIAVLNQLPQLAVFGRGASVMPLALVLAVTAVKDAYEDWRRHRSDRAENNRLAAVL
SPGAGAQFVPTEWKHVRVGDVVRVGANESPPADMVLLATSDTTGVAYVQTLNLDGESNLKTRYAKQ
ETLTTPLEHLAGTVVRCERPNRNIYGFQANLELQGESRRIPLGPSNILLRGCDLKNTSWAVGVVVY
AGRETKAMLNNAGTPTKRSRVETQMNRETLFLSGILIVLCSAVATLTGVWLRTHQADLELAQFFHK
KDYLKVGKDGNENYNYYGIAAQIVFNFLMAVIVFQIMIPISLYISMELVRLGQAYFMIRDAKLYDA
STDSRFQCRALNINEDLGQVKCVFSDKTGTLTQNKMEFRCASIDGVDYSDVARQRPVEGEPAWVPK
VPVNVDREVMELVRNGGATEQGMNAGEFFLALATCNTIVPLIVDGPDPKKKVIDYQGESPDEQALV
SAAAAYGFVLVERSSGHIVIDVLGQKQRFDVLGLHEFDSDRKRMSVIIGCPDKTVKLFVKGADSSM
FGIIDKTLNPDVVQATEKHLHSYSSVGLRTLVIGVRELSQAEFQEWQMAYEKASTALLGRGNLLRS
VAANIERNMRLLGASGVEDKLQDGVPEAIEKLREAGIKVWVLTGDKQETAISIGYSCKLLTRDMTQ
IVINSNSRESCRKSLDDAISMVNKLRSLSSDSQSRVPLALIIDGNSLVYIFDTDREEKLFEVAIAC
DVVLCCRVAPLQKAGIVDLIKKRTSDMTLAIGDGANDVSMIQMADVGIGISGQEGRQAVMASDFAM
GQFRFLVPLLLVHGHWNYQRMSYMILYNFYRNATFVFVLFWYVLYTGYTLSTAINEWSSVLYSVVY
TSAPTVIVAILDKDLSRRTLLKYPQLYGAGQREESYNLRLFIFIMVDSVWQSVAVFFIPYLAYKNS
AIDSGSLGDLWTLSVVILVNIHLAMDVIRWTWVTHAAIWGSIVATWICVIIIDSIPTLPGFWAIYK
VMGTALFWALLLAVIVVGMIPHFAAKAIREHFMPNDIQIAREMEKSQDSRDVYHPEVQMSTSTRA
>HvALA4
MASSGGGRGDTDDGHGRRGRRRRRKVLLSKLYTFAACARRPSAVDDEGSRIGGPGFSRVVHANDAA
AAADAAAAGGYRSNYVSTTKYNAVTFVPKSLFEQFRRVANIYFLVVACLSYTPIAPFRGATAVGPL
VLVLLVTMIKEAIEDWRRKQQDIEVNNRKTKVFQDGAFRHTKWTKLRVGDVVKVEKDEFFPADLVL
LSSSYDDAICYVETMNLDGETNLKLKQSLEVTSRLQDDDSFAGFEAVIRCEDPNANLYSFVGNIEI
EEQQQQYPLSPQQLLLRDSKLRNTEYVYGVVVFTGHDTKVMQNATSAPSKRSKIEKKMDEAIYVLM
SMLVLISVIGSVVFGLATKHDLVDGRMKRWYLRPDEPDKLYDPNNPAVSAALHFFTAMILYGYFIP
ISLYVSIELVKLLQALFINSDIHMYHEESDTPAHARTSNLNEELGQVYTILTDKTGTLTCNSMEFI
KCSIAGTAYGRGITEVERAMAKRNGSPMIADIEDGVEAFHQSEGRAAVKGFNFRDERVMDGNWVHQ
EHSGAIEMFFRLLAICHTCIPEVDEVTGKISYEAESPDEAAFVVAAHELGFTFYQRTQAGVYLHEL
DSSSGEQVDRFYKVLHVLEFSSARKRMSVIVQDEEGKTFIFSKGADSIMYERLSNSESAYGEATQK
HINDYADAGLRTLVLAYRPLEEVEYAKFERKFTAAKNSVSADRDELIDEAADLVERDLILLGATAV
EDKLQKGVPDCIDKLAKAGIKIWVLTGDKMETAINIGYACSLLRQGMKQITITLDTPDIIALEKGG
DKGAINKASKVSVVQQINEGKKLINASGNESFALIIDGKSLTYALKDDTKATFLDLAIACGSVICC
RSSPKQKALVTRLVKTGTGKVTLAIGDGANDVGMIQEADIGVGISGAEGMQAVMASDVSIAQFRFL
ERLLLVHGHWCYSRISSMVCYFLYKNITFGVTLFLYESLTTFSGQTLYNDWSMSLYNVLFTSLPVI
AMGVFDQDVSARFCLKYPMLYQEGPQNLLFRWSRLLGWMLHGVGSAVIIFFLTIASLKHQAFRKDG
EVIDLSILGATAYTCVVWAVNMQMAITVNYFTLVQHICIWSGIFLWYLFLIIYGAITPSFSTTFFM
VFSEALGGAPAYWVVTLLVAVAALIPYFTLAVVKTWFFPDYHNKIQWLQHTAKHEDPEEELGVVLR
QFSVRSTGVGVSARRDAKLVRTNSKIFHADSSSQSQATIELT
>HvALA5
MALGRRRRKLEKLKLSALYSFALCAKGATEDHSKIGTAGFSRVVYVNDPDRHEGEGFRYPKNEVST
TKYSLVTFVPKSLFEQFRRVANFYFLVSGILTLTPLAPYSAVSALLPLSVVITATMLKEGVEDWRR
KKQDIELNNRIVKVHRGNGSFEETKWKYIKIGDVIKVEKDNFFPADLILLSSNYPDGICYVETMNL
DGETNLKIKQALEVTLDLQEDGSFTSLRQIIKCEDPNANLYSFIGTMDYKGMQHPLSPQQLLLRDS
KLRNTDYIYGAVIFTGHDTKVMQNATEPPSKRSKIEKKMDNIIYLLLCSLLGIALLGSVFFGIWTK
DDLRNGEPKRWYLRPDDSTVFYDPKRAPLASFCHLLTALMLYNYFIPISLYISIEMVKILQAVFIN
QDIEMYDEESDKPTHARTSNLNEELGQVDTILSDKTGTLTCNMMEFIKCSIAGTAYGQSVTEVEKA
MALRKGVPLGDEIVGGEHKEKQIEESPHVKGFNLKDPRIMDGNWVHEPNKDVIRDFFRLLAICHTC
IPEVDETNKVTYEAESPDEAAFVIAARELGFEFYKRTQTSIVIRERNPNQNVEDYQYRKYELLNVL
EFSSSRRRMSVIVKEPEGRVLLFSKGADSVMFRRLAPDGRKFEEETKKHINEYSDSGLRTLVLAYR
VLDEKEYQSFAEKFRTAKISGSADRDEQIGEAADSIERDLILLGATAVEDKLQKGVPECIDKLAQA

GIKIWVLTGDKMETAINIGFACSLLRQGMIQIIITLEAPDIIALEKNGDKDSIAKASKQSVMDQIE
DGIKQVPALGQSGMESFALIIDGKSLTYALEDDVKFKFLDLAVKCASVICCRSSPKQKALVTRLVK
HSHKVTLAIGDGANDVGMLQEADIGVGISGVEGMQAVMASDIAIAQFRFLERLLLVHGHWCYRRIS
VMICYFFYKNVTFGVTIFLYEAFASFSGKPAYNDWFLSLYNVFFTSLPVIALGVFDQDVSSRLCLQ
YPELYQEGVQNVLFSWRRILGWMFNGVVNAILIFFFCTTALKDQAFRQDGQVAGLDALGAAMYTCV
VWVVNCQMALSVNYFTIIQHIFIWGSIAVWYIFLMVYGSIDPKYSKTAYMVFIEQLAPALSYWLVT
LFVVTATLVPYFCYAAIQIRFFPMFHNKIQWKRYLGKAEDPEVARQLSSRHRSSSHPRMVGISARR
DGKAMQVKKGTDVEVEG
>HvALA7
MARARKRDRLRWSKLYTFSCFRQPQTDEAAGPAAVSGSPVGGPGFSRIVHCNNSILHRRKPLKYPT
NYISTTKYNVLTFLPKAIFEQFRRVANLYFLLTAILSLTPVCPFSPVSMIAPLAFVVGLSMIKEAL
EDWRRFMQDMKVNNRKVSAHKGDGEFEFRHWEDLCVGDVVRVEKDQFFPADLLLLSSSYEDGICYV
ETMNLDGETNLKLKRSLEVTLPLEEDETFKDFRGVVRCEDPNASLYTFIGNLEYERQIYSLDPSQI
LLRDSKLRNTAFIYAVVIFTGHDSKVMQNSTESPSKRSRIEKKMDLIIYILFTVLVLISLISSIGF
AVRIKLDLPRWWYLQPQNSNKLDDPTRPALSGIFHLITALILYGYLIPISLYVSIEVVKVAQAHFI
NQDIHMFDEETGNTAQARTSNLNEELGQVHTILSDKTGTLTCNQMDFLKCSIAGVSYGVCASEVER
AAAKQMASGAADQDIPVEDVWESNEDEIQLVEGVTFSVGKTQKTSIKGFSFEDDRLMQGHWTKEPN
SNMILMFFRILAVCHTAIPEVNEATGALTYEAESPDEGAFLVAAREFGFEFFKRTQASVFLKEKYT
SSNGTTEREFKILNLLEFNSKRKRMTVIMRDEDNRIVLLCKGADTIIFDRLAKNGRLYEPDTTKHL
NEYGEAGLRTLALSYRMLEESEYESWNAEFLKAKTSIGPDRELQLERVADLIEKELILVGATAVED
KLQTGVPQCIDRLAQAGLKIWVLTGDKMETAINIGYACSLLRQGMKQISLSTTAGDQVAQDAQKAA
KESLMLQIANGSQMVKLEKDPDAAFALVIDGKALTFALEDDMKHMFLNLAIECASVICCRVSPRQK
ALVTRLVKEGIGQTTLAIGDGANDVGMIQEADIGVGISGVEGMQAVMASDFSISQFRFLERLLVVH
GHWCYKRIAQMICYFFYKNITFGLTIFYFEAFAGFSGQSVYDDWFMLLFNVVLTSLPVISLGVFEQ
DVSAEICLQFPALYQQGPNNLFFDWYRILGWMANGLYSSLAIFFLNICIFYDQAIRSGGQTADMAS
VGTTMFSCIIWAVNIQIALTMSHFTWIQHLFVWGSIGTWYVFIITYGMALKSRDNFQIMTEVLGPA
PIYWAATLLVTAACNIPYLIHISYQRSCNPLDHHVIQEIKYLRKDVEDETMWKRERSKARQRTKIG
FTARVDAKIKQIKGRLHKKSPSLTIHTVA
>HvALA8
MVRVATARLGGELSPRAGPSQPGRAESSRTARLGGGGASLRRQPQPQAPTVRTIYCNDREANAPVA
YKGNSVSTTKYSVLTFLPKGLFEQFRRVANLYFLMISILSTTPISPVHPVTNVVPLSLVLLVSLIK
EAFEDWKRFQNDMSINNAHVDVLQGQKWESSPWKRLQVGDIVRIKQDSYFPADLLFLSSTNADGVC
YIETANLDGETNLKIRKALEKTWDYVLPEKASEFKGEIQCEQPNNSLYTFTGNLIVDKQTIPISPN
QILLRGCSLRNTEYIVAVVIFTGHETKVMMNSMNVPSKRSTLEKKLDKLILALFATLFTMCVIGAI
GSGVFINEKYFYLGLRGHVEDQFNPKNRLVVTILTMFTLITLYSTIIPISLYVSIEMIKFIQCAQF
INNDVNMYHAESNTPALARTSNLNEELGQVEYIFSDKTGTLTRNLMEFFKCSIGGEIYGTGITEIE
KGGAERAGVRIDDDEGKRSAVAVHEKGFNFDDARIMRGAWRNEPNPEACMEFFRCLAICHTVLPEG
EETPEKITYQAASPDEAALVAAAKNFGFFFYRRTPTTVMVRESHVDRMGSMQDAAYEILNVLEFNS
TRKRQSVVCRFPNGKLVLYCKGADNVIYERLADGNYDIKKTSREHLEQFGSAGLRTLCLAYRDLSL
DQYKSWNEKFVQAKSSLRDRDKKLDEVAELIEKDLILIGCTAIEDKLQEGVPACIETLSAAGIKIW
VLTGDKMETAINIAYACSLVNNDTKQFIISSETDAIREAEDRGDPVEIARVIKDSVKQSLRSYLEE
AHRSLNNTPERKLAFIIDGRCLMYALDPALRVNLLGLSLICHSVVCCRVSPLQKAQVTSLVRKGAR
KITLSIGDGANDVSMIQAAHVGIGISGQEGMQAVMASDFAIAQFRYLTDLLLVHGRWSYLRLCKVI
TYFFYKNLTFTLTQFWFTFQTGFSGQRFYDDWFQSLYNVIFTALPVIMVGLFDKDVSASLSKKYPQ
LYQEGIRNTFFKWKVIAVWGFFAFYQSIVFYYFTAAASQHGHGSSGKILGQWDVSTMAFTCVVVTV
NLRLLMSCNSITRWHYFSVAGSIAAWFLFIFIYSAIMTSFDRQENVYFVIYVLMSTFFFYLTLILV
PVIALFGDFLYLSLQRWLFPYDYQVVQEMHKDDPHEYSMIRLPERSHLSPEEARSYAISMLPRENS
KHTGFAFDSPGYESFFASQQGVCVPHKSWDVARRASMKQQRQQPQRTGRS
>HvALA9

MKRFVYINDESYQNDYCDNQISNTKYTLWNFLPKNLWEQFRRFMNQYFLLIACLQLWSLITPVNPA
STWGPLIVIFAVSATKEAWDDYNRYISDKQANEKKVWIVKNGARKHIQAQDIRVGNIVWIRENEEV
PCDLVLTGTSEPQGVCHVETAALDGEIDLKTRVIPTTCVGLDSEQLHKIKGVIECPIPDKDIRRFD
ANIRLFPPFIDNDICPLTINNTLLQSCYLRNTEWACGVAVYTGNETKLGMSRGVPEPKLTAMDAMI
DKLTAAIFLFQLAVVVVLGSAGNVWKDTEARKQWYVKYDDDEPWYQILVIPLRFELLCSIMIPISI
KVSLDFVKSMYAKFIDWDEEMYDQETDTPAHAANTAISEDLGQVEYILTDKTGTLTENKMIFRRCC
IAGTLYGNESGDALKDIELLNAVANNSPHVIKFLTVMALCNTVIPIKRLYLCLPGGTISYKAQSQD
EDALVNAASNLHVVLVSKNGNDAEIHFNRRVIQYEILDILEFTSDRKRMSVVISDSQSGKIFLLSK
GADEAMLPLAYSGQQIKTFVDAVDKYAQLGLRTLCLGWRELSLEEYLEWSRLFKEANSALVDREWK
VAEVCQKLEHTLDILGISAIEDRLQDGVPETIEILRQSGINFWMLTGDKQSTAIQIALLCNLISSE
PKGQLLYINGKTEDEVARSLERVLLTMRITSSEPKELAFVVDGWALEIILTRYKEAFTELAVLSKT
AICCRVTPSQKAQLVKLLKSCDYRTLAIGDGGNDVRMIQQAHIGVGISGREGLQAARAADYSIGKF
RFLKRLILVHGRYSYNRTAFLSQYSFYKSLLICFIQILFSFVSGIAGTSLFNSVSLMAYNVFYTSI
PVLTTVLDKDLSEKTVTQNPEILLYCQAGRLLNPSTFAGWFGRSLYHAIVVFLITIHAYANEKSEM
EELSMVALSGSIWLQAFVVTLEMSSFTFLQFLAIWGNFAAFYVINLCISTIPTAGMYTIMFRLCRQ
PSYWITMLLISGVGMGPVLALKYFRYTYSPSAINILQKAERSRGPMYTLVNLESQLRLEKDNSTNS
ILTTPVKNKSSVYEPLLSDSPMASRRSLASSSFDIFQPAQSRVPAAYPRNIKTS
>HvALA10
MQRFVYINDESCRDSYCDNRVSNTKYTLWNFLPKNLLEQFRRFMNQYFLLIACLQLWPTITPVSPA
TTWGPLAIIFIVSASKEAWDDYNRDLSDKKANERTIWVVKDGIRRQIKAKEIHVGNIVWLHENDEI
PCDLVLIGTSDPQGICYVETAALDGETDLKTRIIPSICADLSSEQLGKIKGVVECPNPDNDIRRFD
ANMRLFPPIIDNEKCPLTINNTLLQSCYLRYTEWACGVAIYTGNETKSGMSRGTAEPKLTAADSMI
DKLTVAIFVFQIAVVLLLGLAGNIWKDRHGRKQWYLMYPAERLWYDFLVIPLRFELLCSIMIPISI
KVTLDLAKGVYAKFIDCDDQMFDPETNTPAHSANTAISEDLGQVEYILSDKTGTLTENRMIFRRCC
ISGALYGDNTGDALKDARLLNAVSSNDPDVVKFLMVMALCNTVVPIKSNDGTISYKAQSQDEEALV
NAASNLNMVLSSKDSGGIVEICFNGSKFYYEVLDVLEFTSDRKRMSIVVKEVKSGKFLLLSKGADE
AMFPRSCPGQQTRTYLEAVEMYSHLGLRTLCLGCRDLEEDEYKEWSKKFQDASCSLDNREHRIAEV
CNSLEQSIHILGITAIEDRLQDGVPETIKLLRKAGINVWMLTGDKQTTAIQIGLLCNLITPESNGQ
LLSINGKTEDDILQSLERALAIMKTASERKDLAFVLDGWALEIILKRSLDSFTKLAMMSRTAICCR
MTPLQKAQVVGLLKSSGSLTLAIGDGGNDVRMIQEANVGVGISGREGLQAARAADYSIGKFKFLRR
LILVHGRYSYNRTAFISQYSFYKSLLICFIQILFSFSSGLSGTSMFNSISLMAYNVFYTSLPVMTI
IFDKDISETTVLRYPQILLYSQAGRLLNRSTFAEWFGRSLYHAFVVFVITINAYADEKSDMEELSM
VALSGCIWLQAFVVTLDTNSFTCPQITLIWGNFVAFYMINLILSAVPTLQMHTVMWHLCNQPSYWI
TMALIVAVGMGPVLALRYLRNVYRPSAIDVLQQIEQADGHAQASGNLESSTGTYLDYLLTDLRRNK
SSIHQPLLSDSVVSSR
>HvECA1
MGKGGQDEAVRPDGSGSPGPDPDVPVFPFWARTPSECLAELGVSADRGLSSDDAAARLHKYGPNEL
ERHAPPSVWKLVLEQFNDTLVRILLAAAVVSFVLALYDGAEGGEVRATAFVEPLVIFLILIVNAVV
GVWQESNAEKALEALKEIQSEHATVKRDGRWKHGLPARDLVIGDIVELRVGDKVPADMRVLQLISS
TLRVEQGSLTGETSSVNKTSHKIHLEDTDIQGKECMVFAGTTIVNGSAVCVVTGTGMSTEIGKIHS
QIQEASQEEDDTPLKKKLNEFGEALTAIIGVICILVWLINVKYFLTWEYVDGWPTNFKFSFEKCTY
YFEIAVALAVAAIPEGLPAVITTCLALGTRKMAQKNALVRKLPSVETLGCTTVICSDKTGTLTTNQ
MSAVRLVAIGRWPDTLRNFKVDGTTYDPSDGKIHDWPTLNMDDNLQMIAKIAALCNDASIAHSEHQ
YVATGMPTEAALKVLVEKMGLPGGYTPSLDSSDLLRCCQWWNNDAKRVGTLEFDRTRKSMGVIVKK
AETGKNLLLVKGAVENLLERSAYIQLLDGSVVLLDEGAKALVLSTLREMSGSALRCLGFAYKEDLA
DFATYDGEEHPAHKYLLDPAYYSSIESNLIFCGFVGLRDPPREEVHKAIEDCRAAGIRVMVITGDN
KETAEAICREIGVFGPSENISSKSFAGKEFMALPDKKKLLRQTGGLLFSRAEPKHKQEIVRLLKED
GEVVAMTGDGVNDAPALKLADIGIAMGITGTEVAKEASDMVLADDNFSTIVSAVGEGRSIYNNMKA
FIRYMISSNIGEVASIFLTSALGIPEGLIPVQLLWVNLVTDGPPATALGFNPPDKDIMKKPPRRSD

DSLITPWILFRYMVIGLYVGIATVGIFIIWYTHGSFLGIDLASDGHTLVSYSQLSNWGQCPSWEGF
NVSSFTAGARTFNFDENPCDYFQGGKIKATTLSLSVLVSIEMFNSLNALSEDGSLLSMPPWVNPWL
LLAMSVSFGLHFLILYVPFLTQIFGIVPLSFNEWLLVVAVAFPVVLIDEVLKFVGRCLTARARKQL
GKRKEE
>HvECA2
MGEAGHDAPAAVKEGFPAWARSVEECEKRFGTDRERGLTSGEAAARLRAHGPNELLEHPGPSVLQL
VAQQFEDTLVRILLAAAAVSFALALSSSAGALTLSAFVEPLVIFLILVVNAAVGVWQETNAEKALE
ALRQIQSDHAAVLRDGEWAPALPARDLVPGDVVMLRVGDKVPADMRVLRLVSSTLRVEQGSLTGET
NSVNKTAHAVPAEDADIQAKECMVFAGTTVVNGSAVCLVVHTGMATEIGKIHSQIHEASQEDDDTP
LKKKLNEFGEALTKIIGLICILVWLINVKYFLTFELDGWVPRNIRFSFEKCTYYFEIAVALAVAAI
PEGLPAVITTCLALGTRKMAAKNALVRKLPSVETLGCTTVICSDKTGTLTTNQMSVSKLVAIGDAP
GKVRSFKVDGTSYDPRDGKIYDWPAGRMDANLEMIAKVAAVCNDASVSHSSNQYVSTGMPTEAALK
VLVEKMGVPEGKNGLSVDPSTLGCCRWWSNAAKRIATLEFDRMRKSMGIIATSKSGGNTLLVKGAV
ETLLERSSHIQLQDGSVVPLDEKSRKAVLASLHELSTKALRCLGFAYKEDLGEFATYDGEYHPAHK
LLLDPANYAAIETDLIFVGLAGLRDPPREEVFDAIEDCRAAGIRVMVITGDNKETAEAICHEIGVF
SPDEDITLKSFTGREFMALEDKKTLLRRKGGLLFSRAEPRHKQEIVRLLKEDGEVVAMTGDGVNDA
PALKLADIGIAMGITGTEVAKEASDMVLADDNFSTIVAAVGEGRSIYNNMKAFIRYMISSNIGEVA
CIFLTSALGIPEGLIPVQLLWVNLVTDGPPATALGFNPPDKDIMKKPPRRSDDSLITPWILFRYLV
IGLYVGVATVGIFVIWYTHGSFMGIDLTGDGHTLVSYSQLSNWGQCSTWDNFTVAPFTAGARTFTF
DDNPCDYFQAGKVKATTLSLSVLVAIEMFNSLNALSEDTSLLRMPPWVNPWLLLAMSVSFGLHFLI
LYVPFLAQVFGIVPLSLNEWLLVLLVALPVVLIDEVLKFVGRCMTASGPKRRLKKQKGE
>HvECA3
MEDAYAKSVAEVLEAFGVDRTKGLSDSQVEQHALRYGKNVLPQEESTPFWKLVLKQFDDLLVKILI
AAAVVSFLLARLNGETGLTAFLEPSVIFMILAANAAVGVITETNAEKALEELRAYQADVATVLRNG
CFSILPATELVPGDIVEVGVGCKVPADMRMVEMLSHQLRVDQAILTGESCSVAKELDSTSAMNAVY
QDKTNILFSGTVVVAGRARAVVIGVGSNTAMGSIRDAMLRTEDEATPLKKKLDEFGTFLAKVIAGI
CILVWVVNIGHFRDPSHGGFLRGAIHYFKVAVALAVAAIPEGLPAVVTTCLALGTKRMARLNAIVR
SLPSVETLGCTTVICSDKTGTLTTNMMSVSKVCVVRSVHQRPITDEYSISGTTFAPDGFIYDASEN
QLEFPPQSPCLLHIAMCSALCNESTLQYNPDKKSYEKIGESTEVALRVLVEKVGLPGFDSMPSALN
MLTKHERASYCNHYWENQFRKISVLDFSRDRKMMSVLCSRKQQEIMFSKGAPESVMARCTHILCND
DGSSVPLTMDIRNELEAKFQSFAGKDTLRCLALALKRMPEGQQSLSYDDEANLTFIGLVGMLDPPR
DEVRSAIHSCMSAGIRVIVVTGDNKSTAESLCRQIGAFEHLDDFTGYSYTASEFEGLPPLERANAL
RRMVLFSRVEPSHKKMLVEALQSHNEVVAMTGDGVNDAPALKKADIGIAMGSGTAVAKSASDMVLA
DDNFATIVAAVAEGRAIYNNTKQFIRYMISSNIGEVVCIFVAAVLGMPDTLVPVQLLWVNLVTDGL
PATAIGFNKPDGNIMAVKPRKVNEAVVSGWLFFRYLVIGAYVGLATIAGFVWWFVYSEDGPRLPYS
ELVNFDSCSTRQTSYPCSIFEDRHPSTVSMTVLVVVEMFNALNNLSENQSLLVIHPWSNLWLVGSI
ILTMLLHVAVLYTEPLSSLFSVSPLTLAEWKVVLYLSFPVILIDEVLKFFSRRPRAWSFPLRLWRR
EMLPKEARDN
>HvHMA1
MQFLTTSAACSSSAPPLPRPAHLLRVSRPPPFPHLRRRRAPHPPSVSLSLAPKPSLLAASRRSLLF
TPRAHGDHHHHHHHHHHGHGHGHGHHGHGDDGVEVRGGGGGAAVMRMARTIGWADVADALREHLQL
CCISLGLLLTAAVCPHVPLLNSVGRLPAALIAIAFPLVGVSAALDALVDIADGKINIHVLMALAAF
ASIFMGNSLEGGLLLAMFNLAHIAEEYFTSKSMYDVRELKENHPEFALLLETSGDESAHFSNLNYA
KVPVHDLEVGSHILVRAGEAVPVDGEVYQGSSTITIEHLTGETKPVERTVGDAIPGGARNLEGMMI
VKVTKSWEDSTLNRIVQLTEEGQLNKPKLQRWLDEFGEHYSKVVVALSLAVALLGPFLFKWPFFGN
SVCRGSIYRGLGLMVAASPCALAVAPLAYATAISSLASKGILLKGGHVLDALSSCQSIAFDKTGTL
TTGKLMCKAIEPIHGHLDASNGVDPSCCTPNCESEALAVAAAMEKGTTHPIGRAVLKHSVGRDLPV
VAVESFESLPGRGVVATLSGIKARDNESEFAKASIGSVEYISSLYRSYGESEQIKEAVKCSAFGPE
FVQAALSVDKKVTLFHFEDEPRTGVCEVIYTLREKAKLRIMMLTGDHESSAQRVAKAVCIEEVHFS

LKPEDKLNKVKAVSREGGGGLIMVGDGINDAPALAAATVGIVLAQRASATAVAVADVLLLQDNLCV
VPFCIAKARQTTSLVKQSVALALTCIVFAALPSVLGFLPLWLTVLLHEGGTLLVCLNSIRALNPPT
WSWADDIRQLVHSLKNYVSAKLNSSSSDCSASTVPL
>HvHMA2
MAAPAPAAAGKLEKSYFDVLGICCPSEVPLVEKLLEPLAGVHKVTVVVPSRTVIVLHDAAAISQAQ
IVRALNGARLEASVRAYGGAGQSKVTNKWPSPYVLVCGVLLVVSLFEHFWRPLKWFAVAGAAAGLP
PIILRSVAALRRRTMDVNILMLIAVAGAIALKDYSEAGFIVFLFTIAEWLETRACGKATAGMSSLM
SMAPQNAVLAETGQVVATQDVKINTVIAVKAGEIVPIDGVVVDGRSEVDESTLTGESFPVSKQADS
QVWAGTLNIDGYIAVRTTAMADNSAVAKMARLVEEAQNNRSSTQRLIDTCAKYYTPAVIFMSAAVA
VIPVCLKARNLKHWFELALVLLVSACPCALVLSTPVATFCALLRAARTGLLIKGGDVLESLASIKV
AAFDKTGTITRGEFSVEEFQTVGERVSKQQLLYWVSSIESRSSHPMAAALVGYAQSNSVEPKSENV
AEFQMYPGEGIYGEIGGEGVYVGNKRILARASCQIVPDIVEHMKGVTIGYVACNKELIGVFSLSDS
CRTGSAEAIKELRSLGIKSVMLTGDSTAAATHAQNQLGNILAEVHAELLPEDKVRIVDELKARDGP
TLMIGDGMNDAPALAKADVGVSMGVSGSAVAMETSHITLMSNDIRRIPKAIKLARRTHRTIVVNIV
FSVTTKLAIVALAFAGHPLIWAAVLADVGTCLLVIMYSMLLLREKGSGKVAKKCCASSHSKKHGHR
TTHHCSDGHHHENVSTGGCVDSSAGKHSCHDHHHEHDHHKEPSNLHSVDKHGCHDHGHVHSHCKEP
SSQMVTSKDVAHGHGHTHNICNPHPAANKHDCHDHEHSHHQEPNSSHSADEHDCHGHKHCEEPTSL
LCATEHACHDHDQNHEHHCCDEEKTVHVADTHSCHDHKHEQGAADSVPELSIWIEGQSPDHREQEI
QCSTEHKEEACGHHLKVKDQVPAKTDCSRGGCHGTASSKTCESKGKNVCSSWPVGRTGVVRRCCRT
RTHSCCSQSMLKLPEIIVG
>HvHMA2B
MAGKLEKSYFDVLGICCPSEVPLVEKLLEPLAGVRKVTVVVPSRTVIVLHDAAAISQAQIVKALNG
ARLQASVRAYGGGGQSKINKWPSPYVLLCGVLLVASLFQHFWRPLRWLALVATAAGLPPIVLRSVA
AARRLTLDVNVLMLIAVAGAVALKDYSEAGFIVFLFTTAEWLETRASCKATAGMSSLMSMAPQNAV
LAETGQVVAAQDVKVNTVIAVKAGEVVPIDGVVVEGRSEVDEQTLTGESFPVAKQTDSQVWAGTLN
IDGYISVRTTAMADNSAVAKMARLVEEAQNSRSETQRLIDTCAKYYTPAVIVMAAAVAVTPVIVRA
HNLRHWFQLALVLLVSACPCALVLSTPVATFCALLMAARTGLLIKGGDVLESLAGIKVAAFDKTGT
ITSGEFSVAEFRPVGERVPRQQLLSWVSSVESRSSHPMAAALVDYARSNSAKPTPENVMEFQIYPG
EGIYGEIDGHGVYVGNRRILSRASCETVPEVNDIKGVTVGYVACNKELVGIFGLSDVCRTGSAEAI
RELRSMGIKSVMLTGDSAAAATHAQNQLGNVLAEVHSDLLPEDKVRLVDELKARDGPTLMIGDGMN
DAPALARADVGVSMGVSGSAVAMETSHVTLMSNDIRRIPKAVRLARRTRRTIVTNIVFSVATKLAI
VGLALAGHPLVWAAVLADVGTCLLVIMYSMMLLRGGDGARHGPRSHIEKHGTSASHHHHCSDGPCK
SAGGCGDSSAGGHACGDEHHCHGHGDREEPGSPHHHGCEDHGHGHGHCKEPSKLHPMDSHRCQDHG
HGHGHSHCKEASIKLVTSKNISHGHGHSHCKEKHDEHSTNSVESSTQEHSILIDESAAAQQQILCD
HQIEEECGHHSKAKARATARPTDCGSLSLRRDTVGDDNEGCGTKARDACSSRRAGCAAGETGRCCR
SARASRCGGHASMLKLPEIVVE
>HvHMA3
MTGSGESYPALEASLLSDEAAASARRKWEKTYLDVLGVCCSAEVALVERLLAPLDGVRAVSVVVPS
RTVVVEHDPSAVSQSRIVKVLNGAGLEASVRAYGSSGVIGRWPSPYIVACGALLLASSFRWLLPPL
QWLALGAACAGAPPMVLRGFAAASRLALDINILMLIAVVGAVALKDYTEAGVIVFLFTTAEWLETL
ACTKASAGMSSLMSMIPPKAVLAETGEVVNVRDIDVGAVIAVRAGEMVPVDGVVVDGQSEVDERSL
TGESYPVPKQPLSEVWAGTLNLDGYIAVRTSALAENSTVAKMERLVEEAQQSKSKTQRLIDSCAKY
YTPAVVFLGAGVALLPPLVGARDAERWFRLALVLLVSACPCALVLSTPVATFCALLTAARMGLLVK
GGDVLESLGEIKAVAFDKTGTITRGEFTVDIFDVVGHKVQMSQLLYWISSIESKSSHPMAAALVEY
AQSKSIEPKPECVAEFRILPGEGIYGEIDGKRIYVGNKRVLARASSCQTVPERMNGLKGVSIGYVI
CDGDLVGVFSLSDDCRTGAAEAIRELASMGISSVLLTGDSAEAAVHAQERLGGALEELHSELFPED
KVRLVSAVKARVGPTMMVGDGMNDAPALAMADVGVSMGISGSAAAMETSHATLMSSDILRVPEAVR
LGRRARRTIAVNMVSSVAAKVAVLALALAWRPVLWAAVLADVGTCLLVVLNSMLLLGEGGGRRGKE

EACRATARSLEMRRSQLAAVSPDAATKSVGKTGGDASKGCHCCHKPIKSPEHSVVINVRVDEQREG
PTDATCTPAKNVEVTGLVDASVMPASSSCVSGGGCCSREKTGRNM
>HvHMA4
MERNGQSHLKEPLLHAGDGASPAAARVSPRKERTTRKVMFNVRGMSCGSCAVSIETVVAGLKGVES
IQVSTLQGQAVVQYSPEETDARTIKEAIEDINFEVDELQEQEIAVCRLRIKGMACTSCSESIERAL
LMVPGVKKAAVGLALEEAKVHFDPNITSRDLLIEAIEDAGFGADLISYGDDVNKMHLKLEGVSSPE
DTKLIQSALETVEGVNNVEWDTLGQTVTVAYDPDVTGPRLLIQRIQDAAQPPKCFNASLYSPPKQR
EVERHHEIMSYRNQFLWSCLFSVPVFLFAMVLPMLPPSGDWLFYKIYNNMTVGMLLRWLLCSPVQF
IIGWRFYVGAYHALKRGYSNMDVLVALGTNAAYFYSVYIIVKALTSDSFEGQDLFETSSMLVSFIL
LGKYLEVVAKGKTSDALSKLTELAPETAVLLTMDKDGGVISEVEISTQLLQRNDFIKIVPGEKVPV
DGVVIKGQSHVNESMITGEARPIAKKPGDKVIGGTVNDNGFIIVKATHVGSETALSQIVQLVEAAQ
LARAPVQRLADKISRFFVPTVVVAAFLTWLGWFIPGQLHLYPQRWIPKAMDSFELALQFGISVLVV
ACPCALGLATPTAVMVATGKGASLGVLIKGGNALEKAHKIKTIIFDKTGTLTKGKPSVVQTKTFSK
IPLLELCDLTASAEANSEHPLSKAIVEYTKKLREQYGSPSDHMMDSKDFEVHPGAGVSANVEGKLV
LVGNKRLMQEFEAPMSSEVEEYMSEMEDLARTCVLVAIDRVICGALAVSDPLKPEAGRVISHLSSM
GITSIMVTGDNWATAKSIAKQVGISTVFAEIDPVGKAEKIKDLQTQGLAVAMVGDGVNDSPALAAA
DVGMAIGAGTDVAIEAADIVLMKSSLVDVITAIDLSRKTLAKIRLNYVWALGYNVLGMPIAAGVLF
PFTGIRLPPWLAGACMAASSVSVVCSSLLLQLYKKPLHVEEAPVPAGPGDGGSNLV
>HvHMA5
MAAGTRGAVFLACFRGGGAEGSGHRLALRPRYPPMPRRTKAAAVAGDLEAAAGADEEGEEEEEKVA
VFAVTGMTCAACAGSVEKAVKRLPGIHDAAVDVLGCRAQVAFYPAFVSEEKIRETIEDVGFGAKLI
DEELREKSILVCRLHIKGMTCTSCANTVESTLQAVPGVQRASVALAIEEAEIRYDRRVVAATQLVN
AVEESGFEAILVTAGEDRSRIDLKVDGILDETSVMIVKSSVQALPGVEDIKIDTELQKITISYKPD
KTGPRDLIEVIESAGSGLVAVSIYPEADGREQHRNGEIRRYRQSFLWSLLFTIPVFLTSMVFMYIP
GLKDGLDKKVVNMMSIGELLRWILSTPVQFIIGRKFYTGAYKAMCHGSPNMDVLIALGTNTAYFYS
VYSVLRAATSENYMSIDFFETSSMLISFILLGKYLEILAKGKTSEAIAKLMDLAPETATVLIYDKE
GNVVSEKEIDSRLIQKNDVIKVIPGGKVASDGFVIWGRSHVNESMITGESRPVAKRKGDTVIGGTV
NENGVLHVRATFVGSESALAQIVRLVESAQMAKAPVQKFADQISKVFVPLVIFLSLLTWLTWFLAG
RFHGYPSSWIPSSMDSFQLALQFGISVMVIACPCALGLATPTAVMVATGVGASQGILIKGGQALES
AQKVDCIIFDKTGTLTIGKPIVVNTRLFENMVLREFYDYVAAAEVNSEHPLAKAIVEHAKKFHSEE
THIWPEARDFISVTGHGVKAKIGDKSVIVGNKSFMLSLDIDVPVEASEILMEEEEKAHTGIIVAMD
QEIVGIISVSDPIKPNAHEVISYLESMKVECIMVTGDNWGTANAIGKEVGIQNIIAEAKPEQKAEK
VKELQLLGRTVAMVGDGINDSPALVAANVGMAIGAGTDVAIEAADIVLMKSNLEDVITAIDLSRKA
FFRIRMNYVWALGYNIIGIPIAAGVLFPSTRFRLPPWVAGAAMAASSVSVVCWSLLLRYYKRPLIT
QERQK
>HvHMA6
MAHLQLTAVAGGRDDEMEEVALLGSYGEPEGLSSRTGQEEEEEEDAGMRRAQVRVTGMTCSACTGA
VEAALSARRGVRSAAVSLLQNRAHVVFDPALAKEEDIVEAIEDAGFEAEILPDSAVSQPKSQKALS
GQFRIGGMTCAACVNSVEGILKKLPGVNRAVVALATSLGEVEYDPAAISKDEIVQAIEDAGFEAAL
LQSSEQDKALLGLIGLHTERDVNLLYDILRKTEGLRQFDVNSVRAEVEITFDPEVVGLRSIVDIIE
IESSGRLKAHVQNPYVRSSSNDAQEASKMLHLLRSSLFLSIPVFFMRMVCPHISFINSFLLMHCGP
FRIGDLLKWMLVSVVQFVVGKRFYVAAYRALRHGSTNMDVLVVLGTTATYVYSVCALLYGAFTGFH
PPMYFETSAMIITFVLLGKYLEVLAKGRTSDAIKKLVELVPATAILLLKYKDGKYAGEKEIDALLI
QPGDVLKVLPGSKIPADGIVTWGTSHVDESMVTGESASISKEVSSSVIGGTMNLNGTLHIQAAKVG
SGTVLSQIISLVETAQMSKAPIQKFADYVAGIFVPIVITLSLLTFCTWFVCGTLGAYPNSWVSETS
NCFVFSLMFSISVVVIACPCALGLATPTAVMVATGVGANHGVLVKGGDALERAQNVKYIIFDKTGT
LTQGKATVTTTKVFSGMDVGDFLTLVASAEASSEHPLAKAILDYAFHFHFFGKLPSSKDDVKKRKE
DAFSQWLLEVADFSALPGKGVQCLINGKMILVGNRALISENGVNIPEEAESFLVDMELNAKTGILV
AYDGDFIGLMGVTDPLKREAAVVIQGLKKMGIYPVMVTGDNWRTALAVAKEIGIEDVRAEVMPAGK

ADVIRSLQKDGSVVAMVGDGINDSPALAAADVGMAIGAGTDIAIEAADYVLVRNNLEDVITAIDLS
RKTFSRIRWNYFFAMAYNIVAIPVAAGALFPLIGLQMPPWLAGACMAFSSVSVVCSSLLLRRYRKP
RLTTVLQITVE
>HvHMA7
MDPATPLLAISRAISSRSRTFPASSPHNILLRGRPPTAPLGRAPVFAASAQRRFAVSGDLLFLSLA
RLALRGPASPRAGPRRWFASVSASSLASAGPPGGGCGRGNGDGGGGGDGGGDGWKRPRASQGTAVA
EEAAGLEADTIILDVGGMSCGGCAASVKRILENEPQVVSATVNLATEMAVVWAVPEDRAVQDWKLQ
LGEKLASQLTTCGYKSSQRDSSKVSSQNVFERKMGEKLQNLKQSGRELAVSWALCAVCLLGHISHL
FGVNAPLMHLFHSTGFHLSLSIFTFIGPVSSVAAFIPKLGWKTFFEEPIMLIAFVLLGKNLEQRAK
LKAASDMTGLLNILPSKARLMDRIPADGLVKAGRSTVDESSLTGEPMPVTKIAGAEVSAGSINLNG
KLTVEVRRPGGETVMSDILHLVEEAQTREAPVQRLADKVAGNFTYGVMALSSATFMFWSIFGSQLV
PAAIQQGSAMSLALQLSCSVLVIACPCALGLATPTAVLVGTSLGATRGLLLRGGDVLEKFAEVDAI
VFDKTGTLTIGKPVVTKVIASHSEGGVNTKDYRNNEWTEGDVLSLAAGVESNTNHPLGKAIMEAAQ
AANCINMKAKDGSFMEEPGSGAVATIGEKQVSVGTLDWIRRHGVVREPFPEAENFGQSVAYVAVDG
TLAGLICFEDKIREDSHQVINALSKQGISVYMLSGDKESAAMNVASIVGIQLDKVISEVKPHEKKK
FISELQKEHKLVAMVGDGINDAAALALADVGIAMGGGVGAASDVSSVVLMGNRLSQLVDALELSKE
TMRTVKQNLWWAFLYNIVGLPVAAGALLPVTGTMLTPSIAGALMGFSSVSVMANSLLLRARMSSKH
HVQSRQKPHNTISDVSDGAGEVEQSYPSKWRST
>HvHMA8
MMCGGCAARVRSILAADARVENAAVNLLAESAAVRLRSPAPGAGEELAERLTVCGFPSAARRGGAT
AGAAESALKWKEMAARKSELLTRSRGRVAFAWTLVALCCGSHASHLLHSLGIHIGHGTFFDVLHNS
YVKCGLAVVALFGPGRDILFDGLRAFKQGSPNMNSLVGFGSAAAFAISAVSLLNPELAWNSTFFDE
PVMLLGFVLLGRSLEESARLKASSDMNELISLLSPQSRLIVTSSSDDLPSDGILNSDAITVEVPVD
DVRVGDSVLVLPGETIPVDGNVTGGSSFVDESMLTGESLPVAKEKGCPVFSGTVNWDGPLRIKATT
TGPSSTIAKIIRMVEDAQAHEAPVQRLADAIAGPFVYTVMTLSAATFSFWYLLGTHLFPEVLLNDI
SGPDGDSLLLSLKLAVDVLVVSCPCALGLATPTAILIGTSMGAKRGLLIRGGDVLERLAGIDAIVL
DKTGTLTKGKPVVTSIASLAYDEVDILRLAAAVEKTALHPIANAIMKEAELCKLDIPTTSGQLTQP
GFGCLAEVDGRLVAVGNLDWVHNRFETKASPTELSDLGKRLEFVPSSEASSSNQSKSIAYIGREGE
GIIGAIAISDVLRDDAKSTVDRLQQEGIATYILSGDRKEAVEGIGEAVGIRSENRRSSLTPQEKAG
IISTLQGEGHRVAMVGDGINDAPSLAAADVGIAMRTHSKENAASDAASVVLLGNRLSQVVDALSLS
KATMAKVHQNLAWAVAYNIVAIPVAAGALLPQFDFAMTPSLSGGLMALSSIFVVSNSLLLQLHGSF
QKTERPGPDDLKSRPKSQM
>HvHMA9
MAHLQLAAVAGGGRAGDDMEDVALLGSYDEETGGAAPAGGGGGAEDEEEAEAHVRVTGMTCSACTS
AVEAAVSARRGVRRVAVSLLQNRARVVFDPALAKVEDIIEAIEDAGFDAEILPDSAVPQSKSQKTL
SAQFRIGGMTCANCVNSVEGILKKQPGIKGAVVALATSLGEVEYDPSTISKDEIVQAIEDAGFDAA
FLQSSEQDKVLLGLTGVHAERDADILHDILKKMDGLRQFGVNTALSEVEIVFDPEAVGLRSIVDAI
EMGSNGRFKAHVQNPYSRGASNDAHEASKMLHLLRSSLFLSIPVFFIRMICPSIPFISTLLLMHCG
PFHMGDLVNWILVSIVQFVIGKRFYVAAYRALRHGSTNMDVLVVLGTTASYVYSVCALLYGAFTGF
QPPIYFETSAMIITFVLFGKYLEVLAKGKTSDAIKKLVELVPATAVLLLKDEEGKYVGEREIDALL
VQPGDVLKVLPGSKVPSDGFVVWGTSHINESMITGESAPMPKEVSSVVIGGTINLHGILHIQATKV
GSGTVLSQIISLVETAQMSKAPIQKFADYVASIFVPIVITLSILTFSVWFLCGSFGAYPHSWFDRT
SNCFVFSLMFSISVVVIACPCALGLATPTAVMVATGIGANHGVLVKGGDALERAQNVNYVIFDKTG
TLTQGKAVVTTAKVFSGMDLGDFLTLVASAEASSEHPLAKAVLEYAFHFHFFGKLPSSKDGLEQRK
EQILSQWLLEAEDFSAVPGKGVQCLINEKKVLIGNRALMNENGVSVPPEAESFLVDLELNAKTGIL
VAYDSSFMGLMGIADPLKREAAVVVEGLKKMGIHPVMLTGDNWRTAQAVAKEVGIEDVRAEVMPAG
KADVVRSLQKDGSIVAMVGDGINDSPALAAADVGMAIGGGTDIAIEAADYVLVRNNLEDVITAIDL
SRKTFNRIRWNYFFAMAYNVVAIPVAAGALFPMTGLQMPPWLAGACMAFSSVSVVCSSLLLRRYRK
PRLTTVLQITIE

>HvP5
MARFEVNGKSVEGVDLLRRRHWTARLDFWPFLALYALWLLLAVPALDFTDALVILGVLSASHILAF
LFTAWSVDFRAFVGHSKVKDIHAADACKVIPAKFLGSKEIVPLHIQKTVASSSAAGETEEIYFDFR
KQRFFYSAEKDNFFKLRYPTKDLFGHYIKGTGYGTEAKINTAMDKWGRNIFEYPQPTFQKLMKEQC
MEPFFVFQVFCVGLWCLDEYWYYSLFTLFMLFLFESTMAKNRLKTLTELRRVKVDNQIVLTYRCGK
WVKISGTELLPGDIVSIGRSPSGEDRSVPADMLLLSGSAIVNEAILTGESTPQWKVSVAGRGPDEM
LSIKRDKNHILFGGTKILQHTPDKSVNLRAPDGGCVAFVLRTGFETSQGKLMRTILFSTERVTANS
KESGLFILFLLFFAIIASGYVLMKGLEDPTRSRYKLFLSCSLILTSVIPPELPMELSIAVNTSLIA
LVRRGIFCTEPFRIPFAGKVDICCFDKTGTLTSDDMEFQGVVSLESDAELISDANKLPLRIQEVLS
SCHALVFVDNKLVGDPLEKAAIKGIDWIYTSDEKAMSRRPGGQPVQIVHRHHFASHLKRMSVIVRI
QEKFYAFIKGAPETIQERLVDLPAAYVETYKKYTRQGSRVLSLAYKLLPEMPVSEARSLERDQVES
DLIFAGFAVFNCPIRSDSAAVLLELEQSSHDLVMITGDQALTACHVASQVNICLKPVLILTRMKTG
GFEWVSPDETDRVPYRAEEVKELSESHDLCVSGDCFEMLQRTDAVVQVIPHVKVFARVAPEQKELV
LTTFKTVGRMTLMCGDGTNDVGALKQAHVGIALLNAEPVQKAGSKSQSSKLESKSGKLKKPKPATE
SSSQLVPPATSSAKAPSSRPLTAAEKQREKLQKMLDEMNDESDGRSAPIVKLGDASMASPFTAKHA
SVAPTLDIIRQGRSTLVTTLQMFKILGLNCLATAYVLSVMYLDGVKLGDVQATISGVFTAAFFLFI
SHARPLQALSAERPHPNIFCAYVFLSILGQFAMHLFFLMSAVNLASKYMPEECIEPDSEFHPNLVN
TVSYMVNMMIQVATFAVNYMGHPFNQSISENKPFKYALYSAVVFFTVITSDMFRDLNDYMKLEPLP
EGMRGKLLLWAMLMFCGCYGWERFLRWAFPGKMPAWEKRQKQAVANLDKKQA
>OsAHA1
MAEDKGGLDAVLKESVDLENIPIEEVFQNLKCCRQGLTSEEAQLRLQLFGPNKLEEKEESKFLKFL
GFMWNPLSWVMEAAAIMAIALANGGGKPPDWQDFVGIITLLLINSTISFIEENNAGNAAAALMARL
APKAKVLRNGSWTEEEAAILVPGDIISIKLGDIIPADARLLEGDPLKIDQSALTGESLPATKGPGD
GVYSGSTVKQGEIEAVVIATGVHTFFGKAAHLVDSTNQVGHFQKVLTAIGNFCICSIAVGMFVEII
VMYPIQHRPYRPGIDNLLVLLIGGIPIAMPTVLSVTMAIGSHRLSQQGAITKRMTAIEEMAGMDVL
CSDKTGTLTLNKLTVDKNLIEIFERGVTQDQVILMAARASRTENQDAIDTAIVGMLADPKEARAGI
QEVHFLPFNPTDKRTALTYIDSDGKMYRVSKGAPEQILNLAHNKTQIERRVHAVIDKFAERGLRSL
AVAYQEVPDGRKESPGGPWRFVALLPLFDPPRHDSAETIRRALNLGVNVKMITGDQLAIGKETGRR
LGMGTNMYPSSALLGQNKDESVAALPVDDLIEKADGFAGVFPEHKYEIVKRLQARKHICGMTGDGV
NDAPALKKADIGIAVADATDAARSASDIVLTEPGLSVIISAVLTSRAIFQRMKNYTIYAVSITIRI
VFGFMLLALIWEFDFPPFMVLIIAILNDGTIMTISKDLVKPSPLPDSWKLAEIFTTGVVLGGYLAM
MTVIFFWAAYKTNFFPRIFHVESLEKTAQDDYQKLASAVYLQVSTISQALIFVTRSRSWSFIERPG
FLLVFAFFVAQLIATLIAVYANWAFTSIKGIGWGWAGIVWLYNLVFYFPLDIIKFLIRYALSGKAW
DLVIEQRIAFTRKKDFGKEERELKWAHAHRTLHGLQPPDAKPFPEKTGYSELNQMAEEAKRRAEIA
RLRELHTLKGHVESVVKLKGLDIDTIHQSYTV
>OsAHA10
MASSLSLDDINDDSVDLSKAPVAEVFQKLKCDRKGLTGAEGESRLRLYGPNKLEEKKESKLLKFLG
FMWNPAVIAAIMAIVLANGGGRPPDWQDFVGIVSLLIINSTISYIEEANAGDAAAALMAGLAPKTK
LLRDGRWEEQEAAILVPGDIISIKLGDIIPADARLLEGDPLKIDQSALTGESLPVNKHPGQEVFSG
STVKQGEIEAVVIATGVRTFFGKAAHLVDSTNNVGHFQQVLTAIGNFCIISIGAGMAVEVLVMYPI
QHRAYRDGIDNLLVLLIGGIPIAMPTVLSVTMAIGSHRLSQQGAITKRMTAIEEMAGMDVLCSDKT
GTLTLNKLTVDKTLIEVCSKGVDKDMVLLYAARASRVENQDAIDTCIVNMLDDPKEARAGIQEVHF
LPFNPVDKRTAITYIDGNGDWHRVSKGAPEQIIELCNMAADAEKKVHALIDSYADRGLRSLGVSYQ
QVPEKSKDSGGDPWQFIGLLPLFDPPRHDSAETIRRALHLGVNVKMITGDQLAIAKETGRRLGMGT
NMYPSTTLLGDKNSQVNGLPIDELIERADGFAGVFPEHKYEIVKRLQEMSHICGMTGDGVNDAPAL
KKADIGIAVDDATDAARSASDIVLTEPGLSVIVSAVLTSRAIFQRMKNYTIYAVSITIRIVLGFLL
IAIIWKFDFAPFMVLIIAILNDGTIMTISKDRVKPSPTPDCWKLNEIFLTGVVLGTYMALVTVLFF
YLAHDTNFFTDVFGVTSIRESERELMAALYLQVSIISQALIFVTRSRSWSFVERPGFLLLFAFFAA

QMVATAIAVYARWDFCRIQGIGWRWGGAVWQFSVVTYLPLDVLKFIIRYALTGGKAGDSAQKKASS
PPPTSQP
>OsAHA2
MAEKGDNLEAVLNESVDLENIPLEEVFEHLRCNREGLTSANAEQRLNLFGPNRLEEKKESKFLKFL
GFMWNPLSWVMEAAAIMAIALANGGGKPPDWQDFVGIITLLIINSTISFIEENNAGNAAAALMARL
APKAKVLRNGRWSEEEAAILVPGDIISVKLGDIIPADARLLEGDPLKIDQSALTGESLPVTKGPGD
GVYSGSTCKQGEIEAVVIATGVHTFFGKAAHLVDSTNQVGHFQKVLTAIGNFCICSIAIGMVVEII
VMYPIQHRDYRPGIDNLLVLLIGGIPIAMPTVLSVTMAIGSHRLAQQGAITKRMTAIEEMAGMDVL
CSDKTGTLTLNKLTVDKSLIEVFQRGVDQDTVILMAARASRTENQDAIDATIVGMLADPKEARAGI
QEVHFLPFNPTDKRTALTYIDGEGKMHRVSKGAPEQILNLAHNKTEIERRVRAVIDKFAERGLRSL
GVAYQQVPDGRKESPGGPWQFVGLLPLFDPPRHDSAETIRRALNLGVNVKMITGDQLAIGKETARR
LGMGTNMYPSSALLGQDKDESIVALPVDELIEKADGFAGVFPEHKYEIVKRLQARKHICGMTGDGV
NDAPALKKADIGIAVDDSTDAARSASDIVLTEPGLSVIISAVLTSRAIFQRMKNYTIYAVSITIRI
VLGFMLLALIWKFDFPPFMVLIIAILNDGTIMTISKDRVKPSPQPDSWKLSEIFATGVVLGSYLAM
MTVIFFWVAYKTDFFPRVFHVESLEKTAQDDFQKLASAVYLQVSTISQALIFVTRSRSWSFVERPG
FLLVFAFFVAQLIATLIAVYANWGFASIKGIGWGWAGVIWLYNIVFYLPLDIIKFLIRYALSGRAW
DLVLEQRIAFTRKKDFGTQENQLKWATAQRTIHGLQPAATAAVFRDMTSYNDLNQLAEEARRRAEI
ARLRELTTLKGRMESVVKQKGLDLETIQQSYTV
>OsAHA3
MAEKEGNLDAVLKEAVDLENIPLEEVFENLRCSREGLTTQQAQQRLEIFGPNKLEEKEESKFLKFL
GFMWNPLSWVMEAAAIMAIALANGGGKPPDWQDFVGIITLLVINSTISFIEENNAGNAAAALMARL
APKAKVLRDGRWTEEEAAILVPGDIVSIKLGDIIPADARLLEGDPLKIDQSALTGESLPVTKGPGD
GVYSGSTVKQGEIEAIVIATGVHTFFGKAAHLVDSTNQVGHFQKVLTAIGNFCICSIAVGMFVEII
VMYPIQHRAYRPGIDNLLVLLIGGIPIAMPTVLSVTMAIGSHRLSQQGAITKRMTAIEEMAGMDVL
CSDKTGTLTLNKLTVDKNLIDVFERGITQDQVILMAARASRTENQDAIDTAIVGMLADPKEARAGI
QEVHFLPFNPTDKRTALTYIDGDGKMYRVSKGAPEQILHLAHNKPEIERRVHAVIDKFAERGLRSL
AVAYQEVPEGTKESPGGPWHFVGLMPLFDPPRHDSAETIRRALNLGVNVKMITGDQLAIGKETGRR
LGMGTNMYPSSALLGQNKDESIAALPVDDLIEKADGFAGVFPEHKYEIVKRLQARKHICGMTGDGV
NDAPALKKADIGIAVADATDAARSASDIVLTEPGLSVIISAVLTSRAIFQRMKNYTIYAVSITIRI
VLGFMLLALIWKFDFPPFMVLIIAILNDGTIMTISKDRVKPSPLPDSWKLAEIFTTGVVLGGYLAM
MTVIFFWAAYKTDFFPRIFHVESLEKTAQDDFQKLASAVYLQVSTISQALIFVTRSRSWSFVERPG
FLLVFAFLVAQLIATLIAVYADWAFTSIKGIGWGWAGIVWLYNLIFYFPLDIIKFLIRYALSGKAW
DLVIEQRIAFTRKKDFGKEERELKWAHAQRTLHGLQPPDAKMFSEKAGYNELNQMAEEAKRRAEIA
RLRELHTLKGHVESVVKLKGLDIETIQQSYTV
>OsAHA4
MSVSLEDLKKENVDLESIPIQEVFAVLKSSPQGLTSADGNGRLEIFGRNKLEEKKESKLLKFLGFM
WNPLSWVMEAAAIMAIALANGGGRPPDWQDFVGIVTLLFINSTISFIEENNAGNAAAALMASLAPQ
TKARRACALLRDGKWSEQDAAILVPGDIISIKLGDIIPADARLMEGDPLKIDQSALTGESLPVNKM
PGDSIYSGSTCKQGEIEAVVIATGVHTFFGKAAHLVDSTNNVGHFQKVLTAIGNFCICSIAAGMLI
EIIVMYPIQHRQYRDGIDNLLVLLIGGIPIAMPTVLSVTMAIGSHRLSQQGAITKRMTAIEEMAGM
DVLCSDKTGTLTLNKLTVDKNMIEDPFVKDLDKDAIVLYAAKASRTENQDAIDASIVGMLADPSEA
RAGIQEVHFMPFNPVDKRTAITYIDTKDGSWHRISKGAPEQIIELCRLRDDVSRRVHAIIDKFADR
GLRSLAVARQKVPEGSKDAPGTPWQFLAVLPLFDPPRHDSSETIRRALNLGVNVKMITGDQLAIGK
ETGRRLGMGTNMYPSSSLLKDGDTGGLPVDELIEKADGFAGVFPEHKYEIVRRLQERKHICGMTGD
GVNDAPALKKADIGIAVADATDAARGASDIVLTEPGLSVIISAVLTSRAIFQRMKNYTIYAVSITI
RVVLGFLLLALIWRFDFAPFMVLIIAILNDGTIMTISKDRVKPSPLPDAWRLQEIFATGIVLGTYL
ALATVLFFWAVRDTDFFTRTFGVHPIGGSTEELMAAVYLQVSIISQALIFVTRARSWFFVERPGLL
LVGAFLIAQLMATLIAVYANWPFAKMKGIGWSWGMVIWLFSIVTFFPLDIFKFAIRYFLSGKAWNN

AFDNKTAFANELDYGKSKREAQWAIAQRSLHGLQQAETSTALFDDNKDYLELSEIAEQAKRRAEIA
RLRELHTLKGHVESVVKLKGLDIDTIQNHYTV
>OsAHA5
MAATASSTADALEQIKNEAVDLEHIPLEEVFQHLKCTREGLTNAEGDARTQVFGPNKLEEKKESKI
LKFLGFMWNPLSWVMEVAAIMAIALANGGGRPPDWQDFVGIIALLLINSTISYWEESNAGSAAAAL
MKNLAPKTKVLRDGRWSETDAFVLVPGDVINVKLGDIVPADARLLDGDPLKIDQSALTGESLPVTK
LPGDCVYSGSTCKQGEIDAVVIATGVHTFFGKAAHLVDTTNQVGHFQKVLRAIGNFCIGAIAIGMA
VEVIVMYLIQHRLYRDGIDNLLVLLIGGIPIAMPTVLSVTMAIGSHRLSDQGAITKRMTAIEEMAA
MDVLCSDKTGTLTLNKLSVDRGLIEVFVQGVAKDEVILLTARASRVENQDAIDTAMVGMLDDPKEA
RAGIREEHFLPFNPVDKRTALTYVDLADGSWHRVSKGAPEQILDLCKCRQDVRSKVHAIIDRYADR
GLRSLAVARQEVPERRKDGPGGPWEFVGLLPLLDPPRHDSAETIRRALHLGVNVKMITGDQLAIAK
ETGRRLGMGVNMYPSSALLGQSKDESIASVPVDELIKKADGFAGVFPEHKYEIVKKLQEMKHICGM
TGDGVNDAPALKRADIGIAVADATDAARSASDIVLTQPGLSVIISAVLTSRAIFQRMKNYTIYAVS
ITIRIVLGFMLIALIWKFDFSPFMILVIAILNDGTIMTISKDRVKPSPHPDSWKLPEIFITGIVYG
TYLAVMTVLFFWAMRSTDFFTSTFHVKPLMEKDEMMSALYLQVSIISQALIFVTRSRSWCFVERPG
MLLCGAFVAAQIIATLVTVYATLGFAHIKGIGWGWAGVIWLYSIVTFLPLDIFKFAVRYALSGRAW
DTLIEHKIAFTSKKDYGRGEREAQWATAQRTLHGLQTPEMGTTSAASYRELSEIAEQAKRRAEVAR
LRELSTLKGQMESTVRLKGLDMDNVQHHYTV
>OsAHA6
MASISLEDVRNETVDLETIPVEEVFQHLKCSKQGLSAAEGQNRLNIFGPNKLEEKTESKLLKFLGF
MWNPLSWVMEAAAIMAIVLANGGGRPPDWQDFVGIVVLLVINSTISFIEENNAGNAAAALMAGLAP
KTKVLRDGKWQEQDASILVPGDIISIKLGDIIPADARLLEGDPLKVDQAALTGESMPVNKHAGQGV
FSGSTVKQGEIEAVVIATGVHTFFGKAAHLVDSTNNIGHFQLVLTAIGNFCIISIGVGMIIEIIVM
YPIQHRAYRDGIDNLLVLLIGGIPIAMPTVLSVTMAIGSHRLSQQGAITKRMTAIEEMAGMDVLCS
DKTGTLTLNKLTVDKTLIEVYGRGLDKDSVLLYAARASRVENQDAIDTCIVGMLADPKEARAGIKE
VHFLPFNPVEKRTAITYIDGNGEWHRISKGAPEQIIELCKMSKDAEKKVHTLIDQYADRGLRSLGV
SYQKVPEKSKESEGEPWQFVGLLPLFDPPRHDSAETIRRALHLGVNVKMITGDQLAIGKETARRLG
MGTNMYPSTTLLGDKSSEMSGLPIDELIEKADGFAGVFPEHKYEIVKRLQDRKHICGMTGDGVNDA
PALKKADIGIAVDDATDAARSASDIVLTEPGLSVIVSAVLTSRAIFQRMKNYTIYAVSITIRIVLG
FMLVALLWKFDFAPFMVLIIAILNDGTIMTISKDRVKPSPTPDSWKLKEIFATGIVLGTYMALITA
LFFYLAHDTDFFTETFGVRSIKTNEKEMMAALYLQVSIISQALIFVTRSRSWSFVERPGALLVIAF
LAAQLVATCIAVYAEWEFCKMQGIGWGLGGAIWAFSVVTYFPLDVLKFIIRYALSGRAWNNINNKT
AFVNKNDYGKGEREAQWATAQRTLHGLNQSSTSSDLFNDKTGYRELSEIAEQAAKRAEVARLRELH
TLKGHVESVVKLKGLDIDTIQQSYTV
>OsAHA7
MTGRAHPPIVIENIPIEEVFEQLKCTREGLSSEEGNRRIEMFGPNKLEEKKESKILKFLGFMWNPL
SWVMEMAAIMAIALANGGGKPPDWEDFVGIIVLLVINSTISFIEENNAGNAAAALMANLAPKTKVL
RDGRWGEQEAAILVPGDIISIKLGDIVPADARLLEGDPLKIDQSALTGESLPVTKNPGDEVFSGST
CKQGEIEAVVIATGVHTFFGKAAHLVDSTNQVGHFQTVLTAIGNFCICSIAVGIVIEIIVMFPIQH
RAYRSGIENLLVLLIGGIPIAMPTVLSVTMAIGSHKLSQQGAITKRMTAIEEMAGMDVLCSDKTGT
LTLNKLSVDKNLVEVFTKGVDKDHVLLLAARAFRTETQDAIDAAMVGMLADPKEARAGIREVHFLP
FNPVDKGTALTYIDADGNWHRASKGAPEQILTLCNCKEDVKRKVHAVIDKYAERGLRSLAVARQEV
PEKSKESAGGPWQFVGLLPLFDPPRHDSAETIRKALHLGVNVKMITGDQLAIGKETGRRLGMGTNM
YPSSALLGQNKDASLEALPVDELIEKADGFAGVFPEHKYEIVKRLQEKKHIVGMTGDGVNDAPALK
KADIGIAVADATDAARSASDIVLTEPGLSVIISAVLTSRCIFQRMKNYTIYAVSITIRIVLGFLLI
ALIWKYDFSPFMVLIIAILNDGTIMTISKDRVKPSPLPDSWKLKEIFATGIVLGSYLALMTVIFFW
AMHKTDFFTDKFGVRSIRNSEHEMMSALYLQVSIVSQALIFVTRSRSWSFIERPGLLLVTAFMLAQ
LVATFLAVYANWGFARIKGIGWGWAGVIWLYSIVFYFPLDIFKFFIRFVLSGRAWDNLLENKIAFT

TKKDYGREEREAQWATAQRTLHGLQPPEVASNTLFNDKSSYRELSEIAEQAKRRAEIARLRELNTL
KGHVESVVKLKGLDIDTIQQNYTV
>OsAHA8
MEVANAMDAITKETVDLEHIPVEEVLDHLKCTREGLTSEVAQQRIHSFGYNKLEEKQESKLLKFLG
FMWNPLSWVMEAAAIMAIALAHGGRDARGKRMRIDYHDFVGIVLLLFINSTISFMEENNAGNAAAA
LMARLAPKAKVLRDGTWDELDASLLVPGDIISVKLGDIIPADARLLEGDPLKIDQSALTGESLPVT
KHPGDGIYSGSTCKQGEIEAVVIATGIHTFFGKAAHLVESTTHVGHFQKVLTSIGNFCICSIAAGM
VIELLVMYAVHERKYRQIVDNLLVLLIGGIPIAMPTVLSVTMAIGSHKLAQQGAITKRMTAIEEMA
GMDVLCSDKTGTLTLNKLSVDKNLIEVFEKGIEKDDVVLMAARASRLENQDAIDFAIVSMLPDPKE
ARAGIQEVHFLPFNPTDKRTALTYLDAEGKMHRVSKGAPEQILNLASNKCEIERKVHHVIGNFAER
GLRSLAVAYQEVPEGTKESPGGPWQFVGLLPLFDPPRHDSAETIRRALDLGVSVKMITGDQLAIGK
ETGRRLGMGTNMYPSSSLLGDRKDGDIAVLPVDELIEQADGFAGVFPEHKYEIVQRLQARKHICGM
TGDGVNDAPALKKADIGIAVADATDAARSASDIVLTEPGLSVIISAVLTSRAIFQRMKNYTIYAVS
ITVRIVLGFLLLACFWKFDFPPFLVLVIAILNDGTIMTISKDKVKPSPYPDSWKLTEIFATGVIIG
AYLAVTTVLFFWAAYKTQFFVHLFNVDTLNINKKLASAVYLQVSTISQALIFVTRSRGWSFLERPG
LLLMAAFVIAQLIATVLAAIATWEVASIRGIGWRWAGAIWVYNIVVYLLLDPMKFAVRYGLSGKAW
NLVIDNKVAFTNRKDFGREARVVAWAHEQRTLHGLQSAASREKAASTELNQMAEEARRRAEITRLR
ELHTLKGKVESVAKLKGIDLEDVNNQHYTV
>OsAHA9
MDEPGEPLLGLENFFDEDVDLENLPLEDVFEQLNTSQSGLSSADAAERLKLFGANRLEEKRENKII
KFLSFMWNPLSWVMEAAAVMALVLANGGSQGTDWEDFLGIVCLLIINSTISFIEENNAGDAAAALM
ARLALKTKVLRDEQWQELDASTLVPGDIISIRLGDIVPADARLLEGDPLKIDQSALTGESLPVTKR
TGDIVFTGSTCKHGEIEAVVIATGIHSFFGKAAHLVDSTEVVGHFQKVLTSIGNFCICSIAIGAIV
EVIIMFPIQHRSYRDGINNVLVLLIGGIPIAMPTVLSVTLAIGSHHLSQQGAITKRMTAIEEMAGM
DVLCCDKTGTLTLNHLTVDKNLIEVFSREMDREMIILLAARASRVENQDAIDMAIINMLADPKEAR
SSITEVHFLPFNPVDKRTAITYVDSDGNWFRVSKGAPEQILSLCYNKDDISEKVQLIIDRFAERGL
RSLAVAYQEVPEKSKHGHGGPWVFCGLLPLFDPPRHDSADTIRRALDLGVCVKMITGDHLAIAKET
GRRLGMGTNMYPSASLFGRHGDGGGAAVPVEELVEKADGFAGVFPEHKYEIVRMIQGGGGHVCGMT
GDGVNDAPALKKADIGIAVSDATDAARGAADIVLTEPGLSVIVSAVLTSRAIFQRMKNYTIYAVSI
TIRIVIGFVLLASIWEYDFPPFMVLIIAILNDGTIMTISKDRVKPSPSPDSWKLNEIFAAGVVIGT
YLALVTVLFYWTVTRTTFFESHFKVRSLKQNSDEISSAMYLQVSIISQALIFVTRSQGLSFLERPG
ALLIGAFILAQLVATLIAVYATISFASISAIGWGWAGVIWLYSLVFYAPLDLIKIAVRYTLSGEAW
NLLFDRKAAFASRRDYGGNERRPETRALSDHLLSSGWRPTRIAERAKRRAEIARLGDAHMLRAHVQ
SVMRLKRVDSDVIRSAQTV
>OsACA1
MESYLKENFGGVKAKHSSDEALGRWRRLVGVVKNPKRRFRFTANLDKRSEAAAMKRSNQEKLRVAV
LVSKAALQFIQGLAPASEYTVPDDVKAAGYGICAEELSSIVESHDIKKLKSHGGVEAIAAKLCTSP
EDGLPKSRRRQAVREELFGINRFAETESRSFWVFVWEALQDMTLMILAACAFFSLVVGIATEGWPK
GAHDGLGIVASILLVVFVTATSDYRQSLQFKDLDKEKKKITVQVSRNGYRQKLSIYDLLAGDIVHL
SIGDQVPADGLFLSGFSLLINESSLTGESEPVAVNAENPFLLSGTKVQDGSCKMLVTTVGMRTQWG
KLMATLSEGGDDETPLQVKLNGVATIIGKIGLIFAVVTFAVLTEGLFRRKIMDASYLSWTGDDAME
LLEFFAIAVTIVVVAVPEGLPLAVTLSLAFAMKKMMNDKALVRHLAACETMGSATTICSDKTGTLT
TNHMTVVKACICGKIKDVESASDTKSLFSELPESAMTLLSQSIFNNTGGDVVFNKSGSREILGTPT
ETAILEFGLSLGGDFLAVRKASTLVKVEPFNSAKKRMGVVIQLPGGAMRAHSKGASEIILASCSKY
LNDQGNVVPLDDATVAHLNATINSFANEALRTLCLAYVDVGDGFSANDQIPEDGYTCIGIVGIKDP
VRPGVKESVAICRSAGIMVRMVTGDNINTAKAIARECGILTEGGIAIEGPDFRTKSAEELNELIPK
IQVMARSSPLDKHTLVKHLRTTFDEVVAVTGDGTNDAPALHEADIGLAMGIAGTEVAKESADVIIL
DDNFSTIVTVAKWGRSVYINIQKFVQFQLTVNVVALIVNFSSACLTGSAPLTAVQLLWVNMIMDTL
GALALATEPPNDELMKRTPVGRKGNFISNIMWRNILGQAFYQFIVIWYLQTEGKWLFGLKGENSDL

VLNTLIFNCFVFCQVFNEVSSREMERINVFEGILDNNVFVAVLGSTVIFQFIIVQFLGDFANTTPL
TLKQWFNCIFIGFIGMPIAAAVKLIPVDF
>OsACA10
MALGRSSPPAPEIRSPELDGAEDTDVEEKFDDAFDIPHKNASHDRLRRWRQAALVLNASRRFRYTL
DLKKEEEKEMIRRKIRAHAQVIRAAFLFKEAGEKDLREAYTGIKLETASRSFPIELEKLTALNRDH
DSVLLQEVGGVKGLSDLLKSNLEKGISLNADDLLQRRGIFGANTYPRKKRKSILRFIFEACKDLTL
IILMVAAAISLTLGMTTEGADEGWYDGGSIFLAVFLVILVTAISDYRQSLQFRHLNEEKQNIQVEV
VRGGKRCGTSIFDLVVGDVVPLKIGDQVPADGVLISGHSLAIDESSMTGESKTVHKDKKEPFLMSG
CKVADGYGSMLVTGVGTNTEWGQLMANLSEDNGEETPLQVRLNGVATFIGMVGLTVAGAVLVVLWI
RYFTGHTKDPDGTTQFVAGTTRAKKGFMGAIRILTIAVTIVVVAVPEGLPLAVTLTLAYSMRKMMR
DKALVRRLSSCETMGSATTICSDKTGTLTLNKMTVVQAYFGGTMLDPCDDIRAVSCGATELLIEGI
AQNTTGTIFVPEDGGDAELSGSPTEKAILSWGLKIGMDFNDARSKSQILHVFPFNSEKKRGGVAVQ
SDAGVHVHWKGAAELVLSSCKSWLALDGSVQPMSAEKYNECKKSIEDMATSSLRCVAFAYCPCEIE
RIPKEDIADWKLPEDDLTLLCIVGIKDPCRPGVKSAVQLCTNAGVKVRMVTGDNIETAKAIALECG
ILDANGAFVEPFVIEGKVFREMSEAARGDIVDKITVMGRSSPNDKLLLVQALKRKGHVVAVTGDGT
NDAPALHEADIGLSMGISGTEVAKESSDIIILDDNFTSVVKVVRWGRSVYANIQKFIQFQLTVNVA
ALVINVVAAVSSGDVPLNAVELLWVNLIMDTLGALALATEPPTDNLMKRQPVGRRHEPLVTNIMWR
NLFVQAIYQIAILLIFDFSGRSILRLQNDSREDAEKTQNTFIFNTFVFCQIFNEFNARKPEERNVF
KGITKNHLFMGIIAITTVFQILIIEFLGKFFKTVRLNWRLWLVSVAIGIISWPLAYLGKFIPVPVR
PLQDYFKPTCWRRASRRDEEESGQS
>OsACA11
MESASSSLATSGRRRSSSGGGGGSWGSIGSAADPFDIPAKGAPVESLKKWRQAALVLNASRRFRYT
LDLKREEQREEVISKIRAQAHVVRAAFRFKEAGQVHVQQKEVAAPPVDGALGFGIKEDQLTALTRD
HNYSALQQYGGISGVARMLKTDTEKGISGDDSDLTARRNAFGSNTYPRKKGRSFLAFLWDACKDLT
LIILMVAAAVSLALGITTEGIKEGWYDGASIAFAVLLVVVVTATSDYKQSLQFQNLNEEKQNIKLE
VVRGGRRISVSIYDLVAGDVVPLKIGDQVPADGILISGHSLSVDESSMTGESKIVHKDQKSPFLMS
GCKVADGYGTMLVTAVGINTEWGLLMASISEDSGEETPLQVRLNGVATFIGMVGLSVALAVLVVLL
ARYFTGHTYNPDGSVQYVKGKMGVGQTIRGIVGIFTVAVTIVVVAVPEGLPLAVTLTLAFSMRKMM
RDKALVRRLSACETMGSATTICSDKTGTLTLNQMTVVEAYFGGKKMDPPDNVQVLSASISSLIVEG
IAQNTSGSIFEPENGQDPEVTGSPTEKAILSWGLKLGMRFNDTRTKSSILHVFPFNSEKKRGGVAV
HLGGSEVHIHWKGAAEIILDSCKSWLAADGSKHSMTPEKISEFKKFIEDMAASSLRCVAFAYRTYE
MVDVPSEDRRADWILPEDDLIMLGIVGIKDPCRPGVKDSVRLCAAAGIKVRMVTGDNLQTARAIAL
ECGILSDPNVSEPVIIEGKAFRALSDLEREEAAEKISVMGRSSPNDKLLLVKALRKRGHVVAVTGD
GTNDAPALHEADIGLSMGIQGTEVAKESSDIIILDDNFASVVRVVRWGRSVYANIQKFIQFQLTVN
VAALIINVVAAVSSGNVPLNAVQLLWVNLIMDTLGALALATEPPTDHLMQRPPVGRREPLITNVMW
RNLIIMALFQVIVLLTLNFRGTSLLQLKNDNQAHADKVKNTFIFNTFVLCQVFNEFNARKPDELNI
FKGITGNHLFMAIVAITVVLQALIVEFLGKFTSTTRLTWQLWLVSIGLAFFSWPLAFVGKLIPVPE
RPLGDFFACCCPGSKQAADAKGDDADHSDV
>OsACA2
MESYLEENFGGVKAKNSSEEALRRWRKLCGVVKNPKRRFRFTANLDKRGEAQAIKHANHEKLRVAV
LVSKAALQFIQGLSLRSEYVVPEEVKAAGFQICADELGSIVEGHDSKKLITHGGVTGIADKLATSP
ADGLSTAEESIKRRQDVYGLNKFTESEVRSFWVFVWEALQDTTLIILAVCAFVSLVVGIAMEGWPK
GAHDGLGIVASILLVVFVTATSDYRQSLQFKDLDKEKKKIQVQVTRNGFRQRLSIYDLLPGDVVHL
AIGDQVPADGLFISGFSLLINESSLTGESEPVVVNEDNPFLLSGTKVQDGSCKMLITTVGMRTQWG
KLMATLSEGGDDETPLQVKLNGVATIIGKIGLFFAVITFIVLSQGLISKKYHEGLLLSWSGDDALE
MLEHFAIAVTIVVVAVPEGLPLAVTLSLAFAMKKMMNDKALVRHLAACETMGSATTICSDKTGTLT
TNHMTVVKACICGNIKEVNNPKNASDLCSELPETVVKTLLESIFNNTGGEVVIDQDGKYQILGTPT
ETALLEFALSLGGNFKAKRDETKIVKMEPFNSTKKRMCVVLKLPGGGCRAHCKGASEIVLAACDKF
MDETGAVVPLDKTTADKLNGIIESFANEALRTLCLGYREMEEGLSVEEQIPLQGYTCIGIVGIKDP

VRPGVRESVATCRSAGIMVRMVTGDNINTAKAIARECGILTEDGLAIEGPEFREKSLDELLKLIPK
IQVMARSSPLDKHTLVKHLRTTFNEVVAVTGDGTNDAPALHEADIGLAMGIAGTEVAKESADVIIL
DDNFSTIVTVAKWGRSVYVNIQKFVQFQLTVNVVALLVNFSSACFTGNAPLTAVQLLWVNMIMDTL
GALALATEPPNDDLMKREPVGRTGKFITNVMWRNILGQSFYQFIVMWYLQTQGKSMFGLDGPDAEV
VLNTIIFNSFVFCQVFNEISSREMEKINVLRGILKNYVFLGVLTSTVVFQFIMVQFLGEFANTIPL
TRLQWIASVLLGLIGMPISAIIKLLPVGSS
>OsACA3
MHTGVNGCCPLRLPAAAAVHGRRIPPLLPPRGAWPGCIAAPALHRKPGRGGGGALSICRRASHHEK
LQVAALPSKATLEFEHGVSLRSAYIVPEDVQAAGFQIDADELASIVESRDTKKLTVHGQLNGIADK
LGTSLTNGIVTDKDLLNQRQDIYGVNKFAETEIRSFWEFVWEALEDTTLIILSACAIFSLVVGITT
EGWPQGAHDGVGIVASILLVVSVTGTSNYQQSLQFRDLDKEKRKILVQVTRNGLRQRVLIDDLLPG
DAVHLAVGDQVPADGLFISGFSVLVDESSLTGESEPVFVNEDNPYLLSGTKVLDGSCKMLVTAVGM
RTQWGKLMAVLTDGGDDETPLQTRLNGVANTIGKIGLFFAVLTFIVLSQGIIGQKYLDGLLLSWSG
DDVLEILDHFAVAVTIVVVAVPEGLPLAVTLSLAFAMKKMMNDKALVRQLAACETMGSATVICSDK
TGTLTTNRMTVVKACICGNTIQVNNPQTPNMSSNFPEVAVETLLESIFNNTSGEVVTNQDGKYQIL
GTPTETALLEFALLLDGDCKEKQLGSKIVKVEPFNSTKKRMSTILELPGGGYRAHCKGASEIVLAA
CDKFIDERGCIVPLDDKTSSKLNDIIKAFSSEALRTLCLAYREMEEGFSTQEQIPLQGYTCIGIVG
IKDPVRPGVRQSVATCRSAGISVRMITGDNIDTAKAIARECGILTKDGIAIEGAEFREKSAEELHD
LIPKMQVLARSSPLDKHTLVKHLRTAFNEVVAVTGDGTNDAPALREADIGLAMGIAGTEVAKESAD
VVILDDNFSTIVTVAKWGRSVYVNIQKFVQFQLTVNVVALLVNFTSACFTGDAPLTAVQLLWVNMI
MDTLGALALATEPPNNNLMKKAPVGRKGKFITNVMWRNIVGQSLYQFAVMWYLQTQGKHLFGLEGY
HADIVLNTIIFNTFVFCQVFNEISSREMEDINVLRGMAGNSIFLGVLTGTIFFQFILVQFLGDFAN
TTPLTQQQWLISILFGFLGMPIAAAIKLIAVEPHEKADTRRTP
>OsACA4
MEKLDRYLQEHFDEHFDVPAKNPSEEAQRRWRQAVGTIVKNRRRRFRWVPDLDRRSLDKAKVRSTQ
EKIRVALYVQQAALIFSDEFKLTEDIIKARFSINPDELALITSKHDSKALKMHGGVDGISKKVRSS
FDHGICASDLDTRQNIYGVNRYAEKPSRSFWMFVWDAFQDMTLIILMVCALLSVAVGLATEGWPKG
MYDGLGIILSIFLVVMVTAVSDYKQSLQFKELDNEKKKIFIHVTRDGRRQKISIYDLVVGDIVHLS
IGDQVPADGLYIHGYSLLIDESSLSGESDPVYVSQDKPFILAGTKVQDGSAKMIVTAVGMRTEWGK
LMSTLSEGGEDETPLQVKLNGVATVIGKIGLVFAILTFLVLLVRFLIDKGMTVGLLKWYSTDALTI
VNYFATAVTIIVVAVPEGLPLAVTLSLAFAMKKLMNDKALVRHLSACETMGSAGTICTDKTGTLTT
NYMVVDKIWISEVSKSVTSNTISGELNSVVSSRTLSLLLQGIFENTSAEVVKEKDGKQTVLGTPTE
RAILEFGLGLXGXHDAEYXACTKVKVEPFNSVKKKMAVLISLPXGTSRWFCKGASEIILQMCDMMV
DGDGNAIPLSEAQRKNILDTINSFASDALRTLCLAYKEVDDDIDDNADSPTSGFTLIAIFGIKDPV
RPGVKDAVKTCMSAGITVRMVTGDNINTAKAIAKECGILTEDGVAIEGPEFHSKSPEEMRDLIPNI
QVMARSLPLDKHTLVTNLRGMFDEVVSVTGDGTNDAPALHEADIGLAMGIAGTEVAKESADVIVLD
DNFTTIINVARWGRAVYINIQKFVQFQLTVNIVALVINFVSACITGSAPLTAVQLLWVNMIMDTLG
ALALATEPPNDEMMKRPPVRKGESFITKVMWRNIMGQSLYQLFVLGALMFGGESLLNIKGADSKSI
INTLIFNSFVFCQVFNEINSREMQKINVFRGIISNWIFIAVIAATVAFQVVIIEFLGTFASTVPLN
WQHWLLSVGLGSISLIVGVILKCIPVGSGETSATPNGYRPLANGPDDI
>OsACA5
MEKLDRYLQENFDVPAKNPSEEAQRRWRQAVGTIVKNRRRRFRWVPDLERRSLDKAKEKIRVALYV
QQAALIFSDGAKKKEYKLTGDIIKAGYAINPDELALITSKHDSKALKMHGGVDGISIKVRSSFDHG
IYASELDTRQNIYGVNRYAEKPSRSFWMFVWDALQDMTLIILMVCALLSVAVGLATEGWPKGMYDG
LGIILSIFLVVMVTAVSDYKQSLQFKELDNEKKKIFIHVTRDGRRQKISIYDLVVGDIVHLSIGDQ
VPADGLYIHGYSLLIDESSLSGESDPVYVSQDKPFILAGTKVQDGSAKMIVXAVGXRTEWGKLMST
LSEGGEDETPLQVKLNGVATIIGKIGLVFAILTFLVLLVRFLIDKGMTVGXLKWYSTDALTIVNYF
ATAVTIIVVAVPEGLPLAVTLSLAFAMKKLMNDKALVRHLSACETMGSAGTICTDKTGTLTTNHMV
VDKIWISEVSKSVTSNTISGELNSVVSSSTLSLLLQGIFENTSAEVVKEKDGKQTVLGTPTERAIL

EFGLGLKGDHDAEYRACTKVKVEPFNSVKKKMAVLISLPNGTSRWFCKGASEIILQMCDMMVDGDG
NAIPLSEAQRKNILDTINSFASDALRTLCLAYKEVDDDIDDNADSPTSGFTLIAIFGIKDPVRPGV
KDAVKTCMSAGITVRMVTGDNINTAKAIAKECGILTEDGVAIEGPEFHSKSTEEMRDLILNIQVMA
RSLPLDKHTLVTNLRGMFDEVVSVTGDGTNDAPALHEADIGLAMGIAGTEVAKESADVIVLDDNFT
TIINVARWGRAVYINIQKFVQFQLTVNIVALVINFVSACIIGSAPLTAVQLLWVNMIMDTLGALAL
ATEPPNDEMMKRPPVRKGESFITKFMWRNIMGQSLYQLFVLGALMFGGERLLNIKGADSKSIINTL
IFNSFVFCQVFNEINSREMQKINVFRGIISNWIFIAVIAATVAFQVVIIEFLGTFASTVPLNWQHW
LLSVGLGSISLIVGVILKCIPVGSGETSATPNGYRPLANGPDDI
>OsACA6
MEFLKSFEVPAKNPSEEAQRRWRDAVGTLVKNRRRRFRMVPDLDKRSQAETQRRKIQEKLRVALFV
QKAALQFIDAVRKTEHPLPELARQCGFSVSAEELASIVRGHDTKSLRFHNGVDGIARKVAVSLADG
VKSDDAGLRAEVYGANQYTEKPPRTFWMFLWDASQDMTLLLLAFCAAVSVAIGLATEGWPSGMYDG
VGIMLTILLVVMITAASDYKQSLQFRDLDKEKKKIDVQVTRDGYRQKVSIYDIVVGDIVHLSIGDQ
VPADGLFIDGYSFVVDESNLSGESEPVHVSTANRFLLGGTKVQDGSARMLVTAVGMRTEWGNLMET
LSQGGEDETPLQVKLNGVATIIGKIGLAFAVLTFTVLMARFLLGKAGAPGGLLRWRMVDALAVLNF
FAVAVTIIVVAVPEGLPLAVTLSLAFAMKKLMQERALVRHLSACETMGSASCICTDKTGTLTTNHM
VVEKIWASGAAQTMSNAKGFDQLTSSMSETFAKVLLEGVFHCSGSEVVRGKDGRHTIMGTPTETAI
LEFGLAVEKRARIEHTGAGKLKVEPFNSVKKTMAVVIASPSAGGRPRAFLKGASEVVLSRCSLVLD
GTGNVEKLTDAKAKRVASAIDAFACEALRTLCLAYQDVDGGGGDIPGEGYTLIAVFGIKDPLRPGV
REAVATCHAAGINVRMVTGDNINTAKAIARECGILTDDGIAIEGPEFRNKDPDQMREIIPKIQVMA
RSLPLDKHTLVTNLRGMFNEVVAVTGDGTNDAPALHEADIGLAMGIAGTEVAKENADVIIMDDNFS
TIINVAKWGRSVYINIQKFVQFQLTVNVVALMVNFISASFTGSAPLTIVQLLWVNLIMDTLGALAL
ATEPPNDAMMKRPPVGRGDNFITKVMWRNIVGQSIYQLVVLGVLLLRGKSLLQINGPQADSLLNTF
VFNTFVFCQVFNEVNSREMEKINVFSGIFSSWIFSAVVGVTAGFQVIMVELLGTFANTVHLSGKLW
LTSVLIGSVGLVIGAILKCIPVESGSDASDRHDGYRPIPTGPSAV
>OsACA7
MEGGRSWSIESYLNEYFDIPAKNPPGEARRRWRRAVGLIVRNRRRRFGRFSDVDAIDEAQRRKILG
KVQVVINVHKAALQFIDGVKQYHLPPELIEEGFCISPDELAAIANMREDYTMLRMHGGINGISRKI
KASLEDGAKETDIATRQMLYGANRHAEKPPRSFWMFVWDALHDLTLIILVVCALVSIVVGLATKGW
PMGIYDGFGIILSILLVVLVTATSDYQQARKFMELDREKQKIYIRVTRDKKTKEVLVHDLVVGDIL
HLSIGDVVPADGLFISGDCLMIDESSLSGESEPVNISEERPFLHAGNKVVDGAAKMLVTAVGTRTE
WGKIMGTLNGDGVDETPLQVKLNGVATIIGQIGLVFAVLTFLVLLARFLADKGMHVGLLNWSANDA
LTIVNYFAIAVTIIVVAVPEGLPLAVTLSLAFAMKKLMHDKALVRHLAACETMGSASCICTDKTGT
LTTNHMIVDKVWIGDVKFVGDKKNSELKSTISERVMAILIQGIFVNTASEVVKGDDGKNTILGLAT
ETALLEFGLSLEEHLYDDYNKLTRIKVDPFNSVKKKMSVTIQLPNGGIRTFCKGASEIILEQCNTI
HNTDGNIVPLSEMQKHNVLNIINSFASEALRTLCIAFKDMDEFPNDQPISDDGYTLIAVFGIKDPV
RPGVKDAVRTCMAAGIRVRMVTGDNINTAKAIAKECGILTEDGIAIEGQQLNNKSSDELKELLPKI
QVIARSLPMDKYKLVTSLKSMYQEVVAVTGDGTNDAPALHESDIGLAMGITGTEVAKESADVIIMD
DNFETIVNVARWGRAVYLNIQKFVQFQLTVNIVALIVNFVSACIIGSAPLTAVQLLWVNMIMDTLG
ALALATEPPNDEMMKRPPVRRGDNFITRIMWRNILGQGLYQLLVLATLMVIGKKLLSIEGPQSDKT
INTLIFNSFVFCQVFNEINCREMEKINVLQGIFRNWIFVGILTATVIFQVIIVEFLGTFANTVPLS
GELWLLSVVIGSISMIISVILKCIPVEFNKTNTKPHGYELIPEGPEIL
>OsACA8
MECADYFIGSGRRCSPSTSTSTSREAWRPEKQWRKATNVIRGCHRLLRLGVLSAAAGIMRRNPSYV
EIKVHDEGELDVSSGGDGEAPVAFTVAADDESFKGLVKNKREDCFRLLGGGAGVAAVLASGAERGI
RGDDADVARRKKAFGSNTYPKPKPKGFFRHVWDALADVFLIVLLVCAAVSLAFGIKEHGIKDGWYD
GVSIFLAVFLVAAVSAVSNHSQGKRFDKLARESENIMVSVVRAARRQEVSIFDVVVGDVVVLKIGD
VVPADGVFLDGHALQVDESSMTGEPHPVEVDAVKSPFLASGVKVVDGYGKMVVTAVGTDTAWGEMM
RTITRENTDPTPLQERLEGLTSSIGKVGIAVAVLVFAVLTARHFTGSTRDEQGNALFDKRNVTFNA

VFSGLVGIFQQAVTIIVVAIPEGLPLAVTLTLAFSMKRMVRENALVRRLSACETMGSVTAICTDKT
GTLTLNQMKVTEFWVGADRPRSAAAVNGGVVRLLCQGAGLNTTGSVYKPDNVSPPEITGSPTEKAL
LSWAVEELPMDADALKRKCKVVRVEAFNSDKKRSGVMLRDAATGAVTAHWKGAAEMVLARCTVYVG
ADGAARELGVEQRRKLEQVINDMAAASLRCIAFAYKQVVDGGDSDNAKIDDEGLTLLGFVGLKDPC
RPEVKSAIEACTKAGIAVKMVTGDNVLTARAIAKECGIISGNDDDAAGVVIEGHEFRAMSEQEQLA
IVDNIRVMARSLPLDKLVLVQRLKQKGHVVAVTGDGTNDAPALKEADVGLSMGVQGTEVAKESSDI
VILNDNFDTVVTATRWGRCVYNNIQKFIQFQLTVNVAALVINFVSAVTTGRMPLTTVQLLWVNLIM
DTMGALALATDTPTAGLMRRPPIGRAAPLISNAMWRNLAAQAAYQVAVLLALQYRGFGGAGAGERA
NGTMIFNAFVLCQVFNEFNAREIERRNVFAGVHRNRMFLGIVAVTVALQVVMVELLTKFAGTERLG
WGQWGACVGIAAVSWPIGWAVKCIPVPERPFHEIITARRRRRST
>OsACA9
MCVQIGGGRRLVVSGADPFDIPAKRASVERLRRWRQAALVLNASRRFRYTLDLKKEEEKEQIRRKI
RAHAQVIRAALLFKEAGQKHDRENLTHSYNAEILPRGFGIGEEQLTAMTRDHDYSSLHGYGGAFKG
LANLLKTNTEKGVHGDEVDLACRANAFGANRYPRKKGRSFLVFLWEACQDLTLVILIIAAVISLVL
GIATEGIKEGWYDGASIAFAVFLVILVTAVSDYKQSLQFQHLNEEKQNIQVEVIRGGRRIEVSIFD
IVVGDVVALKIGDQVPADGVLVSGHSLAIDESSMTGESKIVVKDHKSPFLMGGCKVADGYGTMLVT
AVGLNTEWGLLMASISEDNNEETPLQVRLNGVATFIGIVGLSVAAMVLIVLVARYFTGHTTNPDGS
IQFVKGQTSVKSTIFGTIKILTIAVTIVVVAVPEGLPLAVTLTLAYSMQKMMADKALVRRLSACET
MGSATTICSDKTGTLTLNQMTVVRSVVGGIKLKSPADIENLSPVVSSLILEGIAQNSSGSVFEPED
GSPIEITGSPTEKAILSWGVELHMKFAEEKSKSSIIHVSPFNSEKKRAGVAVIVDDSDIHVHWKGA
AEIVLALCTNWLDVNGISHEMTPDKANQFKKYIEEMAEESLRCVAFAYRNLDLNYVPNEEERINWE
LPDNELALIGIVGMKDPCRPGVRNAVDLCKNAGVKVRMVTGDNLQTARAIALECGILTDSQASQPV
IIEGKVFRAYSDAEREAVADQISVMGRSSPSDKLLLVKALKKKGNVVAVTGDGTNDAPALHEADIG
LAMGIQGTEVAKESSDIIILDDNFASVVKVVRWGRSVYANIQKFIQFQLTVNVAALIINVVAAISS
GNVPLNAVQLLWVNLIMDTLGALALATEPPTDQLMKRPPVGRKEPLVTNIMWRNLFIQAVFQVTVL
LTLNFRGRDLLHLTQDTLDHANKVKNTFIFNTFVLCQVFNEFNSRKPYELNIFDGVSRNHLFLAVV
SITVVLQVIIIEFLGKFTSTVRLSWKLWLVSVGIGFVSWPLAFAGKFIPVPRTELKTYIS
>OsECA1
MGKGGQEEGKRRDGSDASGSEPAAAAFPAWARTPSECLAELGVAADRGLSSEEAAARLRRYGPNEL
ERHAAPSVWKLVLEQFDDTLVRILLAAAVVSFVLALYDGAEGGEVGATAFVEPLVIFLILIVNAVV
GVWQESNAEKALEALKEIQSEHATVKRDGRWSHGLPARDLVPGDIVELRVGDKVPADMRVLQLISS
TLRVEQGSLTGETASVNKTSHKIELEDTDIQGKECMVFAGTTIVNGSAVCVVTGTGMDTEIGKIHA
QIQEASQEEDDTPLKKKLNEFGEALTAIIGVICALVWLINVKYFLTWEYVDGWPRNFKFSFEKCTY
YFEIAVALAVAAIPEGLPAVITTCLALGTRKMAQKNALVRKLPSVETLGCTTVICSDKTGTLTTNQ
MSAVKLVAIGRWPDTLRSFKVDGTTYDPSDGKINEWPSLSMDENLQMIAKIAAVCNDASIAHSEHQ
YVATGMPTEAALKVLVEKMGLPGGYTPSLDSSDLLRCCQWWNNAAKRVATLEFDRTRKSMGVIVKK
ADSGKNLLLVKGAVENLLERSGYIQLLDGSVVLLDEGAKALILSTLREMSASALRCLGFAYKEDLA
EFATYDGEEHAAHKYLLDPSYYSSIESNLIFCGFVGLRDPPREEVHKAIEDCRAAGIRVMVITGDN
KETAEAICREIGVFGSTEDISSKSFTGKEFMSLSDKKKLLRQTGGLLFSRAEPKHKQEIVRLLKED
GEVVAMTGDGVNDAPALKLADIGVAMGITGTEVAKEASDMVLADDNFSTIVAAVGEGRSIYDNMKA
FIRYMISSNIGEVASIFLTSALGIPEGLIPVQLLWVNLVTDGPPATALGFNPPDKDIMKKPPRRSD
DSLITPWILFRYMVIGMYVGIATVGVFIIWYTHGSFLGIDLAGDGHSLVSYSQLSNWGQCSSWEGF
KVSPFTAGARTFNFDVNPCDYFQGGKIKATTLSLSVLVAIEMFNSLNALSEDGSLLSMPPWVNPWL
LLAMSVSFGLHFLILYVPFLAQVFGIVPLSFNEWLLVIAVAFPVVLIDEVLKFVGRCLTARARKQS
GKQKED
>OsECA2
MGEAGQDAPPPPEKGVEVEVFPAWARGVEECEARLGVSASRGLSSREAAARLRAHGPNELAEHPGP
TLLQLVAQQFDDTLVRILLAAAAVSFALALSSSAGAVTLSAFVEPLVIFLILVVNAAVGVWQETNA
EKALEALREIQSDHAAVLRDGDWLPSLPARDLVPGDIVQLRVGDKVPADMRVLRLVTSTLRVEQGS

LTGETASVNKTAHQVPHDDADIQAKECMVFAGTTVVNGSAICLVVHTGMATEIGKIHAQIHEAAQE
DDDTPLKKKLNEFGEALTKIIGLICALVWLINVKYFLTFELDGWMPRNIRFSFEKCTYYFEIAVAL
AVAAIPEGLPAVITTCLALGTRKMAAKNALVRKLPSVETLGCTTVICSDKTGTLTTNQMSVAKLVA
IGDAEGKVRSFKVDGTTYDPRDGRIHDWPAGRMDANLQTIAKISAVCNDASVAHSSHQYTATGMPT
EAALKVLVEKMGIPEGMNGLSLDPSETLGCCQWWSNVAKRIATLEFDRTRKSMGVIVKSKSGRNAL
LVKGAVENLLERSSHIQLQDGSVVPLDEKSRKAILENLHEMSIKALRCLGFAYKEDLAEFASYDGE
NHPAHKLLLDPVNYAAIETNLIFTGLAGLRDPPREEVFDAIEDCRAAGIRVMVITGDNKETAEAIC
REIGVFSHDEDITLKSLTGKEFMALEDKKTLLRRKGGLLFSRAEPRHKQEIVRLLKEDGEVVAMTG
DGVNDAPALKLADIGVAMGITGTEVAKEASDMVLADDNFSTIVAAVGEGRSIYNNMKAFIRYMISS
NIGEVASIFLTSALGIPEGLIPVQLLWVNLVTDGPPATALGFNPPDKDIMKKPPRKSDDSLITPWI
LFRYLVIGLYVGIATVGIFVIWYTHGSFMGIDLTGDGHTLVSYSQLSNWGQCSTWNNFTVTPFTAG
ARTFTFDDNPCEYFHGGKVKATTLSLSVLVAIEMFNSLNALSEDTSLLRMPPWVNPWLLLAMSVSF
GLHFLILYVPFLAQVFGIVPLSLNEWLLVLLVALPVVLIDEVLKFVGRCTSSSGPKRRTRKQKGE
>OsECA3
MEDAYAKSVAEVLAAFGVDPTKGLSDEQVASMLGFTAKTSCPKKKVSTPFWKLVLKQFDDLLVKIL
IAAAVISFLLARMNGETGLAAFLEPSVIFLILAANAAVGVITETNAEKALEELRAYQADVATVLRN
GCFSILPATELVPGDIVEVGVGCKVPADMRTIEMLSHQLRVDQAILTGESCSVAKELESTSTMNAV
YQDKTNILFSGTVVVAGRARAVVIGVGSNTAMGSIRDAMLRTEDEATPLKKKLDEFGTFLAKVIAG
ICILVWVVNIGHFRDPSHGGFLRGAIHYFKVAVALAVAAIPEGLPAVVTTCLALGTKRMARLNAIV
RSLPSVETLGCTTVICSDKTGTLTTNMMSVSKVCVVRSVHQRPITDEYSISGTTFAPDGFIYDAGG
LQLEFPPQSSCLLHIAMCSALCNESTLQYNPDKKCYEKIGESTEVALRVLVEKVGLPGFDSMPSAL
NMLTKHERASYCNRYWENQFRKISVLEFSRDRKMMSVLCSRKQQEIMFSKGAPESVMARCTHILCN
DDGSSVPLTMDIRNELEARFQSFAGKDTLRCLALALKRMPEGQQSLSYDDEANLTFIGLVGMLDPP
REEVRNAIHSCMSAGIRVIVVTGDNKSTAESLCRQIGAFEHLEDFTGYSYTASEFEGLPPLEKANA
LQRMVLFSRVEPSHKRMLVEALQLHNEVVAMTGDGVNDAPALKKADIGIAMGSGTAVAKSASDMVL
ADDNFATIVAAVSEGRAIYNNTKQFIRYMISSNIGEVVCIFVAAVLGMPDTLVPVQLLWVNLVTDG
LPATAIGFNKPDSNIMTVKPRKVNEAVVNGWLFFRYLIIGAYVGLATIAGFVWWFVYSEDGPRLPY
SELVNFDSCSTRQTSYPCSIFEDRHPSTVSMTVLVVVEMFNALNNLSENQSLLAIHPWSNLWLVGS
IVLTMLLHISVLYIEPLSALFSVSPLSWAEWKVVLYLSFPVILIDEVLKFFSRSSRGRRFPLRLRR
REILPKESRDN
>OsP5
MARFEVGGKSVEGVDLLRRRHWASRLDFWPFLALYALWLVVVVPALDFTDALVVLGALSASHVLAF
LFTAWSVDFRAFVKDIRAANSCKVTPAKFSGSKEIVPLHIQKTVASSSAAGETEEIYFDFRKQRFI
YSSQEDNFFKLRYPTKEPFEHYIKGTGYGTEAKINTAVDKWGRNIFEYPQPTFQKLMKEQCMEPFF
VFQVFCVGLWCLDEYWYYSLFTLFMLFLFESTMAKNRLKTLTELRRVKVDNQIVATYRCGKWVRIP
GTELLPGDIVSIGRSVSGEDRSVPADMLLLAGSAIVNEAILTGESTPQWKVSVAGRGPEETLSVKR
DKNHILFGGTKILQHTPDKSINLRAPDGGCIAFVLRTGFETSQGKLMRTILFSTERVTANSKESGL
FILFLLFFAVIASGYVLVKGLEDPTRSRYKLFLSCSLILTSVIPPELPMELSIAVNTSLIALARRG
IFCTEPFRIPFAGKVDICCFDKTGTLTSDDMEFQGVVSLEDDEELITDANKLPLRTQEVLSSCHAL
VFVDNKLVGDPLEKAAIKGIDWIYTSDEKAISKKSGGQPVKIVHRYHFASHLKRMSVVVSIHEKYY
AFIKGAPETIQERLVDLPAGYVETYKKYTRQGSRVLALAYKLLPDMPVNEARSLERDQVESDLTFA
GFAVFNCPIRSDSGAVLQELEQSSHDLVMITGDQALTACHVAGQVHICSKPVLILTRTKTGGFEWV
SPDETDRAPYSAEEVAAVSGSHDLCISGDCFEMLQRTDAVIQVIPYVKVFARVAPEQKELVLTTFK
TVGRVTLMCGDGTNDVGALKQVKAHVGIALLNAEPVQKSDTKSQASKSENKQGKLKKPKPSQEGSS
SQLTQPANSSARASSSRPLTAAXRQRERLQKMMDEMNEESDGRSAPIVKLGDASMASPFTAKHASV
APTLDIIRQGRSTLVTTLQMFKILGLNCLATAYVLSVMYLDGVKLGDVQATISGVFTAAFFLFISH
ARPLQTLSAERPHPNIFCAYVFLSILGQFAMHLFFLISAVNEATKYMPEECIEPDSEFHPNLVNTV
SYMVNMMIQVATFAVNYMGHPFNQSITENKPFKYALYAAVAFFTVITSDMFRDLNDYMKLEPLPEG
MRGKLMLWAILMFCGCYGWERILRWAFPGKMPAWEKRQKQAIANREKKHE

>OsHMA1
MQLLTAASASASSAAASPPSPLGSPLLLARRSLPFAPRAHGDHHHGHHHHHHGHGHSHHHGPEVHG
SGGGAAVMRVAKAIGWADVADALREHLQLCCISLGLLLIAAACPHIPVLNSVRRLQDALIAVAFPL
VGVSAALDALVNIADGKINIHVLMALAAFASIFMGNSLEGGLLLAMFNLAHIAEEHFTSKSMIDVR
ELKENHPEFALLLETCGDQSAQFANLCYTKVPVHDLEVGSHILVRAGEAVPVDGEVYQGSSTVTIE
HLTGETKPLERTVGDAIPGGARNLEGMMIVKVTKSWEDSTLNRIVQLTEEGQLNKPKLQRWLDEFG
EHYSRVVVVLSLVVALLGPLLFKWPFFGNSVCRGSIYRGLGLMVAASPCALAVAPLAYATAISSLA
SKGILLKGGHVLDALSACQSIAFDKTGTLTTGKLMCKAIEPIHGHSDVTNDFSDQACCTPNCESEA
LAVAAAMEKGTTHPIGRAVLDHSVGKDLPLVAVESFECLPGRGVVATLSGVKAGNNEDELSKASIG
SVEYISSLYRSSGESEQIKEAVKASAFGPEFVQAALTVDKKVTLFHFEDEPRSGVCEVISTLRDKA
KLRIMMLTGDHESSALRVAKAVCIDEVHCCLKPEDKLNKVKAVSREGGGGLIMVGDGINDAPALAA
ATVGIVLAQRASATAVAVADVLLLQDNICGVPFCIAKARQTTSLVKQSVALALSCIVFAALPSVLG
FLPLWLTVLLHEGGTLLVCLNSIRALNSPTWSWVDDIRQLINSLRKYISSKLQSTSSNYVVDAVPL
>OsHMA2
MAAEGGRCQKSYFDVLGICCPSEVPLVEKLLQPLEGVQKVTVIVPSRTVIVVHDVDAISQSQIVKA
LNQARLEASVRAYGNGSEKITNKWPSPYVLLCGLLLVVSLFEHFWHPLKWFALVAAAAGLPPIVLR
SIAAIRRLTLDVNILMLIAVAGAIALKDYSEAGFIVFLFTTAEWLETRASHKATAGMSALMSMAPQ
KAILAETGEVVAARDVKVNTVIAVKAGEVIPIDGVVVDGRSEVDESTLTGESFPVSKQPDSQVWAG
TLNIDGYIAVRTTAMADNSAVAKMARLVEEAQNSRSSTQRLIDTCAKYYTPAVVVMAGSVAAIPAI
AKAHNLKHWFQLALVLLVSACPCALVLSTPIATFCALLRAARTGLLIKGGDVLESLASIKVAAFDK
TGTITRGEFSVEEFQPVGERVSLQQLLYWVSSVESRSSHPMASVLVDYAQSKSVEPKSENVSESKY
ILVRGFMVKSTEQAYILGTKNFVKSFMRNRLVTIGYVACNNELIGVFTLSDACRTGSAEAIKELRS
LGIKSVMLTGDSSAAATYAQNQLGNILAEVHAELLPEDKVRIVGELKEKDGPTLMVGDGMNDAPAL
AKADVGVSMGVSGSAVAMETSHVALMSNDIRRIPKAVRLARRTHRTIIVNIIFSVITKLAIVGLAF
AGHPLIWAAVLADVGTCLLVIMYSMLLLREKDSRKAKKCAASHHGSPKKCCSSSHHGSHAKKNHGV
SHHCSDGPCKSMVSCKESSVAKNACHDHHHEHNHHEEPAHKHSSNQHGCHDHSHGHSNCKEPSNQL
ITNKHACHDGHNHWRRYEQSARHPXSMNCHGHEHSTCKEELNALPPTNDHACHGHEHSHCEEPVAL
HSTGEHACHEHEHEHIHCDEPIGSHCADKHACHDHEQVHEHHCCDEQQTPHTADLHPCHDHDHDNL
EVEEVKDCHAEPPHHHNHCCHEPHDQVKNDTHPVQEHSISIEESSDHHEHHHNEEHKAEDCGHHPK
PKDCAPPPTDCISRNCCSNTSKGKDICSSLHRDHHTSQASRCCRSYVKCSRPSRSCCSHSIVKLPE
IVVE
>OsHMA3
MAGKDEAEGLEARLLLLPPEAAAEEPTRCGGGDGGGGGRKRKKTYLDVLGVCCSAEVALVERLLAP
LDGVRVVSVVVASRTVVVEHDPAAAPESAIVKALNKAGLEASVRAYGSSGVVSRWPSPYIVASGVL
LTASFFEWLFPPLQCLAVAAVVAGAPPMVRRGFAAASRLSLDINVLMLIAVAGALCLGDYTEAGAI
VFLFTTAEWLETLACTKASAGMSSLMGMLPVKAVIATTGEVVSVRDVRVGDVVAVRAGEIVPVDGV
VVDGQSEVDERSLTGESFPVPKQPHSEVWAGTMNFDGYIAVRTTALAENSTVAKMERLVEAAQNSR
SKTQRLIDSCAKYYTPAVVVVAAGVALIPALLGADGLEQWWKLALVMLVSACPCALVLSTPVASFC
AMLRAARMGIFIKGGDVLESLGEIRAVAFDKTGTITRGEFSIDSFHLVGDHKVEMDHLLYWIASIE
SKSSHPMAAALVEYAQSKSIQPNPENVGDFRIYPGEGIYGEIHGKHIYIGNRRTLARASDQSPEAE
LTELKSVTFTAQEMGEMIKGVSIGYVICDGELAGVFSLSDDCRTGAAEAIRELGSLGIKSVMLTGD
SSAAATHAQGQLGGVMEELHSELLPEDKVRLVSGLKARFGPTMMVGDGMNDAAALAAADVGVSMGI
SGSAAAMETSHATLMSSDVLRVPEAVRLGRCARRTIAVNVAGSVAVKAAVLALAAAWRPVLWAAVL
ADVGTCLLVVLNSMTLLREEWKGGAKEDGACRATARSLVMRSQLAADSQAPNAADAGAAGREQTNG
CRCCPKPGMSPEHSVVIDIRADGERQEERPAEAAVVAKCCGGGGGEGIRCGASKKPTATVVVAKCC
GGGGGGEGTRCGASKNPATAAVVAKCCSGGGGEGIGCGASKKPTATAVVAKCCGGGGEGTRCAASK
KPATAAVVAKCCGGDGGEGTGCGASKRSPPAEGSCSGGEGGTNGVGRCCTSVKRPTCCDMGAAEVS
DSSPETAKDCRNGRCCAKTMNSGEVKG
>OsHMA4

MEQNGENHLKDPLLQADGGGSGASPAGASPRKERKTRKVMFNVRGISCASCAVSIETVVAGLKGVE
SVSVSPLQGQAVVQYRPEEADARTIKEAIEGLNFEVDELQEQEIAVCRLQIKGMACTSCSESVERA
LQMVPGVKKAAVGLALEEAKVHFDPNITSRDLIIEAIEDAGFGADLISSGDDVNKVHLKLEGVSSP
EDIKLIQSRLESVEGVNNVECDTAGQTIIVAYDPDVTGPRLLIQCIQDAAQPPKYFNASLYSPPKQ
REAERHHEIRNYRNQFLWSCLFSVPVFMFSMVLPMISPFGDWLFYKVCNNMTIGMLLRWLLCSPVQ
FIIGWRFYVGAYHALKRGYSNMDVLVALGTNAAYFYSVYIVLKALTSESFEGQDFFETSAMLISFI
LLGKYLEVVAKGKTSDALSKLTELAPETACLLTLDKDGNAISETEISTQLLQRNDVIKIVPGEKVP
VDGVVIKGQSHVNESMITGEARPIAKKPGDKVIGGTVNDNGCIIVKVTHVGSETALSQIVQLVEAA
QLARAPVQKLADRISRFFVPTVVVAAFLTWLGWFVAGQFDIYPREWIPKAMDSFELALQFGISVLV
VACPCALGLATPTAVMVATGKGASQGVLIKGGNALEKAHKVKAIIFDKTGTLTVGKPSVVQTKVFS
KIPLLELCDLAAGAEANSEHPLSKAIVEYTKKLREQYGSHSDHIMESKDFEVHPGAGVSANVEGKL
VLVGNKRLMQEFEVPISSEVEGHMSETEELARTCVLVAIDRTICGALSVSDPLKPEAGRAISYLSS
MGISSIMVTGDNWATAKSIAKEVGIGTVFAEIDPVGKAEKIKDLQMKGLTVAMVGDGINDSPALAA
ADVGLAIGAGTDVAIEAADIVLMRSSLEDVITAIDLSRKTLSRIRLNYVWALGYNVLGMPVAAGVL
FPFTGIRLPPWLAGACMAASSVSVVCSSLLLQLYKKPLHVEEVAAGPKNDPDL
>OsHMA5
MAASTRALFLSCFHGSGGGGGTSEVSRRLVLRPRYPSMPRRPRSAAVAGEGGEGGGGGGDGDLEAA
AVGAEEEEKVAVFEVSGMTCAACAGSVEKAVKRLQGIHDAAVDVLGGRAQVVFYPAFVSEEKIRET
IQDVGFEAKLIDEEVKEKNILVCRLHIKGMTCTSCASTVESILQVVPGVQRASVALATEEAEIRYD
RRIVTASQLTHAVEETGFEAILITTGDDQSRIDLKVDGTLNERSIMIVKSSVQALPGVEDIKVDPE
LHKITISYKPDQTGPRDLIEVIESAASGDLTVSIYPEADGRQQHRHGEIKRYRQSFLWSLVFTIPV
FLTSMVFMYIPGLKDGLEKKVINMMSIGELLRWILSTPVQFVIGRRFYTGAYKALSHGSSNMDVLI
ALGTNTAYFYSVYSILRAASSHNYMATDFFETSSMLISFILLGKYLEILAKGKTSEAIAKLMDLAP
ETATMLIYDHEGNVVGEKEIDSRLIQKNDVIKVVPGGKVASDGFVIWGQSHVNESMITGESRPVAK
RKGDTVIGGTVNENGVLHVRATFVGSESALAQIVRLVESAQMAKAPVQKFADQISRVFVPLVIILS
LLTWLAWFLAGRLHGYPNSWIPSSMDSFQLALQFGISVMVIACPCALGLATPTAVMVATGVGASQG
VLIKGGQALESAQKVDCIVFDKTGTLTIGKPVVVNTRLLKNMVLREFYAYVAAAEVNSEHPLGKAV
VEHAKKFHSEESHVWTEARDFISVTGHGVKAKISGRAVMVGNKSFMLTSGIDIPVEALEILTEEEE
KAQTAIIVAMDQEVVGIISVSDPIKPNAREVISYLKSMKVESIMVTGDNWGTANAISKEVGIENTV
AEAKPEQKAEKVKELQSAGRTVAMVGDGINDSPALVSADVGLAIGAGTDVAIEAADIVLMKSNLED
VITAIDLSRKTFFRIRMNYVWALGYNIIGIPIAAGVLFPSTRFRLPPWVAGAAMAASSVSVVCWSL
LLRYYKSPKLGR
>OsHMA6
MAHLQLTPLAAGGGRGGADEMEEVALLGPDSYDEEAAAAAGPEEEEGMRRVQVRVTGMTCSACTAA
VEAAVSARRGVGGVAVSLLQSRARVVFDPALAKEEDIIEAIEDAGFEAELLPDSTVSQPKLQNTLS
GQFRIGGMTCAACVNSVEGILKKLPGVKRAVVALATSLGEVEYDPSVISKDEIVQAIEDAGFEAAL
LQSSEQDKVLLGLMGLHTEVDVDILHDILKKMEGLRQFNVNLVLSEAEIVFDPEVVGLRSIVDTIE
MESSGRLKAHVQNPYIRAASNDAQEASKMLHLLCSSLFLSIPVFFIRMVCPRIHFTRSLLLMHLGP
FYIGDLLKWILVSIVQFGVGKRFYVAAYRGPEYMVSTNMDVLVVLGTTASYVYSVCALLYGAFTRF
HPPIYFETSAMIITFVLFGKYLEVLAKGRTSDAIKKLVELVPATALLLLKDKEGKYAAEKEIDASL
IQPGDVLKVLPGSKVPADGTVVLGTSHVDESMVTGESAPISKEVSSIVIGGTMNLHGILHIQATKV
GSGTVLSQIISLVETAQMSKAPIQKFADYVAGIFVPIVVTLSLVTFIAWFLCGSLGAYPNSWVDET
SNCFVFSLMFSISVVVIACPCALGLATPTAVMVATGVGANHGVLVKGGDALERAQNVKYVIFDKTG
TLTQGKATVTSTKVFSGIDLGDFLTLVASAEASSEHPLAKAILDYAFHFHFFGKLPSSKDDIKKRK
QQILSQWLLEVAEFSALPGKGVQCLINGKKILVGNRTLITENGINIPEEAESFLVDLELNAKTGVL
VAYDSELIGSIGMTDPLKREAVVVVEGLKKMGIYPVMVTGDNWRTAQAVAKEVGIEDVRAEVMPAG
KADVVRSLQKDGSVVAMVGDGINDSPALAAADVGMAIGAGTDIAIEAADYVLVRNNLEDVITAIDL
SRKTFSRIRWNYFFAMAYNIIAIPVAAGALFPFTGLQMPPWLAGACMAFSSVSVVCSSLWLRRYRK
PRLTTLLQITVE

>OsHMA7
MDPAAPLLALSKAISSSSRSKPSLLASPHHFLLSRGRGSGACGCLPPAPPPPRRTPFAASSASASA
ARRLAVPGDLLLLWLRALRSVARPRARRRADGSRASRRRRTVHRAAVEAGEAVMGAVXGEGGGGWK
RPRASQGTAVAEEASGQEADVIILDVGGMSCGGCAASVKRILESEPQVRSANVNLATEMAVVWAVP
EDEDAKNWKLQLGEKLANQLTTCGYKSNLRDSSKASSQTVFERKMDEKLQQLKQSGRELAVSWALC
AVCLLGHISHLFGVNAPLMHLLHSTGFHLSLSIFTFIGPVSSIAAFVPKLVAPTLLLKGILCHGWK
TFFEEPVMLVAFVLLGKNLEQRAKLKATSDMTGLLNILPSKARLMDRVPADGVVKSGRSTVDESSL
TGEPMPVTKIAGTEVSAGSINLNGKITVEVRRPGGETAMSDILRLVEEAQTREAPVQRLADKVAGN
FTYGVMALSAATYTFWSIFGSQLVPAAIQHGSAMALALQLSCSVLVIACPCALGLATPTAVLVGTS
LGATRGLLLRGGDILEKFSEVDAIVFDKTGTLTIGKPVVTKVIASHREGDENTKVKDSCNNEWTGE
ILSLAAGVESNTTHPLGKAIMEAAQAANCLYLQAKDGSFMEEPGSGAVATIGEKQVSVGTLDWIRR
HGVLHNPFADGENFGQSVAYVAVDGTLAGLICFEDKLREDSHQIIDILSKQGISVYMLSGDKKSAA
MNVASLVGIQADKVIAEVKPHEKKSFISELQKEHKLVAMVGDGINDAAALASADVGIAMGGGVGAA
SDVSSVVLMGNRLSQLVDALELSKETMRTVKQNLWWAFLYNIVGLPIAAGALLPVTGTVLTPSIAG
ALMGFSSVGVMANSLFLRMRLSSRQQPIHKPQATISDVLPNAAESEKSYPSKWSA
>OsHMA8
MAATASRSPLHVTAPVRGVNPLLLRRLRLGRGGGCGKASTAQRFCLVVLPRGPAVATPRSTADPSA
SASSAVDAAAAAGEGEGASDAATVLLDVSGMMCGGCAARVRTILAADERVETAAVNLLAESAAVRL
RSPEPAAGKELAARLTECGFPSVARRGGAASGASDSARKWREMAARKAELLTRSRGRVAFAWTLVA
LCCGSHATHFLHSLGIHVGHGSLSDRFMHGAGTFLDLLHNSYVKCGIAIAALFGPGRGFLSFSQNV
FNVTPDILFDGLRAFKQGSPNMNSLVSLLNPELEWNSTFFDEPVMLLGFVLLGRSLEESARLKASS
DMNELVSLLSPQSRLVVTSSSDDPSSDGVLNSDAITVEVPVDDVRVGDFILVLPGETIPVDGNVLG
GSSFVDESMLTGESLPVPKEKGFPVFAGTVNWDGPLKIKATTTGPSSTIAKIVRMVEDAQAREAPV
QRLADSIAGPFVYTVMTLSAATFSFWYYIGTHIFPEVLLNDISGPDGDSLLLSLKLAVDVLVVSCP
CALGLATPTAILIGTSLGAKRGLLIRGGDVLERLAGIDAIVLDKTGTLTKGRPVVTSIASLAYEEA
EILRLAAAVEKTALHPIANAIMEEAELLKLDIPATSGQLTEPGFGCLAEVDGCLVAVGTLDWVHNR
FETKASSTELTDLGNHLEFVSSSEASSNHSKSIAYVGREGEGIIGAIAVSDVLRDDAKATVDRLQQ
EEILTFLLSGDRKEAVESIGRTVGIRSENIKSSLTPHEKAGIITALQGEGRRVAMVGDGINDAPSL
AAADVGVAMRTNSKESAASDAASVVLLGNRLSQYPYGLFASHHYLKIMSFNIVFVDGIM
>OsHMA9
MAHLQLSAVAGGGRPAAAGGGGDEMEDVRLLDSYDEEMGGGAAAAAAGEEEEAHVRVTGMTCSACT
SAVEGAVSARRGVRRVAVSLLQNRAHVVFDPALLKVEDIIEAIEDAGFDAEIIPDTAISQPKAQKT
LSAQFRIGGMTCANCVNSVEGILKRLSGVKGAVVALATSLGEVEYDPSVINKDEIVEAIEDAGFEA
AFLQSSEQDKILLGLTGLHTERDVNVLHDILKKMIGLRQFDVNATVSEVEIIFDPEAVGLRSIVDA
IETGSNGRLKAHVQNPYARGASNDAHEAAKMLHLLRSSLFLSIPVFFIRMVCPHIPFIRSILMMHC
GPFHMGDLLKWILVSIVQFVVGKRFYIAAYRALRHGSTNMDVLVVLGTTASYVYSVCALLYGAFTG
FHPPIYFETSAMIITFVLFGKYLEVLAKGKTSDAIKKLVELVPATALLLLKDKEGKYTEEREIDAL
LVQPGDILKVLPGSKVPADGVVVWGTSHVNESMITGESAPIPKEVSSAVIGGTMNLHGVLHIQANK
VGSETVLSQIISLVETAQMSKAPIQKFADYVASIFVPIVITLSMITFLVWFLCGWVGAYPNSWISG
TSNCFVFSLMFAIAVVVIACPCALGLATPTAVMVATGVGANHGVLVKGGDALERAQNVNYVIFDKT
GTLTQGKAVVTTAKVFSGMDLGDFLTLVASAEASSEHPLAKAIVEYAFHFHFFGKLPTSKDGIEQR
KEDRLSQLLLQVEDFSALPGKGVQCLINGKRVLVGNRTLVTENGVNVPPEAENFLVDLELNAKTGI
LVSYDDDFVGLMGITDPLKREAAVVVEGLKKMGVHPVMLTGDNWRTAKAVAKEVGIEDVRAEVMPA
GKADVVRSLQKDGSIVAMVGDGINDSPALAAADVGMAIGGGTDIAIEAADYVLVRNNLEDVITAID
LSRKTFSRIRWNYFFAMAYNVVAIPVAAGALFPFTRLQMPPWLAGACMAFSSVSVVCSSLLLRRYR
KPRLTTVLQITVE
>OsALA1
RKVSWGGAMEMQHSPSSLEIGVVSSSQPQEKPNRPQRHDPRLIYINDPNRTNDRYEFTGNEIRTSK
YTLITFLPKNLFIQFHRLAYVYFLVIAALNQLPPLAVFGRTASLFPLLFVLFVTAIKDGYEDWRRH

RSDRNENNREALVLQSGDFRLKTWKNICAGEVVKIHSNETMPCDMVLLGTSDPNGIAYIQTMNLDG
ESNLKTRYARQETMSMISDGSYSGLIKCEQPNRNIYEFTATMELNSHRIPLGQSNIVLRGCQLKNT
EWIVGVVVYAGQETKAMLNSTISPSKSSNLESYMNRETLWLSAFLLITCSVVATGMGVWLFRNSKN
LDALPYYRRKYFTFGRENRKDFKFYGIALEIFFSFLSSVIIFQIMIPISLYITMELVRVGQSYFMI
GDTRMYDSSSGSRFQCRSLNINEDLGQIRYIFSDKTGTLTQNKMEFHQASIYGKNYGSPLQVTESS
RQQGSKSKSGVNVDAELIALLSQPLVGEERLSAHDFFLTLAACNTVIPVSTENSLDLVNEINEIGR
IDYQGESPDEQALVTAASAYGYTLVERTTGHIVVDVQGEKIRLDVLGLHEFDSVRKRMSVVVRFPD
NIVKVLVKGADTSMLSILRREDDDELHNSLHAKIRETTENHLSGYSSEGLRTLVIGSKNLTDAEFG
EWQERYEEASTSMTERSAKLRQAAALVECNLTLLGATGIEDKLQDGVPEAIESLRQAGIKVWVLTG
DKQETAISIGLSCRLLTQNMHLIVINGSSEFECRRLLADAKAKFGINDKSEYSEKVANFADTDLAL
VIDGSSLVYILEKDLESELFDLATSCKVVICCRVAPLQKAGIVDLIKSRTSDMTLAIGDGANDVSM
IQMADVGVGICGQEGRQAVMASDFAMGQFRFLKRLLLVHGHWNYQRIAYMILYNFYRNAVFVLMLF
WYILHTAYSATLALTDWSSVFYSLIYTSIPTVVVGILDKDLSHNTLLHYPRLYETGLQNEGYNLTL
FWITMLDTLWQSLVLFYVPFFTYNISTMDIWSMGSLWTIAVVILVNIHLAMDIQRWVLITHLAVWG
SIAATFLCMVLIDSIPIFPNYGTIYNMAASRTYWLSVCLIIVLGLLPRFLCKVIYQTFWPSDIQIA
REAELLKKLPRQLGSRPASDIS

>OsALA10

MKRFVYINDDSWQDSYCDNRISNTKYNLWNFLPKNLWEQFRRFMNQYFLLIACLQLWSSITPVSPA
TTWGPLAIIFIVSASKEAWDDYNRYLSDKKANGREVLVVKDGNHRQIKAQDIHVGNIVWLYQNDEI
PCDLVLIGTSDPQGICYVETAALDGETDLKTRIVPSICANLSPDQLGRVKGVVECSNPDNDIRRFD
ANMRLFPPIIDSEKCPLTINNTLLQSCYLRYTEWACGVAVYTGNQTKSGMSRGTAEPKLTAADAMI
DKLTVAIFMFQIVVVLVLGFAGNIWKKNQGLKQWYLLYPVEGPWYDFLIIPLRFELLCSIMIPISV
KVTLDLSKGVYAKFIDWDEQMFDRETSYISVSFSTAISEDLGQVEYVLSDKTGTLTENRMIFRRCC
ISDILYGENNEDALKDARLLDAVSRNDPDIVKFLMVMALCNTVVPIKSNDGTITYQAQSQDEEALV
TAASKLNMVLVSKDSNTAEISFNGSKFYYDLLDILEFTSDRKRMSAVVKDVQSGKILLLSKGADEA
ILPRQQIRTYLETVEMYSQLGLRTLCLGWRELEEDEYKDWSKTFQDASCSLENRERKIAEVCHRLE
QDLQILGVSAIEDRLQDGVPETIKLLKSAGINVWMLTGDKQHTAIQIGLLCNLIAPEPNGQLLSIN
GKTEHDVLRSLERALSTMKSMSVTKDCAFVLDGWALEIILKHSKESFTKLAMLSRTAICCRMTPLQ
KAQLVGLLKSVGYLTLAIGDGGNDVRMIQEANIGVGISGREGLQAARAADYSIGKFKFLKRLILVH
GRYSYNRTAFISQYSFYKSLLICFIQILFSFISGLSGTSLFNSISLMAYNVFYTSLPVTTIIFDKD
ISEETVLQYPQILLYSQSGRLLNPTTFAGWFGRSVYHALVVFLTTICAYSDEKSEIEELSMVALSG
CIWLQAFVVTLDTNSFTYPQIILIWGNFIAFYMINLIVSAVPTLQMYTIMFRLCSQPSYWITMGLI
VAVGMGPVLALRYFRNMFRPNAINILQQIEQSNGHTHTTRNMESRIISAGSYLTHLLADSRRNRGA
TYQPLLSDSVASDG

>OsALA2

MASGRPLLDASPRPTQQPPASSLLPPPQPEPPLRADRLAFSLEVPDPFRREPDPSSAASQRGEEEG
GGEEESRAVVVGEPSSSEAAAGFAGNGVRTAKYSVLTFLPRNLFEQFRRLSYVYFLAITVLNQLPQ
VAVFGRGASVLPLAFVLFVTAVKDAYEDLRRHRSDRQENNRLARVLLAPPAAGEFAPKKWKHIRVG
DVVRVASSETLPADMVLLATSDPSGVAHVQTVNLDGETNLKTRYAKQETQLRFSQDGGIGGVLHCE
RPNRNIYGFQANLEIDGKRVSLGPSNIVLRGCELKNTTWAIGVVVYAGKETKVMLNSSGAPSKRSR
LETQLNRETVILSIMLIGMCTTASVLAGIWILNHRGDLEFTQFFREKDYTTGKNYNYYGMGMQIFI
TFLMAVIVYQVIIPISLYISMELVRLGQAYFMGADRDLYDESSRSKFQCRALNINEDLGQIKYVFS
DKTGTLTENKMEFQCASIRGVDYCSGKDSCGYSVVVDDLLWTPKMAVKIDHRLLKLLRGGGTDEET
KLVLEFFLALAACNTIVPLVLDTRDSKQKLIDYQGESPDEQALVYAAASYGIVLVERTSGYVVIDV
LGDRQRFDILGLHEFDSDRKRMSVIVGCPDKTVKLYVKGADSSLFGITKNSLDLDIVRATEAHLHK
YSSFGLRTLVIGMRELSQPEFEEWQLAYENASTSVLGRGNLLRSVAANIENNIRILGATGIEDKLQ
DGVPETIESLRQADIKVWILTGDKQETAISIGYSCKLLTNDMTQIVINNNSKESCKRSLEEAHATI
KKLRIASTGTQSPELASESAGVTLALIVDGNSLVYILETELQEELFKVARECSVVLCCRVAPLQKA
GIVALIKNRTDDMTLAIGDGANDVSMIQMADVGVGISGQEGRQAVMASDFAMGQFRFLVPLLLVHG

HWNYQRMSYMILYNFYKNATFVLVLFWYVLYTAFTLTTAITEWSSLLYTVLYTSLPTIVVGILDKD
LSKETLLAYPKLYGSGQRDEKYNVNLFVLNMLEALWQSLVVFYMPYFAYRQSTIDMSSLGDLWALA
PVIVVNMLLAMDIFRWNWIVHAFVWGTIAATTICLFVIDSIWFLPGYGAIFHIMGTGLFWLLLLII
VVAAMVPHFVIKAFTEYFTPSDIQVAREIEKFENVNQVNRSEVPMTRLHDPRR
>OsALA3
MAEEHDHHGSSRHMSASQKELGDEDARVVRVGDAERTNEQLEFAGNAVRTAKYSPLTFLPRNLFEQ
FHRLAYVYFLVIAVLNQLPQLAVFGRGASVMPLAFVLTVTAVKDAYEDWRRHRSDRAENGRLAAVL
LSPGAGTHFAPTKWKHVRVGDVVRVYSDESLPADMVLLATSDPTGVAYVQTLNLDGESNLKTRYAK
QETLTTPPEQLTGAVIRCERPNRNIYGFQANLELEGESRRIPLGPSNIVLRGCELKNTTWAIGVVV
YAGRETKAMLNNAGAPTKRSRLETQMNRETLFLSAILVVLCSLVAALSGVWLRTHKADLELAQFFH
KKNYVSDDKNANYNYYGIAAQIVFVFLMAVIVFQIMIPISLYISMELVRLGQAYFMIRDTTLYDAS
SNSRFQCRALNINEDLGQVKCVFSDKTGTLTQNKMEFRCASVGGVDYSDIARQQPVEGDRIWVPKI
PVNVDGEIVELLRNGGETEQGRYAREFFLALVTCNTIVPLILDGPDPKKKIVDYQGESPDEQALVS
AAAAYGFVLVERTSGHIVIDVLGEKQRFDVLGLHEFDSDRKRMSVIIGCPDKTVKLFVKGADNSMF
GVIDKTMNPDVVRATEKHLHAYSSLGLRTLVIGVRELSQEEFQEWQMAYEKASTALLGRGGLLRGV
AANIEQNLCLLGASGIEDKLQDGVPEAIEKLREAGIKVWVLTGDKQETAISIGFSCKLLTREMTQI
VINSNSRESCRKSLDDAISMVNKLRSLSTDSQARVPLALIIDGNSLVYIFDTEREEKLFEVAIACD
VVLCCRVAPLQKAGIVDLIKKRTSDMTLAIGDGANDVSMIQMADVGIGISGQEGRQAVMASDFAMG
QFRFLVDLLLVHGHWNYQRMGYMILYNFYRNATFVFVLFWYVLHTGFTLTTAITEWSSVLYSVIYT
AVPTIVVAILDKDLSRRTLLKYPQLYGAGQREESYNLRLFIFVMLDSIWQSLAVFFIPYLAYRKST
IDGASLGDLWTLAVVILVNIHLAMDVIRWNWITHAAIWGSIVATLICVMVIDSIPILPGFWAIYKV
MGTGLFWALLLAVIVVGMIPHFVAKAIREHFLPNDIQIAREMEKSQDSHDVTHPEIQMSTVARA
>OsALA4
MATGGGDGRRRGRRRSKMRLSRLYSFACGRRPTAVDDESSSRIGGPGFTRVVNANGGGGIPEYGYR
SNSVSTTKYNVVTFVPKSLLEQFRRVANIYFLISACLTYTNLAPYTSASAVAPLVLVLLATMVKEA
IEDWRRKQQDTEVNNRKTKVLQDGAFHSTKWMNLQVGDIVKVEKDEFFPADLILLSSSYEDAICYV
ETMNLDGETNLKLKQSLEASSGLQEDDSFNSFRAVIRCEDPNPHLYSFVGNIEIEEQYPLSPQQIL
LRDSKLRNTEYVYGVVIFTGHDTKVMQNAMKAPSKRSKIERKMDRIIYLLLSALVLISVIGSVFFG
ITTRDDLQDGRPKRWYLRPDDSTIYFKPTKAAISAILHFFTAMMLYGNFIPISLYISIEIVKLLQA
LFINQDIHMYHEETDTPAHARTSNLNEELGQVDTILTDKTGTLTCNSMEFIKCSIAGTAYGRGITE
VERAMAKRKGSPLIADMASNTQGSQAAIKGFNFTDERVMNGNWVSQPHSGVIQMFLRLLAVCHTCI
PEVDEESGTISYEAESPDEAAFVVAARELGFTFYQRTQTGVFLHELDPSSGKQVDRSYKLLHVLEF
NSARKRMSVIVRNEEGKIFLFSKGADSVMFERLSSSDCAYREVTXDHINEYADAGLRTLVLAYRQL
DEAEYANFDRKFTAAKNSVSADRDEMIEEAADLLERKLILLGATAVEDKLQKGVPECIDKLAQAGI
KIWVLTGDKMETAINIGYACSLLRQGMTQITITLEQPDIIALEKGGGDKAAVAKASKENVVKQINE
GKKRIDGSVVGEAFALIIDGKSLTYALEEDAKGALMDLAVGCKSVICCRSSPKQKALVTRLVKEST
GKVSLAIGDGANDVGMIQEADIGVGISGAEGMQAVMASDVSIAQFRFLERLLLVHGHWCYSRISAM
ICYFFYKNITFGVTLFLYEAYTSFSGQTFYNDWALSTYNVFFTSLPVIAMGVFDQDVSARFCLRYP
MLYQEGPQNLLFRWSRLLGWMAYGVASGVIIFFLTSAALQHQAFRRGGEVVDLAILSGTAYTCVVW
AVNAQMTVTANYFTLVQHACIWGSVALWYVFLLAYGAITPAFSTNYFMLFTDGLAAAPSYWVVTLL
VPAAALLPYFTYSAAKTRFFPDYHNKIQWLQHRGSNADDPEFGHALRQFSVRSTGVGVSARRDARD
LHLPPPSQSHSHSQTTST
>OsALA5
MAGGRRSRTSRRLKLKLSALYTFALCSKGSGEDHSSRIGTTGFSRVVYVNEPDRHEEEGFRYQPNE
VSTTKYSLVTFIPKSLFEQFRRVANFYFLVSGILALTPLAPYTAVSALLPLCVVIAATMAKEGIED
WRRKHQDHELNNRTVKVHRGDGDFEEKKWKDIKVGDVIKVEKDNFFPADLVLLSSNYPDGICYVET
MNLDGETNLKIKQALDVTLHLEEDNSFVNLRQTIKCEDPNANLYSFIGTMEWKDKQYNLSPQQLLL
RDSKLRNTDYIYGAVIFAGHDTKVMQNATDPPSKRSKIEKRMDKIIYVLMSSLLVIALLGSVLFGI
WTKEDLMNGEMKRWYLRPDDSTIFYDPKRAALASFFHLLTALMLYSYFIPISLYISIEMVKILQAL

FINQDIEMYHEESDKPTHARTSNLNEELGQVDTVLSDKTGTLTCNMMEFIKCSIAGIAYGQGVTEV
EKAMALRKGSVLGDGIENIEHTDQKNDGSPHIKGFNFKDPRIMDGNWIHEPNSDMIRDFFRLLAIC
HTCIPEEDEETHKVSYEAESPDEAAFVIAARELGFEFYHRAQSSIVVHERDPITNIVKDRKYELLN
VLEFSSSRKRMSVIVKEPEGRILLFSKGADSVMFKRLAPTGRKFEEETKRHINEYSDSGLRTLVLA
YRFLDENEYMKFSEKFNTARTSVSADRDEKVEAAAESIERDLLLLGATAVEDKLQKGVPECIDKLA
QAGIKIWVLTGDKMETAINIGFACSLLRQGMTQIIVTLEAPDIIALEKNGDKESIARESKQRVMDQ
IEDGIKQIPPPSQSNTESFALIIDGKSLTYALEDDVKFKFLDLALKCASVICCRSSPKQKALVTRL
VKHTNRVTLAIGDGANDVGMLQEADIGVGISGVEGMQAVMASDFAIAQFRFLERLLLIHGHWCYRR
ISVMICYFFYKNVTFGVTIFLYEAFASFSGKPAYNDWFLSLYNVIFTSLPVIALGVFDQDVSQRLC
LQYPGLYQEGVQNILFSWRRILGWMANGVINAILIFYFCTTAFGIQAFRQDGQVAGLDALGVLMYT
CVVWVVNCQMALSVNYFTIIQHIFIWGSIAVWYLFLLAYGAVDPRFSKSAYMVFIEQVAPALSYWL
VTLFAVMATLIPYFCYAAIQIRFFPMFHNKIQWKRHLGKAEDPEVARQLSSRHRTSSHQRMVGISA
RRDGKAMQVTKETELQVQG
>OsALA6
MRPASASAADERPLVELTSAAATAPASTETSTFSSAPGFTRAVRCSGAGSSSSSSSSSDEGGGGVY
PGNAISTTKYTAASFVPKSLFEQFRRAANCFFLVVACVSFSPLAPYRAVSVLLPLVVVVGAAMAKE
PVEDWRRKQQDIEVNSRKVEVYDGTQSFHQTEWKKLQVGDIVKVKKDEFFPADLVLLSSSYEDGIC
YVETMNLDGETNLKRKQSLDVTAGLNEDHSFHTFKAFIQCEDPNEKLYSFLGTLHYNGQQYPLSPQ
QILLRDSKLRNTNQIYGIVIFTGHDTKVMQNAMEPPSKRSSVERRMDKIIYLLFVILFAIASFGSV
MFGIRTRAELSAGNYAWYLRPDNSTMYFDPNRATLAAICHFLTSLMLYVCLVPISLYISIEIVKVL
QSTFINQDQNMYCEESDKPARARTSNLNEELGQVHTILSDKTGTLTCNSMEFLKCSIAGVAYGNRP
IEVQMPYGGIEEECVDIGQKGAVKSVRPVKGFNFTDDRLMNGQWSKECHQDVIEMFFRVLAVCHTA
IPVADRTSGGMSYEAESPDEGALVAAARELGFEFYHRSQTSISVHEYDPVFGRKVDRTYKLLNTLE
FSSARKRMSVIVSTEEGRLFLFCKGADSVILERLSKDNSKACLTNTKCHIDEYSEAGLRTLALAYR
ELTEDEYVAWNMEYSAAKNSVHNDHDVAVEKASENIEKDLVLLGATAVEDRLQKGVPECIHKLAQA
GIKIWILTGDKLETAVNIGYACNLLRKGMEEVYITLDNPGTNVPEEHNGESSGMAPYEQIGRKLED
ARRQILQKGTSAPFALIIDGNALTHALMGGLKTAFLDLAVDCASVLCCRISPKQKALITRLVKNRI
RKTTLAIGDGANDVGMLQEADIGVGISGAEGMQAVMASDFAIAQFRFLERLLLVHGHWCYRRIAAM
ICYFFFKNITFGFTLFWFEAHAMFSAQPGYNDWFISFYNVAFTSLPVIALGVFDKDVSSRVCLEVP
SLHQDGVNNLFFSWSRILSWMLNGVCCSIIIYFGALHAVLIQAVRQDGHVAGFDILGVTMYTCVVW
TVNCQLALYISYFTWIQHFVIWGSILIWYTFLVIYGSFPPTISTSAYHVFWEACASSPLYWLSTLV
IVVTALIPYFLYKITQSLFCPQHCDQVQRPNSKELVAQ
>OsALA7
NNEAAGGGPAAEGGSAVGGPGFTRVVHCNNSAVHRRKPLKYPTNYISTTKYNILTFLPKAIFEQFR
RVANLYFLLTAILSLTPVCPFSAVSMIAPLAFVVGLSMIKEGVEDWRRFMQDMKVNNRKVAVHKGE
GEFEYRHWEDLAVGDVVKVEKDQFFPADLLLLSSSYEDGICYVETMNLDGETNLKVKRSLEVTLPL
EEDESFKDFQGLIRCEDPNPSLYTFIGNLEYERQIYAIDPFQILLRDSKLRNTSFIYGVVIFTGHD
SKVMQNSTESPSKRSTIEKKMDLIIYILFTVLVLISLISSIGFAVRIKYDLPNWWYLQPEKSNKLD
DPTRPALSGIFHLITALILYGYLIPISLYVSIELVKVLQAHFINQDLHMFDEDTGNTAQARTSNLN
EELGQVHTILSDKTGTLTCNQMDFLKCSIAGVSYGVGSSEVELAAAKQMASGDDGQDIHVQDVWEN
NEDEIQLVEGVTFSVGRTRKSSIKGFSFEDDRLMQGNWTKEPNSSTILMFFRILAVCHTAIPEVNE
ATGALTYEAESPDEGAFLVAAREFGFEFFKRTQSSVFVREKFSSSNGPVEREFKILNLLEFNSKRK
RMSVILKDEDGQILLFCKGADSIIFDRLAKNGRMIEADTSKHLNDYGEAGLRTLALSYRVLDESEY
SSWNAEFLKAKTSIGPDRELQLERVSELIERDLILVGATAVEDKLQSGVPQCIDRLAQAGLKIWVL
TGDKMETAINIGYACSLLRQGMRRICLSIPTDDQVAQDANKAAKESLMSQIANGSQMVKLEKDPDA
AFALVIDGKALTFALEDDMKHMFLNLAIECASVICCRVSPKQKALVTRLVKEGIGKTTLAIGDGAN
DVGMIQEADIGVGISGVEGMQAVMASDFSISQFRFLERLLVVHGHWCYKRIAQMICYFFYKNIAFG
LTIFYFEAFAGFSGQSVYDDWFMLLFNVVLTSLPVISLGVFEQDVSSEICLQFPALYQQGPRNLFF
DWYRILGWMANGLYSSLAIFFLNICIFYDQAIRSGGQTADMAAVGTTMFTCIIWAVNMQIALTMSH

FTWIQHLFVWGSVGTWYLFIIVYGSALRSRDNYQILLEVLGPAPLYWAATLLVTAACNMPYLIHIS
YQRLCNPLDHHVIQEIKYLKKDVEDQTMWKRERSKARQRTKIGFTARVDAKIKQIRGKLHKKAPSL
TIHT
>OsALA8
MVRVATARLGGEPSPRGGAGNSAFGRGESSRTARLGGGGASLRRQPQPQAPSVRTICCNDREANAP
VGYKGNSVSTTKYNVLTFLPKGLFEQFRRVANLYFLMISILSTTPISPVHPVTNVVPLSLVLLVSL
IKEAFEDWKRFQNDMSINNAHVDVLQGQKWETTPWKRLQVGDIVRIKQDGYFPADLLFLSSTNPDG
VCNIETANLDGETNLKIRKALEKTWDYKNPEKAFEFKGEIQCEQPNNSLYTFTGNLIVDKQTMPLS
PNQVLLRGCSLRNTEYIVGVVIFTGHETKVMMNSMNVPSKRSTLEKKLDKLILALFATLFTMCVIG
AIGSGVFINEKYFYLGLRGKVEDQFNPKNKFVVTILTMFTLITLYSTIIPISLYVSIEMIKFIQCT
QFINNDLHMYHAESNTPALARTSNLNEELGQVEYIFSDKTGTLTRNLMEFFKCSIAGEIYGTGITE
IEKGGAERAGIKIDGDEGKRSGAAVHEKGFNFDDARIMCGAWRNEPNPEACKEFFRCLALCHTVLP
EGEETPEKISYQAASPDEAALVAASKNFGFFFYRRTPTTVIVRESHVERMGSIQDVAYEILNVLEF
NSTRKRQSVVCRFPNGRLVLYCKGADNVVYERLADGNNDIKKISREHLEQFGSAGLRTLCLAYRDL
SREQYESWNEKFIQAKSSLRDRDKKLDEVAELIEKDLVLVGCTAIEDKLQEGVPACIQTLSAAGIK
IWVLTGDKMETAINIAYACSLVNNDMKQFIISSETDVIREAEDRGDPVEIARVIKESVKQSLKSYH
EEARGSLISTPGQKLALIIDGRCLMYALDPTLRVDLLGLSLICHSVVCCRVSPLQKAQVASLVKKG
AHKITLSIGDGANDVSMIQAAHVGIGISGQEGMQAVMASDFAIAQFRYLTDLLLVHGRWSYLRLCK
VITYFFYKNLTFTLTQFWFTFQTGFSGQRFYDDWFQSLYNVIFTALPVIMVGLFDKDVSASLSKKY
PKLYQEGIRNTFFKWRVIAVWAFFAFYQSIVFYYFTAAASRYGHGSSGKILGLWDVSTMAFTCVVV
TVNLRLLMSCNSITRWHYISVAGSITAWFMFIFIYSAIMTSFDRQENVYFVIYVLMSTFFFYLTLL
LVPIIALFGDFLYLSIQRWFFPYDYQVIQEMHRDEPHEYSRIQLPETSHLSPEEARSYAISMLPRE
SSKHTGFAFDSPGYESFFASQQGVGVPHKPWDVARRASMKQRQKTGGS
>OsALA9
MKRFVYINDESYQNDYCDNRISNTKYTLLNFLPKNLWEQFRRFMNQYFLLIACLQLWSLITPVNPA
STWGPLIFIFAVSATKEAWDDYNRYISDKQANEKEVWIVKNGTRKHIQAQDIRVGNIVWIRENEEV
PCDLVLIGTSDSQGICHVETAALDGEIDLKTRVIPLTCVGLDSEQLHKIKGVIECPNPDKDIRRLD
ANIRLFPPFIDNDICPLTISNTLLQSCYLRNTEWACGFNFSGNETKLGMSRGVPEPKLTSMDAMID
KLTGAIFLFQIAVVVVLGSAGNVWKDTEARKQWYVKYDDDEPWYQILVIPLRFELLCSIMIPISIK
VSLDFVKSLYAKFIDWDEEMYDHETDTPAHAANTAISEDLGQVEYILTDKTGTLTENKMIFRRCCI
GGTFYGNESGDALRDVELLNAVANNSHVIKFLTVMTLCNTVIPIKRLYFHLSSGAILYKAQSQDED
ALVNAASNLHMVLVNKNGNTAEIHFNRRVVQYEILDILEFTSDRKRMSVVVLDCESGKIFLLSKGA
DEAIIPCAYSGQRIKTFVDAVDKYAQLGLRTLCLGWRELESEEYLEWSRSFKEANSALIDREWKVA
EVCQKLEHSLEILGVSAIEDRLQAGVPETIEILRQSGINFWMLTGDKQSTAIQIALLCNLISSEPK
GQLLYINGRTVDEVARSLERVLLTMRILLLNLRLLAFVVDGWALEIILSRYNEAFTELAALSKTAI
CCRVTPSQKAQLVKLLKSCDYRTLAIGDGGNDVRMIQQADIGVGISGREGLQAARAADYSVGKFRF
LKRLILVHGRYSYNRTAFLSQYSFYKSLLICFIQILFSFLSGIAGTSLFNSVSLMAYNVFYTSIPV
LTTVLDKDLSEKTVMQNPEILLYCQAGRLLNPSTFAGWFGRSLYHAIVVFLITVHAYANEKSEMEE
LSMVALSGSIWLQAFVVTLEMNSFTFVQFLAIWGNFIAFYIINFFISSIPSAGMYTIMFRLCRQPT
YWVTLLLISGVGMGPVLALKYFRYTYRPSAINILQKAERSRGPMYTLVNLESQLRSDMENTNISIS
TPPVKNKNSVMSPXFXSHPFLPEDLXCHPHLTYFSQHNRGPHPLSPEISKQN
>AtECA1
MGKGSEDLVKKESLNSTPVNSDTFPAWAKDVAECEEHFVVSREKGLSSDEVLKRHQIYGLNELEKP
EGTSIFKLILEQFNDTLVRILLAAAVISFVLAFFDGDEGGEMGITAFVEPLVIFLILIVNAIVGIW
QETNAEKALEALKEIQSQQATVMRDGTKVSSLPAKELVPGDIVELRVGDKVPADMRVVALISSTLR
VEQGSLTGESEAVSKTTKHVDENADIQGKKCMVFAGTTVVNGNCICLVTDTGMNTEIGRVHSQIQE
AAQHEEDTPLKKKLNEFGEVLTMIIGLICALVWLINVKYFLSWEYVDGWPRNFKFSFEKCTYYFEI
AVALAVAAIPEGLPAVITTCLALGTRKMAQKNALVRKLPSVETLGCTTVICSDKTGTLTTNQMAVS
KLVAMGSRIGTLRSFNVEGTSFDPRDGKIEDWPMGRMDANLQMIAKIAAICNDANVEQSDQQFVSR

```
GMPTEAALKVLVEKMGFPEGLNEASSDGDVLRCCRLWSELEQRIATLEFDRDRKSMGVMVDSSSGN
KLLLVKGAVENVLERSTHIQLLDGSKRELDQYSRDLILQSLRDMSLSALRCLGFAYSDVPSDFATY
DGSEDHPAHQQLLNPSNYSSIESNLIFVGFVGLRDPPRKEVRQAIADCRTAGIRVMVITGDNKSTA
EAICREIGVFEADEDISSRSLTGIEFMDVQDQKNHLRQTGGLLFSRAEPKHKQEIVRLLKEDGEVV
AMTGDGVNDAPALKLADIGVAMGISGTEVAKEASDMVLADDNFSTIVAAVGEGRSIYNNMKAFIRY
MISSNIGEVASIFLTAALGIPEGMIPVQLLWVNLVTDGPPATALGFNPPDKDIMKKPPRRSDDSLI
TAWILFRYMVIGLYVGVATVGVFIIWYTHSSFMGIDLSQDGHSLVSYSQLAHWGQCSSWEGFKVSP
FTAGSQTFSFDSNPCDYFQQGKIKASTLSLSVLVAIEMFNSLNALSEDGSLVTMPPWVNPWLLLAM
AVSFGLHFVILYVPFLAQVFGIVPLSLNEWLLVLAVSLPVILIDEVLKFVGRCTSGYRYSPRTLST
KQKEE
>AtECA2
MEEEKSFSAWSWSVEQCLKEYKTRLDKGLTSEDVQIRRQKYGFNELAKEKGKPLWHLVLEQFDDTL
VKILLGAAFISFVLAFLGEEHGSGSGFEAFVEPFVIVLILILNAVVGVWQESNAEKALEALKEMQC
ESAKVLRDGNVLPNLPARELVPGDIVELNVGDKVPADMRVSGLKTSTLRVEQSSLTGEAMPVLKGA
NLVVMDDCELQGKENMVFAGTTVVNGSCVCIVTSIGMDTEIGKIQRQIHEASLEESETPLKKKLDE
FGSRLTTAICIVCVLVWMINYKNFVSWDVVDGYKPVNIKFSFEKCTYYFKIAVALAVAAIPEGLPA
VITTCLALGTRKMAQKNAIVRKLPSVETLGCTTVICSDKTGTLTTNQMSATEFFTLGGKTTTTRVF
SVSGTTYDPKDGGIVDWGCNNMDANLQAVAEICSICNDAGVFYEGKLFRATGLPTEAALKVLVEKM
GIPEKKNSENIEEVTNFSDNGSSVKLACCDWWNKRSKKVATLEFDRVRKSMSVIVSEPNGQNRLLV
KGAAESILERSSFAQLADGSLVALDESSREVILKKHSEMTSKGLRCLGLAYKDELGEFSDYSSEEH
PSHKKLLDPSSYSNIETNLIFVGVVGLRDPPREEVGRAIEDCRDAGIRVMVITGDNKSTAEAICCE
IRLFSENEDLSQSSFTGKEFMSLPASRRSEILSKSGGKVFSRAEPRHKQEIVRMLKEMGEIVAMTG
DGVNDAPALKLADIGIAMGITGTEVAKEASDMVLADDNFSTIVSAVAEGRSIYNNMKAFIRYMISS
NVGEVISIFLTAALGIPECMIPVQLLWVNLVTDGPPATALGFNPADIDIMKKPPRKSDDCLIDSWV
LIRYLVIGSYVGVATVGIFVLWYTQASFLGISLISDGHTLVSFTQLQNWSECSSWGTNFTATPYTV
AGGLRTIAFENNPCDYFTLGKVKPMTLSLTVLVAIEMFNSLNALSEDNSLLTMPPWRNPWLLVAMT
VSFALHCVILYVPFLANVFGIVPLSFREWFVVILVSFPVILIDEALKFIGRCRRTRIKKKIKTM
>AtECA3
MEDAYARSVSEVLDFFGVDPTKGLSDSQVVHHSRLYGRNVLPEEKRTPFWKLVLKQFDDLLVKILI
VAAIVSFVLALANGETGLTAFLEPFVILLILAANAAVGVITETNAEKALEELRAYQANIATVLRNG
CFSILPATELVPGDIVEVTVGCKIPADLRMIEMSSNTFRVDQAILTGESCSVEKDVDCTLTTNAVY
QDKKNILFSGTDVVAGRGRAVVIGVGSNTAMGSIHDSMLQTDDEATPLKKKLDEFGSFLAKVIAGI
CVLVWVVNIGHFSDPSHGGFFKGAIHYFKIAVALAVAAIPEGLPAVVTTCLALGTKKMARLNAIVR
SLPSVETLGCTTVICSDKTGTLTTNMMSVSKICVVQSAEHGPMINEFTVSGTTYAPEGTVFDSNGM
QLDLPAQSPCLHHLAMCSSLCNDSILQYNPDKDSYEKIGESTEVALRVLAEKVGLPGFDSMPSALN
MLSKHERASYCNHYWENQFKKVYVLEFTRDRKMMSVLCSHKQMDVMFSKGAPESIIARCNKILCNG
DGSVVPLTAAGRAELESRFYSFGDETLRCLALAFKTVPHGQQTISYDNENDLTFIGLVGMLDPPRE
EVRDAMLACMTAGIRVIVVTGDNKSTAESLCRKIGAFDNLVDFSGMSYTASEFERLPAVQQTLALR
RMTLFSRVEPSHKRMLVEALQKQNEVVAMTGDGVNDAPALKKADIGIAMGSGTAVAKSASDMVLAD
DNFASIVAAVAEGRAIYNNTKQFIRYMISSNIGEVVCIFVAAVLGIPDTLAPVQLLWVNLVTDGLP
ATAIGFNKQDSDVMKAKPRKVGEAVVTGWLFFRYLVIGVYVGLATVAGFIWWFVYSDGGPKLTYSE
LMNFETCALRETTYPCSIFEDRHPSTVAMTVLVVVEMFNALNNLSENQSLLVITPRSNLWLVGSII
LTMLLHVLILYVHPLAVLFSVTPLSWAEWTAVLYLSFPVIIIDELLKFLSRNTGMRFRFRLRKADL
LPKDRRDK
>AtECA4
MGKGGEDCGNKQTNSSELVKSDTFPAWGKDVSECEEKFGVSREKGLSTDEVLKRHQIYGLNELEKP
EGTSIFKLILEQFNDTLVRILLAAAVISFVLAFFDGDEGGEMGITAFVEPLVIFLILIVNAIVGIW
QETNAEKALEALKEIQSQQATVMRDGTKVSSLPAKELVPGDIVELRVGDKVPADMRVVALISSTLR
VEQGSLTGESEAVSKTTKHVDENADIQGKKCMVFAGTTVVNGNCICLVTDTGMNTEIGRVHSQIQE
```

AAQHEEDTPLKKKLNEFGEVLTMIIGLICALVWLINVKYFLSWEYVDGWPRNFKFSFEKCTYYFEI
AVALAVAAIPEGLPAVITTCLALGTRKMAQKNALVRKLPSVETLGCTTVICSDKTGTLTTNQMAVS
KLVAMGSRIGTLRSFNVEGTSFDPRDGKIEDWPTGRMDANLQMIAKIAAICNDANVEKSDQQFVSR
GMPTEAALKVLVEKMGFPEGLNEASSDGNVLRCCRLWSELEQRIATLEFDRDRKSMGVMVDSSSGK
KLLLVKGAVENVLERSTHIQLLDGSTRELDQYSRDLILQSLHDMSLSALRCLGFAYSDVPSDFATY
DGSEDHPAHQQLLNPSNYSSIESNLVFVGFVGLRDPPRKEVRQAIADCRTAGIRVMVITGDNKSTA
EAICREIGVFEADEDISSRSLTGKEFMDVKDQKNHLRQTGGLLFSRAEPKHKQEIVRLLKEDGEVV
AMTGDGVNDAPALKLADIGVAMGISGTEVAKEASDLVLADDNFSTIVAAVGEGRSIYNNMKAFIRY
MISSNIGEVASIFLTAALGIPEGMIPVQLLWVNLVTDGPPATALGFNPPDKDIMKKPPRRSDDSLI
TAWILFRYMVIGLYVGVATVGVFIIWYTHNSFMGIDLSQDGHSLVSYSQLAHWGQCSSWEGFKVSP
FTAGSQTFSFDSNPCDYFQQGKIKASTLSLSVLVAIEMFNSLNALSEDGSLVTMPPWVNPWLLLAM
AVSFGLHFVILYVPFLAQVFGIVPLSLNEWLLVLAVSLPVILIDEVLKFVGRCTSGYRYSPRTPSA
KQKEE
>AtACA1
MESYLNENFGDVKPKNSSDEALQRWRKLCWIVKNPKRRFRFTANLSKRSEAEAIRRSNQEKFRVAV
LVSQAALQFINSLKLSSEYTLPEEVRKAGFEICPDELGSIVEGHDLKKLKIHGGTEGLTEKLSTSI
ASGISTSEDLLSVRKEIYGINQFTESPSRGFWLFVWEALQDTTLMILAACAFVSLIVGILMEGWPI
GAHDGLGIVASILLVVFVTATSDYRQSLQFKDLDAEKKKIVVQVTRDKLRQKISIYDLLPGDVVHL
GIGDQIPADGLFISGFSVLINESSLTGESEPVSVSVEHPFLLSGTKVQDGSCKMLVTTVGMRTQWG
KLMATLSEGGDDETPLQVKLNGVATIIGKIGLFFAVITFAVLVQGLANQKRLDNSHWIWTADELMA
MLEYFAVAVTIVVVAVPEGLPLAVTLSLAFAMKKMMNDKALVRNLAACETMGSATTICSDKTGTLT
TNHMTVVKACICEQAKEVNGPDAAMKFASGIPESAVKLLLQSIFTNTGGEIVVGKGNKTEILGTPT
ETALLEFGLSLGGDFQEVRQASNVVKVEPFNSTKKRMGVVIELPERHFRAHCKGASEIVLDSCDKY
INKDGEVVPLDEKSTSHLKNIIEEFASEALRTLCLAYFEIGDEFSLEAPIPSGGYTCIGIVGIKDP
VRPGVKESVAICKSAGITVRMVTGDNLTTAKAIARECGILTDDGIAIEGPEFREKSDEELLKLIPK
LQVMARSSPMDKHTLVRLLRTMFQEVVAVTGDGTNDAPALHEADIGLAMGISGTEVAKESADVIIL
DDNFSTIVTVAKWGRSVYINIQKFVQFQLTVNVVALIVNFLSACLTGNAPLTAVQLLWVNMIMDTL
GALALATEPPQDDLMKRSPVGRKGNFISNVMWRNILGQSLYQLVIIWCLQTKGKTMFGLDGPDSDL
TLNTLIFNIFVFCQVFNEISSREMEKIDVFKGILKNYVFVAVLTCTVVFQVIIIELLGTFADTTPL
NLGQWLVSIILGFLGMPVAAALKMIPVGSH
>AtACA2
MESYLNENFDVKAKHSSEEVLEKWRNLCGVVKNPKRRFRFTANLSKRYEAAAMRRTNQEKLRIAVL
VSKAAFQFISGVSPSDYTVPEDVKAAGFEICADELGSIVESHDVKKLKFHGGVDGLAGKLKASPTD
GLSTEAAQLSQRQELFGINKFAESEMRGFWVFVWEALQDMTLMILGVCAFVSLIVGIATEGWPKGS
HDGLGIAASILLVVFVTATSDYRQSLQFRDLDKEKKKITVQVTRNGFRQKLSIYDLLPGDIVHLAI
GDQVPADGLFLSGFSVVIDESSLTGESEPVMVNAQNPFLMSGTKVQDGSCKMMITTVGMRTQWGKL
MATLTEGGDDETPLQVKLNGVATIIGKIGLFFAVVTFAVLVQGMFMRKLSTGTHWVWSGDEALELL
EYFAIAVTIVVVAVPEGLPLAVTLSLAFAMKKMMNDKALVRHLAACETMGSATTICSDKTGTLTTN
HMTVVKSCICMNVQDVANKGSSLQSEIPESAVKLLIQSIFNNTGGEVVVNKHGKTELLGTPTETAI
LELGLSLGGKFQEERKSYKVIKVEPFNSTKKRMGVVIELPEGGRMRAHTKGASEIVLAACDKVVNS
SGEVVPLDEESIKYLNVTINEFANEALRTLCLAYMDIEGGFSPDDAIPASGFTCVGIVGIKDPVRP
GVKESVELCRRAGITVRMVTGDNINTAKAIARECGILTDDGIAIEGPVFREKNQEELLELIPKIQV
MARSSPMDKHTLVKQLRTTFDEVVAVTGDGTNDAPALHEADIGLAMGIAGTEVAKESADVIILDDN
FSTIVTVAKWGRSVYINIQKFVQFQLTVNVVALVVNFSSACLTGSAPLTAVQLLWVNMIMDTLGAL
ALATEPPNDELMKRLPVGRRGNFITNAMWRNILGQAVYQFIVIWILQAKGKAMFGLDGPDSTLMLN
TLIFNCFVFCQVFNEISSREMEEIDVFKGILDNYVFVVVIGATVFFQIIIIEFLGTFASTTPLTIT
QWIFSIFIGFLGMPIAAGLKTIPV
>AtACA4

MSNLLRDFEVEAKNPSLEARQRWRSSVSIVKNRTRRFRNIRDLDKLADYENKKHQIQEKIRVAFFV
QKAALHFIDAAARPEYKLTDEVKKAGFSIEADELASMVRKNDTKSLAQKGGVEELAKKVSVSLSEG
IRSSEVPIREKIFGENRYTEKPARSFLMFVWEALHDITLIILMVCAVVSIGVGVATEGFPRGMYDG
TGILLSILLVVMVTAISDYKQSLQFRDLDREKKKIIVQVTRDGSRQEISIHDLVVGDVVHLSIGDQ
VPADGIFISGYNLEIDESSLSGESEPSHVNKEKPFLLSGTKVQNGSAKMLVTTVGMRTEWGKLMET
LVDGGEDETPLQVKLNGVATIIGKIGLSFAVLTFVVLCIRFVLDKATSGSFTNWSSEDALTLLDYF
AISVTIIVVAVPEGLPLAVTLSLAFAMKKLMSDRALVRHLAACETMGSSTCICTDKTGTLTTNHMV
VNKVWICDKVQERQEGSKESFELELSEEVQSTLLQGIFQNTGSEVVKDKDGNTQILGSPTERAILE
FGLLLGGDFNTQRKEHKILKIEPFNSDKKKMSVLIALPGGGARAFCKGASEIVLKMCENVVDSNGE
SVPLTEERITSISDIIEGFASEALRTLCLVYKDLDEAPSGELPDGGYTMVAVVGIKDPVRPGVREA
VQTCQAAGITVRMVTGDNISTAKAIAKECGIYTEGGLAIEGSEFRDLSPHEMRAIIPKIQVMARSL
PLDKHTLVSNLRKIGEVVAVTGDGTNDAPALHEADIGLAMGIAGTEVAKENADVIIMDDNFKTIVN
VARWGRAVYINIQKFVQFQLTVNVVALIINFVSACITGSAPLTAVQLLWVNMIMDTLGALALATEP
PNEGLMKRAPIARTASFITKTMWRNIAGQSVYQLIVLGILNFAGKSLLKLDGPDSTAVLNTVIFNS
FVFCQVFNEINSREIEKINVFKGMFNSWVFTWVMTVTVVFQVIIVEFLGAFASTVPLSWQHWLLSI
LIGSLNMIVAVILKCVPVESRHHHDGYDLLPSGPSSSNSA
>AtACA7
MESYLNSNFDVKAKHSSEEVLEKWRNLCSVVKNPKRRFRFTANLSKRYEAAAMRRTNQEKLRIAVL
VSKAAFQFISGVSPSDYKVPEEVKAAGFDICADELGSIVEGHDVKKLKFHGGVDGLSGKLKACPNA
GLSTGEPEQLSKRQELFGINKFAESELRSFWVFVWEALQDMTLMILGVCAFVSLIVGIATEGWPQG
SHDGLGIVASILLVVFVTATSDYRQSLQFRDLDKEKKKITVQVTRNGFRQKMSIYDLLPGDVVHLA
IGDQVPADGLFLSGFSVVIDESSLTGESEPVMVTAQNPFLLSGTKVQDGSCKMLVTTVGMRTQWGK
LMATLSEGGDDETPLQVKLNGVATIIGKIGLSFAIVTFAVLVQGMFMRKLSLGPHWWWSGDDALEL
LEYFAIAVTIVVVAVPEGLPLAVTLSLAFAMKKMMNDKALVRHLAACETMGSATTICSDKTGTLTT
NHMTVVKSCICMNVQDVASKSSSLQSDIPEAALKLLLQLIFNNTGGEVVVNERGKTEILGTPTETA
ILELGLSLGGKFQEERQSNKVIKVEPFNSTKKRMGVVIELPEGGRIRAHTKGASEIVLAACDKVIN
SSGEVVPLDDESIKFLNVTIDEFANEALRTLCLAYMDIESGFSADEGIPEKGFTCIGIVGIKDPVR
PGVRESVELCRRAGIMVRMVTGDNINTAKAIARECGILTDDGIAIEGPVFREKNQEEMLELIPKIQ
VMARSSPMDKHTLVKQLRTTFDEVVAVTGDGTNDAPALHEADIGLAMGIAGTEVAKEIADVIILDD
NFSTIVTVAKWGRSVYINIQKFVQFQLTVNVVALIVNFSSACLTGSAPLTAVQLLWVNMIMDTLGA
LALATEPPNNELMKRMPVGRRGNFITNAMWRNILGQAVYQFIIIWILQAKGKSMFGLVGSDSTLVL
NTLIFNCFVFCQVFNEVSSREMEEIDVFKGILDNYVFVVVIGATVFFQIIIIEFLGTFASTTPLTI
VQWFFSIFVGFLGMPIAAGLKKIPV
>AtACA8
MTSLLKSSPGRRRGGDVESGKSEHADSDSDTFYIPSKNASIERLQQWRKAALVLNASRRFRYTLDL
KKEQETREMRQKIRSHAHALLAANRFMDMGRESGVEKTTGPATPAGDFGITPEQLVIMSKDHNSGA
LEQYGGTQGLANLLKTNPEKGISGDDDDLLKRKTIYGSNTYPRKKGKGFLRFLWDACHDLTLIILM
VAAVASLALGIKTEGIKEGWYDGGSIAFAVILVIVVTAVSDYKQSLQFQNLNDEKRNIHLEVLRGG
RRVEISIYDIVVGDVIPLNIGNQVPADGVLISGHSLALDESSMTGESKIVNKDANKDPFLMSGCKV
ADGNGSMLVTGVGVNTEWGLLMASISEDNGEETPLQVRLNGVATFIGSIGLAVAAAVLVILLTRYF
TGHTKDNNGGPQFVKGKTKVGHVIDDVVKVLTVAVTIVVVAVPEGLPLAVTLTLAYSMRKMMADKA
LVRRLSACETMGSATTICSDKTGTLTLNQMTVVESYAGGKKTDTEQLPATITSLVVEGISQNTTGS
IFVPEGGGDLEYSGSPTEKAILGWGVKLGMNFETARSQSSILHAFPFNSEKKRGGVAVKTADGEVH
VHWKGASEIVLASCRSYIDEDGNVAPMTDDKASFFKNGINDMAGRTLRCVALAFRTYEAEKVPTGE
ELSKWVLPEDDLILLAIVGIKDPCRPGVKDSVVLCQNAGVKVRMVTGDNVQTARAIALECGILSSD
ADLSEPTLIEGKSFREMTDAERDKISDKISVMGRSSPNDKLLLVQSLRRQGHVVAVTGDGTNDAPA
LHEADIGLAMGIAGTEVAKESSDIIILDDNFASVVKVVRWGRSVYANIQKFIQFQLTVNVAALVIN
VVAAISSGDVPLTAVQLLWVNLIMDTLGALALATEPPTDHLMGRPPVGRKEPLITNIMWRNLLIQA
IYQVSVLLTLNFRGISILGLEHEVHEHATRVKNTIIFNAFVLCQAFNEFNARKPDEKNIFKGVIKN

RLFMGIIVITLVLQVIIVEFLGKFASTTKLNWKQWLICVGIGVISWPLALVGKFIPVPAAPISNKL
KVLKFWGKKKNSSGEGSL
>AtACA9
MSTSSSNGLLLTSMSGRHDDMEAGSAKTEEHSDHEELQHDPDDPFDIDNTKNASVESLRRWRQAAL
VLNASRRFRYTLDLNKEEHYDNRRRMIRAHAQVIRAALLFKLAGEQQIAFGSSTPAASTGNFDIDL
EKLVSMTRNQNMSNLQQYGGVKGVAEKLKSNMEQGINEDEKEVIDRKNAFGSNTYPKKKGKNFFMF
LWEAWQDLTLIILIIAAVTSLALGIKTEGLKEGWLDGGSIAFAVLLVIVVTAVSDYRQSLQFQNLN
DEKRNIQLEVMRGGRTVKISIYDVVVGDVIPLRIGDQVPADGVLISGHSLAIDESSMTGESKIVHK
DQKSPFLMSGCKVADGVGNMLVTGVGINTEWGLLMASISEDTGEETPLQVRLNGLATFIGIVGLSV
ALVVLVALLVRYFTGTTQDTNGATQFIKGTTSISDIVDDCVKIFTIAVTIVVVAVPEGLPLAVTLT
LAYSMRKMMADKALVRRLSACETMGSATTICSDKTGTLTLNQMTVVETYAGGSKMDVADNPSGLHP
KLVALISEGVAQNTTGNIFHPKDGGEVEISGSPTEKAILSWAYKLGMKFDTIRSESAIIHAFPFNS
EKKRGGVAVLRGDSEVFIHWKGAAEIVLACCTQYMDSNGTLQSIESQKEFFRVAIDSMAKNSLRCV
AIACRTQELNQVPKEQEDLDKWALPEDELILLAIVGIKDPCRPGVREAVRICTSAGVKVRMVTGDN
LQTAKAIALECGILSSDTEAVEPTIIEGKVFRELSEKEREQVAKKITVMGRSSPNDKLLLVQALRK
NGDVVAVTGDGTNDAPALHEADIGLSMGISGTEVAKESSDIIILDDNFASVVKVVRWGRSVYANIQ
KFIQFQLTVNVAALIINVVAAMSSGDVPLKAVQLLWVNLIMDTLGALALATEPPTDHLMHRTPVGR
REPLITNIMWRNLLVQSFYQVAVLLVLNFAGLSILGLNHENHAHAVEVKNTMIFNAFVMCQIFNEF
NARKPDEMNVFRGVNKNPLFVAIVGVTFILQIIIVTFLGKFAHTVRLGWQLWLASIIIGLVSWPLA
IVGKLIPVPKTPMSVYFKKPFRKYKASRNA
>AtACA10
MSGQFNNSPRGEDKDVEAGTSSFTEYEDSPFDIASTKNAPVERLRRWRQAALVLNASRRFRYTLDL
KREEDKKQMLRKMRAHAQAIRAAHLFKAAASRVTGIASPLPTPGGGDFGIGQEQIVSISRDQNIGA
LQELGGVRGLSDLLKTNLEKGIHGDDDDILKRKSAFGSNTYPQKKGRSFWRFVWEASQDLTLIILI
VAAVASLALGIKTEGIEKGWYDGISIAFAVLLVIVVTATSDYRQSLQFQNLNEEKRNIRLEVTRDG
RRVEISIYDIVVGDVIPLNIGDQVPADGVLVAGHSLAVDESSMTGESKIVQKNSTKHPFLMSGCKV
ADGNGTMLVTGVGVNTEWGLLMASVSEDNGGETPLQVRLNGVATFIGIVGLTVAGVVLFVLVVRYF
TGHTKNEQGGPQFIGGKTKFEHVLDDLVEIFTVAVTIVVVAVPEGLPLAVTLTLAYSMRKMMADKA
LVRRLSACETMGSATTICSDKTGTLTLNEMTVVECYAGLQKMDSPDSSSKLPSAFTSILVEGIAHN
TTGSVFRSESGEIQVSGSPTERAILNWAIKLGMDFDALKSESSAVQFFPFNSEKKRGGVAVKSPDS
SVHIHWKGAAEIVLGSCTHYMDESESFVDMSEDKMGGLKDAIDDMAARSLRCVAIAFRTFEADKIP
TDEEQLSRWELPEDDLILLAIVGIKDPCRPGVKNSVLLCQQAGVKVRMVTGDNIQTAKAIALECGI
LASDSDASEPNLIEGKVFRSYSEEERDRICEEISVMGRSSPNDKLLLVQSLKRRGHVVAVTGDGTN
DAPALHEADIGLAMGIQGTEVAKEKSDIIILDDNFESVVKVVRWGRSVYANIQKFIQFQLTVNVAA
LVINVVAAISAGEVPLTAVQLLWVNLIMDTLGALALATEPPTDHLMDRAPVGRREPLITNIMWRNL
FIQAMYQVTVLLILNFRGISILHLKSKPNAERVKNTVIFNAFVICQVFNEFNARKPDEINIFRGVL
RNHLFVGIISITIVLQVVIVEFLGTFASTTKLDWEMWLVCIGIGSISWPLAVIGKLIPVPETPVSQ
YFRINRWRRNSSG
>AtACA11
MSNLLKDFEVASKNPSLEARQRWRSSVGLVKNRARRFRMISNLDKLAENEKKRCQIQEKIRVVFYV
QKAAFQFIDAGARPEYKLTDEVKKAGFYVEADELASMVRNHDTKSLTKIGGPEGIAQKVSVSLAEG
VRSSELHIREKIYGENRYTEKPARSFLTFVWEALQDITLIILMVCAVVSIGVGVATEGFPKGMYDG
TGILLSIILVVMVTAISDYKQSLQFRDLDREKKKIIIQVTRDGSRQEVSIHDLVVGDVVHLSIGDQ
VPADGIFISGYNLEIDESSLSGESEPSHVNKEKPFLLSGTKVQNGSAKMLVTTVGMRTEWGKLMDT
LSEGGEDETPLQVKLNGVATIIGKIGLGFAVLTFVVLCIRFVVEKATAGSITEWSSEDALTLLDYF
AIAVTIIVVAVPEGLPLAVTLSLAFAMKQLMSDRALVRHLAACETMGSSTCICTDKTGTLTTNHMV
VNKVWICENIKERQEENFQLNLSEQVKNILIQAIFQNTGSEVVKDKEGKTQILGSPTERAILEFGL
LLGGDVDTQRREHKILKIEPFNSDKKKMSVLTSHSGGKVRAFCKGASEIVLKMCEKVVDSNGESVP
LSEEKIASISDVIEGFASEALRTLCLVYTDLDEAPRGDLPNGGYTLVAVVGIKDPVRPGVREAVQT

CQAAGITVRMVTGDNISTAKAIAKECGILTAGGVAIEGSDFRNLPPHEMRAILPKIQVMARSLPLD
KHTLVNNLRKMGEVVAVTGDGTNDAPALHEADIGLAMGIAGTEVAKENADVIIMDDNFATIVNVAK
WGRAVYINIQKFVQFQLTVNVVALIINFVSACITGSAPLTAVQLLWVNMIMDTLGALALATEPPNE
GLMKRQPIGRTASFITRAMWRNIIGQSIYQLIVLGILNFAGKQILNLNGPDSTIVLNTIIFNSFVF
CQVFNEVNSREIEKINVFEGMFKSWVFVAVMTATVGFQVIIVEFLGAFASTVPLSWQHWLLCILIG
SVSMILAVGLKCIPVESNRHHDGYELLPSGPSDSA
>AtACA12
MRDLKEYDYSALLLNLTTSSLNKAQRRWRFAYAAIYSMRAMLSLVKEIVPARIDPKTSDASLSLSY
TALESGEGAKINSMPLSYVPAIDQEQLVEIMKGKDLPGIQALGGVEGVAASLRTNPTKGIHGNEQE
VSRRRDLFGSNTYHKPPPKGLLFFVYEAFKDLTILILLVCAIFSLGFGIKEHGIKEGWYEGGSIFV
AVFLVIVVSALSNFRQERQFDKLSKISNNIKVEVLRDSRRQHISIFDVVVGDVVFLKIGDQIPADG
LFLEGHSLQVDESSMTGESDHLEVDHKDNPFLFSGTKIVDGFAQMLVVSVGMSTTWGQTMSSINQD
SSERTPLQVRLDTLTSTIGKIGLTVAALVLVVLLVRYFTGNTEKEGKREYNGSKTPVDTVVNSVVR
IVAAAVTIVVVAIPEGLPLAVTLTLAYSMKRMMSDQAMVRKLSACETMGSATVICTDKTGTLTLNE
MKVTKFWLGQESIHEDSTKMISPDVLDLLYQGTGLNTTGSVCVSDSGSTPEFSGSPTEKALLSWTV
LNLGMDMESVKQKHEVLRVETFSSAKKRSGVLVRRKSDNTVHVHWKGAAEMVLAMCSHYYTSTGSV
DLMDSTAKSRIQAIIQGMAASSLRCIAFAHKIASNDSVLEEDGLTLMGIVGLKDPCRPGVSKAVET
CKLAGVTIKMITGDNVFTAKAIAFECGILDHNDKDEEDAVVEGVQFRNYTDEERMQKVDKIRVMAR
SSPSDKLLMVKCLRLKGHVVAVTGDGTNDAPALKEADIGLSMGIQGTEVAKESSDIVILDDNFASV
ATVLKWGRCVYNNIQKFIQFQLTVNVAALVINFIAAISAGEVPLTAVQLLWVNLIMDTLGALALAT
ERPTNELLKRKPVGRTEALITNVMWRNLLVQSLYQIAVLLILQFKGMSIFSVRKEVKDTLIFNTFV
LCQVFNEFNAREMEKKNVFKGLHRNRLFIGIIAITIVLQVIMVEFLKKFADTVRLNGWQWGTCIAL
ASLSWPIGFFTKFIPVSETPFLSYFKNPRSLFKGSRSPSLKKP
>AtACA13
MRRNVSDHAEKKDKVGVEVLLELPKTLSKSNKKWQLALIKLYCSRTLLNCAKHAIRKPGLFPRSLS
YTAIDLDHHHGDDHFKIDTETLNDLVKNKNQEKLESLGGPNGLVSALKSNTRLGINEEGDEIQRRR
STFGSNTYTRQPSKGLFHFVVEAFKDLTILILLGCATLSLGFGIKEHGLKEGWYDGGSIFVAVFLV
VAVSAVSNFRQNRQFDKLSKVSSNIKIDVVRNGRRQEISIFDIVVGDIVCLNIGDQVPADGVFVEG
HLLHVDESSMTGESDHVEVSLTGNTFLFSGTKIADGFGKMAVTSVGMNTAWGQMMSHISRDTNEQT
PLQSRLDKLTSSIGKVGLLVAFLVLLVLLIRYFTGTTKDESGNREYNGKTTKSDEIVNAVVKMVAA
AVTIIVVAIPEGLPLAVTLTLAYSMKRMMKDNAMVRKLSACETMGSATVICTDKTGTLTLNQMKVT
DFWFGLESGKASSVSQRVVELFHQGVAMNTTGSVFKAKAGTEYEFSGSPTEKAILSWAVEELEMGM
EKVIEEHDVVHVEGFNSEKKRSGVLMKKKGVNTENNVVHWKGAAEKILAMCSTFCDGSGVVREMKE
DDKIQFEKIIQSMAAKSLRCIAFAYSEDNEDNKKLKEEKLSLLGIIGIKDPCRPGVKKAVEDCQFA
GVNIKMITGDNIFTARAIAVECGILTPEDEMNSEAVLEGEKFRNYTQEERLEKVERIKVMARSSPF
DKLLMVKCLKELGHVVAVTGDGTNDAPALKEADIGLSMGIQGTEVAKESSDIVILDDNFASVATVL
KWGRCVYNNIQKFIQFQLTVNVAALVINFVAAVSAGDVPLTAVQLLWVNLIMDTLGALALATEKPT
NDLMKKKPIGRVAPLITNIMWRNLLAQAFYQISVLLVLQFRGRSIFNVTEKVKNTLIFNTFVLCQV
FNEFNARSLEKKNVFKGLHKNRLFIGIIVVTVVLQVVMVEFLKRFADTERLNLGQWGVCIAIAAAS
WPIGWLVKSVPVPERHFFSYLKWKKRS
>AtAHA1
MSGLEDIKNETVDLEKIPIEEVFQQLKCTREGLTTQEGEDRIVIFGPNKLEEKKESKILKFLGFMW
NPLSWVMEAAALMAIALANGDNRPPDWQDFVGIICLLVINSTISFIEENNAGNAAAALMAGLAPKT
KVLRDGKWSEQEAAILVPGDIVSIKLGDIIPADARLLEGDPLKVDQSALTGESLPVTKHPGQEVFS
GSTCKQGEIEAVVIATGVHTFFGKAAHLVDSTNQVGHFQKVLTSIGNFCICSIAIGIAIEIVVMYP
IQHRKYRDGIDNLLVLLIGGIPIAMPTVLSVTMAIGSHRLSQQGAITKRMTAIEEMAGMDVLCSDK
TGTLTLNKLSVDKNLVEVFCKGVEKDQVLLFAAMASRVENQDAIDAAMVGMLADPKEARAGIREVH
FLPFNPVDKRTALTYIDSDGNWHRVSKGAPEQILDLANARPDLRKKVLSCIDKYAERGLRSLAVAR
QVVPEKTKESPGGPWEFVGLLPLFDPPRHDSAETIRRALNLGVNVKMITGDQLAIGKETGRRLGMG

TNMYPSAALLGTDKDSNIASIPVEELIEKADGFAGVFPEHKYEIVKKLQERKHIVGMTGDGVNDAP
ALKKADIGIAVADATDAARGASDIVLTEPGLSVIISAVLTSRAIFQRMKNYTIYAVSITIRIVFGF
MLIALIWEFDFSAFMVLIIAILNDGTIMTISKDRVKPSPTPDSWKLKEIFATGIVLGGYQAIMSVI
FFWAAHKTDFFSDKFGVRSIRDNNDELMGAVYLQVSIISQALIFVTRSRSWSFVERPGALLMIAFV
IAQLVATLIAVYADWTFAKVKGIGWGWAGVIWIYSIVTYFPQDILKFAIRYILSGKAWASLFDNRT
AFTTKKDYGIGEREAQWAQAQRTLHGLQPKEDVNIFPEKGSYRELSEIAEQAKRRAEIARLRELHT
LKGHVESVAKLKGLDIDTAGHHYTV
>AtAHA2
MSSLEDIKNETVDLEKIPIEEVFQQLKCSREGLTTQEGEDRIQIFGPNKLEEKKESKLLKFLGFMW
NPLSWVMEMAAIMAIALANGDGRPPDWQDFVGIICLLVINSTISFIEENNAGNAAAALMAGLAPKT
KVLRDGKWSEQEAAILVPGDIVSIKLGDIIPADARLLEGDPLKVDQSALTGESLPVTKHPGQEVFS
GSTCKQGEIEAVVIATGVHTFFGKAAHLVDSTNQVGHFQKVLTAIGNFCICSIAIGMVIEIIVMYP
IQRRKYRDGIDNLLVLLIGGIPIAMPTVLSVTMAIGSHRLSQQGAITKRMTAIEEMAGMDVLCSDK
TGTLTLNKLSVDKNLVEVFCKGVEKDQVLLFAAMASRVENQDAIDAAMVGMLADPKEARAGIREVH
FLPFNPVDKRTALTYIDGSGNWHRVSKGAPEQILELAKASNDLSKKVLSIIDKYAERGLRSLAVAR
QVVPEKTKESPGAPWEFVGLLPLFDPPRHDSAETIRRALNLGVNVKMITGDQLAIGKETGRRLGMG
TNMYPSSALLGTHKDANLASIPVEELIEKADGFAGVFPEHKYEIVKKLQERKHIVGMTGDGVNDAP
ALKKADIGIAVADATDAARGASDIVLTEPGLSVIISAVLTSRAIFQRMKNYTIYAVSITIRIVFGF
MLIALIWEFDFSAFMVLIIAILNDGTIMTISKDRVKPSPTPDSWKLKEIFATGVVLGGYQAIMTVI
FFWAAHKTDFFSDTFGVRSIRDNNHELMGAVYLQVSIISQALIFVTRSRSWSFVERPGALLMIAFL
IAQLIATLIAVYANWEFAKIRGIGWGWAGVIWLYSIVTYFPLDVFKFAIRYILSGKAWLNLFENKT
AFTMKKDYGKEEREAQWALAQRTLHGLQPKEAVNIFPEKGSYRELSEIAEQAKRRAEIARLRELHT
LKGHVESVVKLKGLDIETPSHYTV
>AtAHA3
MASGLEDIVNENVDLEKIPIEEVFQQLKCSREGLSGAEGENRLQIFGPNKLEEKKESKLLKFLGFM
WNPLSWVMEAAAIMAIALANGGGKPPDWQDFVGIVCLLVINSTISFVEENNAGNAAAALMAGLAPK
TKVLRDGKWSEQEASILVPGDIVSIKLGDIIPADARLLEGDPLKVDQSALTGESLPATKGPGEEVF
SGSTCKQGEIEAVVIATGVHTFFGKAAHLVDSTNQVGHFQKVLTAIGNFCICSIAVGIAIEIVVMY
PIQRRHYRDGIDNLLVLLIGGIPIAMPTVLSVTMAIGSHKLSQQGAITKRMTAIEEMAGMDVLCSD
KTGTLTLNKLSVDKNLIEVYCKGVEKDEVLLFAARASRVENQDAIDAAMVGMLADPKEARAGIREI
HFLPFNPVDKRTALTFIDSNGNWHRVSKGAPEQILDLCNARADLRKRVHSTIDKYAERGLRSLAVS
RQTVPEKTKESSGSPWEFVGVLPLFDPPRHDSAETIRRALDLGVNVKMITGDQLAIAKETGRRLGM
GSNMYPSSSLLGKHKDEAMAHIPVEDLIEKADGFAGVFPEHKYEIVKKLQERKHICGMTGDGVNDA
PALKKADIGIAVADATDAARGASDIVLTEPGLSVIISAVLTSRAIFQRMKNYTIYAVSITIRIVFG
FMLIALIWKFDFSPFMVLIIAILNDGTIMTISKDRVKPSPTPDSWKLKEIFATGVVLGGYMAIMTV
VFFWAAYKTDFFPRTFHVRDLRGSEHEMMSALYLQVSIVSQALIFVTRSRSWSFTERPGYFLLIAF
WVAQLIATAIAVYGNWEFARIKGIGWGWAGVIWLYSIVFYFPLDIMKFAIRYILAGTAWKNIIDNR
TAFTTKQNYGIEEREAQWAHAQRTLHGLQNTETANVVPERGGYRELSEIANQAKRRAEIARLRELH
TLKGHVESVVKLKGLDIETAGHYTV
>AtAHA4
MTTTVEDNREVLEAVLKEAVDLENVPIEEVFENLRCSKEGLTTQAADERLALFGHNKLEEKKESKF
LKFLGFMWNPLSWVMEAAAIMAIALANGGGKPPDWQDFVGIITLLVINSTISFIEENNAGNAAAAL
MARLAPKAKVLRDGRWGEQDAAILVPGDIISIKLGDIVPADARLLEGDPLKIDQSALTGESLPVTK
SSGDGVYSGSTCKQGEIEAVVIATGVHTFFGKAAHLVDTTNQIGHFQQVLTAIGNFCICSIAVGML
IEIVVMYPIQHRAYRPGIDNLLVLLIGGIPIAMPTVLSVTMAIGSHRLSQQGAITKRMTAIEEMAG
MDVLCSDKTGTLTLNKLTVDKNLIEVFMKGVDADTVVLMAARASRLENQDAIDAAIVGMLADPKDA
RAGIQEVHFLPFNPTDKRTALTYIDNEGNTHRVSKGAPEQILNLAHNKSEIERRVHAVIDKFAERG
LRSLAVAYQDVPEGRKDSAGGPWQFVGLMPLFDPPRHDSAETIRRALNLGVSVKMITGDQLAIGKE
TGRRLGMGTNMYPSSALLGQNKDESIVALPVDELIEKADGFAGVFPEHKYEIVKRLQARKHICGMT

GDGVNDAPALKKADIGIAVADATDAARSASDIVLTEPGLSVIISAVLTSRAIFQRMKNYTIYAVSI
TIRIVLGFMLLALIWQFDFPPFMVLIIAILNDGTIMTISKDRVKPSPLPDSWKLSEIFATGVVFGS
YMAMMTVIFFWVSYKTDFFPRTFGVATLEKTAHDDFRKLASAIYLQVSIISQALIFVTRSRSWSFV
ERPGIFLMIAFILAQLVATLIAVYANWSFAAIEGIGWGWAGVIWLYNIIFYIPLDFIKFFIRYALS
GRAWDLVIEQRVAFTRQKDFGKEQRELQWAHAQRTLHGLQAPDTKMFTDRTHVSELNQMAEEAKRR
AEIARLRELHTLKGHVESVVRLKGLDIETIQQAYTV
>AtAHA5
MSELDHIKNESVDLVRIPMEEVFEELKCTKQGLTANEASHRLDVFGPNKLEEKKESKLLKFLGFMW
NPLSWVMEVAALMAIALANGGGRPPDWQDFVGIVCLLLINSTISFIEENNAGNAAAALMAGLAPKT
KVLRDNQWSEQEASILVPGDVISIKLGDIIPADARLLDGDPLKIDQSSLTGESIPVTKNPSDEVFS
GSICKQGEIEAIVIATGVHTFFGKAAHLVDNTNQIGHFQKVLTSIGNFCICSIALGIIVELLVMYP
IQRRRYRDGIDNLLVLLIGGIPIAMPSVLSVTMATGSHRLFQQGAITKRMTAIEEMAGMDVLCCDK
TGTLTLNKLTVDKNLVEVFAKGVGKEHVFLLAARASRIENQDAIDAAIVGMLADPKEARAGVREVH
FFPFNPVDKRTALTYVDSDGNWHRASKGAPEQILNLCNCKEDVRRKVHGVIDKFAERGLRSLAVAR
QEVLEKKKDAPGGPWQLVGLLPLFDPPRHDSAETIRRALNLGVNVKMITGDQLAIGKETGRRLGMG
TNMYPSSALLGQVKDSSLGALPVDELIEKADGFAGVFPEHKYEIVHRLQQRNHICGMTGDGVNDAP
ALKKADIGIAVVDATDAARGASDIVLTEPGLSVIISAVLTSRAIFQRMKNYTIYAVSITIRIVFGF
MFIALIWQFDFSPFMVLIIAILNDGTIMTISKDRMKPSPQPDSWKLRDIFSTGVVLGGYQALMTVV
FFWVMKDSDFFSNYFGVRPLSQRPEQMMAALYLQVSIISQALIFVTRSRSWSYAECPGLLLLGAFV
IAQLVATFIAVYANWSFARIEGAGWGWAGVIWLYSFLTYIPLDLLKFGIRYVLSGKAWLNLLENKT
AFTTKKDYGKEEREAQWAAAQRTLHGLQPAEKNNIFNEKNSYSELSQIAEQAKRRAEVVRLREINT
LKGHVESVVKLKGLDIDTIQQHYTV
>AtAHA6
MAADISWDEIKKENVDLEKIPVDEVFQQLKCSREGLSSEEGRNRLQIFGANKLEEKVENKFLKFLG
FMWNPLSWVMEAAAIMAIVLANGGGRPPDWQDFVGITCLLIINSTISFIEENNAGNAAAALMANLA
PKTKVLRDGRWGEQEAAILVPGDLISIKLGDIVPADARLLEGDPLKIDQSALTGESLPATKHQGDE
VFSGSTCKQGEIEAVVIATGVHTFFGKAAHLVDSTNNVGHFQKVLTAIGNFCICSIGIGMLIEIII
MYPIQHRKYRDGIDNLLVLLIGGIPIAMPTVLSVTMAIGSHRLSQQGAITKRMTAIEEMAGMDVLC
SDKTGTLTLNKLTVDKNLIEVFSKDVDKDYVILLSARASRVENQDAIDTSIVNMLGDPKEARAGIT
EVHFLPFNPVEKRTAITYIDTNGEWHRCSKGAPEQIIELCDLKGETKRRAHEIIDKFAERGLRSLG
VARQRVPEKDKESAGTPWEFVGLLPLFDPPRHDSAETIRRALDLGVNVKMITGDQLAIGKETGRRL
GMGTNMYPSSSLLENKDDTTGGVPVDELIEKADGFAGVFPEHKYEIVRKLQERKHIVGMTGDGVND
APALKKADIGIAVDDATDAARSASDIVLTEPGLSVIVSAVLTSRAIFQRMKNYTIYAVSITIRIVL
GFMLVALIWEFDFSPFMVLIIAILNDGTIMTISKDRVKPSPIPDSWKLKEIFATGVVLGTYMALVT
VVFFWLAHDTTFFSDKFGVRSLQGKDEELIAVLYLQVSIISQALIFVTRSRSWSFVERPGLLLLIA
FFVAQLIATLIATYAHWEFARIKGCGWGWCGVIWIYSIVTYIPLDILKFITRYTLSGKAWNNMIEN
RTAFTTKKDYGRGEREAQWALAQRTLHGLKPPESMFEDTATYTELSEIAEQAKKRAEVARLREVHT
LKGHVESVVKLKGLDIDNLNQHYTV
>AtAHA7
MTDIEALKAITTESIDLENVPVEEVFQHLKCTKEGLTSNEVQERLTLFGYNKLEEKKESKILKFLG
FMWNPLSWVMEAAALMAIGLAHGGGKPADYHDFVGIVVLLLINSTISFVEENNAGNAAAALMAQLA
PKAKAVRDGKWNEIDAAELVPGDIVSIKLGDIIPADARLLEGDPLKIDQATLTGESLPVTKNPGAS
VYSGSTCKQGEIEAVVIATGVHTFFGKAAHLVDSTTHVGHFQKVLTAIGNFCICSIAVGMAIEIVV
IYGLQKRGYRVGIDNLLVLLIGGIPIAMPTVLSVTMAIGAHRLAQQGAITKRMTAIEEMAGMDVLC
SDKTGTLTLNKLSVDKNLIEVFKRGIDRDMAVLMAARAARLENQDAIDTAIVSMLSDPKEARAGIK
ELHFLPFSPANRRTALTYLDGEGKMHRVSKGAPEEILDMAHNKLEIKEKVHATIDKFAERGLRSLG
LAYQEVPDGDVKGEGGPWDFVALLPLFDPPRHDSAQTIERALHLGVSVKMITGDQLAIAKETGRRL
GMGTNMYPSSSLLSDNNTEGVSVDELIENADGFAGVFPEHKYEIVKRLQSRKHICGMTGDGVNDAP
ALKKADIGIAVDDATDAARGASDIVLTEPGLSVIISAVLTSRAIFQRMKNYTIYAVSITIRIVMGF

MLLCVFWEFDFPPFMVLVIAILNDGTIMTISKDRVKPSPTPDCWKLKEIFATGVVLGAYLAIMTVV
FFWAAYETNFFHNIFHVRNFNQHHFKMKDKKVAAHLNEQMASAVYLQVSTISQALIFVTRSRSWSF
VERPGFLLVIAFLIAQLVASVISAMANWPFAGIRSIGWGWTGVIWIFNIVTYMLLDPIKFLVRYAL
SGKSWDRMVEGRTALTGKKNFGQEERMAAWATEKRTQHGLETGQKPVYERNSATELNNMAEEAKRR
AEIARMRELQTLKGKVESAAKLKGYDLEDPNSNNYTI
>AtAHA8
MATEFSWDEIKKENVDLERIPVEEVFEQLKCSKEGLSSDEGAKRLEIFGANKLEEKSENKFLKFLG
FMWNPLSWVMESAAIMAIVLANGGGKAPDWQDFIGIMVLLIINSTISFIEENNAGNAAAALMANLA
PKTKVLRDGKWGEQEASILVPGDLISIKLGDIVPADARLLEGDPLKIDQSALTGESLPTTKHPGDE
VFSGSTCKQGEIEAVVIATGVHTFFGKAAHLVDSTNNVGHFQKVLTSIGNFCICSIGLGMLIEILI
MYPIQHRTYRDGIDNLLVLLIGGIPIAMPTVLSVTMAIGSHRLSQQGAITKRMTAIEEMAGMDVLC
SDKTGTLTLNKLSVDKSLIEVFPKNMDSDSVVLMAARASRIENQDAIDASIVGMLGDPKEARAGIT
EVHFLPFNPVDKRTAITYIDESGDWHRSSKGAPEQIIELCNLQGETKRKAHEVIDGFAERGLRSLG
VAQQTVPEKTKESDGSPWEFVGLLPLFDPPRHDSAETIRRALELGVNVKMITGDQLAIGIETGRRL
GMGTNMYPSTSLLGNSKDESLVGIPIDELIEKADGFAGVFPEHKYEIVKKLQERKHICGMTGDGVN
DAPALKKADIGIAVADATDAARSASDIVLTEPGLSVIISAVLTSRAIFQRMKNYTIYAVSITIRIV
LGFMLVALIWRFDFAPFMVLIIAILNDGTIMTISKDRVKPSPVPDSWKLNEIFATGVVLGTYMALT
TVLFFWLAHDTDFFSKTFGVRSIQGNEEELMAALYLQVSIISQALIFVTRSRSWSFVERPGFLLLI
AFVIAQLVATLIAVYANWGFARIVGCGWGWAGGIWVYSIITYIPLDILKFIIRYALTGKAWDNMIN
QKTAFTTKKDYGKGEREAQWALAQRTLHGLPPPEAMFNDNKNELSEIAEQAKRRAEVARLRELHTL
KGHVESVVKLKGLDIDTIQQHYTV
>AtAHA9
MAGNKDSSWDDIKNEGIDLEKIPIEEVLTQLRCTREGLTSDEGQTRLEIFGPNKLEEKKENKVLKF
LGFMWNPLSWVMELAAIMAIALANGGGRPPDWQDFVGITVLLIINSTISFIEENNAGNAAAALMAG
LAPKTKVLRDGKWSEQEAAILVPGDIISIKLGDIVPADGRLLDGDPLKIDQSALTGESLPVTKHPG
QEVYSGSTCKQGELEAVVIATGVHTFFGKAAHLVDSTNQEGHFQKVLTAIGNFCICSIAIGMLIEI
VVMYPIQKRAYRDGIDNLLVLLIGGIPIAMPTVLSVTMAIGSHRLSQQGAITKRMTAIEEMAGMDV
LCSDKTGTLTLNKLTVDKSMVEVFVKDLDKDQLLVNAARASRVENQDAIDACIVGMLGDPREAREG
ITEVHFFPFNPVDKRTAITYIDANGNWHRVSKGAPEQIIELCNLREDASKRAHDIIDKFADRGLRS
LAVGRQTVSEKDKNSPGEPWQFLGLLPLFDPPRHDSAETIRRALDLGVNVKMITGDQLAIGKETGR
RLGMGTNMYPSSALLGQDKDESIASLPVDELIEKADGFAGVFPEHKYEIVKRLQEMKHICGMTGDG
VNDAPALKRADIGIAVADATDAARSASDIVLTEPGLSVIVSAVLTSRAIFQRMKNYTIYAVSITIR
IVMGFMLLALIWKFDFSPFMVLIVAILNDGTIMTISKDRVKPSPLPDSWKLKEIFATGVVLGTYLA
VMTVVFFWAAESTDFFSAKFGVRSISGNPHELTAAVYLQVSIVSQALIFVTRSRSWSYVERPGFWL
ISAFFMAQLIATLIAVYANWNFARIRGIGWGWAGVIWLYSIVFYIPLDILKFIIRYSLSGRAWDNV
IENKTAFTSKKDYGKGEREAQWAQAQRTLHGLQPAQTSDMFNDKSTYRELSEIADQAKRRAEVARL
RERHTLKGHVESVVKQKGLDIEAIQQHYTL
>AtAHA10
MAEDLDKPLLDPDTFNRKGIDLGILPLEEVFEYLRTSPQGLLSGDAEERLKIFGPNRLEEKQENRF
VKFLGFMWNPLSWVMEAAALMAIALANSQSLGPDWEDFTGIVCLLLINATISFFEENNAGNAAAAL
MARLALKTRVLRDGQWQEQDASILVPGDIISIKLGDIIPADARLLEGDPLKIDQSVLTGESLPVTK
KKGEQVFSGSTCKQGEIEAVVIATGSTTFFGKTARLVDSTDVTGHFQQVLTSIGNFCICSIAVGMV
LEIIIMFPVQHRSYRIGINNLLVLLIGGIPIAMPTVLSVTLAIGSHRLSQQGAITKRMTAIEEMAG
MDVLCCDKTGTLTLNSLTVDKNLIEVFVDYMDKDTILLLAGRASRLENQDAIDAAIVSMLADPREA
RANIREIHFLPFNPVDKRTAITYIDSDGKWYRATKGAPEQVLNLCQQKNEIAQRVYAIIDRFAEKG
LRSLAVAYQEIPEKSNNSPGGPWRFCGLLPLFDPPRHDSGETILRALSLGVCVKMITGDQLAIAKE
TGRRLGMGTNMYPSSSLLGHNNDEHEAIPVDELIEMADGFAGVFPEHKYEIVKILQEMKHVVGMTG
DGVNDAPALKKADIGIAVADATDAARSSADIVLTDPGLSVIISAVLTSRAIFQRMRNYTVYAVSIT
IRIVLGFTLLALIWEYDFPPFMVLIIAILNDGTIMTISKDRVRPSPTPESWKLNQIFATGIVIGTY

LALVTVLFYWIIVSTTFFEKHFHVKSIANNSEQVSSAMYLQVSIISQALIFVTRSRGWSFFERPGT
LLIFAFILAQLAATLIAVYANISFAKITGIGWRWAGVIWLYSLIFYIPLDVIKFVFHYALSGEAWN
LVLDRKTAFTYKKDYGKDDGSPNVTISQRSRSAEELRGSRSRASWIAEQTRRRAEIARLLEVHSVS
RHLESVIKLKQIDQRMIRAAHTV
>AtAHA11
MGDKEEVLEAVLKETVDLENVPIEEVFESLRCSREGLTTEAADERLALFGHNKLEEKKESKFLKFL
GFMWNPLSWVMEAAAIMAIALANGGGKPPDWQDFVGIITLLVINSTISFIEENNAGNAAAALMARL
APKAKVLRDGRWGEQDAAILVPGDIISIKLGDIVPADARLLEGDPLKIDQSSLTGESLPVTKGPGD
GVYSGSTCKQGELEAVVIATGVHTFFGKAAHLVDTTNHVGHFQQVLTAIGNFCICSIAVGMIIEIV
VMYPIQHRAYRPGIDNLLVLLIGGIPIAMPTVLSVTMAIGSHRLSQQGAITKRMTAIEEMAGMDVL
CSDKTGTLTLNKLTVDKNLIEVFTKGVDADTVVLMAAQASRLENQDAIDAAIVGMLADPKEARAGV
REVHFLPFNPTDKRTALTYIDSDGKMHRVSKGAPEQILNLAHNRAEIERRVHAVIDKFAERGLRSL
AVAYQEVPEGTKESAGGPWQFMGLMPLFDPPRHDSAETIRRALNLGVNVKMITGDQLAIGKETGRR
LGMGTNMYPSSALLGQHKDESIGALPIDDLIEKADGFAGVFPEHKYEIVKRLQARKHICGMTGDGV
NDAPALKKADIGIAVADATDAARSASDIVLTEPGLSVIISAVLTSRAIFQRMKNYTIYAVSITIRI
VLGFMLLALIWKFDFPPFMVLIIAILNDGTIMTISKDRVKPSPLPDSWKLSEIFATGVVFGSYMAM
MTVIFFWAAYKTDFFPRTFGVSTLEKTAHDDFRKLASAIYLQVSIISQALIFVTRSRSWSYVERPG
MLLVVAFILAQLVATLIAVYANWSFAAIEGIGWGWAGVIWLYNIVFYIPLDIIKFLIRYALSGRAW
DLVIEQRVAFTRQKDFGKEQRELQWAHAQRTLHGLQAPDAKMFPERTHFNELSQMAEEAKRRAEIA
RLRELHTLKGHVESVVRLKGLDIETIQQAYTV
>AtALA1
MDPRKSIDKPPHHDPILGVSSRWSVSSKDNKEVTFGDLGSKRIRHGSAGADSEMLSMSQKEIKDED
ARLIYINDPDRTNERFEFTGNSIKTAKYSVFTFLPRNLFEQFHRVAYIYFLVIAVLNQLPQLAVFG
RGASIMPLAFVLLVSAIKDAYEDFRRHRSDRVENNRLALVFEDHQFREKKWKHIRVGEVIKVQSNQ
TLPCDMVLLATSDPTGVVYVQTTNLDGESNLKTRYAKQETLLKAADMESFNGFIKCEKPNRNIYGF
QANMEIDGRRLSLGPSNIILRGCELKNTAWALGVVVYAGGETKAMLNNSGAPSKRSRLETRMNLEI
ILLSLFLIVLCTIAAATAAVWLRTHRDDLDTILFYRRKDYSERPGGKNYKYYGWGWEIFFTFFMAV
IVYQIMIPISLYISMELVRIGQAYFMTNDDQMYDESSDSSFQCRALNINEDLGQIKYLFSDKTGTL
TDNKMEFQCACIEGVDYSDREPADSEHPGYSIEVDGIILKPKMRVRVDPVLLQLTKTGKATEEAKR
ANEFFLSLAACNTIVPIVSNTSDPNVKLVDYQGESPDEQALVYAAAAYGFLLIERTSGHIVINVRG
ETQRFNVLGLHEFDSDRKRMSVILGCPDMSVKLFVKGADSSMFGVMDESYGGVIHETKIQLHAYSS
DGLRTLVVGMRELNDSEFEQWHSSFEAASTALIGRAGLLRKVAGNIETNLRIVGATAIEDKLQRGV
PEAIESLRIAGIKVWVLTGDKQETAISIGFSSRLLTRNMRQIVINSNSLDSCRRSLEEANASIASN
DESDNVALIIDGTSLIYVLDNDLEDVLFQVACKCSAILCCRVAPFQKAGIVALVKNRTSDMTLAIG
DGANDVSMIQMADVGVGISGQEGRQAVMASDFAMGQFRFLVPLLLVHGHWNYQRMGYMILYNFYRN
AVFVLILFWYVLFTCYTLTTAITEWSSVLYSVIYTAIPTIIIGILDKDLGRQTLLDHPQLYGVGQR
AEGYSTTLFWYTMIDTIWQSAAIFFIPMFAYWGSTIDTSSLGDLWTIAAVVVVNLHLAMDVIRWNW
ITHAAIWGSIVAACICVIVIDVIPTLPGYWAIFQVGKTWMFWFCLLAIVVTSLLPRFAIKFLVEYY
RPSDVRIAREAEKLGTFRESQPVGVEMNLIQDPPRR
>AtALA2
MKRFVYINDDEASKELCCDNRISNRKYTLWNFLPKNLWEQFSRFMNQYFLLIACLQLWSLITPVNP
ASTWGPLIFIFAVSASKEAWDDYHRYLSDKKANEKEVWIVKQGIKKHIQAQDIQVGNIVWLRENDE
VPCDLVLLGTSDPQGVCYVETAALDGETDLKTRVIPSACVGIDLELLHKMKGVIECPVPDKDIRRF
DANMRLFPPFIDNDVCSLTIKNTLLQSCYLRNTEWACGVSVYTGNQTKLGMSRGIAEPKLTAMDAM
IDKLTGAIFVFQIVVVLVLGIAGNVWKDTEARKQWYVQYPEEAPWYELLVIPLRFELLCSIMIPIS
IKVSLDLVKGLYAKFIEWDVEMIDQETGTASYAANTAISEDLGQVEYILTDKTGTLTDNKMIFRRC
CIGGIFYGNENGDALKDAQLLNAITSGSTDVIRFLTVMAICNTVLPVQSKAGDIVYKAQSQDEDAL
VIAASKLHMVFVGKNANLLEIRFNGSVIRYEVLEILEFTSDRKRMSVVVKDCQNGKIILLSKGADE
AILPYARAGQQTRTIGDAVEHYSQLGLRTLCLAWRELEENEYLEWSVKFKEASSLLVDREWRIAEV

CQRLEHDLYILGVTAIEDRLQDGVPETIETLRKAGINFWMLTGDKQNTAIQIALSCNFISPEPKGQ
LLMIDGKTEEDVSRSLERVLLTMRITASEPKDVAFVIDGWALEIALKHHRKDFVELAILSRTAICC
RVTPSQKAQLVEILKSCDYRTLAIGDGGNDVRMIQQADIGVGISGREGLQAARAADYSIGRFRFLK
RLILVHGRYSYNRTAFLSQYSFYKSLLICFIQIFFSFISGVSGTSLFNSVSLMAYNVFYTSVPVLV
SVIDKDLSEASVMQHPQILFYCQAGRLLNPSTFAGWFGRSLFHAIIVFVITIHAYAYEKSEMEELG
MVALSGCIWLQAFVVAQETNSFTVLQHLSIWGNLVGFYAINFLFSAIPSSGMYTIMFRLCSQPSYW
ITMFLIVGAGMGPIFALKYFRYTYRPSKINILQQAERMGGPILTLGNIETQPRTIEKDLSPISITQ
PKNRSPVYEPLLSDSPNATRRSFGPGTPFEFFQSQSRLSSSSGYTRNCKDN

>AtALA3

MVRSGSFSVDSSATHQRTPSRTVTLGHIQPQAPTYRTVYCNDRESNQPVRFKGNSISTTKYNVFTF
LPKGLFEQFRRIANIYFLGISCLSMTPISPVSPITNVAPLSMVLLVSLIKEAFEDWKRFQNDMSIN
NSTVEILQDQQWVSIPWRKLQVGDIVKIKKDGFFPADILFMSSTNSDGICYVETANLDGETNLKIR
KALERTWDYLVPEKAYEFKGEIQCEQPNNSLYTFTGNLVVQKQTLPLSPDQLLLRGCSLRNTEYIV
GAVVFTGHETKVMMNAMNAPSKRSTLEKKLDKLIITIFCVLVTMCLIGAIGCSIVTDREDKYLGLH
NSDWEYRNGLMIGFFTFFTLVTLFSSIIPISLYVSIEMIKFIQSTQFINRDLNMYHAETNTPASAR
TSNLNEELGQVEYIFSDKTGTLTRNLMEFFKCSIGGVSYGCGVTEIEKGIAQRHGLKVQEEQRSTG
AIREKGFNFDDPRLMRGAWRNEPNPDLCKELFRCLAICHTVLPEGDESPEKIVYQAASPDEAALVT
AAKNFGFFFYRRTPTMVYVRESHVEKMGKIQDVAYEILNVLEFNSTRKRQSVVCRFPDGRLVLYCK
GADNVIFERLANGMDDVRKVTREHLEHFGSSGLRTLCLAYKDLNPETYDSWNEKFIQAKSALRDRE
KKLDEVAELIEKDLILIGSTAIEDKLQEGVPTCIETLSRAGIKIWVLTGDKMETAINIAYACNLIN
NEMKQFVISSETDAIREAEERGDQVEIARVIKEEVKRELKKSLEEAQHSLHTVAGPKLSLVIDGKC
LMYALDPSLRVMLLSLSLNCTSVVCCRVSPLQKAQVTSLVRKGAQKITLSIGDGANDVSMIQAAHV
GIGISGMEGMQAVMASDFAIAQFRFLTDLLLVHGRWSYLRICKVVMYFFYKNLTFTLTQFWFTFRT
GFSGQRFYDDWFQSLFNVVFTALPVIVLGLFEKDVSASLSKRYPELYREGIRNSFFKWRVVAVWAT
SAVYQSLVCYLFVTTSSFGAVNSSGKVFGLWDVSTMVFTCLVIAVNVRILLMSNSITRWHYITVGG
SILAWLVFAFVYCGIMTPHDRNENVYFVIYVLMSTFYFYFTLLLVPIVSLLGDFIFQGVERWFFPY
DYQIVQEIHRHESDASKADQLEVENELTPQEARSYAISQLPRELSKHTGFAFDSPGYESFFASQLG
IYAPQKAWDVARRASMRSRPKVPKK

>AtALA4

MARGRIRSKLRLSHIYTFGCLRPSADEGQDPHPIQGPGFSRTVYCNQPHMHKKKPLKYRSNYVSTT
RYNLITFFPKCLYEQFHRAANFYFLVAAILSVFPLSPFNKWSMIAPLVFVVGLSMLKEALEDWSRF
MQDVKINASKVYVHKSDGEFRRRKWKKISVGDIVKVEKDGFFPADLLLLSSSYEDGICYVETMNLD
GETNLKVKRSLEVTLSLDDYDSFKDFTGIIRCEDPNPSLYTFVGNLEYERQIFPLDPSQILLRDSK
LRNTPYVYGVVVFTGHDTKVMQNSTKSPSKRSRIEKTMDYIIYTLLVLLILISCISSSGFAWETKF
HMPKWWYLRPEEPENLTNPSNPVYAGFVHLITALLLYGYLIPISLYVSIEVVKVLQASFINKDLHM
YDSESGVPAHARTSNLNEELGQVDTILSDKTGTLTCNQMDFLKCSIAGTSYGVRSSEVEVAAAQQM
AVDLDEHGEVSSRTSTPRAQARDIEVESSITPRIPIKGFGFEDIRLMDGNWLREPHTDDILLFFRI
LAICHTAIPELNEETGKYTYEAESPDEASFLTAASEFGFVFFKRTQSSVYVHERLSHSGQTIEREY
KVLNLLDFTSKRKRMSVVVRDEEGQILLLCKGADSIIFERLAKNGKVYLGPTTKHLNEYGEAGLRT
LALSYRKLDEEEYSAWNAEFHKAKTSIGSDRDELLERISDMIEKDLILVGATAVEDKLQKGVPQCI
DKLAQAGLKLWVLTGDKMETAINIGYSCSLLRQGMKQICITVVNSEGASQDAKAVKDNILNQITKA
VQMVKLEKDPHAAFALIIDGKTLTYALEDEMKYQFLALAVDCASVICCRVSPKQKALVTRLVKEGT
GKITLAIGDGANDVGMIQEADIGVGISGVEGMQAVMASDFSIAQFRFLERLLVVHGHWCYKRIAQM
ICYFFYKNIAFGLTLFYFEAFTGFSGQSVYNDYYLLLFNVVLTSLPVIALGVFEQDVSSEICLQFP
ALYQQGKKNLFFDWYRILGWMGNGVYSSLVIFFLNIGIIYEQAFRVSGQTADMDAVGTTMFTCIIW
AVNVQIALTVSHFTWIQHVLIWGSIGLWYLFVALYGMMPPSLSGNIYRILVEILAPAPIYWIATFL
VTVTTVLPYFAHISFQRFLHPLDHHIIQEIKYYKRDVEDRRMWTRERTKAREKTKIGFTARVDAKI
RHLRSKLNKKQSNMSQFSTQDTMSPRSV

>AtALA5

MARGRIRSKLRLSLLYTFGCLRPATLEGQDSQPIQGPGFSRTVFCNQPHMHKKKPLRYRSNYVSTT
RYNLITFFPKSLYEQFHRAANLYFLVAAILSVFPLSPFNKWSMIAPLVFVVGLSMLKEALEDWRRF
MQDVKINARKTCVHKSDGVFRQRKWKKVSVGDIVKVEKDEFFPADLLLLSSSYEDGICYVETMNLD
GETNLKVKRSLEVSLPLDDDESFKNFMATIRCEDPNPNLYTFVGNLEFERQTFPLDPSQILLRDSK
LRNTTYVYGVVVFTGFDTKVMQNSTKSPSKRSRIERTMDYIIYTLLVLLILISCISSSGFAWETEF
HMPKMWYLRPGEPIDFTNPINPIYAGVVHLITALLLYGYLIPISLYVSIEVVKVWQASFINQDLHM
YDDESGVPANARTSNLNEELGQVHTILSDKTGTLTCNQMDFLKCSIAGTSYGVRSSEVEVAAAKQM
AVDLEEHGEISSTPQSQTKVYGTWDSSRTQEIEVEGDNNYNTPRAPIKGFGFEDNRLMNGNWLRES
QPNDILQFFRILAICHTAIPELNEETGKYTYEAESPDEASFLAAAREFGFEFFKRTQSSVFIRERF
SGSGQIIEREYKVLNLLEFTSKRKRMTVIVRDEEGQILLLCKGADSIIFERLAKNGKTYLGPTTRH
LTEYGEAGLRTLALAYRKLDEDEYAAWNSEFLKAKTSIGSDRDELLETGADMIEKELILIGATAVE
DKLQKGVPQCIDKLAQAGLKLWVLTGDKMETAINIGFACSLLRQGMRQICITSMNSEGGSQDSKRV
VKENILNQLTKAVQMVKLEKDPHAAFALIIDGKTLTYALEDDMKYQFLALAVDCASVICCRVSPKQ
KALVVRLVKEGTGKTTLAIGDGANDVGMIQEADIGVGISGVEGMQAVMASDFSIAQFRFLERLLVV
HGHWCYKRIAQMICYFFYKNIAFGLTLFYFEAFTGFSGQSVYNDYYLLLFNVVLTSLPVIALGVFE
QDVSSEICLQFPALYQQGTKNLFFDWSRILGWMCNGVYASLVIFFLNIGIIYSQAFRDNGQTADMD
AVGTTMFTCIIWAANVQIALTMSHFTWIQHVLIWGSIGMWYLFVAIYSMMPPSYSGNIYRILDEIL
APAPIYWMATLLVTVAAVLPYVAHIAFQRFLNPLDHHIIQEIKYYGRDIEDARLWTRERTKAREKT
KIGFTARVDAKIRHLRSKLNKKQSNLSHFSAQDAMSPRSL
>AtALA6
MARRRIRSRIRKSHFYTFRCLRPKTLDDQGPHVINGPGYTRIVHCNQPHLHLATKLIRYRSNYVST
TRYNLLTFLPKCLYEQFHRVANFYFLVAAILSVFPLSPFNKWSMIAPLVFVVGLSMGKEALEDWRR
FMQDVEVNSRKASVHKGSGDFGRRTWKRIRVGDIVRVEKDEFFPADLLLLSSSYEDGICYVETMNL
DGETNLKVKRCLDATLALEKDESFQNFSGTIKCEDPNPNLYTFVGNLECDGQVYPLDPNQILLRDS
KLRNTAYVYGVVVFTGHDTKVMQNSTKSPSKRSRIEKRMDYIIYTLFALLLTVSFISSLGFAVMTK
LLMAEWWYLRPDKPESLTNPTNPLYAWVVHLITALLLYGYLIPISLYVSIEVVKVLQAHFINQDLQ
LYDSESGTPAQARTSNLNEELGQVDTILSDKTGTLTCNQMDFLKCSIAGTSYGVRASEVELAAAKQ
MAMDLEEKGEEVANLSMNKGRTQRYAKLASKTSSDFELETVVTASDEKDQKQNTGVKGFSFEDNRL
MNENWLNEPNSDDILMFFRILAVCHTAIPEVDEDTGMCTYEAESPDEVAFLVASREFGFEFTKRTQ
SSVFIAERFSSSGQPVDREYKILNLLDFTSKRKRMSAIVRDEEGQILLLCKGADSIIFERLSKSGK
EYLGATSKHLNVYGEAGLRTLALGYRKLDETEYAAWNSEFHKAKTSVGADRDEMLEKVSDMMEKEL
ILVGATAVEDKLQKGVPQCIDNLAQAGLKIWVLTGDKMETAINIGYACSLLRQGMKQISISLTNVE
ESSQNSEAAAKESILMQITNASQMIKIEKDPHAAFALIIDGKTLTYALKDDVKYQFLALAVDCASV
ICCRVSPKQKALVTRLAKEGTGKTTLAIGDGANDVGMIQEADIGVGISGVEGMQAVMASDFSIAQF
RFLERLLVVHGHWCYKRIAQMICYFFYKNITFGLTLFYFECFTGFSGQSIYNDSYLLLFNVVLTSL
PVISLGVFEQDVPSDVCLQFPALYQQGPKNLFFDWYRILGWMGNGVYASIVIFTLNLGIFHVQSFR
SDGQTADMNAMGTAMFTCIIWAVNVQIALTMSHFTWIQHVMIWGSIGAWYVFLALYGMLPVKLSGN
IFHMLVEILAPAPIFWLTSLLVIAATTLPYLFHISYQRSVNPLDHHIIQEIKHFRIDVEDERMWKR
EKSKAREKTKIGFTARVDAKIRQLRGRLQRKHSVLSVMSGTSSNDTPSSNSQ
>AtALA7
MGRRRIRSRIRKSHFYTFKCLRPKTLEDQGPHIINGPGYTRIVHCNQPHLHLAKVLRYTSNYVSTT
RYNLITFLPKCLYEQFHRVANFYFLVAAILSVFPLSPFNKWSMIAPLIFVVGLSMGKEALEDWRRF
MQDVKVNSRKATVHRGDGDFGRRKWKKLRVGDVVKVEKDQFFPADLLLLSSSYEDGICYVETMNLD
GETNLKVKRCLDVTLPLERDDTFQSFSGTIKCEDPNPNLYTFVGNLEYDGQVYPLDPSQILLRDSK
LRNTSYVYGVVVFTGHDTKVMQNSTKSPSKRSRIEKRMDYIIYTLFALLVLVSFISSLGFAVMTKM
HMGDWWYLRPDKPERLTNPRNPFHAWVVHLITAVLLYGYLIPISLYVSIELVKVLQATFINQDLQM
YDSESGTPAQARTSNLNEELGQVDTILSDKTGTLTCNQMDFLKCSIAGTSYGVRASEVELAAAKQM
AIDLDEEQGEEVTHLPRTRGRMHGYAKMPSKTSSDIELETVITATDEGDQTQSTGIKGFSFEDQRL
MGGNWLNEPNSDDILMFLRILAVCHTAIPEVDEDTGKCTYEAESPDEVAFLVAAGEFGFEFTKRTQ

SSVFISERHSGQPVEREYKVLNVLDFTSKRKRMSVIVRDEKGQILLLCKGADSIIFERLSKNGKNY
LEATSKHLNGYGEAGLRTLALSYRKLDETEYSIWNSEFHKAKTSVGADRDEMLEKVSDMMEKELIL
VGATAVEDKLQKGVPQCIDKLAQAGLKIWVLTGDKMETAINIGYACSLLRQGMKQIYIALRNEEGS
SQDPEAAARENILMQIINASQMIKLEKDPHAAFALIIDGKTLTYALEDDIKYQFLALAVDCASVIC
CRVSPKQKALVTRLAKEGTGKTTLAIGDGANDVGMIQEADIGVGISGVEGMQAVMASDFSIAQFRF
LERLLVVHGHWCYKRIAQMICYFFYKNITFGLTLFYFEAFTGFSGQAIYNDSYLLLFNVILTSLPV
IALGVFEQDVSSEVCLQFPALYQQGPKNLFFDWYRIIGWMANGVYASVVIFSLNIGIFHVQSFCSG
GQTADMDAMGTAMFTCIIWAVNVQIALTMSHFTWIQHVLIWGSIVTWYIFLALFGMLPPKVSGNIF
HMLSETLAPAPIFWLTSLLVIAATTLPYLAYISFQRSLNPLDHHIIQEIKHFRIDVQDECMWTRER
SKAREKTKIGVTARVDAKIRQLRGRLQRKHSILSVMSGLSGVSASTDTTSTTQHS
>AtALA8
MAGERRKGMKFSKLYSFKCFKPFSREDHSQIGSRGYSRVVFCNDPDNPEALQLNYRGNYVSTTKYT
AANFIPKSLFEQFRRVANIYFLVVAFVSFSPLAPYTAPSVLAPLLIVIGATMVKEGVEDLRRRKQD
VEANNRKVEVLGKTGTFVETKWKNLRVGDLVKVHKDEYFPADLLLLSSSYEDGICYVETMNLDGET
NLKLKHALEITSDEESIKNFRGMIKCEDPNEHLYSFVGTLYFEGKQYPLSPQQILLRDSKLKNTDY
VYGVVVFTGHDTKVMQNATDPPSKRSKIEKKMDQIIYILFSILIVIAFTGSVFFGIATRRDMSDNG
KLRRWYLRPDHTTVFYDPRRAVAAAFFHFLTALMLYGYLIPISLYVSIEVVKVLQSIFINQDQEMY
HEETDRPARARTSNLNEELGQVDTILSDKTGTLTCNSMEFVKCSIAGTAYGRGMTEVEVALRKQKG
LMTQEEVGDNESLSIKEQKAVKGFNFWDERIVDGQWINQPNAELIQKFFRVLAICHTAIPDVNSDT
GEITYEAESPDEAAFVIASRELGFEFFSRSQTSISLHEIDHMTGEKVDRVYELLHVLEFSSSRKRM
SVIVRNPENRLLLLSKGADSVMFKRLAKHGRQNERETKEHIKKYAEAGLRTLVITYREIDEDEYIV
WEEEFLNAKTLVTEDRDALIDAAADKIEKDLILLGSTAVEDKLQKGVPDCIEKLSQAGVKIWVLTG
DKTETAINIGYACSLLREGMKQILVTLDSSDIEALEKQGDKEAVAKASFQSIKKQLREGMSQTAAV
TDNSAKENSEMFGLVIDGKSLTYALDSKLEKEFLELAIRCNSVICCRSSPKQKALVTRLVKNGTGR
TTLAIGDGANDVGMLQEADIGVGISGAEGMQAVMASDFAIAQFRFLERLLLVHGHWCYRRITLMIC
YFFYKNLAFGFTLFWYEAYASFSGKPAYNDWYMSCYNVFFTSLPVIALGVFDQDVSARLCLKYPLL
YQEGVQNVLFSWERILGWMLNGVISSMIIFFLTINTMATQAFRKDGQVVDYSVLGVTMYSSVVWTV
NCQMAISINYFTWIQHCFIWGSIGVWYLFLVIYGSLPPTFSTTAFQVFVETSAPSPIYWLVLFLVV
FSALLPYFTYRAFQIKFRPMYHDIIVEQRRTERTETAPNAVLGELPVQVEFTLHHLRANLSRRDSW
N
>AtALA9
MVGGGTKRRRRRLQLSKLYTLTCAQACFKQDHSQIGGPGFSRVVYCNEPDSPEADSRNYSDNYVRT
TKYTLATFLPKSLFEQFRRVANFYFLVTGVLAFTPLAPYTASSAIVPLLFVIGATMVKEGVEDWRR
QKQDNEVNNRKVKVHRGDGSFDAKEWKTLSIGDIVKVEKNEFFPADLVLLSSSYEDAICYVETMNL
DGETNLKVKQGLEVTSSLRDEFNFKGFEAFVKCEDPNANLYSFVGTMELKGAKYPLSPQQLLLRDS
KLRNTDFIFGAVIFTGHDTKVIQNSTDPPSKRSMIEKKMDKIIYLMFFMVITMAFIGSVIFGVTTR
DDLKDGVMKRWYLRPDSSSIFFDPKRAPVAAIYHFLTAVMLYSYFIPISLYVSIEIVKVLQSIFIN
QDIHMYYEEADKPARARTSNLNEELGQVDTILSDKTGTLTCNSMEFIKCSVAGTAYGRGVTEVEMA
MGRRKGGPLVFQSDENDIDMEYSKEAITEESTVKGFNFRDERIMNGNWVTETHADVIQKFFRLLAV
CHTVIPEVDEDTEKISYEAESPDEAAFVIAARELGFEFFNRTQTTISVRELDLVSGKRVERLYKVL
NVLEFNSTRKRMSVIVQEEDGKLLLLCKGADNVMFERLSKNGREFEEETRDHVNEYADAGLRTLIL
AYRELDEKEYKVFNERISEAKSSVSADRESLIEEVTEKIEKDLILLGATAVEDKLQNGVPDCIDKL
AQAGIKIWVLTGDKMETAINIGFACSLLRQDMKQIIINLETPEIQSLEKTGEKDVIAKASKENVLS
QIINGKTQLKYSGGNAFALIIDGKSLAYALDDDIKHIFLELAVSCASVICCRSSPKQKALVTRLVK
SGNGKTTLAIGDGANDVGMLQEADIGVGISGVEGMQAVMSSDIAIAQFRYLERLLLVHGHWCYRRI
STMICYFFYKNITFGFTLFLYETYTTFSSTPAYNDWFLSLYNVFFSSLPVIALGVFDQDVSARYCL
KFPLLYQEGVQNVLFSWRRILGWMFNGFYSAVIIFFLCKSSLQSQAFNHDGKTPGREILGGTMYTC
IVWVVNLQMALAISYFTLIQHIVIWSSIVVWYFFITVYGELPSRISTGAYKVFVEALAPSLSYWLI

TLFVVVATLMPYFIYSALQMSFFPMYHGMIQWLRYEGQCNDPEYCDIVRQRSIRPTTVGFTARLEA
KKRSVRISEPAS
>AtALA10
MAGPSRRRRRLHLSKIYSYTCGKSSFQEDHSNIGGPGFSRVVYCNEPGSPAAERRNYAGNYVRSTK
YTVASFFPKSLFEQFRRVANFYFLVTGILSLTDLSPYGAVSALLPLALVISATMVKEGIEDWRRKQ
QDIEVNNRKVKVHDGNGIFRQEEWRNLRVGDIVRVEKDEFFPADLLLLSSSYEDSVCYVETMNLDG
ETNLKVKQGLEATSSLLNQDSDFKDFRGVVRCEDPNVNLYVFVGTLALEEERFPLSIQQILLRDSK
LRNTEYVYGAVVFTGHDTKVIQNSTDPPSKRSRIERTMDKIIYLMFGLVFLMSFVGSIIFGVETRE
DKVKNGRTERWYLKPDDADIFFDPERAPMAAIYHFFTATMLYSYFIPISLYVSIEIVKVLQSIFIN
RDIHMYYEETDKPAQARTSNLNEELGMVDTILSDKTGTLTCNSMEFIKCSIAGKAYGRGITEVERA
MAVRSGGSPLVNEDLDVVVDQSGPKVKGFNFEDERVMNGNWVRQPEAAVLQKFFRLLAVCHTAIPE
TDEESGNVSYEAESPDEAAFVVAAREFGFEFFNRTQNGISFRELDLVSGEKVERVYRLLNVLEFNS
TRKRMSVIVRDDDGKLLLLSKGADNVMFERLAKNGRQFEAKTQEHVNQYADAGLRTLVLAYREVDE
NEYIEFNKSFNEAKASVSEDREALIDEITDKMERDLILLGATAVEDKLQNGVPECIDKLAQAGIKI
WVLTGDKMETAINIGFASSLLRQEMKQIIINLETPQIKSLEKSGGKDEIELASRESVVMQLQEGKA
LLAASGASSEAFALIIDGKSLTYALEDEIKKMFLDLATSCASVICCRSSPKQKALVTRLVKSGTGK
TTLAIGDGANDVGMLQEADIGVGISGVEGMQAVMSSDIAIAQFRYLERLLLVHGHWCYSRIASMIC
YFFYKNITFGVTVFLYEAYTSFSGQPAYNDWFLSLFNVFFSSLPVIALGVFDQDVSARFCYKFPLL
YQEGVQNILFSWKRIIGWMFNGFISALAIFFLCKESLKHQLFDPDGKTAGREILGGTMYTCVVWVV
NLQMALSISYFTWVQHIVIWGSIAFWYIFLMIYGAMTPSFSTDAYMVFLEALAPAPSYWLTTLFVM
IFALIPYFVYKSVQMRFFPKYHQMIQWIRYEGHSNDPEFVEMVRQRSIRPTTVGYTARRAASVRRS
ARFHDQIYKDLVGV
>AtALA11
MTKCRRRRLHLSNIYAFKGRKSNFQEDHSHIGGPGFSRVVYCNEPNSPAAERRNYVGNYVRSTKYT
LASFIPKSLFEQFRRVANFYFLVTGVLSLTALSPYSPISALLPLTFVIAASMVKEAIEDWGRKKQD
IEMNNRKVKVHDGNGIFRREGWRDLKVGNIVRVEKDEFFPADLLLLSSSYEDSICYVETMNLDGET
NLKVKQGLEATSSALHEDSDFKELKAVVKCEDPNADLYTFVGTLHFEEQRLPLSITQLLLRDSKLR
NTEYIYGVVVFTGHDTKVIQNSTDPPSKRSRIERKMDKIIYLMFGVVFLMSFIGSIVFGIETREDR
VRNGGRTERWYLRPDNADIFFDPDRAPMAAVYHFFTAVMLYSYFIPISLYVSIEIVKVLQSLFINN
DILMYYEENDKPAHARTSNLNEELGMVDTILSDKTGTLTCNSMEFIKCSIAGTAYGRGITEVERSM
AMRSNGSSLVGDDLDVVVDQSGPKIKGFNFLDERVMKGNWVKQRDAAVLQKFFRLLAVCHTAIPET
DEATGSVSYEAESPDEAAFVVAAREFGFEFFSRTQNGISFRELDLASGKTVERVYRLLNVLEFNSA
RKRMSVIVRDEDGRLLLLSKGADNVMFERLAKNGRKFEEKTREHVNEYADAGLRTLILAYREVDEN
EYIEFSKNFNEAKNSVTADRESLIDEITEQMERDLILLGATAVEDKLQNGVPDCIDKLAQAGIKIW
VLTGDKMETAINIGFACSLLRQEMKQIIINLETPHIKALEKAGEKDAIEHASRESVVNQMEEGKAL
LTASSSASSHEAFALIIDGKSLTYALEDDFKKKFLDLATGCASVICCRSSPKQKALVTRLVKSGTG
KTTLAIGDGANDVGMLQEADIGVGISGVEGMQAVMSSDIAIAQFRYLERLLLVHGHWCYSRISSMI
CYFFYKNITFGVTVFLYEAYTSFSAQPAYNDWFLSLFNVFFSSLPVIALGVFDQDVSARYCYKFPL
LYQEGVQNLLFSWKRIIGWMFNGVFTALAIFFLCKESLKHQLYNPNGKTAGREILGGTMYTCVVWV
VNLQMALAISYFTWLQHIVIWGSVAFWYIFLMIYGAITPSFSTDAYKVFIEALAPAPSYWLTTLFV
MFFALIPFFVFKSVQMRFFPGYHQMIQWIRYEGHSNDPEFVEMVRQRSIRPTTVGFTARRAASVRR
SGRFHDQLNKNFIAF
>AtALA12
MATVSGRRRKRKIQLSKLFTLTGAKACFKPDHSKIGRSGFSRVVFCNQPDSPEAESRNYCDNYVRT
TKYTLATFLPKSLFEQFRRVANFYFLVVGILSFTPLAPYTAVSAIVPLTFVILATMFKEGVEDWRR
KQQDIEVNNRKVRVHRGNGNFDLREWKTLRVGDILKVEKNEFFPADLVLLSSSYEDAVCYVETMNL
DGETNLKLKQGLEVTLSLREELNFRDFEAFIKCEDPNANLYSFVGTMDLKGEKYPLSPQQLLLRGS
KLRNTDYIYGVVIFTGPDTKVVQNSTDPPSKRSMIERKMDKIIYLMFLMVFSLAFFGSVLFGIWTR
DDFQNGVMERWYLKPDDSSIFFDPKRAPMAAIYHFLTALMLNSYFIPISLYVSIEIVKVLQSIFIN

QDIHMYYEEADKPAHARTSNLNEELGQVGTILSDKTGTLTCNSMEFIKCSIAGTAYGRGVTEVEMA
MDKRKGSALVNQSNGNSTEDAVAAEPAVKGFNFRDERIMDGNWVTETHADVIQKFFQLLAVCHTVI
PEVDEDTGKISYEAESPDEAAFVIAARELGFEFFTRTQTTISVRELDLVTGERVERLYSVLNVLEF
SSSKKRMSVIVQDQDGKLLLLCKGADSVMFERLSESGRKYEKETRDHVNEYADAGLRTLILAYREL
DENEYEVFTERISEAKNSVSADREALIDEVTEKIEKNLVLLGATAVEDKLQNGVPDCINKLAQAGI
KIWVLTGDKMETAINIGFACSLLRRDMKQIIINLETPEIQQLEKSGEKDAIAALKENVLHQITSGK
AQLKASGGNAKAFALIIDGKSLAYALEEDMKGIFLELAIGCASVICCRSSPKQKALVTRLVKTGSG
QTTLAIGDGANDVGMLQEADIGVGISGVEGMQAVMSSDIAIAQFRYLERLLLVHGHWCYRRISKMI
CYFFYKNITFGFTLFLYEAYTSFSATPAYNDWYLSLYSVFFTSLPVICLGIFDQDVSAPFCLKFPV
LYQEGVQNLLFSWRRILSWMFHGFCSAIIIFFLCKTSLESQAFNHEGKTAGRDILGGTMYTCVVWV
VSLQMVLTISYFTLIQHVVVWGSVVIWYLFLMVYGSLPIRMSTDAYMVFLEALAPAPSYWITTLFV
VLSTMMPYFIFSAIQMRFFPMSHGTVQLLRYEDQCSNSGNFEMGRQGSVRPTLVMRSHQPES
>AtHMA1
MEPATLTRSSSLTRFPYRRGLSTLRLARVNSFSILPPKTLLRQKPLRISASLNLPPRSIRLRAVED
HHHDHHHDDEQDHHNHHHHHHQHGCCSVELKAESKPQKMLFGFAKAIGWVRLANYLREHLHLCCSA
AAMFLAAAVCPYLAPEPYIKSLQNAFMIVGFPLVGVSASLDALMDIAGGKVNIHVLMALAAFASVF
MGNALEGGLLLAMFNLAHIAEEFFTSRSMVDVKELKESNPDSALLIEVHNGNVPNISDLSYKSVPV
HSVEVGSYVLVGTGEIVPVDCEVYQGSATITIEHLTGEVKPLEAKAGDRVPGGARNLDGRMIVKAT
KAWNDSTLNKIVQLTEEAHSNKPKLQRWLDEFGENYSKVVVVLSLAIAFLGPFLFKWPFLSTAACR
GSVYRALGLMVAASPCALAVAPLAYATAISSCARKGILLKGAQVLDALASCHTIAFDKTGTLTTGG
LTCKAIEPIYGHQGGTNSSVITCCIPNCEKEALAVAAAMEKGTTHPIGRAVVDHSVGKDLPSIFVE
SFEYFPGRGLTATVNGVKTVAEESRLRKASLGSIEFITSLFKSEDESKQIKDAVNASSYGKDFVHA
ALSVDQKVTLIHLEDQPRPGVSGVIAELKSWARLRVMMLTGDHDSSAWRVANAVGITEVYCNLKPE
DKLNHVKNIAREAGGGLIMVGEGINDAPALAAATVGIVLAQRASATAIAVADILLLRDNITGVPFC
VAKSRQTTSLVKQNVALALTSIFLAALPSVLGFVPLWLTVLLHEGGTLLVCLNSVRGLNDPSWSWK
QDIVHLINKLRSQEPTSSSSNSLSSAH
>AtHMA2
MASKKMTKSYFDVLGICCTSEVPLIENILNSMDGVKEFSVIVPSRTVIVVHDTLILSQFQIVKALN
QAQLEANVRVTGETNFKNKWPSPFAVVSGILLLLSFFKYLYSPFRWLAVAAVVAGIYPILAKAVAS
LARFRIDINILVVVTVGATIGMQDYTEAAVVVFLFTIAEWLQSRASYKASAVMQSLMSLAPQKAVI
AETGEEVEVDELKTNTVIAVKAGETIPIDGVVVDGNCEVDEKTLTGEAFPVPKLKDSTVWAGTINL
NGYITVNTTALAEDCVVAKMAKLVEEAQNSKTETQRFIDKCSKYYTPAIILISICFVAIPFALKVH
NLKHWVHLALVVLVSACPCGLILSTPVATFCALTKAATSGLLIKGADYLETLAKIKIVAFDKTGTI
TRGEFIVMDFQSLSEDISLQSLLYWVSSTESKSSHPMAAAVVDYARSVSVEPKPEAVEDYQNFPGE
GIYGKIDGKEVYIGNKRIASRAGCLSVPDIDVDTKGGKTIGYVYVGETLAGVFNLSDACRSGVAQA
MKELKSLGIKIAMLTGDNHAAAMHAQEQLGNAMDIVRAELLPEDKSEIIKQLKREEGPTAMVGDGL
NDAPALATADIGISMGVSGSALATETGNIILMSNDIRRIPQAIKLAKRAKRKVVENVVISITMKGA
ILALAFAGHPLIWAAVLADVGTCLLVILNSMLLLSDKHKTGNKCYRESSSSSVLIAEKLEGDAAGD
MEAGLLPKISDKHCKPGCCGTKTQEKAMKPAKASSDHSHSGCCETKQKDNVTVVKKSCCAEPVDLG
HGHDSGCCGDKSQQPHQHEVQVQQSCHNKPSGLDSGCCGGKSQQPHQHELQQSCHDKPSGLDIGTG
PKHEGSSTLVNLEGDAKEELKVLVNGFCSSPADLAITSLKVKSDSHCKSNCSSRERCHHGSNCCRS
YAKESCSHDHHHTRAHGVGTLKEIVIE
>AtHMA3
MAEGEESKKMNLQTSYFDVVGICCSSEVSIVGNVLRQVDGVKEFSVIVPSRTVIVVHDTFLISPLQ
IVKALNQARLEASVRPYGETSLKSQWPSPFAIVSGVLLVLSFFKYFYSPLEWLAIVAVVAGVFPIL
AKAVASVTRFRLDINALTLIAVIATLCMQDFTEAATIVFLFSVADWLESSAAHKASIVMSSLMSLA
PRKAVIADTGLEVDVDEVGINTVVSVKAGESIPIDGVVVDGSCDVDEKTLTGESFPVSKQRESTVM
AATINLNGYIKVKTTALARDCVVAKMTKLVEEAQKSQTKTQRFIDKCSRYYTPAVVVSAACFAVIP
VLLKVQDLSHWFHLALVVLVSGCPCGLILSTPVATFCALTKAATSGFLIKTGDCLETLAKIKIVAF

DKTGTITKAEFMVSDFRSLSPSINLHKLLNWVSSIECKSSHPMAAALIDYAISVSVEPKPDIVENF
QNFPGEGVYGRIDGQDIYIGNKRIAQRAGCLTDNVPDIEATMKRGKTIGYIYMGAKLTGSFNLLDG
CRYGVAQALKELKSLGIQTAMLTGDNQDAAMSTQEQLENALDIVHSELLPQDKARIIDDFKIQGPT
MMVGDGLNDAPALAKADIGISMGISGSALATETGDIILMSNDIRKIPKGMRLAKRSHKKVIENVVL
SVSIKGAIMVLGFVGYPLVWAAVLADAGTCLLVILNSMILLRDEREAVSTCYRSSTSSPVKLEEDE
VEDLEVGLLQKSEETSKKSCCSGCCSGPKDNQQK

>AtHMA4

MALQNKEEEKKKVKKLQKSYFDVLGICCTSEVPIIENILKSLDGVKEYSVIVPSRTVIVVHDSLLI
SPFQIAKALNEARLEANVRVNGETSFKNKWPSPFAVVSGLLLLLSFLKFVYSPLRWLAVAAVAAGI
YPILAKAFASIKRPRIDINILVIITVIATLAMQDFMEAAAVVFLFTISDWLETRASYKATSVMQSL
MSLAPQKAIIAETGEEVEVDEVKVDTVVAVKAGETIPIDGIVVDGNCEVDEKTLTGEAFPVPKQRD
STVWAGTINLNGYICVKTTSLAGDCVVAKMAKLVEEAQSSKTKSQRLIDKCSQYYTPAIILVSACV
AIVPVIMKVHNLKHWFHLALVVLVSGCPCGLILSTPVATFCALTKAATSGLLIKSADYLDTLSKIK
IVAFDKTGTITRGEFIVIDFKSLSRDINLRSLLYWVSSVESKSSHPMAATIVDYAKSVSVEPRPEE
VEDYQNFPGEGIYGKIDGNDIFIGNKKIASRAGCSTVPEIEVDTKGGKTVGYVYVGERLAGFFNLS
DACRSGVSQAMAELKSLGIKTAMLTGDNQAAAMHAQEQLGNVLDVVHGDLLPEDKSRIIQEFKKEG
PTAMVGDGVNDAPALATADIGISMGISGSALATQTGNIILMSNDIRRIPQAVKLARRARRKVVENV
CLSIILKAGILALAFAGHPLIWAAVLVDVGTCLLVIFNSMLLLREKKKIGNKKCYRASTSKLNGRK
LEGDDDYVVDLEAGLLTKSGNGQCKSSCCGDKKNQENVVMMKPSSKTSSDHSHPGCCGDKKEEKVK
PLVKDGCCSEKTRKSEGDMVSLSSCKKSSHVKHDLKMKGGSGCCASKNEKGKEVVAKSCCEKPKQQ
VESVGDCKSGHCEKKKQAEDIVVPVQIIGHALTHVEIELQTKETCKTSCCDSKEKVKETGLLLSSE
NTPYLEKGVLIKDEGNCKSGSENMGTVKQSCHEKGCSDEKQTGEITLASEEETDDQDCSSGCCVNE
GTVKQSFDEKKHSVLVEKEGLDMETGFCCDAKLVCCGNTEGEVKEQCRLEIKKEEHCKSGCCGEEI
QTGEITLVSEEETESTNCSTGCCVDKEEVTQTCHEKPASLVVSGLEVKKDEHCESSHRAVKVETCC
KVKIPEACASKCRDRAKRHSGKSCCRSYAKELCSHRHHHHHHHHHHHVSA

>AtHMA5

MATKLLSLTCIRKERFSERYPLVRKHLTRSRDGGGGSSSETAAFEIDDPISRAVFQVLGMTCSACA
GSVEKAIKRLPGIHDAVIDALNNRAQILFYPNSVDVETIRETIEDAGFEASLIENEANERSRQVCR
IRINGMTCTSCSSTIERVLQSVNGVQRAHVALAIEEAEIHYDPRLSSYDRLLEEIENAGFEAVLIS
TGEDVSKIDLKIDGELTDESMKVIERSLEALPGVQSVEISHGTDKISVLYKPDVTGPRNFIQVIES
TVFGHSGHIKATIFSEGGVGRESQKQGEIKQYYKSFLWSLVFTVPVFLTAMVFMYIPGIKDLLMFK
VINMLTVGEIIRCVLATPVQFVIGWRFYTGSYKALRRGSANMDVLIALGTNAAYFYSLYTVLRAAT
SPDFKGVDFFETSAMLISFIILGKYLEVMAKGKTSQAIAKLMNLAPDTAILLSLDKEGNVTGEEEI
DGRLIQKNDVIKIVPGAKVASDGYVIWGQSHVNESMITGEARPVAKRKGDTVIGGTLNENGVLHVK
VTRVGSESALAQIVRLVESAQLAKAPVQKLADRISKFFVPLVIFLSFSTWLAWFLAGKLHWYPESW
IPSSMDSFELALQFGISVMVIACPCALGLATPTAVMVGTGVGASQGVLIKGGQALERAHKVNCIVF
DKTGTLTMGKPVVVKTKLLKNMVLREFYELVAATEVNSEHPLAKAIVEYAKKFRDDEENPAWPEAC
DFVSITGKGVKATVKGREIMVGNKNLMNDHKVIIPDDAEELLADSEDMAQTGILVSINSELIGVLS
VSDPLKPSAREAISILKSMNIKSIMVTGDNWGTANSIAREVGIDSVIAEAKPEQKAEKVKELQAAG
HVVAMVGDGINDSPALVAADVGMAIGAGTDIAIEAADIVLMKSNLEDVITAIDLSRKTFSRIRLNY
VWALGYNLMGIPIAAGVLFPGTRFRLPPWIAGAAMAASSVSVVCCSLLLKNYKRPKKLDHLEIREI
QVERV

>AtHMA6

MESTLSAFSTVKATAMARSSGGPSLPLLTISKALNRHFTGARHLHPLLLARCSPSVRRLGGFHGSR
FTSSNSALRSLGAAVLPVIRHRLECLSSSSPSFRSISSGGGSGFGGYNGGSGGGGGGGSESGDSKS
KLGANASDGVSVPSSDIIILDVGGMTCGGCSASVKKILESQPQVASASVNLTTETAIVWPVPEAKS
VPDWQKSLGETLANHLTNCGFQSTPRDLVTENFFKVFETKTKDKQARLKESGRELAVSWALCAVCL
VGHLTHFLGVNAPWIHAIHSTGFHVSLCLITLLGPGRKLVLDGIKSLLKGSPNMNTLVGLGALSSF
SVSSLAAMIPKLGWKTFFEEPVMLIAFVLLGRNLEQRAKIKATSDMTGLLSVLPSKARLLLDGDLQ

NSTVEVPCNSLSVGDLVVILPGDRVPADGVVKSGRSTIDESSFTGEPLPVTKESGSQVAAGSINLN
GTLTVEVHRSGGETAVGDIIRLVEEAQSREAPVQQLVDKVAGRFTYGVMALSAATFTFWNLFGAHV
LPSALHNGSPMSLALQLSCSVLVVACPCALGLATPTAMLVGTSLGARRGLLLRGGDILEKFSLVDT
VVFDKTGTLTKGHPVVTEVIIPENPRHNLNDTWSEVEVLMLAAAVESNTTHPVGKAIVKAARARNC
QTMKAEDGTFTEEPGSGAVAIVNNKRVTVGTLEWVKRHGATGNSLLALEEHEINNQSVVYIGVDNT
LAAVIRFEDKVREDAAQVVENLTRQGIDVYMLSGDKRNAANYVASVVGINHERVIAGVKPAEKKNF
INELQKNKKIVAMVGDGINDAAALASSNVGVAMGGGAGAASEVSPVVLMGNRLTQLLDAMELSRQT
MKTVKQNLWWAFGYNIVGIPIAAGVLLPLTGTMLTPSMAGALMGVSSLGVMTNSLLLRYRFFSNRN
DKNVKPEPKEGTKQPHENTRWKQSS
>AtHMA7
MAPSRRDLQLTPVTGGSSSQISDMEEVGLLDSYHNEANADDILTKIEEGRDVSGLRKIQVGVTGMT
CAACSNSVEAALMNVNGVFKASVALLQNRADVVFDPNLVKEEDIKEAIEDAGFEAEILAEEQTQAT
LVGQFTIGGMTCAACVNSVEGILRDLPGVKRAVVALSTSLGEVEYDPNVINKDDIVNAIEDAGFEG
SLVQSNQQDKLVLRVDGILNELDAQVLEGILTRLNGVRQFRLDRISGELEVVFDPEVVSSRSLVDG
IEEDGFGKFKLRVMSPYERLSSKDTGEASNMFRRFISSLVLSIPLFFIQVICPHIALFDALLVWRC
GPFMMGDWLKWALVSVIQFVIGKRFYVAAWRALRNGSTNMDVLVALGTSASYFYSVGALLYGAVTG
FWSPTYFDASAMLITFVLLGKYLESLAKGKTSDAMKKLVQLTPATAILLTEGKGGKLVGEREIDAL
LIQPGDTLKVHPGAKIPADGVVVWGSSYVNESMVTGESVPVSKEVDSPVIGGTINMHGALHMKATK
VGSDAVLSQIISLVETAQMSKAPIQKFADYVASIFVPVVITLALFTLVGWSIGGAVGAYPDEWLPE
NGTHFVFSLMFSISVVVIACPCALGLATPTAVMVATGVGATNGVLIKGGDALEKAHKVKYVIFDKT
GTLTQGKATVTTTKVFSEMDRGEFLTLVASAEASSEHPLAKAIVAYARHFHFFDESTEDGETNNKD
LQNSGWLLDTSDFSALPGKGIQCLVNEKMILVGNRKLMSENAINIPDHVEKFVEDLEESGKTGVIV
AYNGKLVGVMGIADPLKREAALVVEGLLRMGVRPIMVTGDNWRTARAVAKEVGIEDVRAEVMPAGK
ADVIRSLQKDGSTVAMVGDGINDSPALAAADVGMAIGAGTDVAIEAADYVLMRNNLEDVITAIDLS
RKTLTRIRLNYVFAMAYNVVSIPIAAGVFFPVLRVQLPPWAAGACMALSSVSVVCSSLLLRRYKKP
RLTTVLKITTE
>AtHMA8
MASNLLRFPLPPPSSLHIRPSKFLVNRCFPRLRRSRIRRHCSRPFFLVSNSVEISTQSFESTESSI
ESVKSITSDTPILLDVSGMMCGGCVARVKSVLMSDDRVASAVVNMLTETAAVKFKPEVEVTADTAE
SLAKRLTESGFEAKRRVSGMGVAENVKKWKEMVSKKEDLLVKSRNRVAFAWTLVALCCGSHTSHIL
HSLGIHIAHGGIWDLLHNSYVKGGLAVGALLGPGRELLFDGIKAFGKRSPNMNSLVGLGSMAAFSI
SLISLVNPELEWDASFFDEPVMLLGFVLLGRSLEERAKLQASTDMNELLSLISTQSRLVITSSDNN
TPVDSVLSSDSICINVSVDDIRVGDSLLVLPGETFPVDGSVLAGRSVVDESMLTGESLPVFKEEGC
SVSAGTINWDGPLRIKASSTGSNSTISKIVRMVEDAQGNAAPVQRLADAIAGPFVYTIMSLSAMTF
AFWYYVGSHIFPDVLLNDIAGPDGDALALSLKLAVDVLVVSCPCALGLATPTAILIGTSLGAKRGY
LIRGGDVLERLASIDCVALDKTGTLTEGRPVVSGVASLGYEEQEVLKMAAAVEKTATHPIAKAIVN
EAESLNLKTPETRGQLTEPGFGTLAEIDGRFVAVGSLEWVSDRFLKKNDSSDMVKLESLLDHKLSN
TSSTSRYSKTVVYVGREGEGIIGAIAISDCLRQDAEFTVARLQEKGIKTVLLSGDREGAVATVAKN
VGIKSESTNYSLSPEKKFEFISNLQSSGHRVAMVGDGINDAPSLAQADVGIALKIEAQENAASNAA
SVILVRNKLSHVVDALSLAQATMSKVYQNLAWAIAYNVISIPIAAGVLLPQYDFAMTPSLSGGLMA
LSSIFVVSNSLLLQLHKSETSKNSL
>AtP5
MSSFRVGGKVVEKVDLCRKKQLVWRLDVWPFAILYTVWLTTIVPSIDFSDACIALGGLSAFHILVL
LFTTWSVDFKCFVQFSKVNSIDQADACKVTPAKFSGSKEVVPLHFRSQMTDSASSGDMEEIFFDFR
KQRFIYSKELGAFSKLPYPTKETFGHYLKCTGHGTEAKIATATEKWGRNVFDYPQPTFQKLMKENC
MEPFFVFQVFCVGLWCLDEFWYYSVFTLFMLFMFESTMAKSRLKTLTDLRSVRVDSQTVMVYRSGK
WVKLLGTDLLPGDVVSIGRPSTQTGGEDKTVPADMLLLVGSAIVNEAILTGESTPQWKVPIVGQRS
DEKLSIKRNKNHVLFGGTKILQHSPDKSFSLKTPDGGCLAVVLRTGFETSQGKLMRTILFSTERVT
ANSWESGLFILFLVVFAVIAAGYVLVKGLEDPTRSKYKLLLGCSLIITSVIPPELPMELSIAVNTS

LLALVRRGIFCTEPFRIPFAGKVDLCCFDKTGTLTSDDMEFRGVGGLSNCEEAETDMSKVPVRTLE
ILASCHALVFVENKLVGDPLEKAALKGIDWSYKADEKALPRRGNGNSVQIMQRYHFASHLKRMSVI
VRIQEEYLAFVKGAPETIQERLVDVPAQYIETYKRYTRQGSRVLALAYKRLPDMMVSEARDMDRDA
VESDLTFAGFAVFNCPIRPDSAPVLLELKNSSHDLVMITGDQALTACHVAGQVHIVSNPVLILGRS
GSGNEYKWVSPDEKEIIPYSEKEIETLAETHDLCIGGDSIEMLQATSAVLRVIPFVKVFARVAPQQ
KELILTTFKAVGRGTLMCGDGTNDVGALKQAHVGVALLNNKLPLSPSDSSKDDKSKSKKSKLPLEP
ASKTITQNGEGSSKGKIPPQNRHLTAAELQRQKLKKIMDDLNNDEGDGRSAPLVKLGDASMASPFT
AKHASVAPVTDIIRQGRSTLVTTLQMFKILGLNCLATAYVLSVMYLDGVKLGDVQATISGVLTAAF
FLFISHARPLQTLSAERPHPSVFSVYLFLSLIGQFAVHLTFLVYSVKEAEKHMPEECIEPDASFHP
NLVNTVSYMVSMMLQVATFAVNYMGHPFNQSIRENKPFFYALIAGAGFFTVIASDLFRDLNDSLKL
VPLPQGLRDKLLIWASLMFIICYSWERLLRWAFPGKISSWKHKQRAVTANLEKKKKV
