# Supplementary material for: A two-sequence motif-based method for the inventory of gene families in fragmented and poorly annotated genome sequences
Source: BMC Genomics. 2024 Jan 3;25:26. doi: 10.1186/s12864-023-09859-4 (PMC10763278; doi:10.1186/s12864-023-09859-4)
Supplement: Supplementary file 14 — Additional file 14: Supplementary Figure 1. Alignment of Heavy Metal ATPases (P1B ATPases) from A. thaliana, O. sativa, and H. vulgare. Supplementary Figure 2. Alignment of P2A ATPases from A. thaliana, O. sativa, and H. vulgare. Supplementary Figure 3. Alignment of P2B ATPases from A. thaliana, O. sativa, and H. vulgare. Supplementary Figure 4. Alignment of P3A ATPases from A. thaliana, O. sativa, and H. vulgare. Supplementary Figure 5. Alignment of P4 ATPases from A. thaliana, O. sativa, and H. vulgare. Supplementary Figure 6. Alignment of P5 ATPases from A. thaliana, O. sativa, and H. vulgare. [file 12864_2023_9859_MOESM14_ESM.pdf]

```
                    CPCXXXXXXXXXXXXXXXXXXXXXXXXXXXXXXXXXXXXXXXXXDKTGT
                     **      * *           *                    ***** *
Consensus      VLVIACPCALGLATPTAVMVATGVGAXRGLLIKGGDVLERLAKXKAIAFDKTGTLTRG   481
AtHMA1         LMVAASPCALAVA-PLAYATAISSCARKGILLKGAQVLDALASCHTIAFDKTGTLTTG   460
AtHMA2         VLVSACPCGLILSTPVATFCALTKAATSGLLIKGADYLETLAKIKIVAFDKTGTITRG   384
AtHMA3         VLVSGCPCGLILSTPVATFCALTKAATSGFLIKTGDCLETLAKIKIVAFDKTGTITKA   390
AtHMA4         VLVSGCPCGLILSTPVATFCALTKAATSGLLIKSADYLDTLSKIKIVAFDKTGTITRG   394
AtHMA5         VMVIACPCALGLATPTAVMVGTGVGASQGVLIKGGQALERAHKVNCIVFDKTGTLTMG   653
AtHMA6         VLVVACPCALGLATPTAMLVGTSLGARRGLLLRGGDILEKFSLVDTVVFDKTGTLTKG   598
AtHMA7         VVVIACPCALGLATPTAVMVATGVGATNGVLIKGGDALEKAHKVKYVIFDKTGTLTQG   650
AtHMA8         VLVVSCPCALGLATPTAILIGTSLGAKRGYLIRGGDVLERLASIDCVALDKTGTLTEG   539
OsHMA1         LMVAASPCALAVA-PLAYATAISSLASKGILLKGGHVLDALSACQSIAFDKTGTLTTG   428
OsHMA2         LLVSACPCALVLSTPIATFCALLRAARTGLLIKGGDVLESLASIKVAAFDKTGTITRG   388
OsHMA3         MLVSACPCALVLSTPVASFCAMLRAARMGIFIKGGDVLESLGEIRAVAFDKTGTITRG   420
OsHMA4         VLVVACPCALGLATPTAVMVATGKGASQGVLIKGGNALEKAHKVKAIIFDKTGTLTVG   633
OsHMA5         VMVIACPCALGLATPTAVMVATGVGASQGVLIKGGQALESAQKVDCIVFDKTGTLTIG   673
OsHMA6         VVVIACPCALGLATPTAVMVATGVGANHGVLVKGGDALERAQNVKYVIFDKTGTLTQG   649
OsHMA7         VLVIACPCALGLATPTAVLVGTSLGATRGLLLRGGDILEKFSEVDAIVFDKTGTLTIG   561
OsHMA8         VLVVSCPCALGLATPTAILIGTSLGAKRGLLIRGGDVLERLAGIDAIVLDKTGTLTKG   562
OsHMA9         VVVIACPCALGLATPTAVMVATGVGANHGVLVKGGDALERAQNVNYVIFDKTGTLTQG   650
HvHMA1         LMVAASPCALAVA-PLAYATAISSLASKGILLKGGHVLDALSSCQSIAFDKTGTLTTG   464
HvHMA2         LLVSACPCALVLSTPVATFCALLRAARTGLLIKGGDVLESLASIKVAAFDKTGTITRG   393
HvHMA2-like    LLVSACPCALVLSTPVATFCALLMAARTGLLIKGGDVLESLAGIKVAAFDKTGTITSG   385
HvHMA3         LLVSACPCALVLSTPVATFCALLTAARMGLLVKGGDVLESLGEIKAVAFDKTGTITRG   406
HvHMA4         VLVVACPCALGLATPTAVMVATGKGASLGVLIKGGNALEKAHKIKTIIFDKTGTLTKG   632
HvHMA5         VMVIACPCALGLATPTAVMVATGVGASQGILIKGGQALESAQKVDCIIFDKTGTLTIG   662
HvHMA6         VVVIACPCALGLATPTAVMVATGVGANHGVLVKGGDALERAQNVKYIIFDKTGTLTQG   648
HvHMA7         VLVIACPCALGLATPTAVLVGTSLGATRGLLLRGGDVLEKFAEVDAIVFDKTGTLTIG   536
HvHMA8         VLVVSCPCALGLATPTAILIGTSMGAKRGLLIRGGDVLERLAGIDAIVLDKTGTLTKG   454
HvHMA9         VVVIACPCALGLATPTAVMVATGIGANHGVLVKGGDALERAQNVNYVIFDKTGTLTQG   649
```

Supplementary Figure 1. **Alignment of Heavy Metal ATPases (P1B ATPases) from *A. thaliana*, *O. sativa*, and *H. vulgare*.** The alignment depicts the area used for searching genomes. The number on the right side is the last amino acid for each sequence in the alignment. The

top shows the query string where only the subgroup and P-type ATPase are used with a specific distance between the two. To create a bait that could identify family members in which an intron was between the two motifs, the consensus sequence (marked in blue) surrounding the two was used. The subgroup and ATPase motifs are marked in orange. All mismatches to the consensus sequence are marked in black.

```
                            PEXLXXXXXXXXXXXXXXXXXXXXXXXXXXXXXXXXXXXXXXXDKTGT
               ** ***************** ********  ****** ** ************************
Consensus      YFEIAVALAVAAIPEGLPAVITTCLALGTRKMAQKNALVRKLPSVETLGCTTVICSDKTGTLTTN   371
AtECA1         YFEIAVALAVAAIPEGLPAVITTCLALGTRKMAQKNALVRKLPSVETLGCTTVICSDKTGTLTTN   389
AtECA2         YFKIAVALAVAAIPEGLPAVITTCLALGTRKMAQKNAIVRKLPSVETLGCTTVICSDKTGTLTTN   374
AtECA3         YFKIAVALAVAAIPEGLPAVVTTCLALGTKKMARLNAIVRSLPSVETLGCTTVICSDKTGTLTTN   355
AtECA4         YFEIAVALAVAAIPEGLPAVITTCLALGTRKMAQKNALVRKLPSVETLGCTTVICSDKTGTLTTN   389
OsECA1         YFEIAVALAVAAIPEGLPAVITTCLALGTRKMAQKNALVRKLPSVETLGCTTVICSDKTGTLTTN   393
OsECA2         YFEIAVALAVAAIPEGLPAVITTCLALGTRKMAAKNALVRKLPSVETLGCTTVICSDKTGTLTTN   385
OsECA3         YFKVAVALAVAAIPEGLPAVVTTCLALGTKRMARLNAIVRSLPSVETLGCTTVICSDKTGTLTTN   356
HvECA1         YFEIAVALAVAAIPEGLPAVITTCLALGTRKMAQKNALVRKLPSVETLGCTTVICSDKTGTLTTN   393
HvECA2         YFEIAVALAVAAIPEGLPAVITTCLALGTRKMAAKNALVRKLPSVETLGCTTVICSDKTGTLTTN   380
HvECA3         YFKVAVALAVAAIPEGLPAVVTTCLALGTKRMARLNAIVRSLPSVETLGCTTVICSDKTGTLTTN   355
```

Supplementary Figure 2. Alignment of P2A ATPases from *A. thaliana*, *O. sativa*, and *H. vulgare*. The alignment depicts the area used for searching genomes. The number on the right side is the last amino acid for each sequence in the alignment. The top shows the query string where only the subgroup and P-Type ATPase are used with a specific distance between the two. To create a bait that could identify family members in which an intron was between the two motifs, the consensus sequence (marked in blue) surrounding the two was used. The subgroup and ATPase motif are marked in orange. All mismatches to the consensus sequence are marked in black.

```
                            PEXLXXXXXXXXXXXXXXXXXXXXXXXXXXXXXXXXXXXXXXXDKTGT
                   **** *** ********** **  *        *** *   *****   ** ******* *
Consensus       FAIAVTIVVVAVPEGLPLAVTLSLAFAMKKMMNDKALVRHLSACETMGSATTICSDKTGTLTTNH    490
AtACA1          FAVAVTIVVVAVPEGLPLAVTLSLAFAMKKMMNDKALVRNLAACETMGSATTICSDKTGTLTTNH    465
AtACA2          FAIAVTIVVVAVPEGLPLAVTLSLAFAMKKMMNDKALVRHLAACETMGSATTICSDKTGTLTTNH    463
AtACA4          FAISVTIIVVAVPEGLPLAVTLSLAFAMKKLMSDRALVRHLAACETMGSSTCICTDKTGTLTTNH    460
AtACA7          FAIAVTIVVVAVPEGLPLAVTLSLAFAMKKMMNDKALVRHLAACETMGSATTICSDKTGTLTTNH    464
AtACA8          LTVAVTIVVVAVPEGLPLAVTLTLAYSMRKMMADKALVRRLSACETMGSATTICSDKTGTLTLNQ    491
AtACA9          FTIAVTIVVVAVPEGLPLAVTLTLAYSMRKMMADKALVRRLSACETMGSATTICSDKTGTLTLNQ    504
AtACA10         FTVAVTIVVVAVPEGLPLAVTLTLAYSMRKMMADKALVRRLSACETMGSATTICSDKTGTLTLNE    491
AtACA11         FAIAVTIIVVAVPEGLPLAVTLSLAFAMKQLMSDRALVRHLAACETMGSSTCICTDKTGTLTTNH    460
AtACA12         VAAAVTIVVVAIPEGLPLAVTLTLAYSMKRMMSDQAMVRKLSACETMGSATVICTDKTGTLTLNE    462
AtACA13         VAAAVTIIVVAIPEGLPLAVTLTLAYSMKRMMKDNAMVRKLSACETMGSATVICTDKTGTLTLNQ    458
OsACA1          FAIAVTIVVVAVPEGLPLAVTLSLAFAMKKMMNDKALVRHLAACETMGSATTICSDKTGTLTTNH    465
OsACA2          FAIAVTIVVVAVPEGLPLAVTLSLAFAMKKMMNDKALVRHLAACETMGSATTICSDKTGTLTTNH    465
OsACA3          FAVAVTIVVVAVPEGLPLAVTLSLAFAMKKMMNDKALVRQLAACETMGSATVICSDKTGTLTTNR    470
OsACA4          FATAVTIIVVAVPEGLPLAVTLSLAFAMKKLMNDKALVRHLSACETMGSAGTICTDKTGTLTTNY    464
OsACA5          FATAVTIIVVAVPEGLPLAVTLSLAFAMKKLMNDKALVRHLSACETMGSAGTICTDKTGTLTTNH    460
OsACA6          FAVAVTIIVVAVPEGLPLAVTLSLAFAMKKLMQERALVRHLSACETMGSASCICTDKTGTLTTNH    461
OsACA7          FAIAVTIIVVAVPEGLPLAVTLSLAFAMKKLMHDKALVRHLAACETMGSASCICTDKTGTLTTNH    467
OsACA8          FQQAVTIIVVAIPEGLPLAVTLTLAFSMKRMVRENALVRRLSACETMGSVTAICTDKTGTLTLNQ    469
OsACA9          LTIAVTIVVVAVPEGLPLAVTLTLAYSMQKMMADKALVRRLSACETMGSATTICSDKTGTLTLNQ    481
OsACA10         LTIAVTIVVVAVPEGLPLAVTLTLAYSMRKMMRDKALVRRLSSCETMGSATTICSDKTGTLTLNK    494
OsACA11         FTVAVTIVVVAVPEGLPLAVTLTLAFSMRKMMRDKALVRRLSACETMGSATTICSDKTGTLTLNQ    495
HvACA1          FAIAVTIVVVAVPEGLPLAVTLSLAFAMKKMMNDKALVRHLAACETMGSATSICSDKTGTLTTNH    465
HvACA2          FAIAVTIVVVAVPEGLPLAVTLSLAFAMKKMMNDKALVRNLAACETMGSATTICSDKTGTLTTNH    465
HvACA3          FAVAVTIVVVAVPEGLPLAVTLSLAYAMEKMMNDKALVRQLAACETMGSATVICSDKTGTLTSNR    468
HvACA4_5        FATAVTIIVVAVPEGLPLAVTLSLAFAMKKLMNDKALVRHLAACETMGSAGTICTDKTGTLTTNH    463
HvACA6          FAVAVTIIVVAVPEGLPLAVTLSLAFAMKKLMQERALVRHLSACETMGSASCICTDKTGTLTTNH    461
HvACA7          FAIAVTIIVVAVPEGLPLAVTLSLAFAMKKLMNDKALVRHLAACETMGSVSCICTDKTGTLTTNH    471
HvACA8          FQQAVTIIVVAIPEGLPLAVTLTLAFSMKRMVKENALVRRLSACETMGSVTAICTDKTGTLTLNQ    465
HvACA9          LTVAVTIVVVAVPEGLPLAVTLTLAYSMRKMMADKALVRRLSACETMGSATTICSDKTGTLTLNQ    506
HvACA10         FTIAVTIVVVAVPEGLPLAVTLTLAYSMRKMMRDKALVRRLSSCETMGSATTICSDKTGTLTLNK    490
HvACA10-like    FTVAVTIVVVAVPEGLPLAVTLTLAYSMRKMMRDKALVRRLSSCETMGSATTICSDKTGTLTLNK    498
HvACA11         LTIAVTIVVVAVPEGLPLAVTLTLAFSMRKMMRDKALVRRLSACETMGSATTICSDKTGTLTLNQ    490
```

Supplementary Figure 3. Alignment of P2B ATPases from *A. thaliana*, *O. sativa*, and *H. vulgare*. The alignment depicts the area used for searching genomes. The number on the right side is the last amino acid for each sequence in the alignment. The top shows the query string where only the

subgroup and P-type ATPase are used with a specific distance between the two. To create a bait that could identify family members in which an intron was between the two motifs, the consensus sequence (marked in blue) surrounding the two was used. The subgroup and ATPase motifs are marked in orange. All mismatches to the consensus sequence are marked in black.

```
                    PIAXXXXXXXXXXXXXXXXXXXXXXXXXXXXXXXXXXXXXXXXDKTGT
          * ************* ***** * * * *  ************* * ***** ********* *
Consensus NLLVLLIGGIPIAMPTVLSVTMAIGSHRLSQQGAITKRMTAIEEMAGMDVLCSDKTGTLTLNKL   358
AtAHA1    NLLVLLIGGIPIAMPTVLSVTMAIGSHRLSQQGAITKRMTAIEEMAGMDVLCSDKTGTLTLNKL   339
AtAHA2    NLLVLLIGGIPIAMPTVLSVTMAIGSHRLSQQGAITKRMTAIEEMAGMDVLCSDKTGTLTLNKL   339
AtAHA3    NLLVLLIGGIPIAMPTVLSVTMAIGSHKLSQQGAITKRMTAIEEMAGMDVLCSDKTGTLTLNKL   340
AtAHA4    NLLVLLIGGIPIAMPTVLSVTMAIGSHRLSQQGAITKRMTAIEEMAGMDVLCSDKTGTLTLNKL   347
AtAHA5    NLLVLLIGGIPIAMPSVLSVTMATGSHRLFQQGAITKRMTAIEEMAGMDVLCCDKTGTLTLNKL   339
AtAHA6    NLLVLLIGGIPIAMPTVLSVTMAIGSHRLSQQGAITKRMTAIEEMAGMDVLCSDKTGTLTLNKL   342
AtAHA7    NLLVLLIGGIPIAMPTVLSVTMAIGAHRLAQQGAITKRMTAIEEMAGMDVLCSDKTGTLTLNKL   342
AtAHA8    NLLVLLIGGIPIAMPTVLSVTMAIGSHRLSQQGAITKRMTAIEEMAGMDVLCSDKTGTLTLNKL   342
AtAHA9    NLLVLLIGGIPIAMPTVLSVTMAIGSHRLSQQGAITKRMTAIEEMAGMDVLCSDKTGTLTLNKL   344
AtAHA10   NLLVLLIGGIPIAMPTVLSVTLAIGSHRLSQQGAITKRMTAIEEMAGMDVLCCDKTGTLTLNSL   347
AtAHA11   NLLVLLIGGIPIAMPTVLSVTMAIGSHRLSQQGAITKRMTAIEEMAGMDVLCSDKTGTLTLNKL   343
OsAHA1    NLLVLLIGGIPIAMPTVLSVTMAIGSHRLSQQGAITKRMTAIEEMAGMDVLCSDKTGTLTLNKL   343
OsAHA2    NLLVLLIGGIPIAMPTVLSVTMAIGSHRLAQQGAITKRMTAIEEMAGMDVLCSDKTGTLTLNKL   343
OsAHA3    NLLVLLIGGIPIAMPTVLSVTMAIGSHRLSQQGAITKRMTAIEEMAGMDVLCSDKTGTLTLNKL   343
OsAHA4    NLLVLLIGGIPIAMPTVLSVTMAIGSHRLSQQGAITKRMTAIEEMAGMDVLCSDKTGTLTLNKL   346
OsAHA5    NLLVLLIGGIPIAMPTVLSVTMAIGSHRLSDQGAITKRMTAIEEMAAMDVLCSDKTGTLTLNKL   347
OsAHA6    NLLVLLIGGIPIAMPTVLSVTMAIGSHRLSQQGAITKRMTAIEEMAGMDVLCSDKTGTLTLNKL   341
OsAHA7    NLLVLLIGGIPIAMPTVLSVTMAIGSHKLSQQGAITKRMTAIEEMAGMDVLCSDKTGTLTLNKL   336
OsAHA8    NLLVLLIGGIPIAMPTVLSVTMAIGSHKLAQQGAITKRMTAIEEMAGMDVLCSDKTGTLTLNKL   348
OsAHA9    NVLVLLIGGIPIAMPTVLSVTLAIGSHHLSQQGAITKRMTAIEEMAGMDVLCCDKTGTLTLNHL   346
OsAHA10   NLLVLLIGGIPIAMPTVLSVTMAIGSHRLSQQGAITKRMTAIEEMAGMDVLCSDKTGTLTLNKL   338
HvAHA1    NLLVLLIGGIPIAMPTVLSVTMAIGSHRLSQQGAITKRMTAIEEMAGMDVLCSDKTGTLTLNKL   348
HvAHA3    NLLVLLIGGIPIAMPTVLSVTMAIGSHRLSQQGAITKRMTAIEEMAGMDVLCSDKTGTLTLNKL   344
HvAHA4    NLLVLLIGGIPIAMPTVLSVTMAIGSHKLSQQGAITKRMTAIEEMAGMDVLCSDKTGTLTLNKL   340
HvAHA5    NLLVLLIGGIPIAMPTVLSVTMAIGSHRLSKQGAITKRMTAIEEMAGMDVLCSDKTGTLTLNKL   345
HvAHA6    NLLVLLIGGIPIAMPTVLSVTMAIGSHRLSQQGAITKRMTAIEEMAGMDVLCSDKTGTLTLNKL   341
HvAHA7    NLLVLLIGGIPIAMPTVLSVTMAIGSHKLSQQGAITKRMTAIEELAGMDVLCSDKTGTLTLNKL   339
HvAHA8    NLLVLLIGGIPIAMPTVLSVTMAIGSHKLAQQGAITKRMTAIEEMAGMDVLCSDKTGTLTLNKL   364
HvAHA9    NVLVLLIGGIPIAMPTVLSVTLAIGSHRLSQQGAITKRMTAIEEMAGMDVLCCDKTGTLTLNHL   348
HvAHA10   NLLVLLIGGIPIAMPTVLSVTMAIGSHRLSQQGAITKRMTAIEEMAGMDVLCSDKTGTLTLNKL   341
```

Supplementary Figure 4. Alignment of P3A ATPases from *A. thaliana*, *O. sativa*, and *H. vulgare*. The alignment depicts the area used for searching genomes. The number on the right side is the last amino acid for each sequence in the alignment. The top shows the query string where only the subgroup and P-type ATPase are used with a specific distance between the two. To create a bait that could identify family members in which an intron was between the two motifs, the consensus sequence (marked in blue) surrounding the two was used. The subgroup and ATPase motifs are marked in orange. All mismatches to the consensus sequence are marked in black.

```
           PISXXXXXXXXXXXXXXXXXXXXXXXXXXXXXXXXXXXXXXXXXXXXXXXXXXXDKTGT
           ***               *   *                      **       ******      *
Consensus  PISLYVSIELVKVLQAY-FINQDIHMYDEESDTPAQARTSNLNEELGQVDTILSDKTGTLTCNKMEF   500
AtALA1     PISLYISMELVRIGQAY-FMTNDDQMYDESSDSSFQCRALNINEDLGQIKYLFSDKTGTLTDNKMEF   465
AtALA2     PISIKVSLDLVKGLYAK-FIEWDVEMIDQETGTASYAANTAISEDLGQVEYILTDKTGTLTDNKMIF   389
AtALA3     PISLYVSIEMIKFIQSTQFINRDLNMYHAETNTPASARTSNLNEELGQVEYIFSDKTGTLTRNLMEF   425
AtALA4     PISLYVSIEVVKVLQAS-FINKDLHMYDSESGVPAHARTSNLNEELGQVDTILSDKTGTLTCNQMDF   437
AtALA5     PISLYVSIEVVKVWQAS-FINQDLHMYDDESGVPANARTSNLNEELGQVHTILSDKTGTLTCNQMDF   437
AtALA6     PISLYVSIEVVKVLQAH-FINQDLQLYDSESGTPAQARTSNLNEELGQVDTILSDKTGTLTCNQMDF   438
AtALA7     PISLYVSIELVKVLQAT-FINQDLQMYDSESGTPAQARTSNLNEELGQVDTILSDKTGTLTCNQMDF   437
AtALA8     PISLYVSIEVVKVLQSI-FINQDQEMYHEETDRPARARTSNLNEELGQVDTILSDKTGTLTCNSMEF   436
AtALA9     PISLYVSIEIVKVLQSI-FINQDIHMYYEEADKPARARTSNLNEELGQVDTILSDKTGTLTCNSMEF   442
AtALA10    PISLYVSIEIVKVLQSI-FINRDIHMYYEETDKPAQARTSNLNEELGMVDTILSDKTGTLTCNSMEF   442
AtALA11    PISLYVSIEIVKVLQSL-FINNDILMYYEENDKPAHARTSNLNEELGMVDTILSDKTGTLTCNSMEF   441
AtALA12    PISLYVSIEIVKVLQSI-FINQDIHMYYEEADKPAHARTSNLNEELGQVGTILSDKTGTLTCNSMEF   442
OsALA1     PISLYITMELVRVGQSY-FMIGDTRMYDSSSGSRFQCRSLNINEDLGQIRYIFSDKTGTLTQNKMEF   442
OsALA2     PISLYISMELVRLGQAY-FMGADRDLYDESSRSKFQCRALNINEDLGQIKYVFSDKTGTLTENKMEF   475
OsALA3     PISLYISMELVRLGQAY-FMIRDTTLYDASSNSRFQCRALNINEDLGQVKCVFSDKTGTLTQNKMEF   433
OsALA4     PISLYISIEIVKLLQAL-FINQDIHMYHEETDTPAHARTSNLNEELGQVDTILTDKTGTLTCNSMEF   446
OsALA5     PISLYISIEVVKILQAL-FINQDIEMYHEESDKPTHARTSNLNEELGQVDTVLSDKTGTLTCNMMEF   445
OsALA6     PISLYISIEIVKVLQST-FINQDQNMYCEESDKPARARTSNLNEELGQVHTILSDKTGTLTCNSMEF   448
OsALA7     PISLYVSIELVKVLQAH-FINQDLHMFDEDTGNTAQARTSNLNEELGQVHTILSDKTGTLTCNQMDF   420
OsALA8     PISLYVSIEVIKFIQCTQFINNDLHMYHAESNTPALARTSNLNEELGQVEYIFSDKTGTLTRNLMEF   446
OsALA9     PISIKVSLDFVKSLYAK-FIDWDEEMYDHETDTPAHAANTAISEDLGQVEYILTDKTGTLTENKMIF   387
OsALA10    PISVKVTLDLSKGVYAK-FIDWDEQMFDRETSYISVSFSTAISEDLGQVEYVLSDKTGTLTENRMIF   388
HvALA1     PISLYITMELVRVGQSY-FMIGDTRMYDSSSGSRFQCRSLNINEDLGQIRYIFSDKTGTLTQNKMEF   556
HvALA2     PISLYISMELVRLGQAY-FMGADNDLYDGSSRSRFQCRALNINEDLGQIKYVFSDKTGTLTENKMEF   466
HvALA3     PISLYISMELVRLGQAY-FMIRDAKLYDASTDSRFQCRALNINEDLGQVKCVFSDKTGTLTQNKMEF   434
HvALA4     PISLYVSIELVKLLQAL-FINSDIHMYHEESDTPAHARTSNLNEELGQVYTILTDKTGTLTCNSMEF   461
HvALA5     PISLYISIEMVKILQAV-FINQDIEMYDEESDKPTHARTSNLNEELGQVDTILSDKTGTLTCNMMEF   442
HvALA7     PISLYVSIEVVKVAQAH-FINQDIHMFDEETGNTAQARTSNLNEELGQVHTILSDKTGTLTCNQMDF   443
HvALA8     PISLYVSIEMIKFIQCAQFINNDVNMYHAESNTPALARTSNLNEELGQVEYIFSDKTGTLTRNLMEF   444
HvALA9     PISIKVSLDFVKSMYAK-FIDWDEEMYDQETDTPAHAANTAISEDLGQVEYILTDKTGTLTENKMIF   388
HvALA10    PISIKVTLDLAKGVYAK-FIDCDDQMFDPETNTPAHSANTAISEDLGQVEYILSDKTGTLTENRMIF   388
```

Supplementary Figure 5. Alignment of P4 ATPases from *A. thaliana*, *O. sativa*, and *H. vulgare*. The alignment depicts the area used for searching genomes. The number on the right side is the last amino acid for each sequence in the alignment. The top shows the query string where only the subgroup and P-Type ATPase are used with a specific distance between the two. To create a bait that could identify family members in which an intron was between the two motifs, the consensus sequence (marked in blue) surrounding the two was used. The subgroup and ATPase motifs are marked in orange. All mismatches to the consensus sequence are marked in black.

```
                            PPXPXXXXXXXXXXXXXXXXXXXXXXXXXXXXXXXXXXXXXXXDKTGT
              ** * **** ******************** ** ******************* ****************
Consensus     KLFLSCSLILTSVIPPELPMELSIAVNTSLIALVRRGIFCTEPFRIPFAGKVDICCFDKTGTLTSDDMEF   500
AtP5          KLLLGCSLIITSVIPPELPMELSIAVNTSLLALVRRGIFCTEPFRIPFAGKVDLCCFDKTGTLTSDDMEF   503
OsP5          KLFLSCSLILTSVIPPELPMELSIAVNTSLIALARRGIFCTEPFRIPFAGKVDICCFDKTGTLTSDDMEF   495
HvP5          KLFLSCSLILTSVIPPELPMELSIAVNTSLIALVRRGIFCTEPFRIPFAGKVDICCFDKTGTLTSDDMEF   500
```

Supplementary Figure 6. Alignment of P5 ATPases from *A. thaliana*, *O. sativa*, and *H. vulgare.* The alignment depicts the area used for searching genomes. The number on the right side is the last amino acid for each sequence in the alignment. The top shows the query string where only the subgroup and P-type ATPase are used with a specific distance between the two. To create a bait that could identify family members in which an intron was between the two motifs, the consensus sequence (marked in blue) surrounding the two was used. The subgroup and ATPase motifs are marked in orange. All mismatches to the consensus sequence are marked in black.
